# Enantioselective Intermolecular C–H Functionalization of Primary Benzylic C–H Bonds Using ((Aryl)(diazo)methyl)phosphonates

**DOI:** 10.1021/acscatal.3c04661

**Published:** 2023-12-11

**Authors:** Yasir Naeem, Bianca T. Matsuo, Huw M. L. Davies

**Affiliations:** Department of Chemistry, Emory University, Atlanta, Georgia 30322, United States

**Keywords:** donor/acceptor carbenes, C−H functionalization, diazophosphonates, chiral catalyst, dirhodium tetracarboxylate

## Abstract

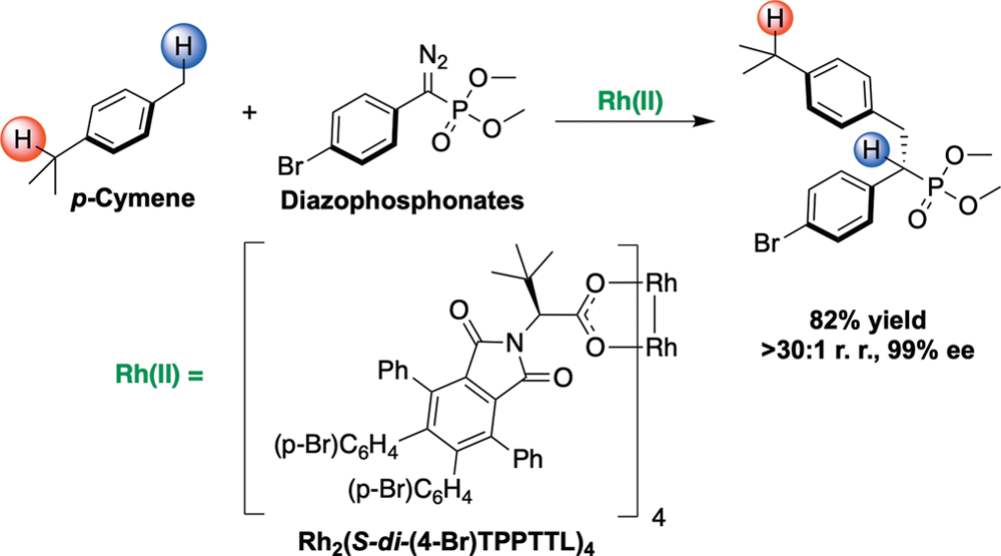

Catalyst-controlled C–H functionalization using donor/acceptor carbenes has been shown to be an efficient process capable of high levels of site control and stereocontrol. This study demonstrated that the scope of the donor/acceptor carbene C–H functionalization can be extended to systems where the acceptor group is a phosphonate. When using the optimized dirhodium catalyst, Rh_2_(*S-di*-(4-Br)TPPTTL)_4_, ((aryl)(diazo)methyl)phosphonates undergo highly enantioselective (84–99% ee) and site-selective (>30:1 r.r.) benzylic C–H functionalization. The phosphonate group is much more sterically demanding than the previously studied carboxylate ester group, leading to much higher selectivity for a primary site versus more sterically crowded positions. The effectiveness of this methodology has been demonstrated by the late-stage primary C–H functionalization of estrone, adapalene, (*S*)-naproxen, clofibrate, and gemfibrozil derivatives.

Enantioselective intermolecular sp^3^ C–H functionalization is an attractive but challenging proposition, requiring appropriate control of site-selectivity in addition to efficient asymmetric induction.^1−21^ Considerable progress in achieving synthetically useful processes has been made by using directing groups to coordinate with a metal catalyst and, thus, organize the system to react with a specific C–H bond.^1−5^ Although this approach has been highly successful, it would be desirable to achieve site-selectivity by using catalyst control without the need for a directing group. An effective approach has been to use group transfer reactions, which do not require prior coordination to directing groups.^6^ The most common of these group transfer reactions involve metal-bound carbenes,^7−10^ nitrenes,^11,12^ and oxo species,^13−15^ catalyzed by transition-metal complexes. This approach has been further refined in recent years with the use of evolved artificial enzymes with the capacity for exquisite site- and stereocontrol.^22,23^

In the carbene arena, the most selective system has been the donor/acceptor carbenes because they have several components that can be manipulated to influence the outcome of the C–H functionalization (Scheme 1).^24^ The acceptor group ensures that the carbene is sufficiently electrophilic to be capable of reacting with C–H bonds, whereas the donor group modulates the reactivity, making the system highly susceptible to catalyst control. Various chiral dirhodium catalysts have been designed to control the regio- and stereochemical outcomes of their C–H functionalization reactions.^8,25^ Most of the studies have been conducted using aryldiazoacetates as the carbene precursors, and selective reactions at primary, secondary, or tertiary C–H bonds can be achieved depending on which catalyst is used.^26−28^ Subtle control can be achieved by changing the nature of the donor and acceptor groups. For example, even though methyl esters **1** are effective carbene precursors for functionalization at activated C–H bonds (benzylic, allylic, α to oxygen or nitrogen),^9^ the trichloroethyl esters **2** are superior for reactions at unactivated C–H bonds.^26−28^ In order to further expand the scope of the C–H functionalization chemistry of donor/acceptor carbenes, we explored the use of phosphonates as the acceptor group (**3**), which opens up interesting possibilities because the resulting carbenes behave more sterically demanding than their ester counterparts.

**Scheme 1:**
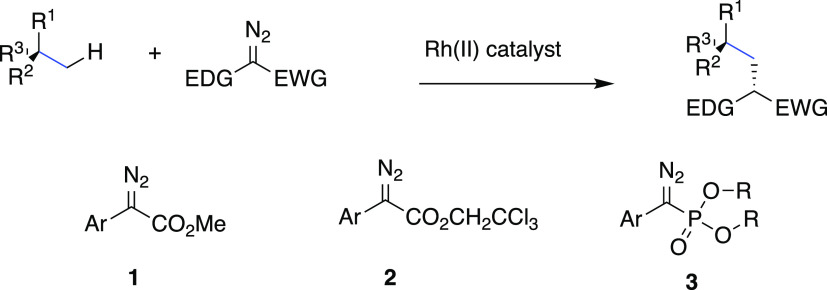
C–H Functionalization with Donor/Acceptor Carbenes

The first stage of the study was to determine the optimum chiral catalyst for C–H functionalization (Table 1). The diazophosphonate **4** is readily prepared via an Arbuzov reaction using 4-bromobenzyl bromide followed by a traditional diazo transfer reaction.^29^ In order to examine the selectivity profile of the phosphonate, *p*-cymene (**5**) with both tertiary and primary benzylic C–H bonds was used as the model substrate. The initial studies using standard reaction conditions resulted in slow reactions and poor conversions. Further examination revealed the reaction was sensitive to moisture, and in order to have an effective transformation, the reactions needed to be conducted in the presence of both activated molecular sieves and 20 equiv of HFIP. The original standard dirhodium catalyst, Rh_2_(*S*-DOSP)_4_, gave good yield (80%) but a poor level of enantioselectivity (48% ee) of the C–H functionalization product **6**, with a strong preference for the reaction occurring at the primary C–H bond (>30:1 r.r.) (Table 1, entry 1). The positive influence of HFIP is consistent with recent studies, demonstrating that in the reactions of donor/acceptor carbenes HFIP blocks interference by water^30^ and a range of other nucleophilic poisons.^31^

**Table 1:**
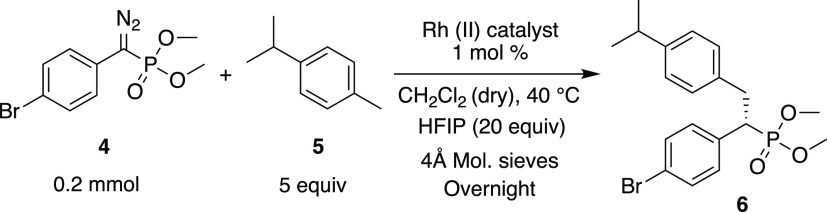
Catalyst Optimization Studies^a^

entry	catalyst	yield, %^b^	r.r.	ee, %^c^
1	Rh_2_(*R*-DOSP)_4_	80	>30:1	–48
2	Rh_2_(*S*-*p*-Br-TPCP)_4_	trace		
3	Rh_2_(*S*-PTAD)_4_	75	>30:1	83
4	Rh_2_(*R*-TCPTAD)_4_	65	>30:1	–93
5	Rh_2_(*R*-NTTL)_4_	77	>30:1	–92
6	Rh_2_(*R*-TPPTTL)_4_	81	>30:1	–92
7	Rh_2_(*S*-*tetra-*(4-Br)TPPTTL)_4_	35	>30:1	97
8	Rh_2_(*S*-*di-*(4-Br)TPPTTL)_4_	82	>30:1	99

a Reaction conditions: To a mixture of **5** (1.0 mmol) and [Rh] catalyst (1 mol %) in dry CH_2_Cl_2_ solvent (5.0 mL) was added a solution of **4** (0.2 mmol) in dry CH_2_Cl_2_ solvent (10.0 mL) via an automatic syringe pump over 5 h at 40 °C. The mixture was stirred overnight at 40 °C.

b Combined isolated yield of **6**.

c ee was determined by chiral HPLC analysis of the isolated products. All reactions were performed with freshly distilled dry CH_2_Cl_2_ and HFIP stored over activated 4 Å molecular sieves.

Having demonstrated that the C–H functionalization of benzylic sites with the diazophosphonate **4** is a viable process, an optimization study with a series of the most established chiral catalysts was conducted (Table 1 and Figure 1). The bulky triarylcyclopropanecarboxylate catalyst, Rh_2_(*S-p*-Br-TPCP)_4_,^32^ gave only a trace of the desired product **6** (entry 2) even though it is an exceptional catalyst for C–H functionalization reactions with aryldiazoacetates. In contrast, the phthalimido-derived catalyst Rh_2_(*S*-PTAD)_4_, which had been shown to be effective in cyclopropanation reactions with **4**,^29^ gave the desired product **6** in 75% isolated yield with 83% ee (entry 3). The naphthalimido-derived catalyst Rh_2_(*R*-NTTL)_4_^33^ as well as the related phthalimido catalysts Rh_2_(*R*-TCPTAD)_4_^27^ and Rh_2_(*R*-TPPTTL)_4_^34^ all performed well, generating **6** in 92–93% ee and in good yields (entries 4–6). We recently prepared an extended series of Rh_2_(*R*-TPPTTL)_4_ derivatives,^25^ and so, these were also evaluated. The tetrabromo derivative, Rh_2_(*S-tetra*-(4-Br)TPPTTL)_4_, gave improved enantioselectivity (97% ee), but the yield was low (35%) (entry 7), whereas the dibromo derivative (Rh_2_(*S-di*-(4-Br)TPPTTL)_4_) was found to be exceptionally effective, generating **6** in 82% yield and 99% ee (entry 8). All of the extended Rh_2_(*R*-TPPTTL)_4_ derivatives performed well in benzylic C–H functionalization, but Rh_2_(*S-di*-(4-Br)TPPTTL)_4_ was the best (see Table S2 for details on the enantioselective C–H functionalization with the complete series of Rh_2_(*R*-TPPTTL)_4_ derivatives).

**Figure 1:**
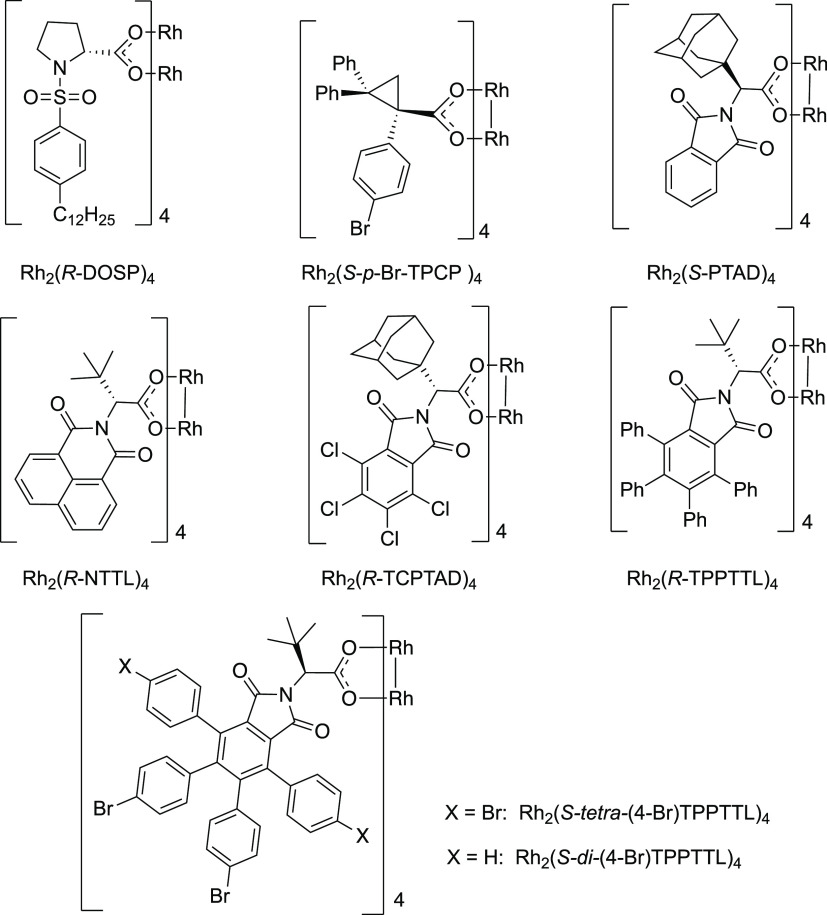
Chiral dirhodium catalysts.

The site-selectivity profile of diazophosphonate **4** is distinctively different from the corresponding reaction with the aryldiazoacetates (Scheme 2). Previous studies have shown that the Rh_2_(*S-tetra*-(4-Br)TPPTTL)_4_ catalyzed reaction of the aryldiazoacetate **7** with **5** strongly preferred the formation of the tertiary C−H functionalization product 8 over the primary C−H functionalization product **9** (>20:1 r.r.),^25^ the complete reverse from what was seen with phosphonate **4**. This is an indication that the size of the electron-withdrawing group can have a controlling influence on the C–H functionalization site-selectivity exhibited by donor/acceptor carbenes. The high asymmetric induction exhibited by these catalysts has been discussed in an earlier publication.^34^ The ligand self-assembles to generate C_4_-symmetric bowl-shaped structures, in which the 16 aryl rings on the periphery of the catalyst are tilted, leading to an induced helical chirality. This ligand motif has been shown to be capable of high asymmetric induction in a variety of carbene C–H functionalization reactions. Interestingly, the carbene face selectivity for the reactions with aryldiazophosphonates is opposite to what is seen with aryldiazoacetates.

**Scheme 2:**
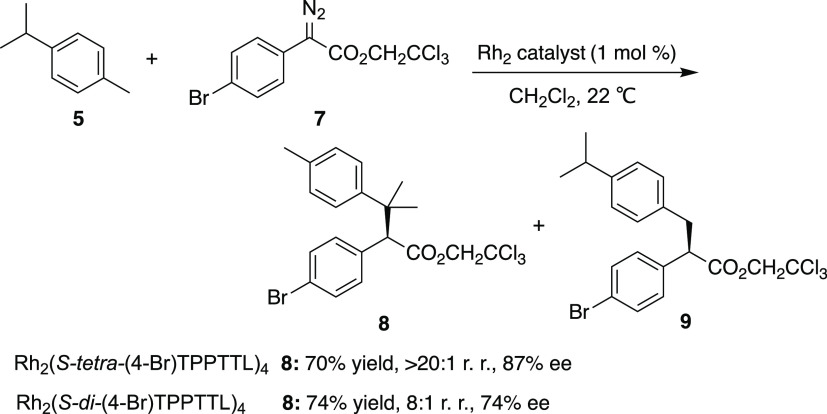
Aryldiazoacetate C–H Functionalization

Once Rh_2_(*S-di*-(4-Br)TPPTTL)_4_ was established as the optimum catalyst, the scope of the reaction was conducted with a series of representative substrates as illustrated in Table 2. The reactions with the substrates **10a**–**k** to form **11a**–**k** show a variety of functional groups that are compatible with this chemistry (Table 2). Notable examples are the boronates **11c** and **11e** and the alkynes **11d**, **11j**, and **11k**. Many of the substrates have potentially competing C–H bonds, but all of the reactions occur cleanly at the primary benzylic site. A particularly impressive example is **11f** with an extended bicyclohexyl side chain, which does not interfere with the reaction. Furthermore, all of the reactions are highly enantioselective (91–98% ee). The reactions could also be applied to late-stage C–H functionalization of primary benzylic sites of substrates derived from pharmaceutical agents (**10l**–**p**), generating **11l**–**p** in 84–97% ee (or d. e. for chiral substrates). Crystals of **11f** and **11p** were obtained to be suitable for determination of the absolute configurations by X-ray crystallography. The absolute configurations of the other C–H functionalization products are tentatively assigned by analogy.

**Table 2:**
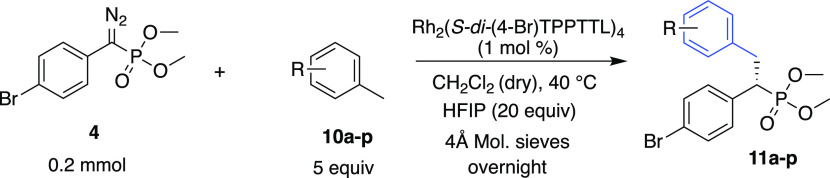

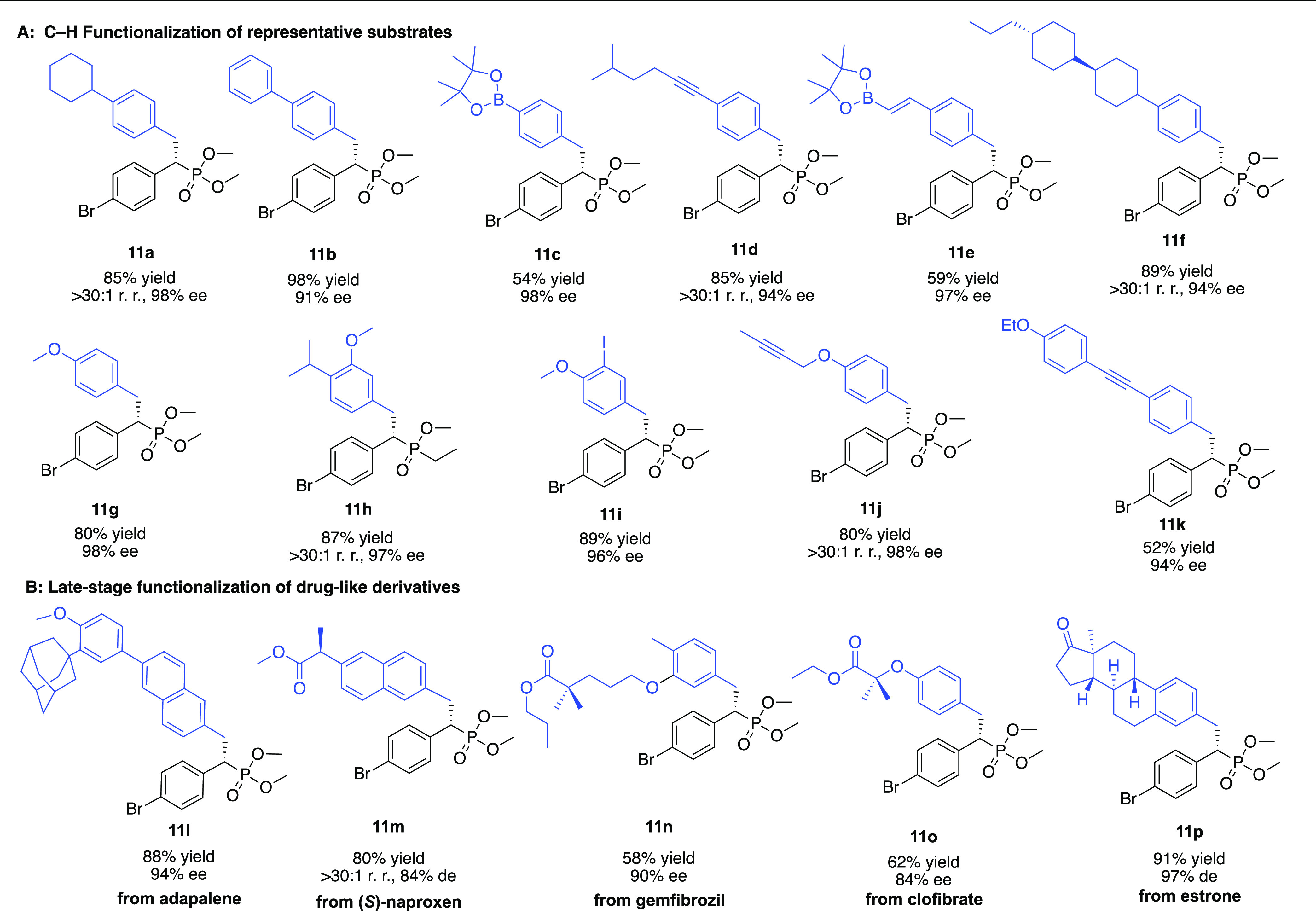
Scope of Primary Benzylic C–H Functionalization^a^

a Reaction conditions: To a mixture of **10a**–**p** (1.0 mmol) and [Rh] catalyst (1 mol %) in dry CH_2_Cl_2_ solvent (5.0 mL) was added a solution of **4** (0.2 mmol) in dry CH_2_Cl_2_ solvent (10.0 mL) via an automatic syringe pump over 5 h at 40 °C. The mixture was stirred overnight at 40 °C. The yield of **11a**−**p** is reported as isolated yield after purification. Enantioselectivity (ee) was determined by chiral HPLC analysis of the isolated products. All reactions were performed with freshly distilled dry CH_2_Cl_2_ and HFIP stored over activated 4 Å molecular sieves.

The reaction can be applied to a range of diazophosphonates **12a**–**e** as can be seen in the representative reaction with *p*-cymene **5** (Table 3). In all cases, the reaction occurs cleanly at the primary benzylic site to form **13a**–**e** (>30:1 r.r.). Furthermore, the enantioselectivity is high in all of the cases. Compounds **13a**, **13b**, and **13e** were each formed in 98% ee, while slightly lower levels of enantioselectivity were observed for the *meta*-methoxyphenyl analogue **13c** (97% ee) and the 2-naphthyl analogue **13e** (94% ee).

**Table 3:**
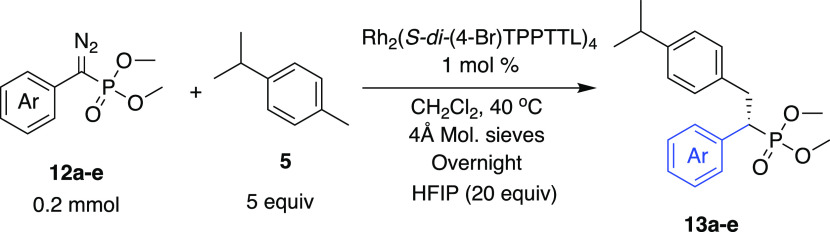

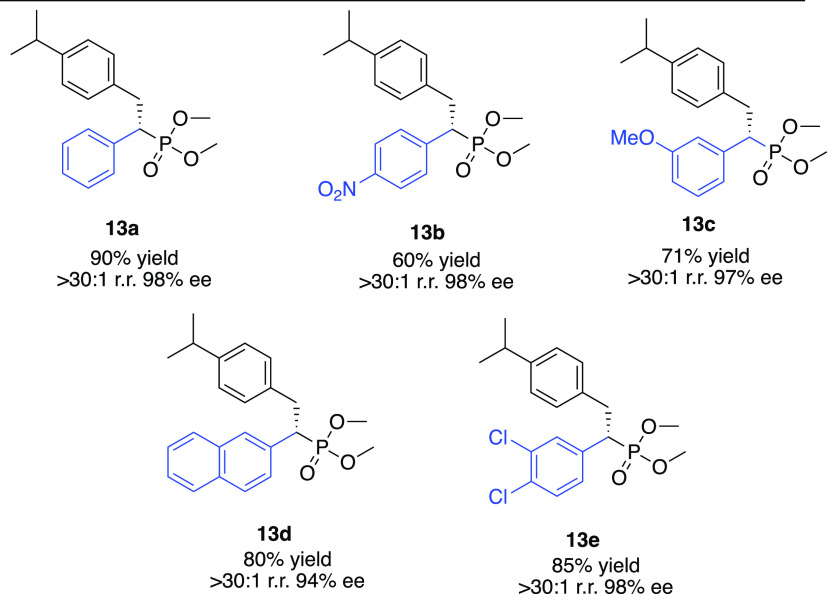
C–H Functionalization with Modified Aryldiazomethylphosphonate Derivatives

In the case of C–H functionalization studies with aryldiazoacetate, enhanced performance was often seen when the methyl ester was replaced with a trihaloethyl ester.^26−28^ Consequently, we decided to evaluate the trifluoroethyl phosphonate (**13**) to determine if it had a beneficial effect on C–H functionalization. As a test reaction, the C–H functionalization of **5** was examined (Scheme 3). Once again, a very clean reaction was observed, generating **15** in 84% yield, >30:1 d.r. and with very high asymmetric induction (98% ee). The absolute configuration of **15** was confirmed by X-ray crystallography and was the same as that observed for the methyl phosphonate derivatives.

**Scheme 3:**
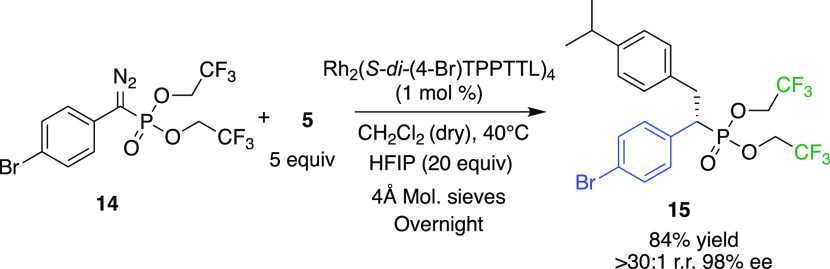
Impact of Bis(trifluoroethyl) Phosphonate

In summary, these studies are a further demonstration of the unique properties of rhodium-stabilized donor/acceptor carbenes for site and stereoselective C–H functionalization. ((Aryl)(diazo)methyl)phosphonates are effective carbene precursors for C–H functionalization, broadening the range of functionality that can be introduced in carbene-induced enantioselective C–H functionalization. They require slightly more forcing conditions than the aryldiazoacetates, and the use of molecular sieves and HFIP is a requirement for reasonable yields of product. Under the optimized conditions, highly efficient C–H functionalization at primary benzylic sites was achieved. Even though dimethyl phosphonates are highly effective substrates for C–H functionalization, the trifluoroethyl derivatives can also be used if needed. A particularly significant feature of this study is the demonstration that the tetrahedral phosphonate group behaves more sterically demanding than the trigonal ester group, which enhances the preference for primary C–H functionalization over more sterically congested sites. The illustration that the nature of the acceptor group can have a dramatic influence on site-selectivity offers interesting new opportunities for further refinement of the carbene-induced C–H functionalization chemistry.

## Data Availability

The data underlying this study are available in the published article and its online Supporting Information.

## Supporting Information Available

Complete experimental procedures, and compound characterization (PDF)

## Accession Codes

The following crystal structures have been deposited in the Cambridge Crystallographic Data Centre: **11f** (CCDC 2194599), **11p** (CCDC 2194598), and **15** (CCDC 2254293). These data can be obtained free of charge via www.ccdc.cam.ac.uk/data_request/cif, or by emailing data_request@ccdc.cam.ac.uk, or by contacting The Cambridge Crystallographic Data Centre, 12 Union Road, Cambridge CB2 1EZ, UK; fax: + 44 902 1223 336033.
