# Supplementary material for: Enantioselective Intermolecular C–H Functionalization of Primary Benzylic C–H Bonds Using ((Aryl)(diazo)methyl)phosphonates
Source: ACS Catal. 2023 Dec 11;14(1):124–30. doi: 10.1021/acscatal.3c04661 (PMC10775147; doi:10.1021/acscatal.3c04661)
Supplement: Supplementary file 1 — cs3c04661_si_001.pdf [file cs3c04661_si_001.pdf]

Supporting Information

Enantioselective Intermolecular C–H Functionalization of Primary Benzylic C–H Bonds Using ((Aryl)(diazo)methyl)phosphonates.

Yasir Naeem, Bianca T. Matsuo, and Huw M. L. Davies*

Department of Chemistry, Emory University, 1515 Dickey Drive, Atlanta, Georgia 30322, United States.

hmdavie@emory.edu

Table of Contents

1. Catalysts, Diazo, and Substrate Structures Utilized in This Study……………………….…..S1

2. Crude NMRs with Catalysts Screening for Diazo bis(2,2,2-trifluoroethyl) ((4-bromophenyl)(diazo)methyl)phosphonate **(15)**…………………………………………………...………......S3

3. New TPPTTL derivatized Catalysts Screening with *p*-Cresol………………………………..S4

4. General Information S5

5. Preparation and Characterization of Substrates...S7

6. Rhodium Catalyzed C–H Functionalization of Substrates and Characterization of Products...S23

7. Reference……………………..S48

8. ^{1}H NMR and ^{13}C NMR Spectroscopic Data...S51

9. Enantioselectivity Determination by HPLC or SFC...S156

10. X-Ray Crystallographic Data for Compound **11f, 11p,** and **15**..S176

11. Bibliography…………………………………………………………………………....S221

1. Catalysts, Diazo, and Substrate Structures Utilized in This Study.

Chart 1. Catalysts Utilized in this Study.

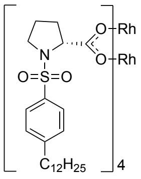

Rh_2(*R*-DOSP)$_4$

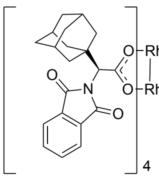

Rh_2(*S*-PTAD)$_4$

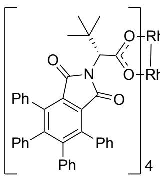

Rh_2(*R*-TPPTTL)$_4$

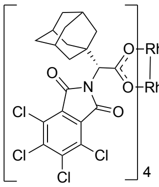

Rh_2(*R*-TCPTAD)$_4$

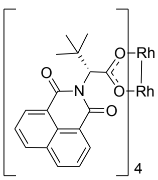

Rh_2(*R*-NTTL)$_4$

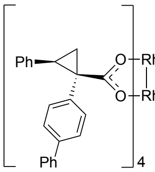

Rh_2(*S*-DPCP)$_4$

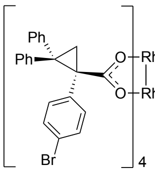

Rh_2(*S*-*p*-Br-TPCP)$_4$

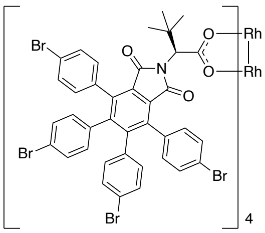

Rh_2(*S*-*tetra*-(4-Br)TPPTTL)$_4$

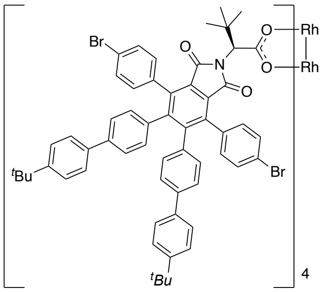

Rh_2(*S*-*di*-(4-Br)-di(4-^{t}BuPh)TPPTTL)$_4$

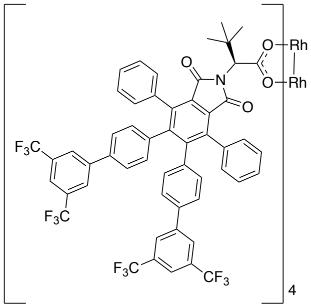

Rh_2(*S*-*di*-(3,5-diCF$_3$Ph)TPPTTL)$_4$

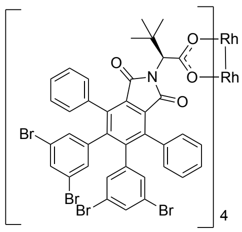

Rh_2(*S*-*di*-(3,5-diBr)TPPTTL)$_4$

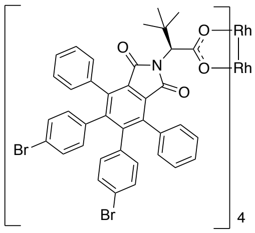

Rh_2(*S*-*di*-(4-Br)TPPTTL)$_4$

Chart 2. Substrate Structures Utilized in this Study.

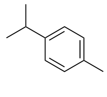

5

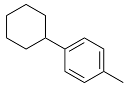

10a

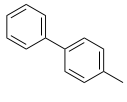

10b

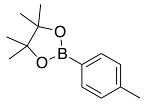

10c

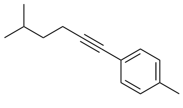

10d

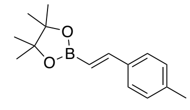

10e

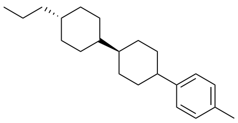 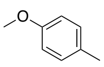 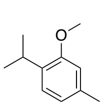 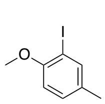 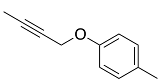 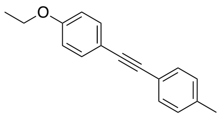

10f **10g** **10h** **10i** **10j** **10k**

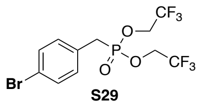

S29

Pharmaceutically Relevant Substrates

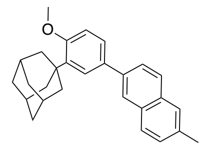 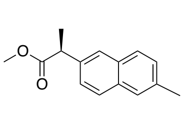 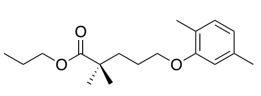 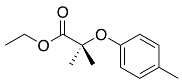 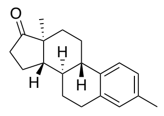

10l from adapalene

10m from (*S*)-naproxen

10n from gemfibrozil

10o from clofibrate

10p from estrone

Chart 3. Diazo Compounds Utilized in this Study.

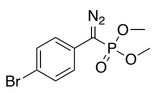 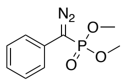 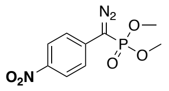 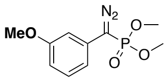 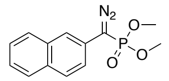 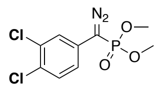 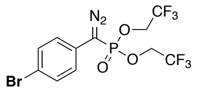

4 **12a** **12b** **12c** **12d** **12e** **14**

2. *Table S1. Crude NMRs with Catalysts Screening for New Diazo bis(2,2,2-trifluoroethyl) ((4-bromophenyl)(diazo)methyl)phosphonate (15).*[a]

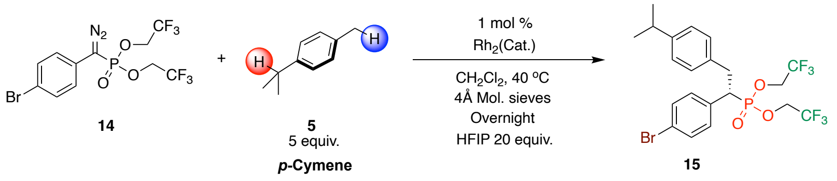

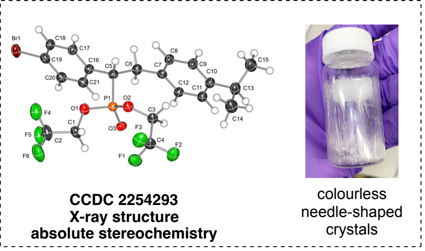

Entry	Catalyst	Yield, %[b]	r. r.	ee %[c]
1	Rh_2(*S*-PTAD)$_4$	59	>30:1	65
2	Rh_2(*R*-TPPTTL)$_4$	82	>30:1	–83
3	Rh_2(*S*-DOSP)$_4$	75	>30:1	67
4	Rh_2(*R*-BPCP)$_4$	24	>30:1	–20
5	Rh_2(*R*-TCPTAD)$_4$	33	>30:1	–60
6	Rh_2(*R*-DPCP)$_4$	…	…	…
7	Rh_2(*S*-*p*-Br-TPCP)$_4$	…	…	…
8	**Rh_2(*S*-*di*-(4-Br) TPPTTL)$_4$**	**84**	>30:1	**98**

[a]Reaction conditions: To a mixture of **5** (1.0 mmol) and [Rh] catalyst (1 mol %) in dry CH_2Cl_2 solvent (5.0 mL) was added a solution of **14** (0.2 mmol) in dry CH_2Cl_2 solvent (10.0 mL) via an automatic syringe pump over 5 h at 40 °C. The mixture was stirred overnight at 40 °C. [b]Combined isolated yield of **15.** [c]ee determined by chiral HPLC analysis of the isolated products. All reactions were performed with freshly distilled dry CH_2Cl_2 and HFIP stored over activated 4Å molecular sieves

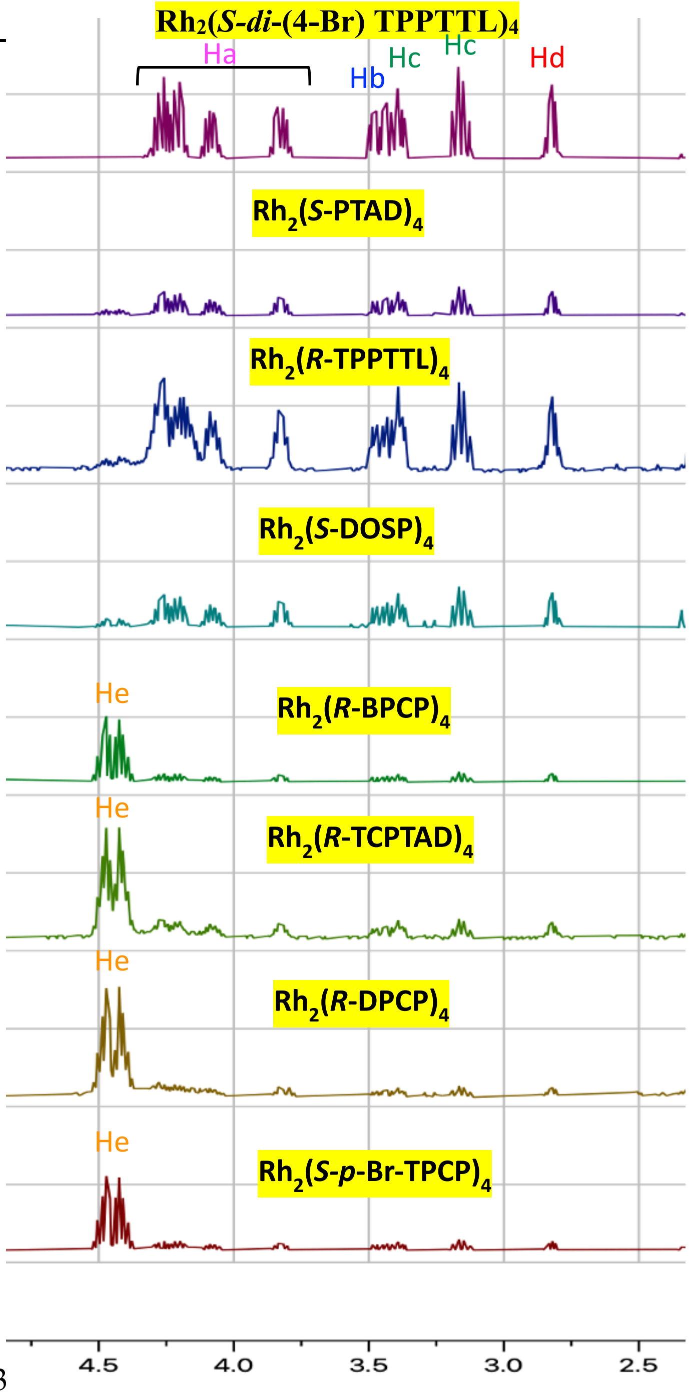

<u>NMR peaks</u>

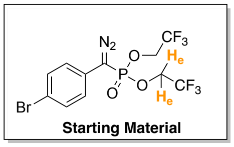

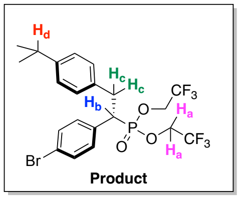

The crude NMR showed that we should focus on the TPPTTL type/derivatized catalysts for a respectful yield and enantioselectivity. We had TPPTTL derivatized catalysts already in our toolbox, and the catalyst that gave us the best yield and enantioselectivity is Rh_2(*S*-*di*-(4-Br) TPPTTL)$_4$. The crude NMR showed that the catalysts that had a majority of the product formed are Rh_2(*S*-PTAD)$_4$, Rh_2(*R*-TPPTTL)$_4$, and Rh_2(*S*-DOSP)$_4$. The product is shown in the crude NMR from 4.34 – 2.82 ppm, and the starting material is shown at 4.53–4.36 ppm.

3. *Table S2. New TPPTTL derivatized Catalysts Screening with p-Cresol.*[a]

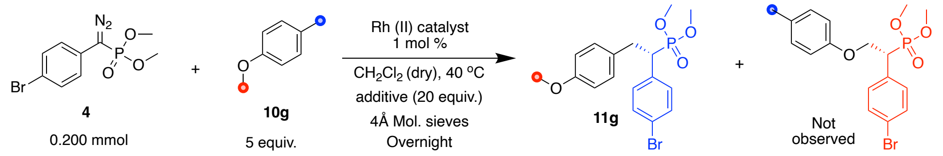

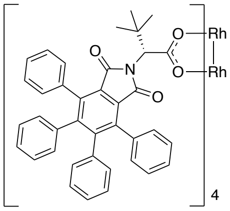

Rh_2(*R*-TPPTTL)$_4$

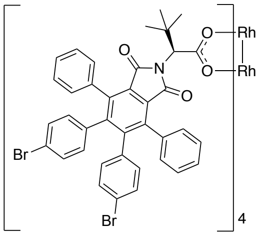

Rh_2(*S-di*-(4-Br)TPPTTL)$_4$

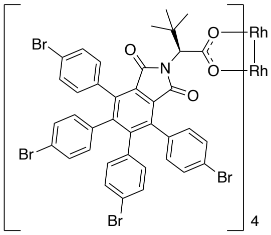

Rh_2(*S-tetra*-(4-Br)TPPTTL)$_4$

A

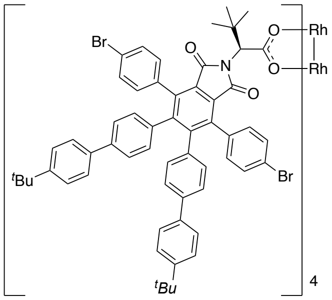

Rh_2(*S-di*-(4-Br)-di(4-^{t}BuPh)TPPTTL)$_4$

B

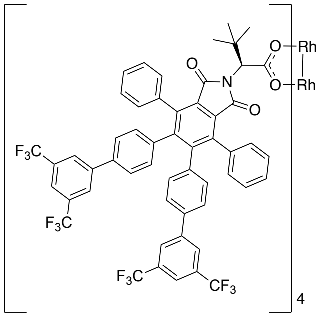

Rh_2(*S-di*-(3,5-diCF_3Ph)TPPTTL)$_4$

C

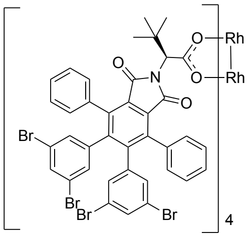

Rh_2(*S-di*-(3,5-diBr)TPPTTL)$_4$

D

Entry	Catalyst	Yield, %[b]	r. r.	ee, %[c]
1	Rh_2(*R*-TPPTTL)$_4$	83	>30:1	–90
2	A	35	>30:1	97
3	B	43	>30:1	73
4	C	50	>30:1	94
5	D	80	>30:1	94
6	**Rh_2(*S-di*-(4-Br) TPPTTL)$_4$**	**80**	>30:1	**97**

[a]Reaction conditions: To a mixture of **10g** (1.0 mmol) and [Rh] catalyst (1 mol %) in dry CH_2Cl_2 solvent (5.0 mL) was added a solution of **4** (0.2 mmol) in dry CH_2Cl_2 solvent (10.0 mL) via an automatic syringe pump over 5 h at 40 °C. The mixture was stirred overnight at 40 °C. [b]Combined isolated yield of **11g.** [c]ee determined by chiral HPLC analysis of the isolated products (a negative value indicates the asymmetric induction favors the opposite enantiomer to the one drawn). All reactions were performed with freshly distilled dry CH_2Cl_2 and HFIP stored over activated 4Å molecular sieves.

2. *General Information.*

All chemicals were used as purchased or purified according to Purification of Common Laboratory Chemicals if necessary. All chemical reagents were purchased from commercial sources and use as received unless otherwise noted. ^{1}H, ^{13}C, ^{19}F, and ^{31}P NMR spectra were recorded at either 400, 500 or 600 MHz on Bruker spectrometer or Varian spectrometer, (^{13}C at 101, or 151 MHz), 400 or 600 MHz on Bruker spectrometer, (^{19}F at 376 or 565) on Bruker-400 or Bruker-600 spectrometers, and (^{31}P at 162 or 243 MHz) on Bruker-400 or Bruker-600 spectrometers and all were reported in parts per million (ppm). All NMR samples solvent were performed using deuterated chloroform ($CDCl_3$) solvent unless otherwise noted. Coupling constant (*J*) values are recorded in Hertz (Hz). Abbreviations for ^{1}H NMR signals coupling are as follows: s = singlet, d = doublet, t = triplet, m = multiplet, dd = doublet of a doublet, ddd = doublet of a doublet of a doublet, dtd doublet of a triplet of a doublet. Mass spectral determinations were carried out by FTMS+p-NSI or APCI as ionization source unless otherwise noted. Reactions were carried out under argon in flame-dried or oven-dried glassware before use unless otherwise specified. Dichloromethane used for C–H functionalization reactions were dried using calcium hydride (CaH_2) and stored under argon overnight before use in 4Å molecular sieves and degassed for one hour with argon. Thin-layer chromatography (TLC) analysis was performed with aluminum-sheet silica gel plates, visualizing with UV light, and staining with potassium permanganate (aqueous $KMnO_4$), ceric ammonium molybdate (CAM), or phosphomolybdic acid (PMA). Flash column chromatography was carried out on Merck silica gel 60 Å (230-400 mesh). Solvents THF, Et_2O, CH_3CN, CH_2Cl_2, trifluorotoluene, and toluene were dried by solvent purifier. The dirhodium catalysts were prepared using the published procedures: Rh_2(*R*-DOSP)$_4$,[1] Rh_2(*S*-PTAD)$_4$,[2,3] Rh_2(*R*-TPPTTL)$_4$,[4] Rh_2(*R*-TCPTAD)$_4$,[5] Rh_2(*R*-

NTTL)$_4$,[6] Rh_2(*S*-DPCP)$_4$, Rh_2(*S*-*p*-Br-TPCP)$_4$,[7] Rh_2(*S*-tetra-(4-Br)TPPTTL)$_4$, Rh_2(*S*-*di*-(4-Br)-di(4-^{t}BuPh)TPPTTL)$_4$, Rh_2(*S*-*di*-(3,5-diCF3Ph)TPPTTL)$_4$, Rh_2(*S*-*di*-(3,5-diBr)TPPTTL)$_4$, and Rh_2(*S*-*di*-(4-Br)TPPTTL)$_4$. [8]

Infrared (IR) spectra were collected on a Nicolet Impact Series 10 FT-IR spectrometer.

Mass spectrometric determinations were carried out on a Thermo Finnigan Exactive Plus Mass spectrometer with electrospray ionization (ESI) or atmospheric pressure chemical ionization (APCI) Quadrupole-Orbitrap Mass spectrometer with ESI and APCI.

Melting points (m.p.) were measured in open capillary tubes with a Mel-Temp Electrothermal melting points apparatus and are uncorrected.

Enantiomeric excess (ee) data were obtained on Agilent 1100, or Agilent 1290 Infinity II instruments, eluting the purified products using a mixed solution of HPLC-grade 2-propanol (*i*PrOH) and n-hexane. Supercritical Fluid Chromatography (SFC) was performed on a Waters Acquity UPC2 system using methanol/isopropanol with 0.2% formic acid in supercritical carbon dioxide as eluent.

2. Preparation and Characterization of Substrates.

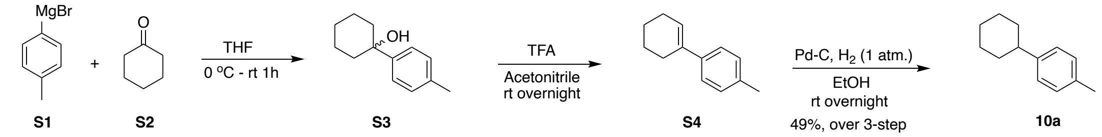

1-cyclohexyl-4-methylbenzene (10a)

To a stirred solution of *p*-tolylmagnesium bromide **S1** (3.5 g, 1 equiv.) in THF (42 mL) at 0 °C under argon, cyclohexanone **S2** (1.8 g, 0.9 equiv.) in THF (8 mL) was added dropwise. The reaction mixture was stirred for 1 hour at the room temperature, quenched with sat. NH_4Cl, and extracted with ethyl acetate. The organic layer was washed with brine, dried over Na_2SO_4, and the residue was evaporated under reduced pressure to remove the volatile materials. The crude product was purified by flash column chromatography silica gel (hexanes/ethyl acetate 20:1) to afford 1-(*p*-tolyl)cyclohexan-1-ol **S3** (2.0 g, 60%) as colorless oil.

To a stirred solution of 1-(*p*-tolyl)cyclohexan-1-ol **S3** (1.0 g, 5.0 mmol) was dissolved in acetonitrile (10 mL), then TFA (0.4 mL, 1.0 equiv.) was added at room temperature. The reaction mixture was stirred for overnight, diluted with ethyl acetate, washed with sat. $NaHCO_3$, dried over Na_2SO_4, and evaporated to afford 1-(4-Methylphenyl)cyclohexene **S4** (0.85 g) as colorless oil.

To a stirred solution 1-(4-Methylphenyl)cyclohexene **S4** (0.85 g, 4.9 mmol) in ethanol (25 mL), was added palladium on activated carbon (Pd/C 5%, 0.26 g). The reaction solution was purged with hydrogen balloon for 15 minutes and then went overnight under hydrogen balloon. Then, the reaction was filtered over a short path of Celite, concentrated in vacuum, and the crude mixture was purified by flash column chromatography (SiO_2; hexanes/EtOAc, 25:1 gradient; R_f

= 0.87 in 9:1 hexanes/EtOAc) to afford the final product **10a** as colorless oil (0.77 g, 49%, 3-step). Spectroscopic data are in agreement with those reported in the literature. [9-11]

^{1}H NMR (600 MHz, $CDCl_3$) δ 7.12 (s, 4H), 2.48 (ddt, *J* = 11.5, 8.8, 3.5 Hz, 1H), 2.33 (s, 3H), 1.90 – 1.82 (m, 4H), 1.76 (ddq, *J* = 1.6, 3.3, 12.8 Hz, 1H), 1.47 – 1.35 (m, 4H), 1.26 (tdd, *J* = 13.5, 8.1, 4.0 Hz, 1H). (Coupling constants are uncorrected)

^{13}C NMR (151 MHz, $CDCl_3$): δ 145.2, 135.2, 129.0, 126.8, 44.3, 34.9, 27.1, 26.3, 21.0.

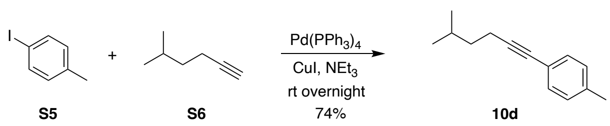

1-methyl-4-(5-methylhex-1-yn-1-yl)benzene (10d)

To a stirred solution of 4-iodotoluene **S5** (14 mmol, 3.0 g), 5- methyl-1-hexyne **S6** (21 mmol, 2.0 g, 1.5 equiv.), $Pd(PPh_3)_4$ (2.5 mol%, 400 mg), CuI (5 mol%. 130 mg) under argon atmosphere, triethylamine (40 mL) was added, and the reaction mixture was stirring at room temperature overnight. Then, the reaction was quenched with saturated aqueous ammonium chloride and the crude mixture was extracted with ether, washed by brine, dried over anhydrous $MgSO_4$, concentrated in vacuum. The crude mixture was purified by flash column chromatography (SiO_2; hexanes/EtOAc, 50:1 gradient; R_f = 0.85 in 9:1 hexanes/EtOAc) to afford the product **10d** as colorless oil (1.90 g, 74% yield). Spectroscopic data are in agreement with those reported in the literature. [12]

^{1}H NMR (600 MHz, $CDCl_3$) δ 7.27 (d, *J* = 7.8 Hz, 2H), 7.08 (d, *J* = 7.8 Hz, 2H), 2.40 (t, *J* = 7.4 Hz, 2H), 2.33 (s, 3H), 1.76 (dp, *J* = 13.4, 6.7 Hz, 1H), 1.50 (q, *J* = 7.4 Hz, 2H), 0.94 (dd, *J* = 6.6, 0.8 Hz, 6H). (Coupling constants are uncorrected)

^{13}C NMR (151 MHz, $CDCl_3$) δ 137.4, 131.4, 128.9, 121.0, 89.7, 80.4, 37.8, 27.3, 22.2, 21.4, 17.4.

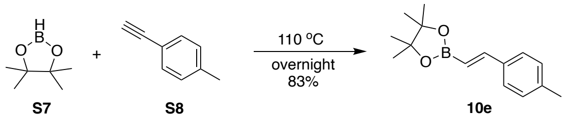

S7 S8 10e

(*E*)-4,4,5,5-tetramethyl-2-(4-methylstyryl)-1,3,2-dioxaborolane (10e)

To a stirred solution of phenylacetylene **S8** (17.2 mmol, 1 equiv.), and **S7** pinacolborane (34.4 mmol, 2 equiv.) under an argon atmosphere at room temperature, and the reaction mixture was heated to 110 °C and stirred for overnight. Then, the reaction was cooled to room temperature and was quenched with water. The crude mixture was extracted with ethyl acetate (3×25 mL) and the organic layer was dried over anhydrous Na_2SO_4 and was concentrated under vacuum. The crude mixture was purified by flash column chromatography (SiO_2; hexanes/DCM, 7:3 gradient; R_f = 0.79 in 9:1 hexanes/DCM) to afford the product **10e** as light-yellow oil (3.5 g, 83% yield). Spectroscopic data are in agreement with those reported in the literature. [13]

^{1}H NMR (600 MHz, $CDCl_3$) δ 7.39 (d, *J* = 7.8 Hz, 3H), 7.36 (s, 1H), 7.14 (d, *J* = 7.8 Hz, 2H), 6.11 (d, *J* = 18.4 Hz, 1H), 2.34 (s, 3H), 1.31 (s, 12H). (Coupling constants are uncorrected)

^{13}C NMR (151 MHz, $CDCl_3$) δ 149.5, 139.0, 134.8, 129.3, 127.0, 83.3, 24.8, 21.3.

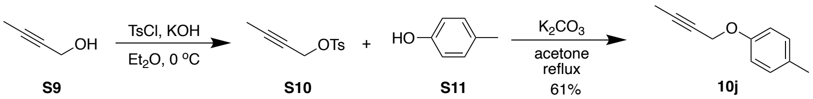

S9 S10 S11 10j

1-(but-2-yn-1-yloxy)-4-methylbenzene (10j)

To a solution of 2-butyn-1-ol **S9** (3.0 g, 42.8 mmol) in diethyl ether (100 mL) at 0 °C was added *p*-toluenesulfonyl chloride (8.98 g, 47.1 mmol) and crushed KOH (12 g) sequentially. The resulting mixture was stirred at 0 °C for 1 h before it was poured into an ice-water mixture. Ethyl

ether (50 mL) was added, and the organic phase was separated, dried over $MgSO_4$, and concentrated to give **S10** as light-yellow oil.

To a solution of *p*-cresol **S11** (3.00 g, 27.7 mmol) and **S10** (2.66 g, 41.6 mmol) in acetone (40 mL) was added K_2CO_3 (4.60 g, 33.3 mmol). The mixture was stirred at reflux for 18 h and then cooled to rt. The solvent was removed under vacuum and hexane was added to the residue. The organic phase was washed with water and brine, dried over anhydrous Na_2SO_4, concentrated, and purified by flash chromatography (SiO_2; hexanes/EtOAc, 20:1 gradient; R_f = 0.90 in 9:1 hexanes/EtOAc) to afford the final product **10j** as colorless oil (2.70 g, 61% yield). Spectroscopic data are in agreement with those reported in the literature. [14]

^{1}H NMR (600 MHz, $CDCl_3$) δ 7.10 (d, *J* = 8.7 Hz, 2H), 6.88 (d, *J* = 8.7 Hz, 2H), 4.63 (q, *J* = 2.1 Hz, 2H), 2.31 (s, 3H), 1.87 (t, *J* = 2.4 Hz, 3H). (Coupling constants are uncorrected)

^{13}C NMR (101 MHz, $CDCl_3$) δ 156.0, 130.7, 130.1, 114.9, 83.8, 74.5, 56.7, 20.8, 3.9.

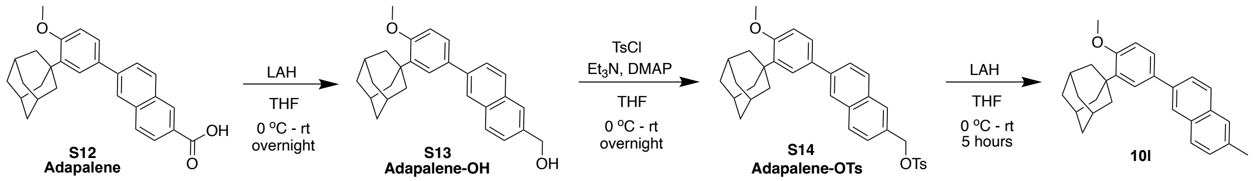

1-(2-methoxy-5-(6-methylnaphthalen-2-yl)phenyl)adamantane (10l).

To a stirred solution of adapalene **S12** (1.1 g, 2.67 mmol) and THF (25 mL) were added under argon atmosphere. $LiAlH_4$ (202 mg, 5.33 mmol) was added slowly at 0 °C, and then the mixture was stirred at room temperature overnight. The reaction was quenched by water (4 mL) and then extracted with dichloromethane, dried over Na_2SO_4, and evaporated to give the naphthyl alcohol product **S13**. The naphthyl alcohol **S13** compound was used directly for the next reaction.

Adapalene alcohol **S13** was dissolved in dry THF (25 mL) and Et_3N (0.23 mL, 1.68 mmol) was added at 0 °C under argon atmosphere. Then TsCl (213 mg, 1.12 mmol) followed by DMAP (6 mg, 0.05 mmol) were added to the resulting reaction mixture and stirring was continued overnight at room temperature. The reaction mixture was quenched with aqueous saturated NH_4Cl solution. Afterwards it was extracted with EtOAc, washed with water, brine and dried over anhydrous $MgSO_4$. The organic extracts were filtered and concentrated under reduced pressure to give **S14**. The tosylated adapalene compound **S14** was used directly for the next reaction.

Compound **S14** was dissolved in anhydrous THF (20 mL) and $LiAlH_4$ (85 mg, 2.24 mmol) was added at 0°C under argon atmosphere. The reaction mixture was warmed to room temperature and stirring was continued for next 5 h. The reaction was quenched with aqueous saturated NH_4Cl solution at 0 °C and the white precipitate was filtered. The filtrate was taken up in EtOAc, washed with water, brine and dried over anhydrous $MgSO_4$. The crude mixture was filtered, and the solvent was removed under reduced pressure to remove the volatile materials. The crude product was purified by flash column chromatography silica gel (SiO_2; hexanes/EtOAc, 20:1 gradient; R_f = 0.90 in 9:1 hexanes/EtOAc) to afford the product **10l** as white solid (0.82 g, 51% yield). Spectroscopic data are in agreement with those reported in the literature. [9, 15]

^{1}H NMR (600 MHz, $CDCl_3$) δ 7.95 (d, J = 1.9 Hz, 1H), 7.64 (dd, J = 8.6, 16.8 Hz, 2H), 7.58 (s, 1H), 7.50 (dd, J = 2.0, 8.7 Hz, 1H), 7.34 (dd, J = 1.7, 8.5 Hz, 1H), 7.29 (d, J = 2.6 Hz, 1H), 7.26 (dd, J = 2.4, 8.6 Hz, 1H), 6.73 (d, J = 8.6 Hz, 1H), 3.81 (s, 3H), 2.50 (s, 3H), 2.06 (s, 9H), 1.76 (d, J = 2.5 Hz, 6H). (Coupling constants are uncorrected)

^{13}C NMR (101 MHz, CDCl$_3$) δ 158.8, 139.2, 138.4, 135.5, 133.7, 132.8, 132.4, 128.8, 128.2, 127.9, 126.2, 126.1, 125.8, 125.1, 112.4, 55.5, 41.0, 38.1, 37.5, 29.5, 22.1.

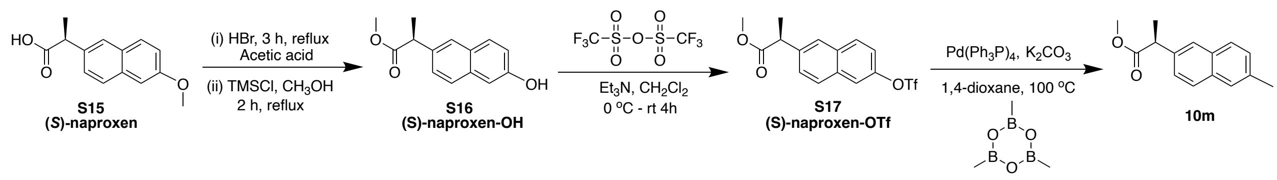

Methyl (*S*)-2-(6-methylnaphthalen-2-yl)propanoate (10m).

To (*S*)-naproxen **S15** (3 g, 13 mmol) in acetic acid (20 mL) was added 48% HBr (11.0 g, 7.37 mL, 65.1 mmol) at 0 °C. The mixture was stirred at reflux for 3 h and then cooled to room temperature, water (50 mL) was added to precipitate out the product, which was isolated by filtration. To the crude product was added methanol (35 mL) and TMSCl (1.7 g, 2.0 mL, 15.6 mmol), and the mixture was stirred at room temperature for 2 h. The solvent was removed in vacuo, resulting in a tan solid compound **S16**, which was dissolved in CH_2Cl_2 (25 mL). Following the addition of triethylamine (2.6 g, 3.6 mL, 26.1 mmol) at 0 °C, trifluoromethanesulfonic anhydride (4.9 g, 2.9 mL, 15.7 mmol) was added dropwise and the mixture was warmed to room temperature and allowed to stir for 4 h. The mixture was then diluted with diethyl ether, quenched with 1 M HCl, and washed with saturated sodium bicarbonate and brine. The organic layer was dried over $MgSO_4$ and then concentrated, resulting in the (*S*)-naproxen-OTf product **S17** (4.5 g, 72%). [16]

A clean oven-dried and flame-dried 50 mL round bottom flask equipped with activated 4 Å molecular sieves, and a magnetic stir-bar was evacuated and purged with argon (2-3 times). After cooling down to room temperature and charged with (*S*)-naproxen-OTf compound **S17** (2.1 g, 5.8 mmol), $Pd(PPh_3)_4$ (5.0 mol%. 335 mg, 290 μmol) and K_2CO_3 (2.4 g, 17.4 mmol, 3.0 equiv.).

Freshly distilled and degassed 1,4-dioxane (25 ml) was added, and the resulting solution was stirred for 5 min. Then trimethylboroxine (1.0 g, 17.4 mmol, 3.0 equiv.) was introduced, and the mixture was stirred at 100 °C for overnight and then cooled to room temperature. Then, the reaction was filtered over a pad of Celite, concentrated in vacuum, and the crude mixture was purified by flash column chromatography. (SiO_2; hexanes:EtOAc, 10:1 to 1:1 gradient; R_f = 0.75 in 9:2 hexanes:EtOAc), which afforded **10m** as a white solid (1.03 g, 65% yield).

m.p. 44-46 °C.

^{1}H NMR (600 MHz, $CDCl_3$) δ 7.75–7.69 (m, 3H), 7.59 (s, 1H), 7.41 (d, *J* = 8.5 Hz, 1H), 7.32 (d, *J* = 8.5 Hz, 1H), 3.89 (q, *J* = 7.1 Hz, 1H), 3.68 (s, 3H), 2.51 (s, 3H), 1.60 (d, *J* = 7.2 Hz, 3H). (Coupling constants are uncorrected)

^{13}C NMR (151 MHz, $CDCl_3$) δ 175.4, 137.4, 135.8, 133.1, 132.0, 128.8, 128.0, 127.9, 126.9, 126.2, 126.0, 52.4, 45.8, 22.0, 18.9.

IR (neat) 2978, 1735, 1607, 1504, 1434, 1376, 1329, 1251, 1195, 1167, 1093, 1066, 912, 882, 817, 657, 477, 462, 435, 424, 416, 404 cm^{-1}.

HRMS (APCl) calcd. for $C_{15}H_{16}O_2$ $[M - H]^-$ 227.1078, found 227.1080.

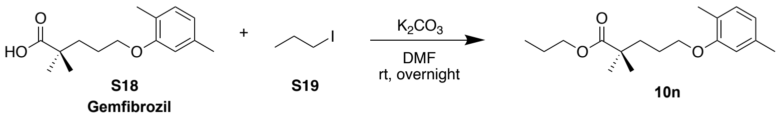

Propyl 5-(2,5-dimethylphenoxy)-2,2-dimethylpentanoate (10n).

To a solution of gemfibrozil **S18** (2.0 g, 7.99 mmol) and K_2CO_3 (1.3 g, 16.00 mmol) in 25 mL DMF was added 1-iodopropane **S19** (2.72 g, 10.4 mmol) at room temperature and the reaction was stirred overnight. Then the reaction was diluted with 25 mL EtOAc and washed with sat. brine (3×25 mL). The organic layer was dried over anhydrous Na_2SO_4 and was

concentrated under vacuum. The crude mixture was purified by flash column chromatography (SiO_2; hexanes/EtOAc, 20:1 gradient; R_f = 0.77 in 9:1 hexanes/EtOAc) to afford the product **10n** as colorless oil (1.90 g, 81% yield). Spectroscopic data are in agreement with those reported in the literature. [17]

^{1}H NMR (400 MHz, $CDCl_3$) δ 7.00 (d, J = 7.4 Hz, 1H), 6.66 (dd, J = 1.6, 7.5 Hz, 1H), 6.61 (d, J = 1.6 Hz, 1H), 4.03 (t, J = 6.6 Hz, 2H), 3.92 (t, J = 5.6 Hz, 2H), 2.31 (s, 3H), 2.18 (s, 3H), 1.84 – 1.61 (m, 6H), 1.22 (s, 6H), 0.95 (t, J = 7.4 Hz, 3H). (Coupling constants are uncorrected)

^{13}C NMR (101 MHz, $CDCl_3$) δ 178.2, 157.3, 136.8, 130.6, 123.9, 121.0, 112.3, 68.3, 66.3, 42.4, 37.5, 25.5, 22.4, 21.7, 16.1, 10.8.

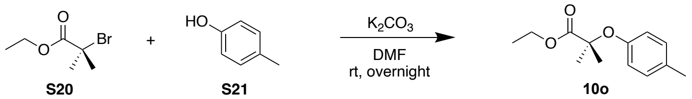

S20 S21 *p*-Cresol 10o

Ethyl 2-methyl-2-(*p*-tolyloxy)propanoate (10o).

To a solution of *p*-Cresol **S21** (2.0 g, 18.5 mmol) and K_2CO_3 (5.11 g, 37.0 mmol) in 20 mL DMF was added ethyl 2-bromoisobutyrate **S20** (2.41 g, 12.3 mmol) at room temperature and the reaction was stirred overnight. Then the reaction was diluted with 25 mL EtOAc and washed with sat. brine (3×25 mL). The organic layer was dried over anhydrous Na_2SO_4 and was concentrated under vacuum. The crude mixture was purified by flash column chromatography (SiO_2; hexanes/EtOAc, 50:1 gradient; R_f = 0.78 in 9:1 hexanes/EtOAc) to afford the product **10o** as colorless oil (2.2 g, 35% yield). Spectroscopic data are in agreement with those reported in the literature. [18]

^{1}H NMR (600 MHz, $CDCl_3$) δ 7.03 (d, J = 8.8 Hz, 2H), 6.76 (d, J = 8.5 Hz, 2H), 4.24 (d, J = 7.2 Hz, 2H), 2.27 (s, 3H), 1.26 (d, J = 7.2 Hz, 3H). (Coupling constants are uncorrected)

^{13}C NMR (101 MHz, CDCl$_3$) δ 174.8, 153.4, 132.0, 129.9, 119.8, 79.4, 61.7, 25.7, 20.9, 14.4.

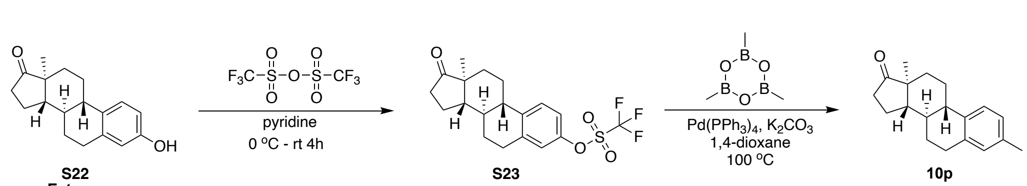

(8*R*,9*S*,13*S*)-3,13-dimethyl-6,7,8,9,11,12,13,14,15,16-decahydro-17*H*-cyclopenta[*a*]phenanthren-17-one (10p).

To a stirred solution of Estrone **S22** (2.5 g, 9.3 mmol) in pyridine (25 mL) at 0 °C under argon, trifluoromethanesulfonic anhydride (1.55 mL, 9.3 mmol) was dropwise added causing the solution to turn orange. The reaction mixture was stirred at room temperature for 4 hours. Then, the reaction was diluted with DCM (20 mL) and washed with water and brine, dried over anhydrous Na_2SO_4. The residue was evaporated under reduced pressure to remove the volatile materials. The crude product was purified by flash column chromatography providing estrone trifluoromethanesulfonic ester **S23** (2.4 g yield). [19]

To a stirred solution of estrone trifluoromethanesulfonic ester **S23** (2.4 g, 5.9 mmol, 1.0 equiv.), Pd(PPh$_3$)$_4$ (207 mg, 3.0 mol%) and K_2CO_3 (2.5 g, 17.9 mmol, 3.0 equiv.). Freshly distilled and degassed 1,4-dioxane (50 ml) was added, and the resulting solution was stirred for 5 min. Then trimethylboroxine (TMB) (17.9 mmol, 3.0 equiv.) was introduced, and the vial was then sealed by a screw cap and stirred at 100 °C overnight. The crude mixture was filtered over a pad of Celite, and the solvent was removed under reduced pressure to remove the volatile materials. The crude product was purified by flash column chromatography silica gel (SiO_2; hexanes:EtOAc, 10:1 to 1:1 gradient; R_f = 0.81 in 9:2 hexanes:EtOAc) to afford) to afford the

product **10p** as white solid (0.82 g, 51% yield). Spectroscopic data are in agreement with those reported in the literature. [15, 20]

^{1}H NMR (600 MHz, CDCl$_3$) δ 7.19 (d, *J* = 7.9 Hz, 1H), 6.98 (dd, *J* = 1.9, 7.9 Hz, 1H), 6.93 (s, 1H), 2.91 – 2.86 (m, 2H), 2.50 (dd, *J* = 8.8, 18.9 Hz, 1H), 2.45 – 2.39 (m, 1H), 2.29 (s, 3H), 2.18 – 2.10 (m, 1H), 2.09 – 1.92 (m, 3H), 1.68 – 1.39 (m, 7H), 0.90 (s, 3H). (Coupling constants are uncorrected)

^{13}C NMR (101 MHz, CDCl$_3$) δ 137.0, 136.6, 135.7, 130.1, 126.9, 125.6, 50.8, 48.4, 44.6, 38.6, 36.2, 31.9, 29.7, 26.9, 26.1, 21.9, 21.2, 14.2.

Procedure for the synthesis of phosphonate diazo compounds:

Bestmann-Ohira method. [21-23]

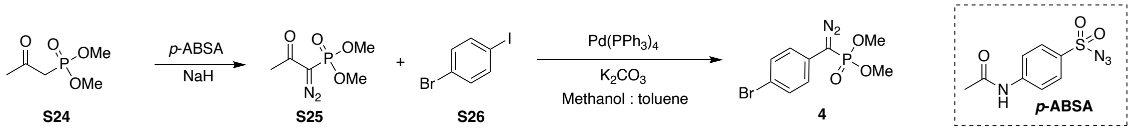

To a 250 mL flask was added a solution of dimethyl 2-oxopropylphosphonate **S24** (6.26 g, 37.7 mmol) in toluene (160 mL) and THF (30 mL) was stirred at 0 °C for 30 min. Sodium hydride (60% dispersion in mineral oil, 1.66 g, 41.5 mmol) was slowly added into the flask and the solution was stirred at 0 °C for 1 hour under nitrogen, *p*-ABSA (9.96 g, 41.5 mmol) was added in one portion and the reaction was warmed to room temperature and stirring was continued overnight. The reaction mixture was filtered through celite, and the residue was evaporated under reduced pressure to remove the volatile materials. The crude diazo product was purified by flash column chromatography on silica gel (petroleum ether : EtOAc, 1:1) giving

product S**25** (6.1g , 84%) as a yellow liquid. Spectroscopic data are in agreement with those reported in the literature. [24]

^{1}H NMR (400 MHz, $CDCl_3$) δ 3.79 (d, J_{HP} = 11.9 Hz, 6H), 2.21 (s, 3H). (Coupling constants are uncorrected)

^{13}C NMR (101 MHz, $CDCl_3$) δ 190.07 (d, J_{CP} = 13.1 Hz), 63.73 (d, J_{CP} = 220.5 Hz), 53.80 (d, J_{CP} = 5.6 Hz), 27.34 (d, J_{CP} = 1.3 Hz).

^{31}P NMR (162 MHz, $CDCl_3$) δ 14.21.

To a 50 mL flask was added $Pd(PPh_3)_4$ (232 mg, 5 mol%), K_2CO_3 (1.0 g, 8.0 mmol, 2.0 equiv.) and 1-bromo-4-iodobenzene **S26** (1.0 g, 4.0 mmol, 1.0 equiv.) were suspended in a mixture of methanol and toluene (20 mL, 1:1) under nitrogen. Dimethyl (1-diazo-2-oxopropyl)phosphonate **S25** (1.0 g, 5 mmol, 1.3 equiv.) was then added drop wise, and the resulting solution was stirred at room temperature for 5 h. The reaction mixture was filtered through a silica gel plug, eluted with ethyl acetate, and the filtrate solvent was evaporated in vacuo to remove the volatile materials. The crude diazo product was purified by flash column chromatography on silica gel (petroleum ether : EtOAc, 1:1) giving product **4** (90% yield) as a yellow liquid. Spectroscopic data are in agreement with those reported in the literature. (This procedure is used to synthesize all the (Aryl(diazo)methyl)phosphonates) derivatives) [25]

^{1}H NMR (600 MHz, $CDCl_3$) δ 7.47 (d, J = 8.7 Hz, 2H), 7.03 (d, J = 8.8 Hz, 2H), 3.81 (d, J = 12.0 Hz, 6H). (Coupling constants are uncorrected)

Dimethyl (diazo(phenyl)methyl)phosphonate (12a).

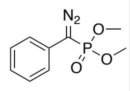

Bestmann-Ohira method was used for the title compound **12a**. Spectroscopic data are in agreement with those reported in the literature. The material was purified by flash chromatography (SiO_2; hexanes/EtOAc, 9:1 to 1:1 gradient; $R_f = 0.45$ in 1:1 hexanes/EtOAc), which afforded major product **12a** as a yellow liquid (80% yield). Spectroscopic data are in agreement with those reported in the literature. [25, 26]

^{1}H NMR (400 MHz, $CDCl_3$) δ 7.40 – 7.31 (m, 2H), 7.19 – 7.11 (m, 3H), 3.81 (d, J = 11.9 Hz, 6H). (Coupling constants are uncorrected)

Dimethyl (diazo(4-nitrophenyl)methyl)phosphonate (12b).

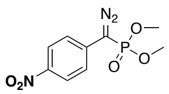

Bestmann-Ohira method was used for the title compound **12b**. Spectroscopic data are in agreement with those reported in the literature. The material was purified by flash chromatography (SiO_2; hexanes/EtOAc, 9:1 to 1:1 gradient; $R_f = 0.40$ in 1:1 hexanes/EtOAc), which afforded major product **12b** as a yellow liquid (85% yield). Spectroscopic data are in agreement with those reported in the literature. [25]

^{1}H NMR (400 MHz, $CDCl_3$) δ 8.21 (d, J = 8.9 Hz, 2H), 7.28 (d, J = 9.0 Hz, 2H), 3.85 (d, J = 12.0 Hz, 6H). (Coupling constants are uncorrected)

Dimethyl (diazo(3-methoxyphenyl)methyl)phosphonate (12c)

Bestmann-Ohira method was used for the title compound **12c**. Spectroscopic data are in agreement with those reported in the literature. The material was purified by flash

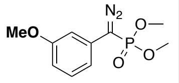
12c

chromatography (SiO_2; hexanes/EtOAc, 9:1 to 1:1 gradient; R_f = 0.40 in 1:1 hexanes/EtOAc), which afforded major product **12c** as a yellow liquid (55% yield). Spectroscopic data are in agreement with those reported in the literature. [25, 26]

^{1}H NMR (400 MHz, $CDCl_3$) δ 7.33 – 7.26 (m, 1H), 6.77 (d, *J* = 8.1 Hz, 1H), 6.74 – 6.69 (m, 2H), 3.83 (d, *J* = 11.9 Hz, 6H), 3.83 (s, 3H). (Coupling constants are uncorrected)

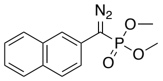
12d

Bestmann-Ohira method was used for the title compound **12d**. Spectroscopic data are in agreement with those reported in the literature. The material was purified by flash chromatography (SiO_2; hexanes/EtOAc, 9:1 to 1:1 gradient; R_f = 0.45 in 1:1 hexanes/EtOAc), which afforded major product **12d** as a yellow liquid (65% yield). Spectroscopic data are in agreement with those reported in the literature. [27]

^{1}H NMR (400 MHz, $CDCl_3$) δ 7.84 (d, *J* = 8.7 Hz, 1H), 7.78 (d, *J* = 9.0 Hz, 2H), 7.59 (d, *J* = 2.0 Hz, 1H), 7.51 – 7.38 (m, 2H), 7.28 (dd, *J* = 2.1, 8.7 Hz, 1H), 3.85 (d, *J* = 12.0 Hz, 6H). (Coupling constants are uncorrected)

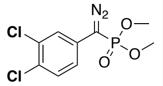
12e

Bestmann-Ohira method was used for the title compound **12e**. Spectroscopic data are in agreement with those reported in the literature. The material was purified by flash chromatography (SiO_2; hexanes/EtOAc, 9:1 to 1:1 gradient; R_f = 0.45 in 1:1 hexanes/EtOAc), which afforded major product **12e** as a yellow liquid (90% yield). Spectroscopic data are in agreement with those reported in the literature. [25]

^{1}H NMR (400 MHz, $CDCl_3$) δ 7.40 (d, *J* = 8.5 Hz, 0H), 7.22 (d, *J* = 2.3 Hz, 0H), 6.99 (dd, *J* = 2.3, 8.5 Hz, 0H), 3.82 (d, *J* = 12.0 Hz, 1H). (Coupling constants are uncorrected)

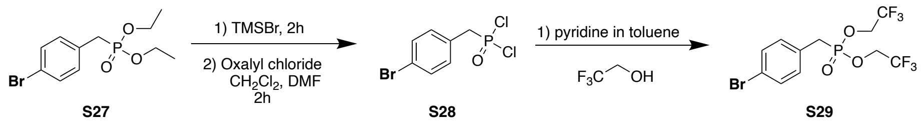

Bis(2,2,2-trifluoroethyl) (4-bromobenzyl)phosphonate (16).

Bromotrimethylsilane (TMSBr) (6.45 mL, 48.8 mmol) was added dropwise over 30 min to diethyl (4-bromobenzyl)phosphonate **S27** (5.0 g, 16.3 mmol) under N_2 at room temperature. The mixture was stirred for 2 h, then evaporated under reduced pressure to give crude product (6.4 g, 99%) as a light-yellow liquid. To this crude material in CH_2Cl_2 (35 mL) and DMF (40 μL) oxalyl chloride (4.2 mL, 49.0 mmol) was added dropwise accompanied by intense gas evolution. The mixture was stirred for 2 hours at room temperature, then evaporated under reduced pressure to give the crude product **S28** (4.6 g, 98%) as a brownish liquid. [28, 29]

To a solution of 2,2,2-trifluoroethanol (500 μL, 7.0 mmol) in pyridine (10 mL) (4-bromobenzyl)phosphonic dichloride **S28** (1.0 g, 3.5 mmol) in toluene (5 mL) was added at 50 °C under N_2. The mixture was stirred for overnight, then quenched with 1 M $NaHSO_4$ (100 mL) and the aqueous phase was extracted with toluene (2 × 20 mL). The combined organic phases were washed with H_2O (20 mL), 1 M $NaHCO_3$ (20 mL), and brine (2 × 15 mL), then dried over anhydrous Na_2SO_4 and was concentrated under vacuum to give crude product **S29** (0.62 g, 43%) as a white solid.

^{1}H NMR (400 MHz, $CDCl_3$) δ 7.48 (d, J = 8.5 Hz, 2H), 7.17 (dd, J = 2.7, 8.5 Hz, 2H), 4.39–4.16 (m, 4H), 3.28 (d, J_{HP} = 22.3 Hz, 2H). (Coupling constants are uncorrected)

^{13}C NMR (151 MHz, $CDCl_3$) δ 132.5 (d, J_{CP} = 3.4 Hz), 131.8 (d, J_{CP} = 7.1 Hz), 128.5 (d, J_{CP} = 9.7 Hz), 122.8 (dq, J_{CP} = 7.5, J_{CF} = 278.1 Hz), 122.3 (d, J_{CP} = 5.2 Hz), 62.7 (dq, J_{CP} = 6.5, J_{CF} = 38.1 Hz), 33.3 (d, J_{CP} = 140.8 Hz).

^{19}F NMR (376 MHz, $CDCl_3$) δ -75.30 (t, J = 8.0 Hz).

^{31}P NMR (162 MHz, $CDCl_3$) δ 28.78.

IR (neat): 3020, 2921, 2850, 1489, 1464, 1419, 1401, 1291, 1263, 1221, 1168, 1105, 1085, 1073, 1012, 966, 894, 856, 828, 814, 749, 709, 655, 647, 627, 550, 526, 503, 477, 445, 434, 426 cm^{-1}.

m.p. 167-172 °C.

HRMS (+p APCI) calcd. for $C_{11}H_{10}BrF_6O_3P$ $[M + H]^+$ 414.9527, found 414.9517.

Bis(2,2,2-trifluoroethyl) ((4-bromophenyl)(diazo)methyl)phosphonate (14).

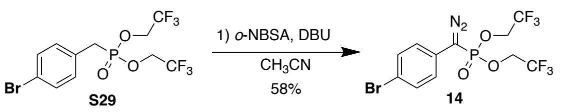

To a mixture of bis(2,2,2-trifluoroethyl) (4-bromobenzyl) phosphonate **S29** (0.62 g, 1.5 mmol) and ortho-nitrobenzenesulfonyl azide (*o*-NBSA) (0.41 g, 1.8 mmol) were dissolved in 25 mL anhydrous CH_3CN. The solution was kept stirring at room temperature and DBU (0.34 mL, 2.2 mmol) was added dropwise to the solution. The color of the solution gradually turned orange and it was quenched after 2 hours by diluting it with 50 mL Et_2O followed by adding 50 mL NH_4Cl (sat.) solution. Extract the aqueous layer with Et_2O (3×25 mL), and the organic layer was dried over anhydrous Na_2SO_4 and was concentrated under vacuum. The crude mixture was purified by flash column chromatography. (SiO_2; hexanes/EtOAc, 9:1 to 1:1 gradient; R_f = 0.68

in 1:1 hexanes/EtOAc) which afforded major product **14** as a yellow-orange oil (410 mg, 58% yield).

^{1}H NMR (600 MHz, $CDCl_3$) δ 7.51 (d, *J* = 8.3 Hz, 2H), 7.03 (d, *J* = 8.5 Hz, 2H), 4.53–4.37 (m, 4H). (Coupling constants are uncorrected)

^{13}C NMR (101 MHz, $CDCl_3$) δ 133.0, 124.8 (d, *J* = 4.7 Hz), 124.2 (d, *J* = 10.3 Hz), 122.5 (qd, *J* = 9.7, 277.6 Hz), 120.1, 63.3 (qd, *J* = 4.4, 38.3 Hz).

^{19}F NMR (565 MHz, $CDCl_3$) δ -74.94 (t, *J* = 8.1 Hz).

^{31}P NMR (243 MHz, $CDCl_3$) δ 20.22.

IR (neat): 2971, 2085, 1585, 1562, 1490, 1455, 1490, 1455, 1519, 1286, 1258, 1163, 1094, 1055, 1008, 960, 876, 812, 715, 657, 585, 550, 515, 482, 468, 433, 419, 413, 404 cm^{-1}.

HRMS (+p APCI) calcd. for $C_{11}H_8BrF_6N_2O_3P$ $[M + H]^+$ 440.9432, found 440.9423.

4. *Rhodium Catalyzed C–H Functionalization of Substrates and Characterization of Products.*

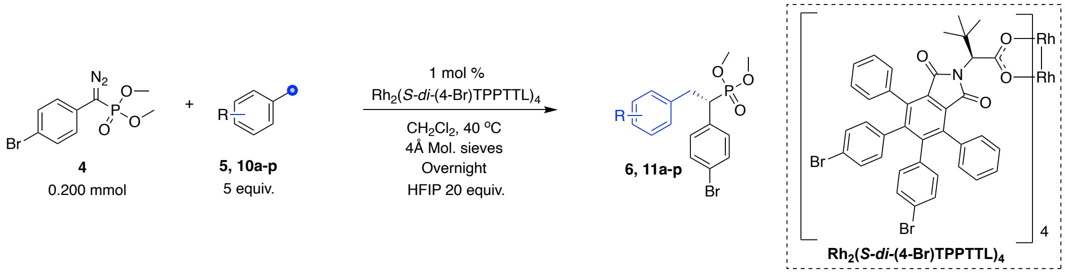

General Procedure for C-H Functionalization with Diazophosphonates.

A clean oven-dried and flame-dried 25.0 mL round bottom flask (flask-A) equipped with activated 4 Å molecular sieves, and a magnetic stir-bar was evacuated and purged with argon (2-3 times). After cooling down to room temperature, **10a-p** (5 equiv., 1.00 mmol) followed by Rh_2(*S*-*di*-(4-Br)TPPTTL)$_4$ (6.2 mg, 0.01 equiv.), and 1,1,1,3,3,3-hexafluoro-2-propanol (HFIP) (20 equiv.) were then added. The flask was once again evacuated and purged with argon (3-5 times) and anhydrous CH_2Cl_2 (5.0 mL) was added. The flask and its contents were then set to stir at reflux (40 °C) using a hotplate under an argon atmosphere. To a second oven-dried round bottom flask (flask-B), that was evacuated and purged with argon was added dimethyl ((4-bromophenyl)(diazo) methyl)phosphonate, **4** (61 mg, 0.20 mmol). flask-B and its contents were evacuated and purged with argon (2-3 times) and anhydrous CH_2Cl_2 (10.0 mL) was then added to obtain a solution of the diazo-compound. The solution was transferred into a 12.0 mL plastic syringe. Using a well-calibrated syringe-pump, a slow addition of the diazo solution into the stirring solution of flask-A under an inert atmosphere was initiated. After the complete addition of the solution (5 h), the residual diazo compound in the 12.0 mL plastic syringe was rinsed with anhydrous CH_2Cl_2 (1.0 mL) and transferred dropwise into the stirring reaction mixture of flask-A. The resulting solution was refluxed for 7 to 9 hours (overnight) before concentrating the solution under reduced pressure.

Purification by flash column chromatography on silica gel (hexanes : EtOAc) was used to afford the final products.

Dimethyl (*S*)-(1-(4-bromophenyl)-2-(4-isopropylphenyl) ethyl)phosphonate (6).

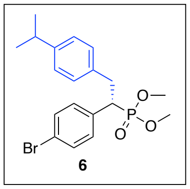

The material was purified by flash chromatography (SiO_2; hexanes/EtOAc, 9:1 to 1:1 gradient; R_f = 0.35 in 1:1 hexanes/EtOAc), which afforded major product **6** as a colorless oil (67 mg, 82% yield).

^{1}H NMR (600 MHz, $CDCl_3$) δ 7.39 (d, *J* = 8.6 Hz, 2H), 7.15 (dd, *J* = 2.3, 8.5 Hz, 2H), 7.02 (d, *J* = 7.7 Hz, 2H), 6.89 (d, *J* = 7.9 Hz, 2H), 3.70 (d, J_{HP} = 10.7 Hz, 3H), 3.52 (d, J_{HP} = 10.5 Hz, 3H), 3.40 (ddd, *J* = 3.9, 9.4, 13.8 Hz, 1H), 3.28 (ddd, *J* = 3.9, 11.2, 22.3 Hz, 1H), 3.10 (ddd, *J* = 9.3, 11.2, 14.2 Hz, 1H), 2.81 (hept, *J* = 7.0 Hz, 1H), 1.18 (dd, *J* = 1.4, 6.9 Hz, 6H). (Coupling constants are uncorrected)

^{13}C NMR (151 MHz, $CDCl_3$) δ 147.0, 135.7 (d, J_{CP} = 16.1 Hz), 134.7 (d, J_{CP} = 6.6 Hz), 131.6 (d, J_{CP} = 2.4 Hz), 131.1 (d, J_{CP} = 6.8 Hz), 128.6, 126.4, 121.2 (d, J_{CP} = 3.9 Hz), 53.1 (dd, J_{CP} = 7.2, 108.0 Hz), 45.7 (d, J_{CP} = 136.5 Hz), 35.6 (d, J_{CP} = 2.8 Hz), 33.6, 24.0, 23.9.

^{31}P NMR (243 MHz, $CDCl_3$) δ 29.83.

IR (neat): 2954, 1738, 1513, 1487, 1446, 1365,1231, 1215, 1054, 1031, 1011, 868, 826, 759, 634, 553 cm^{-1}.

$[\alpha]^{20}$ D: + 41.9° (c 0.40, $CHCl_3$)

HRMS (+p APCI) calcd. for $C_{19}H_{24}BrO_3P$ $[M + H]^+$ 411.0719, found 411.0718.

HPLC analysis: (Chiralcel ADH, 25 cm x 4.6 mm, 3.0% *i*-PrOH/hexanes, 1.0 mL/min, 230 nm) indicated 99% ee: t_R (major enantiomer) = 25.5 min, t_R (minor enantiomer) = 23.3 min.

Dimethyl (*S*)-(1-(4-bromophenyl)-2-(4-cyclohexylphenyl) ethyl) phosphonate (11a).

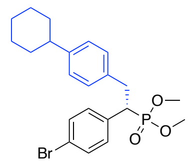

11a

The material was purified by flash chromatography (SiO_2; hexanes/EtOAc, 9:1 to 1:1 gradient; R_f = 0.40 in 1:1 hexanes/EtOAc), which afforded major product **11a** as a colorless oil (77 mg, 85% yield).

^{1}H NMR (600 MHz, CDCl$_3$) δ 7.39 (d, J = 8.2 Hz, 2H), 7.15 (dd, J = 2.3, 8.6 Hz, 2H), 7.00 (d, J = 7.9 Hz, 2H), 6.89 (d, J = 7.9 Hz, 2H), 3.69 (d, J_{HP} = 10.8 Hz, 3H), 3.51 (d, J_{HP} = 10.5 Hz, 3H), 3.39 (ddd, J = 3.9, 9.4, 13.6, Hz, 1H), 3.28 (ddd, J = 3.95, 10.95, 22.09 Hz, 1H), 3.09 (ddd, J = 9.3, 11.0, 14.3 Hz, 1H), 2.44–2.35 (m, 1H), 1.82 – 1.77 (m, 4H), 1.71 (d, J = 13.1 Hz, 1H), 1.38–1.28 (m, 4H), 1.25 – 1.18 (m, 1H). (Coupling constants are uncorrected)

^{13}C NMR (151 MHz, CDCl$_3$) δ 146.6, 136.1 (d, J_{CP} = 16.1 Hz), 135.0 (d, J_{CP} = 6.6 Hz), 131.9 (d, J_{CP} = 2.3 Hz), 131.4 (d, J_{CP} = 6.9 Hz), 129.0, 127.07, 121.6 (d, J_{CP} = 4.0 Hz), 53.5 (dd, J_{CP} = 7.2, 107.8 Hz), 46.0 (d, J_{CP} = 136.4 Hz), 44.4, 36.0 (d, J_{CP} = 2.9 Hz), 34.8 (d, J_{CP} = 5.6 Hz), 27.2, 26.5.

^{31}P NMR (243 MHz, CDCl$_3$) δ 29.85.

IR (neat): 2955, 1513, 1488, 1460, 1405, 1363, 1244, 1182, 1053, 1029, 1010, 908, 868, 825, 760, 729, 644, 634, 621, 575, 553, 479, 436, 406 cm^{-1}.

[α]^{20} D: + 109.2° (c 3.50, CHCl$_3$)

HRMS (+p APCI) calcd. for $C_{22}H_{28}BrO_3P$ [M + H]$^+$ 451.1032, found 451.1038.

HPLC analysis: (Chiralcel ADH, 25 cm x 4.6 mm, 2.0% *i*-PrOH/hexanes, 1.0 mL/min, 230 nm) indicated 98% ee: t_R (major enantiomer) = 33.2 min, t_R (minor enantiomer) = 29.8 min.

Dimethyl (*S*)-(2-([1,1'-biphenyl]-4-yl)-1-(4-bromophenyl) ethyl) phosphonate (11b).

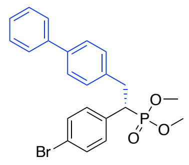

11b

The material was purified by flash chromatography (SiO_2; hexanes/EtOAc, 9:1 to 1:1 gradient; R_f = 0.39 in 1:1 hexanes/EtOAc), which afforded majorproduct **11b** as a white solid (79 mg, 89% yield). **m.p.** 139-142°C.

^{1}H NMR (400 MHz, $CDCl_3$) δ 7.57–7.49 (m, 2H), 7.43–7.36 (m, 6H), 7.34–7.28 (m, 1H), 7.17 (dd, J = 2.2, 8.6 Hz, 2H), 7.04 (d, J = 8.3 Hz, 2H), 3.73 (d, J_{HP} = 10.8 Hz, 3H), 3.54 (d, J_{HP} = 10.5 Hz, 3H), 3.48 (ddd, J = 3.7, 9.0, 13.3 Hz, 1H), 3.33 (ddd, J = 3.7, 11.3, 21.8 Hz, 1H), 3.17 (ddd, J = 8.8, 11.2, 13.9 Hz, 1H). (Coupling constants are uncorrected)

^{13}C NMR (101 MHz, $CDCl_3$) δ 141.0, 139.6, 138.0, 137.8, 134.8 (d, J_{CP} = 6.4 Hz), 132.0 (d, J_{CP} = 2.4 Hz), 131.4 (d, J_{CP} = 6.9 Hz), 129.5, 129.1, 127.5, 127.3 (d, J_{CP} = 7.9 Hz), 121.7 (d, J_{CP} = 3.9 Hz), 53.5 (dd, J_{CP} = 7.1, 76.7 Hz), 46.0 (d, J_{CP} = 136.9 Hz), 36.1 (d, J_{CP} = 2.6 Hz).

^{31}P NMR (162 MHz, $CDCl_3$) δ 29.63.

IR (neat): 2922, 1487, 1247, 1181, 1057, 1033, 1011, 833, 758, 699, 596, 553, 529, 512, 500, 292 cm^{-1}.

$[\alpha]^{20}$ D: + 68.7° (c 0.70, $CHCl_3$)

HRMS (+p APCI) calcd. for $C_{22}H_{22}BrO_3P$ $[M + H]^+$ 445.0563, found 445.0571.

HPLC analysis: (Chiralcel ADH, 25 cm x 4.6 mm, 4.0% *i*-PrOH/hexanes, 1.0 mL/min, 230 nm) indicated 91% ee: t_R (major enantiomer) = 28.7 min, t_R (minor enantiomer) = 26.5 min.

Dimethyl (*S*)-(1-(4-bromophenyl)-2-(4-(4,4,5,5-tetramethyl-1,3,2-dioxaborolan-2-yl)phenyl)ethyl)phosphonate (11c).

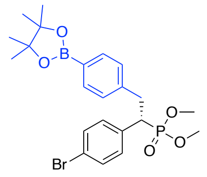

11c

The material was purified by flash chromatography (SiO_2; hexanes/EtOAc, 9:1 to 1:1 gradient; R_f = 0.38 in 1:1 hexanes/EtOAc), which afforded major product **11c** as a colorless oil (53 mg, 54% yield).

^{1}H NMR (600 MHz, $CDCl_3$) δ 7.60 (d, J = 7.7 Hz, 2H), 7.36 (d, J = 8.4 Hz, 2H), 7.12 (dd, J = 2.3, 8.5 Hz, 2H), 6.97 (d, J = 7.9 Hz, 2H), 3.72 (d, J_{HP} = 10.7 Hz, 3H), 3.52 (d, J_{HP} = 10.5 Hz, 3H), 3.44 (ddd, J = 3.8, 8.9, 13.3 Hz, 1H), 3.29 (ddd, J = 3.9, 11.4, 22.1 Hz, 1H), 3.12 (ddd, J = 8.8, 11.4, 14.0 Hz, 1H), 1.31 (bs, 12H). (Coupling constants are uncorrected)

^{13}C NMR (151 MHz, $CDCl_3$) δ 142.0 (d, J_{CP} = 16.4 Hz), 135.1, 134.6 (d, J_{CP} = 6.3 Hz), 132.0 (d, J_{CP} = 2.5 Hz), 132.4 (d, J_{CP} = 7.1 Hz), 128.6, 125.9, 121.7 (d, J_{CP} = 3.7 Hz), 84.9, 53.5 (dd, J_{CP} = 7.2, 112.1 Hz), 45.9 (d, J_{CP} = 137.1 Hz), 36.6 (d, J_{CP} = 2.7 Hz), 30.7, 25.2 (d, J_{CP} = 3.8 Hz).

^{31}P NMR (243 MHz, $CDCl_3$) δ 29.60.

IR (neat): 2978, 2953, 1611, 1487, 1447, 1398, 1340, 1320, 1248, 1213, 1183, 1166, 1144, 1089, 1056, 1032, 1012, 962, 859, 827, 771, 753, 734, 658, 613, 557, 519, 498 cm^{-1}.

$[\alpha]^{20}$ D: + 17.9° (c 0.30, $CHCl_3$)

HRMS (+p APCI) calcd. for $C_{22}H_{29}BBrO_5P$ $[M + H]^+$ 494.1138, found 494.1151.

HPLC analysis: (Chiralcel ADH, 25 cm x 4.6 mm, 5.0% *i*-PrOH/hexanes, 1.0 mL/min, 230 nm) indicated 98% ee: t_R (major enantiomer) = 12.6 min, t_R (minor enantiomer) = 15.2 min.

Dimethyl (*S*)-(1-(4-bromophenyl)-2-(4-(5-methylhex-1-yn-1-yl)phenyl)ethyl)phosphonate (11d).

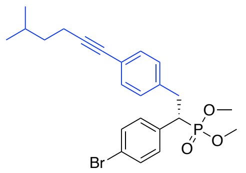

11d

The material was purified by flash chromatography (SiO_2; hexanes/EtOAc, 9:1 to 1:1 gradient; R_f = 0.40 in 1:1 hexanes/EtOAc), which afforded major product **11d** as a colorless oil (79 mg, 85%):

^{1}H NMR (600 MHz, $CDCl_3$) δ 7.37 (d, *J* = 8.1 Hz, 2H), 7.18 (d, *J* = 8.1 Hz, 2H), 7.10 (d, *J* = 8.0 Hz, 2H), 6.87 (d, *J* = 7.9 Hz, 2H), 3.71 (dd, J_{HP} = 1.3, 10.7 Hz, 3H), 3.52 (dd, J_{HP} = 1.3, 10.5 Hz, 3H), 3.40 (ddd, *J* = 3.9, 8.8, 13.3 Hz, 1H), 3.24 (ddd, *J* = 3.7, 11.1, 22.1 Hz, 1H), 3.08 (ddd, *J* = 9.0, 11.1, 14.3 Hz, 1H), 2.37 (t, *J* = 7.5 Hz, 2H), 1.72 (hept, *J* = 6.7 Hz, 1H), 1.47 (q, *J* = 7.4 Hz, 2H), 0.90 (d, *J* = 6.6 Hz, 6H). (Coupling constants are uncorrected)

^{13}C NMR (151 MHz, $CDCl_3$) δ 138.2 (d, J_{CP} = 16.1 Hz), 134.6 (d, J_{CP} = 6.2 Hz), 132.0 (d, J_{CP} = 2.3 Hz), 131.8, 131.4 (d, J_{CP} = 6.9 Hz), 129.0, 122.5, 121.7 (d, J_{CP} = 3.5 Hz), 90.8, 80.5, 53.5 (dd, J_{CP} = 7.1, 117.4 Hz), 46.0 (d, J_{CP} = 137.1 Hz), 38.0, 36.4 (d, J_{CP} = 2.5 Hz), 27.6, 22.5, 17.7.

^{31}P NMR (243 MHz, $CDCl_3$) δ 29.48.

IR (neat): 3028, 2954, 2869, 2238,1733, 1510, 1488, 1466, 1244, 1182, 1058, 1034, 1012, 908, 830, 731, 568 cm^{-1}.

$[\alpha]^{20}$ D: + 115.5° (c 0.50, $CHCl_3$)

HRMS (+p APCl) calcd. for $C_{23}H_{28}BrO_3P$ $[M + H]^+$ 463.1032, found 463.1039.

HPLC analysis: (Chiralcel ASH, 25 cm x 4.6 mm, 5.0% *i*-PrOH/hexanes, 1.0 mL/min, 254 nm) indicated 94% ee: t_R (minor enantiomer) = 9.4 min, t_R (major enantiomer) = 16.7 min.

Dimethyl (*S*,*E*)-(1-(4-bromophenyl)-2-(4-(2-(4,4,5,5-tetramethyl-1,3,2-dioxaborolan-2-yl)vinyl)phenyl)ethyl)phosphonate (11e).

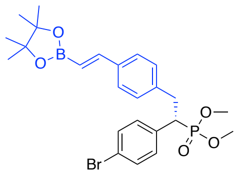

11e

The material was purified by flash chromatography (SiO_2; hexanes/EtOAc, 9:1 to 1:1 gradient; R_f = 0.45 in 9:1 hexanes/EtOAc), which afforded major product **11e** as a colorless oil (61 mg, 59%):

^{1}H NMR (600 MHz, $CDCl_3$) δ 7.37 (d, *J* = 8.3 Hz, 2H), 7.33–7.26 (m, 3H), 7.12 (dd, *J* = 2.2, 8.5 Hz, 2H), 6.94 (d, *J* = 8.2 Hz, 2H), 6.07 (d, *J* = 18.5 Hz, 1H), 3.71 (d, J_{HP} = 10.7 Hz, 3H), 3.52 (d, J_{HP} = 10.5 Hz, 3H), (ddd, *J* = 3.8, 8.7, 13.5 Hz, 1H), 3.27 (ddd, *J* = 4.1, 11.2, 22.2 Hz, 1H), 3.11 (ddd, *J* = 9.4, 11.2, 14.2 Hz, 1H), 1.29 (s, 12H). (Coupling constants are uncorrected)

^{13}C NMR (151 MHz, $CDCl_3$) δ 149.4, 139.8, 136.1, 134.6, 132.0 (d, J_{CP} = 2.2 Hz), 131.4 (d, J_{CP} = 6.9 Hz), 129.4, 127.4, 121.7, 83.7, 53.5 (dd, J_{CP} = 7.2, 116.0 Hz), 45.9 (d, J_{CP} = 137.1 Hz), 36.4, 30.1, 25.1 (d, J_{CP} = 3.3 Hz).

^{31}P NMR (243 MHz, $CDCl_3$) δ 29.54.

IR (neat): 2977, 1625, 1570, 1511, 1455, 1412, 1380, 1370, 1346, 1319, 1271, 1218, 1206, 1141, 1107, 995, 969, 900, 852, 845, 798, 760, 679, 644, 575, 495 cm^{-1}.

$[\alpha]^{20}$ D: + 43.6° (c 0.50, $CHCl_3$)

HRMS (+p APCl) calcd. for $C_{23}H_{28}BrO_3P$ $[M + H]^+$ 520.1294, found 520.1303.

SFC analysis: (Chiralpak AS-3, 3 μm particle size, 150 mm x 3 mm, 7.0% MeOH/IPA, with 0.2% Formic Acid, 2.5 mL/min, 280 nm) indicated 97% ee: t_R (minor enantiomer) = 1.4 min, t_R (major enantiomer) = 2.2 min.

Dimethyl ((1*S*)-1-(4-bromophenyl)-2-(4-((1'*s*,4'*R*)-4'-ethyl-[1,1'-bi(cyclohexan)]-4-yl)phenyl)ethyl)phosphonate (11f).

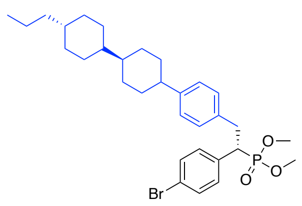

The material was purified by flash chromatography (SiO_2; hexanes/EtOAc, 10:0 to 9:1 gradient; R_f = 0.62 in 9:1 hexanes/EtOAc), which afforded major product **11f** as a white solid (102 mg, 89%).

m.p. 135-138 °C.

^{1}H NMR (600 MHz, $CDCl_3$) δ 7.38 (d, J = 7.8 Hz, 2H), 7.15 (dd, J = 2.3, 8.6 Hz, 2H), 6.99 (d, J = 8.1 Hz, 2H), 6.88 (d, J = 8.1 Hz, 2H), 3.69 (d, J_{HP} = 10.8 Hz, 3H), 3.51 (d, J_{HP} = 10.6 Hz, 3H), 3.38 (ddd, J = 3.9, 9.3, 13.8 Hz, 1H), 3.31 – 3.24 (m, 1H), 3.09 (ddd, J = 9.3, 11.1, 14.3 Hz, 1H), 2.34 (tt, J = 3.3, 12.2 Hz, 1H), 1.85 – 1.77 (m, 4H), 1.77 – 1.70 (m, 4H), 1.37 – 1.26 (m, 4H), 1.16 – 1.07 (m, 6H), 1.05 – 0.94 (m, 3H), 0.87 (t, J = 7.3 Hz, 3H). (Coupling constants are uncorrected).

^{13}C NMR (151 MHz, $CDCl_3$) δ 146.4, 136.1 (d, J_{CP} = 16.1 Hz), 135.0 (d, J_{CP} = 6.6 Hz), 131.9 (d, J_{CP} = 2.4 Hz), 131.4 (d, J_{CP} = 7.0 Hz), 128.9, 127.1, 121.6 (d, J_{CP} = 3.8 Hz), 53.5 (dd, J_{CP} = 7.2, 107.6 Hz), 46.0 (d, J_{CP} = 136.5 Hz), 44.5, 43.7, 43.3, 40.2, 38.0, 36.0 (d, J_{CP} = 2.8 Hz), 34.9 (d, J_{CP} = 3.9 Hz), 34.0, 30.7, 30.5, 20.4, 14.8.

^{31}P NMR (162 MHz, $CDCl_3$) δ 29.87.

IR (neat): 2950, 2913, 2848, 1513, 1486, 1449, 1405, 1247, 1197, 1098, 1052, 1022, 977, 879, 823, 773, 737, 707, 643, 623, 600 cm^{-1}.

$[\alpha]^{20}$ D: + 75.6° (c 0.95, $CHCl_3$)

HRMS (+p APCI) calcd. for $C_{31}H_{44}BrO_3P$ $[M + H]^+$ 575.2284, found 575.2295.

HPLC analysis: (Chiralcel ADH, 25 cm x 4.6 mm, 1.5% *i*-PrOH/hexanes, 1.0 mL/min, 230 nm) indicated 94% ee: t_R (minor enantiomer) = 24.6 min, t_R (major enantiomer) = 30.2 min.

The crystal structure information for compound (**11f**) is located in the crystallography section.

Dimethyl (*S*)-(1-(4-bromophenyl)-2-(4-methoxyphenyl)ethyl)phosphonate (11g).

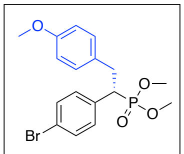

The material was purified by flash chromatography (SiO_2; hexanes/EtOAc, 10:0 to 1:1 gradient; R_f = 0.40 in 1:1 hexanes/EtOAc), which afforded **11g** as a colorless oil (64 mg, 80% yield).

^{1}H NMR (600 MHz, $CDCl_3$) δ 7.38 (d, J = 8.6 Hz, 2H), 7.12 (dd, J = 2.2, 8.5 Hz, 2H), 6.87 (d, J = 8.6 Hz, 2H), 6.69 (d, J = 8.7 Hz, 2H), 3.73 (s, 3H), 3.72 (d, J_{HP} = 10.5 Hz, 3H), 3.52 (d, J_{HP} = 10.5 Hz, 3H), 3.38 (ddd, J = 3.9, 8.7, 14.1 Hz, 1H), 3.24 (ddd, J = 3.8, 11.4, 22.1 Hz, 1H), 3.06 (ddd, J = 8.9, 11.4, 14.1 Hz, 1H). (Coupling constants are uncorrected)

^{13}C NMR (151 MHz, $CDCl_3$) δ 158.4, 134.8 (d, J_{CP} = 6.3 Hz), 132.0 (d, J_{CP} = 2.3 Hz), 131.4 (d, J_{CP} = 7.0 Hz), 130.7 (d, J_{CP} = 16.9 Hz), 130.1, 121.6 (d, J_{CP} = 3.9 Hz), 114.0, 55.5, 53.5 (dd, J_{CP} = 7.2, 112.2 Hz), 46.3 (d, J_{CP} = 136.2 Hz), 35.6 (d, J_{CP} = 2.8 Hz).

^{31}P NMR (243 MHz, $CDCl_3$) δ 29.83.

IR (neat): 2954, 2922, 2852, 1716, 1513, 1487, 1462, 1246, 1180, 1034, 1011, 904, 828, 730, 628, 534, 483 cm^{-1}.

$[\alpha]^{20}$ D: + 55.3° (c 0.40, $CHCl_3$)

HRMS (+p APCl) calcd. for $C_{17}H_{20}BrO_4P$ $[M + H]^+$ 399.0355, found 399.0360.

HPLC analysis: (Chiralcel ADH, 25 cm x 4.6 mm, 1.0% *i*-PrOH/hexanes, 1.0 mL/min, 230 nm) indicated 98% ee: t_R (minor enantiomer) = 24.7 min, t (major enantiomer) = 28.0 min.

Dimethyl (*S*)-(1-(4-bromophenyl)-2-(4-isopropyl-3-methoxyphenyl)ethyl)phosphonate (11h).

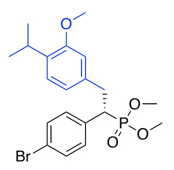

The material was purified by flash chromatography (SiO_2; hexanes/EtOAc, 10:0 to 1:1 gradient; R_f = 0.34 in 1:1 hexanes/EtOAc), which afforded **11h** as a colorless oil (77 mg, 87% yield).

^{1}H NMR (600 MHz, $CDCl_3$) δ 7.40 (d, J = 8.5 Hz, 2H), 7.17 (dd, J = 2.2, 8.5 Hz, 2H), 6.99 (d, J = 7.7 Hz, 1H), 6.54 (dd, J = 1.7, 7.7 Hz, 1H), 6.35 (d, J = 1.8 Hz, 1H), 3.71 (d, J_{HP} = 10.7 Hz, 3H), 3.64 (s, 3H), 3.54 (d, J_{HP} = 10.5 Hz, 3H), 3.39 (ddd, J = 3.7, 9.2, 13.4 Hz, 1H), 3.27 (ddd, J = 3.8, 11.4, 22.1 Hz, 1H), 3.18 (hept, J = 7.0 Hz, 1H), 3.10 (ddd, J = 8.7, 11.3, 14.1 Hz, 1H), 1.13 (dd, J = 4.8, 6.9 Hz, 6H). (Coupling constants are uncorrected)

^{13}C NMR (151 MHz, $CDCl_3$) δ 156.8, 137.2 (d, J_{CP} = 16.6 Hz), 135.5, 135.2 (d, J_{CP} = 6.5 Hz), 131.9 (d, J_{CP} = 2.5 Hz), 131.5 (d, J_{CP} = 7.1 Hz), 126.2, 121.6 (d, J_{CP} = 3.9 Hz), 121.1, 111.4, 55.6, 53.5 (dd, J_{CP} = 7.1, 108.5 Hz), 46.1 (d, J_{CP} = 136.5 Hz), 36.4 (d, J_{CP} = 2.8 Hz), 26.8, 23.0 (d, J_{CP} = 8.5 Hz).

^{31}P NMR (243 MHz, $CDCl_3$) δ 29.78.

IR (neat): 2955, 2855, 1610, 1577, 1506, 1488, 1463, 1417, 1349, 1251, 1184, 1162, 1093, 1057, 1035, 1011, 957, 867, 826, 768, 731, 636, 548, 501, 479 cm^{-1}.

$[\alpha]^{20}$ D: + 72.8° (c 0.50, $CHCl_3$)

HRMS (+p APCl) calcd. for $C_{20}H_{26}BrO_4P$ $[M + H]^+$ 441.0825, found 441.0824.

HPLC analysis: (Chiralcel ADH, 25 cm x 4.6 mm, 3.0% *i*-PrOH/hexanes, 0.5 mL/min, 230 nm) indicated 97% ee: t_R (major enantiomer) = 37.5 min, t (minor enantiomer) = 39.9 min.

Dimethyl (*S*)-(1-(4-bromophenyl)-2-(3-iodo-4-methoxyphenyl) ethyl)phosphonate (11i).

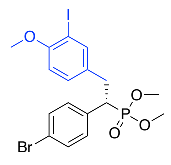

11i

The material was purified by flash chromatography (SiO_2; hexanes/EtOAc, 10:0 to 1:1 gradient; R_f = 0.34 in 1:1 hexanes/EtOAc), which afforded (**11i**) as a colorless oil (93 mg, 89% yield).

^{1}H NMR (400 MHz, $CDCl_3$) δ 7.44 (d, J = 2.2 Hz, 1H), 7.40 (d, J = 8.5 Hz, 2H), 7.13 (dd, J = 2.2, 8.6 Hz, 2H), 6.82 (dd, J = 2.2, 8.4 Hz, 1H), 6.58 (d, J = 8.4 Hz, 1H), 3.79 (s, 3H), 3.72 (d, J_{HP} = 10.8 Hz, 3H), 3.52 (d, J_{HP} = 10.6 Hz, 3H), 3.34 (ddd, J = 3.9, 8.8, 13.3 Hz, 1H), 3.21 (ddd, J = 4.1, 11.0, 21.9 Hz, 1H), 3.01 (ddd, J = 9.2, 11.1, 13.9 Hz, 1H). (Coupling constants are uncorrected)

^{13}C NMR (101 MHz, $CDCl_3$) δ 157.1, 139.9, 134.6 (d, J_{CP} = 6.4 Hz), 132.9 (d, J_{CP} = 16.5 Hz), 132.1 (d, J_{CP} = 2.4 Hz), 131.4(d, J_{CP} = 6.9 Hz), 130.2, 121.8 (d, J_{CP} = 3.8 Hz), 110.9, 86.1, 56.6, 53.5 (dd, J_{CP} = 7.2, 81.4 Hz), 46.2 (d, J_{CP} = 136.9 Hz), 35.2 (d, J_{CP} = 2.6 Hz).

^{31}P NMR (162 MHz, $CDCl_3$) δ 29.38.

IR (neat): 3001, 2960, 2833, 1582, 1566, 1472, 1439, 1414, 1326, 1283, 1241, 1227, 1181, 1168, 1159, 1090, 1060, 1028, 988, 859, 840, 821, 762, 679, 651, 563 cm^{-1}.

$[\alpha]^{20}$ D: + 116.2° (c 0.90, $CHCl_3$)

HRMS (+p APCl) calcd. for $C_{17}H_{19}BrO_4IP$ $[M + H]^+$ 524.9337, found 524.9322.

HPLC analysis: (Chiralcel ODH, 25 cm x 4.6 mm, 3.0% *i*-PrOH/hexanes, 1.0 mL/min, 230 nm) indicated 96% ee: t_R (major enantiomer) = 23.8 min, t (minor enantiomer) = 34.6 min.

Dimethyl (*S*)-(1-(4-bromophenyl)-2-(4-(but-2-yn-1-yloxy)phenyl)ethyl)phosphonate (11j).

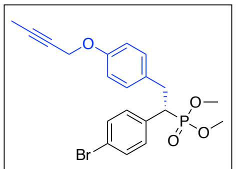

11j

The material was purified by flash chromatography (SiO_2; hexanes/EtOAc, 10:0 to 1:1 gradient; R_f = 0.38 in 1:1 hexanes/EtOAc), which afforded **11j** as a colorless oil (70 mg, 85% yield).

^{1}H NMR (600 MHz, $CDCl_3$) δ 7.38 (d, J = 8.4 Hz, 2H), 7.13 (dd, J = 2.2, 8.5 Hz, 2H), 6.88 (d, J = 8.6 Hz, 2H), 6.76 (d, J = 8.7 Hz, 2H), 4.56 (q, J = 2.3 Hz, 2H), 3.71 (d, J_{HP} = 10.7 Hz, 3H), 3.52 (d, J_{HP} = 10.5 Hz, 3H), 3.38 (ddd, J = 4.0, 8.9, 13.5 Hz, 1H), 3.24 (ddd, J = 4.0, 11.1, 22.1 Hz, 1H), 3.06 (ddd, J = 9.2, 11.2, 14.2 Hz, 1H), 1.84 (t, J = 2.3 Hz, 3H). (Coupling constants are uncorrected)

^{13}C NMR (151 MHz, $CDCl_3$) δ 156.8, 134.9 (d, J_{CP} = 6.2 Hz), 132.0 (d, J_{CP} = 2.2 Hz), 131.4 (d, J_{CP} = 6.9 Hz), 130.0, 125.9, 121.6 (d, J_{CP} = 3.7 Hz), 115.0, 84.0, 74.4, 56.8, 53.5 (dd, J_{CP} = 7.2, 111.5 Hz), 46.3 (d, J_{CP} = 136.5 Hz), 35.7 (d, J_{CP} = 2.7 Hz), 4.0.

^{31}P NMR (243 MHz, $CDCl_3$) δ 29.74.

IR (neat): 3032, 2981, 2920, 2227, 1612, 1585, 1509, 1488, 1455, 1370, 1293, 1217, 1177, 1141, 1110, 1052, 1012, 964, 854, 816, 770, 715, 702, 626, 576, 550, 508, 497, 489 cm^{-1}.

$[\alpha]^{20}$ D: + 70.5° (c 1.00, $CHCl_3$)

HRMS (+p APCl) calcd. for $C_{20}H_{22}BrO_4P$ $[M + H]^+$ 437.0512, found 437.0511.

HPLC analysis: (Chiralcel ODH, 25 cm x 4.6 mm, 5.0% *i*-PrOH/hexanes, 1.0 mL/min, 230 nm) indicated 98% ee: t_R (major enantiomer) = 22.3 min, *t* (minor enantiomer) = 31.6 min.

Dimethyl (*S*)-(1-(4-bromophenyl)-2-(4-((4-ethoxyphenyl)ethynyl)phenyl)ethyl)phosphonate (11k).

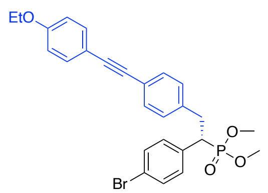

11k

The material was purified by flash chromatography (SiO_2; hexanes/EtOAc, 10:0 to 1:1 gradient; R_f = 0.39 in 1:1 hexanes/EtOAc), which afforded (**11k**) as a colorless oil (53 mg, 52% yield).

^{1}H NMR (600 MHz, CDCl$_3$) δ 7.41 (d, J = 8.7 Hz, 2H), 7.38 (d, J = 8.4 Hz, 2H), 7.30 (d, J = 8.1 Hz, 2H), 7.12 (dd, J = 2.2, 8.5 Hz, 2H), 6.93 (d, J = 8.2 Hz, 2H), 6.84 (d, J = 8.7 Hz, 2H), 4.04 (q, J = 7.0 Hz, 2H), 3.73 (d, J_{HP} = 10.8 Hz, 3H), 3.53 (d, J_{HP} = 10.6 Hz, 3H), 3.43 (ddd, J = 3.9, 8.7, 14.1 Hz, 1H), 3.26 (ddd, J = 3.9, 11.6, 22.1 Hz, 1H), 3.11 (ddd, J = 9.2, 11.4, 14.0 Hz, 1H), 1.42 (t, J = 7.0 Hz, 4H). (Coupling constants are uncorrected)

^{13}C NMR (151 MHz, CDCl$_3$) δ 159.3, 138.7 (d, J_{CP} = 16.4 Hz), 134.5 (d, J_{CP} = 6.5 Hz), 133.3, 132.1 (d, J_{CP} = 2.3 Hz), 131.7, 131.4 (d, J_{CP} = 6.8 Hz), 129.1, 122.1, 121.8 (d, J_{CP} = 3.8 Hz), 115.5, 114.8, 89.8, 88.0, 63.9, 53.6 (dd, J_{CP} = 7.2, 119.5 Hz), 46.0 (d, J_{CP} = 137.2 Hz), 36.5 (d, J_{CP} = 2.6 Hz), 15.1.

^{31}P NMR (243 MHz, CDCl$_3$) δ 29.44.

IR (neat): 2979, 2952, 2926, 2849, 1602, 1569, 1516, 1488, 1476, 1392, 1284, 1246, 1174, 1115, 1034, 1011, 921, 872, 830, 769, 706, 635, 617, 595, 570, 549 cm^{-1}.

$[\alpha]^{20}$ D: + 41.9° (c 0.70, CHCl$_3$)

HRMS (+p APCI) calcd. for $C_{20}H_{22}BrO_4P$ $[M + H]^+$ 437.0512, found 437.0511.

SFC analysis: (ChiralCel OJ-3, 3 µm particle size, 150 mm x 3 mm, 10.0% MeOH/IPA with 0.2% Formic Acid, 2.5 mL/min, 280 nm) indicated 94% ee: t_R (major enantiomer) = 4.9 min, t_R (minor enantiomer) = 5.6 min.

Dimethyl ((1*S*)-2-(6-(3-(adamantan-1-yl)-4-methoxyphenyl)naphthalen-2-yl)-1-(4-bromophenyl)ethyl)phosphonate (11l).

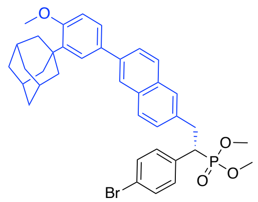

11l

The material was purified by flash chromatography (SiO_2; hexanes/EtOAc, 10:0 to 1:1 gradient; R_f = 0.39 in 1:1 hexanes/EtOAc), which afforded **11l** as a white solid (116 mg, 88% yield).

m.p. 144-148 °C.

^{1}H NMR (600 MHz, CDCl$_3$): δ 7.87 (d, *J* = 1.8 Hz, 1H), 7.74 – 7.64 (m, 3H), 7.53 (d, *J* = 2.4 Hz, 1H), 7.48 (dd, *J* = 2.3, 8.4 Hz, 1H), 7.43 (d, *J* = 1.5 Hz, 1H), 7.37 (d, *J* = 8.3 Hz, 2H), 7.17 (dd, *J* = 2.1, 6.4 Hz, 2H), 7.11 (dd, *J* = 1.8, 8.4 Hz, 1H), 7.00 – 6.95 (m, 1H), 3.89 (s, 3H), 3.74 (d, J_{HP} = 10.7 Hz, 3H), 3.61 (ddd, *J* = 3.8, 8.9, 13.6 Hz, 1H), 3.55 (d, J_{HP} = 10.7 Hz, 3H), 3.46 – 3.37 (m, 1H), 3.29 (ddd, *J* = 8.9, 11.3, 14.1 Hz, 1H), 2.16 (d, *J* = 2.9 Hz, 6H), 2.09 (d, *J* = 4.6 Hz, 3H), 1.82 – 1.75 (m, 6H). (Coupling constants are uncorrected)

^{13}C NMR (101 MHz, CDCl$_3$) δ 158.9, 139.2, 139.1, 136.0, 135.9, 134.7 (d, *J* = 6.2 Hz), 133.5, 132.8, 132.5, 132.0 (d, *J* = 2.5 Hz), 131.4 (d, *J* = 6.8 Hz), 128.5, 128.2, 127.6 (d, *J* = 19.0 Hz), 126.2 (d, *J* = 2.1 Hz), 125.9, 125.1, 121.68 (d, *J* = 3.8 Hz), 112.4, 55.5, 53.6 (dd, *J* = 7.2, 114.4 Hz), 46.1 (d, *J* = 136.7 Hz), 41.0, 37.5 (d, *J* = 3.9 Hz), 36.7 (d, *J* = 2.7 Hz), 29.5.

^{31}P NMR (243 MHz, CDCl$_3$) δ 29.64.

IR (neat): 2902, 2849, 1488, 1237, 1182, 1058, 1031, 880, 817, 757, 561, 492, 287, 479, 429, 217, 412, 406 cm^{-1}.

[α]^{20} D: + 80.2° (c 2.50, CHCl$_3$)

HRMS (+p APCI) calcd. for $C_{37}H_{40}BrO_4P$ $[M + H]^+$ 659.1920, found 659.1927.

SFC analysis: (Chiralpak AS-3, 3 μm particle size, 150 mm x 3 mm, 10.0% MeOH/IPA, with 0.2% Formic Acid, 2.5 mL/min, 280 nm) indicated 94% ee: t_R (minor enantiomer) = 1.8 min, t_R (major enantiomer) = 2.2 min.

Methyl (*S*)-2-(6-((*S*)-2-(4-bromophenyl)-2-(dimethoxyphosphoryl)ethyl)naphthalen-2-yl)propanoate (11m).

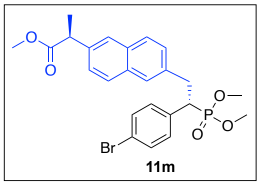

The material was purified by flash chromatography (SiO_2; hexanes/EtOAc, 10:0 to 1:1 gradient; R_f = 0.35 in 1:1 hexanes/EtOAc), which afforded **11m** as a colorless oil (81 mg mg, 80% yield).

^{1}H NMR (400 MHz, CDCl$_3$) δ 7.63 (d, J = 8.4 Hz, 3H), 7.42 – 7.32 (m, 4H), 7.15 (dd, J = 2.3, 8.6 Hz, 2H), 7.10 (dd, J = 1.8, 8.4 Hz, 1H), 3.85 (q, J = 7.1 Hz, 1H), 3.73 (d, J_{HP} = 10.7 Hz, 3H), 3.65 (s, 3H), 3.64 – 3.50 (m, 4H), 3.39 (ddd, J = 3.8, 11.2, 21.7 Hz, 1H), 3.27 (ddd, J = 8.4, 11.4, 14.0 Hz, 1H), 1.56 (d, J = 7.3 Hz, 3H). (Coupling constants are uncorrected)

^{13}C NMR (101 MHz, CDCl$_3$) δ 175.3, 138.1, 136.3 (d, J_{CP} = 16.6 Hz), 134.7 (d, J_{CP} = 6.5 Hz), 132.7 (d, J_{CP} = 33.5 Hz), 132.0 (d, J_{CP} = 2.4 Hz), 131.4 (d, J_{CP} = 6.9 Hz), 128.3 (d, J_{CP} = 6.4 Hz), 127.6 (d, J_{CP} = 14.1 Hz), 126.2 (d, J_{CP} = 9.8 Hz), 121.7, 53.6 (dd, J_{CP} = 7.2, 75.3 Hz), 52.4, 46.1 (d, J_{CP} = 137.0 Hz), 45.8, 36.6, 18.9.

^{31}P NMR (162 MHz, CDCl$_3$) δ 29.58.

IR (neat): 2952, 1851, 1732, 1606, 1488, 1455, 1377, 1329, 1246, 1197, 1056, 1031, 1011, 888, 820, 767, 732, 556, 499, 479 cm^{-1}.

$[\alpha]^{20}$ D: + 85.5° (c 1.00, CHCl$_3$)

HRMS (+p APCI) calcd. for $C_{24}H_{26}BrO_5P$ $[M + H]^+$ 505.0774, found 505.0777.

SFC analysis: (Chiralpak AS-3, 3 μm particle size, 150 mm x 3 mm, 5.0% MeOH/IPA, with 0.2% Formic Acid, 2.5 mL/min, 230 nm) indicated 84% ee: t_R (minor enantiomer) = 1.4 min, t_R (major enantiomer) = 1.8 min.

Propyl (*S*)-5-(5-(2-(4-bromophenyl)-2-(dimethoxyphosphoryl)ethyl)-2-methylphenoxy)-2,2-dimethylpentanoate (11n).

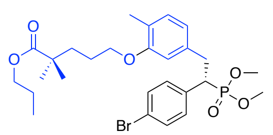

11n

The material was purified by flash chromatography (SiO_2; hexanes/EtOAc, 10:0 to 1:1 gradient; R_f = 0.40 in 1:1 hexanes/EtOAc)), which afforded **11n** as a colorless oil (66 mg, 58% yield).

^{1}H NMR (600 MHz, $CDCl_3$) δ 7.39 (d, J = 8.4 Hz, 2H), 7.14 (dd, J = 2.2, 8.6 Hz, 2H), 6.90 (dd, J = 0.9, 7.5 Hz, 1H), 6.45 (dd, J = 1.6, 7.5 Hz, 1H), 6.28 (d, J = 1.7 Hz, 1H), 4.02 (t, J = 6.6 Hz, 2H), 3.79 – 3.69 (m, 4H), 3.68 – 3.60 (m, 1H), 3.54 (d, J_{HP} = 10.5 Hz, 3H), 3.37 (ddd, J = 3.7, 8.8, 14.2Hz, 1H), 3.25 (ddd, J = 3.6, 11.5, 22.1 Hz, 1H), 3.06 (ddd, J = 8.3, 11.5, 14.0 Hz, 1H), 2.10 (s, 3H), 1.67 – 1.58 (m, 6H), 1.21 (s, 7H), 0.94 (t, J = 7.4 Hz, 3H). (Coupling constants are uncorrected)

^{13}C NMR (151 MHz, $CDCl_3$) δ 178.2, 157.1, 137.3 (d, J_{CP} = 16.7 Hz), 135.0 (d, J_{CP} = 6.3 Hz), 131.9 (d, J_{CP} = 2.3 Hz), 131.5 (d, J_{CP} = 6.8 Hz), 130.7, 125.2, 121.6 (d, J_{CP} = 3.8 Hz), 120.8, 111.9, 68.3, 66.3, 53.5 (dd, J_{CP} = 7.0, 107.6 Hz), 46.2 (d, J_{CP} = 136.5 Hz), 42.4, 37.4, 36.4 (d, J_{CP} = 2.6 Hz), 25.5 (d, J_{CP} = 15.5 Hz), 22.4, 16.1, 10.8.

^{31}P NMR (243 MHz, $CDCl_3$) δ 29.76.

IR (neat): 2954, 2921, 2850, 1724, 1509, 1488, 1473, 1253, 1192, 1144, 1057, 1034, 1012, 871, 828, 770, 560, 552, 540, 534, 498, 490, 485, 477 cm^{-1}.

$[\alpha]^{20}$ D: + 55.3° (c 0.80, $CHCl_3$)

HRMS (+p APCI) calcd. for $C_{27}H_{38}BrO_6P$ [M + H]$^+$ 569.1662, found 569.1671.

SFC analysis: (Chiralpak AS-3, 3 μm particle size, 150 mm x 3 mm, 5.0% MeOH/IPA, with 0.2% Formic Acid, 2.5 mL/min, 230 nm) indicated 90% ee: t_R (minor enantiomer) = 2.3 min, t_R (major enantiomer) = 2.7 min.

Ethyl (*S*)-2-(4-(2-(4-bromophenyl)-2-(dimethoxyphosphoryl)ethyl)phenoxy)-2-methylpropanoate (11o).

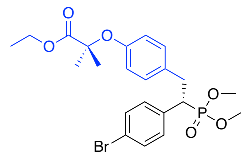

11o

The material was purified by flash chromatography (SiO_2; hexanes/EtOAc, 10:0 to 1:1 gradient; R_f = 0.32 in 1:1 hexanes/EtOAc), which afforded **11o** as a colorless oil (62 mg, 62% yield).

^{1}H NMR (400 MHz, CDCl$_3$) δ 7.36 (d, J = 8.6 Hz, 2H), 7.10 (dd, J = 2.3, 8.6 Hz, 2H), 6.82 (d, J = 8.6 Hz, 2H), 6.64 (d, J = 8.7 Hz, 2H), 4.18 (qd, J = 1.5, 7.1 Hz, 2H), 3.70 (d, J_{HP} = 10.8 Hz, 3H), 3.51 (d, J_{HP} = 10.5 Hz, 3H), 3.36 (ddd, J = 4.0, 8.7, 13.2 Hz, 1H), 3.21 (ddd, J = 4.1, 11.2, 22.1 Hz, 1H), 3.03 (ddd, J = 9.5, 11.2, 14.1 Hz, 1H), 1.53 (s, 6H), 1.18 (t, J = 7.1 Hz, 3H). (Coupling constants are uncorrected)

^{13}C NMR (101 MHz, CDCl$_3$) δ 174.6, 154.3, 134.8 (d, J_{CP} = 6.2 Hz), 132.4 (d, J_{CP} = 16.1 Hz), 131.9 (d, J_{CP} = 2.2 Hz), 131.4 (d, J_{CP} = 7.1 Hz), 129.8, 121.6 (d, J_{CP} = 3.8 Hz), 119.3, 79.4, 61.7, 53.5 (dd, J_{CP} = 7.1, 114.5 Hz), 46.2 (d, J_{CP} = 136.7 Hz), 35.8 (d, J_{CP} = 2.7 Hz), 25.7 (d, J_{CP} = 32.9 Hz), 14.4.

^{31}P NMR (243 MHz, CDCl$_3$) δ 29.71.

IR (neat): 2988, 2553, 2850, 1731, 1508, 1488, 1466, 1382, 1283, 1239, 1178, 1140, 1056, 1031, 1011, 971, 912, 830, 766, 731, 645, 559, 520, 508, 476 cm^{-1}.

$[\alpha]^{20}$ D: + 28.7° (c 0.40, CHCl$_3$)

HRMS (+p APCI) calcd. for $C_{22}H_{28}BrO_6P$ $[M + H]^+$ 499.0880, found 499.0885.

SFC analysis: (Chiralpak AS-3, 3 µm particle size, 150 mm x 3 mm, 5.0% MeOH/IPA, with 0.2% Formic Acid, 2.5 mL/min, 230 nm) indicated 84% ee: t_R (minor enantiomer) = 1.4 min, t_R (major enantiomer) = 2.0 min.

Dimethyl ((1*S*)-1-(4-bromophenyl)-2-((8*R*,9*S*,13*S*)-13-methyl-17-oxo-7,8,9,11,12,13,14,15,16,17-decahydro-6*H*-cyclopenta[*a*]phenanthren-3-yl)ethyl)phosphonate (11p).

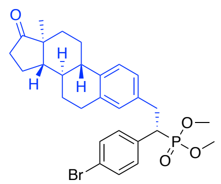

11p

The material was purified by flash chromatography (SiO_2; hexanes/EtOAc, 10:0 to 1:1 gradient; R_f = 0.38 in 1:1 hexanes/EtOAc), which afforded **11p** as a white solid (99 mg, 91% yield). **m.p.** 123-126 °C.

^{1}H NMR (600 MHz, $CDCl_3$) δ 7.40 (d, J = 8.3 Hz, 2H), 7.16 (d, J = 8.3 Hz, 2H), 7.08 (d, J = 7.9 Hz, 1H), 6.76 (d, J = 7.9 Hz, 1H), 6.68 (s, 1H), 3.72 (d, J_{HP} = 10.6 Hz, 2H), 3.53 (d, J_{HP} = 10.6 Hz, 3H), 3.37 (t, J = 11.9 Hz, 1H), 3.33 – 3.25 (m, 1H), 3.08 (q, J = 11.1 Hz, 1H), 2.84 – 2.70 (m, 2H), 2.49 (dd, J = 18.9, 8.8 Hz, 1H,) 2.36 – 2.32 (m, 1H), 2.21 (d, J = 10.8 Hz, 1H), 2.19 – 2.08 (m, 1H), 2.07 – 1.99 (m, 1H), 1.99 – 1.91 (m, 2H), 1.65 – 1.32 (m, 6H), 0.89 (s, 3H). (Coupling constants are uncorrected)

^{13}C NMR (151 MHz, $CDCl_3$) δ 138.2, 136.7, 136.2 (d, J_{CP} = 16.0 Hz), 135.0 (d, J_{CP} = 7.0 Hz), 131.9, 131.5 (d, J_{CP} = 6.7 Hz), 129.8, 126.3, 125.6, 121.6, 53.4 (dd, J_{CP} = 7.5, 107.9 Hz), 50.9, 48.3, 45.8 (d, J_{CP} = 136.9 Hz), 44.6, 38.4, 36.2, 35.7, 31.9, 30.1, 29.6, 26.8, 26.0, 21.9, 14.2.

^{31}P NMR (243 MHz, $CDCl_3$) δ 29.83.

IR (neat): 2926, 1737, 1487, 1248, 1055, 1032, 1011, 824, 767, 552, 515, 504, 493, 485, 471, 466 cm^{-1}.

$[\alpha]^{20}$ D: + 78.4° (c 1.00, $CHCl_3$)

HRMS (+p APCl) calcd. for $C_{28}H_{34}BrO_4P$ $[M + H]^+$ 545.1451, found 545.1463.

HPLC analysis: (Chiralcel ADH, 25 cm x 4.6 mm, 7.0% *i*-PrOH/hexanes, 1.0 mL/min, 230 nm) indicated 98% ee: t_R (major enantiomer) = 36.8 min, *t* (minor enantiomer) = 46.9 min.

The crystal structure information for compound (**11p**) is located in the crystallography section.

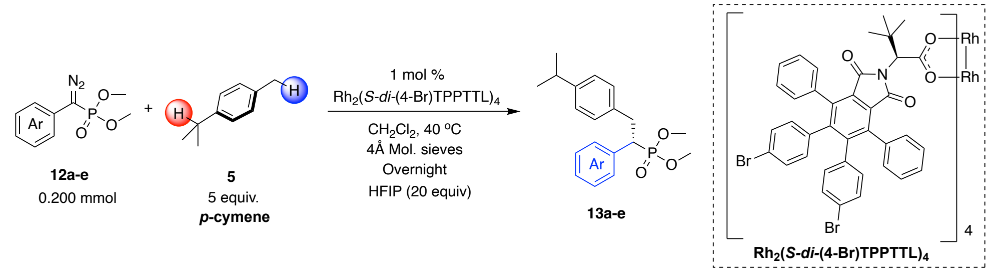

A clean oven-dried and flame-dried 25.0 mL round bottom flask (flask-A) equipped with activated 4 Å molecular sieves, and a magnetic stir-bar was evacuated and purged with argon (2-3 times). After cooling down to room temperature, **5** (5 equiv., 1.00 mmol) followed by Rh_2(*S*-*di*-(4-Br)TPPTTL)$_4$ (6.2 mg, 0.01 equiv.), and 1,1,1,3,3,3-hexafluoro-2-propanol (HFIP) (20 equiv.) were then added. The flask was once again evacuated and purged with argon (3-5 times) and anhydrous CH_2Cl_2 (5.0 mL) was added. The flask and its contents were then set to stir at reflux (40 $^{\circ}$C) using a hotplate under an argon atmosphere. To a second oven-dried round bottom flask (flask-B), that was evacuated and purged with argon was added aryldiazophosphonates **12a-e** (0.20 mmol). flask-B and its contents were evacuated and purged with argon (2-3 times) and anhydrous CH_2Cl_2 (10.0 mL) was then added to obtain a solution of the diazo-compound. The solution was transferred into a 12.0 mL plastic syringe. Using a well-calibrated syringe-pump, a slow addition of the diazo solution into the stirring solution of flask-A under an inert atmosphere was initiated. After the complete addition of the solution (5 h), the residual diazo compound in the 12.0 mL plastic syringe was rinsed with anhydrous CH_2Cl_2 (1.0 mL) and

transferred dropwise into the stirring reaction mixture of flask-A. The resulting solution was refluxed for 7 to 9 hours (overnight) before concentrating the solution under reduced pressure. Purification by flash column chromatography on silica gel (hexanes : EtOAc) was used to afford the final products.

Dimethyl (*S*)-(2-(4-isopropylphenyl)-1-phenylethyl)phosphonate (13a)

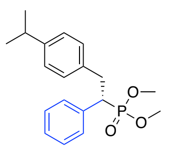

13a

The material was purified by flash chromatography (SiO_2; hexanes/EtOAc, 10:0 to 1:1 gradient; R_f = 0.38 in 1:1 hexanes/EtOAc), which afforded **13a** as a colorless oil (63 mg, 90% yield).

^{1}H NMR (600 MHz, $CDCl_3$) δ 7.27 – 7.20 (m, 5H), 7.18 (td, J = 1.9, 6.6 Hz, 1H), 6.97 (d, J = 7.9 Hz, 2H), 6.88 (d, J = 7.9 Hz, 2H), 3.64 (d, J_{HP} = 10.7 Hz, 3H), 3.42 (d, J_{HP} = 10.7 Hz, 3H), 3.37 (td, J = 5.0, 9.9 Hz, 1H), 3.30 (ddd, J = 4.0, 10.6, 22.1 Hz, 1H), 3.14 (dt, J = 10.4, 14.0 Hz, 1H), 2.76 (hept, J = 6.9 Hz, 1H), 1.13 (dd, J = 2.0, 7.0 Hz, 6H). (Coupling constants are uncorrected)

^{13}C NMR (151 MHz, $CDCl_3$) δ 147.1, 136.6 (d, J_{CP} = 15.8 Hz), 135.9 (d, J_{CP} = 6.6 Hz), 129.8 (d, J_{CP} = 6.7 Hz), 129.0, 128.8 (d, J_{CP} = 2.5 Hz), 127.6 (d, J_{CP} = 3.1 Hz), 126.6, 53.4 (dd, J_{CP} = 7.1, 125.5 Hz), 46.6 (d, J_{CP} = 135.9 Hz), 36.2 (d, J_{CP} = 2.7 Hz), 33.9, 24.3 (d, J_{CP} = 3.3 Hz).

^{31}P NMR (243 MHz, CDCl3) δ 30.67.

IR (neat): 2956, 1513, 1495, 1454, 1248, 1248, 1183, 1056, 1029, 826, 775, 762, 747, 699, 592, 574, 563, 563, 549, 535, 525, 510, 506, 497, 489, 483 cm^{-1}.

[α]^{20} D: + 93.5° (c 4.50, $CHCl_3$)

HRMS (+p APCl) calcd. for $C_{19}H_{25}O_3P$ $[M + H]^+$ 333.1614, found 333.1619.

HPLC analysis: (Chiralcel ADH, 25 cm x 4.6 mm, 2.0% *i*-PrOH/hexanes, 1.0 mL/min, 230 nm) indicated 98% ee: t_R (minor enantiomer) = 21.1 min, t (major enantiomer) = 28.6 min.

Dimethyl (2-(4-isopropylphenyl)-1-(4-nitrophenyl)ethyl)phosphonate (13b)

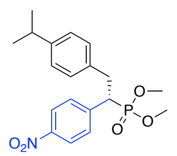

13b

The material was purified by flash chromatography (SiO_2; hexanes/EtOAc, 10:0 to 1:1 gradient; R_f = 0.32 in 1:1 hexanes/EtOAc), which afforded **13b** as a colorless oil (48 mg, 60%).

^{1}H NMR (600 MHz, $CDCl_3$) δ 8.12 (d, J = 8.7 Hz, 2H), 7.44 (dd, J = 2.2, 8.7 Hz, 2H), 7.02 (d, J = 8.0 Hz, 2H), 6.88 (d, J = 8.0 Hz, 2H), 3.74 (d, J_{HP} = 10.7 Hz, 3H), 3.58 (d, J_{HP} = 10.7 Hz, 3H), 3.51 – 3.42 (m, 2H), 3.15 (dt, J = 10.0, 13.4 Hz, 1H), 2.80 (p, J = 6.9 Hz, 1H), 1.16 (dd, J = 2.2, 6.9 Hz, 6H). (Coupling constants are uncorrected)

^{13}C NMR (151 MHz, $CDCl_3$) δ 147.7, 147.5, 143.9 (d, J_{CP} = 6.4 Hz), 135.4 (d, J_{CP} = 16.0 Hz), 130.6 (d, J_{CP} = 6.8 Hz), 128.9, 126.9, 124,0 (d, J_{CP} = 2.3 Hz), 53.6 (dd, J_{CP} = 7.0, 66.7 Hz), 46.7 (d, J_{CP} = 136.2 Hz), 36.0 (d, J_{CP} = 3.3 Hz), 33.9, 24.2 (d, J_{CP} = 4.2 Hz).

^{31}P NMR (243 MHz, $CDCl_3$) δ 28.73.

IR (neat): 2955, 1513, 1488, 1460, 1405, 1363, 1244, 1182, 1053, 1029, 1010, 908, 868, 825, 760, 729, 644, 634, 621, 575, 553, 479 cm^{-1}.

$[\alpha]^{20}$ D: + 82.2° (c 2.00, $CHCl_3$)

HRMS (+p APCl) calcd. for $C_{19}H_{24}NO_5P$ $[M + H]^+$ 378.1465, found 378.1473

HPLC analysis: (Chiralcel ADH, 25 cm x 4.6 mm, 2.0% *i*-PrOH/hexanes, 1.0 mL/min, 254 nm) indicated 98% ee: t_R (minor enantiomer) = 25.1 min, *t* (major enantiomer) = 26.6 min.

Dimethyl (2-(4-isopropylphenyl)-1-(3-methoxyphenyl)ethyl)phosphonate (13c)

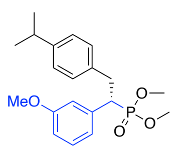

The material was purified by flash chromatography (SiO_2; hexanes/EtOAc, 10:0 to 1:1 gradient; R_f = 0.38 in 1:1 hexanes/EtOAc), which afforded **13c** as a colorless oil (51 mg, 71%).

^{1}H NMR (600 MHz, $CDCl_3$) δ 7.18 (t, J = 7.9 Hz, 1H), 7.02 (d, J = 8.0 Hz, 2H), 6.93 (d, J = 8.0 Hz, 2H), 6.89 (d, J = 7.6 Hz, 1H), 6.82 (d, J = 2.2 Hz, 1H), 6.76 (dd, J = 2.2, 8.3 Hz, 1H), 3.76 (s, 3H), 3.68 (d, J_{HP} = 10.7 Hz, 3H), 3.49 (d, J_{HP} = 10.5 Hz, 3H), 3.39 (ddd, J = 4.1, 10.0, 14.1 Hz, 1H), 3.29 (ddd, J = 4.1, 10.7, 22.0 Hz, 1H), 3.15 (dt, J = 10.4, 14.0 Hz, 1H), 2.80 (hept, J = 6.9 Hz, 1H), 1.18 (dd, J = 1.4, 7.0 Hz, 6H). (Coupling constants are uncorrected)

^{13}C NMR (151 MHz, $CDCl_3$) δ 159.9 (d, J_{CP} = 2.3 Hz), 147.1, 137.4 (d, J_{CP} = 6.5 Hz), 136.6 (d, J_{CP} = 15.9 Hz), 129.7 (d, J_{CP} = 2.4 Hz), 129.0, 126.6, 122.2 (d, J_{CP} = 6.7 Hz), 115.5 (d, J_{CP} = 7.1 Hz), 113.1 (d, J_{CP} = 3.1 Hz), 55.6, 53.4 (dd, J_{CP} = 7.0, 126.4 Hz), 46.6 (d, J_{CP} = 135.9 Hz), 36.2 (d, J_{CP} = 2.8 Hz), 34.0, 24.3 (d, J_{CP} = 4.2 Hz).

^{31}P NMR (243 MHz, $CDCl_3$) δ 30.55.

IR (neat): 2960, 2833, 1582, 1566, 1472, 1439, 1414, 1326, 1283, 1241, 1227, 1181, 1168, 1159, 1090, 1060, 1028, 988, 859, 840, 821, 763, 679, 651, 563, 478 cm^{-1}.

$[\alpha]^{20}$ D: + 77.5° (c 4.40, $CHCl_3$)

HRMS (+p APCI) calcd. for $C_{20}H_{27}O_4P$ $[M + H]^+$ 363.1718, found 363.1725.

HPLC analysis: (Chiralcel ADH, 25 cm x 4.6 mm, 2.0% *i*-PrOH/hexanes, 1.0 mL/min, 280 nm) indicated 97% ee: t_R (minor enantiomer) = 11.9 min, *t* (major enantiomer) = 26.1 min.

Dimethyl (2-(4-isopropylphenyl)-1-(naphthalen-2-yl)ethyl)phosphonate (13d)

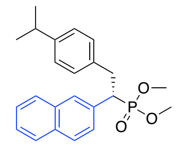

13d

The material was purified by flash chromatography (SiO_2; hexanes/EtOAc, 10:0 to 1:1 gradient; R_f = 0.40 in 1:1 hexanes/EtOAc), which afforded **13d** as a colorless oil (61 mg, 80%).

^{1}H NMR (600 MHz, CDCl$_3$) δ 7.78 (dt, J = 5.4, 14.8 Hz, 3H), 7.71 (s, 1H), 7.48 (d, J = 8.4 Hz, 1H), 7.46 – 7.42 (m, 2H), 6.98 (d, J = 8.0 Hz, 2H), 6.94 (d, J = 8.0 Hz, 2H), 3.69 (d, J_{HP} = 10.6 Hz, 3H), 3.55 – 3.46 (m, 2H), 3.44 (d, J_{HP} = 10.6 Hz, 3H), 3.30 (dt, J = 11.1, 14.4 Hz, 1H), 2.77 (hept, J = 6.9 Hz, 1H), 1.14 (dd, J = 3.3, 6.9 Hz, 6H). (Coupling constants are uncorrected)

^{13}C NMR (151 MHz, CDCl$_3$) δ 147.13, 136.49 (d, J_{CP} = 15.7 Hz), 133.69 (d, J_{CP} = 2.5 Hz), 133.40 (d, J_{CP} = 6.8 Hz), 132.95 (d, J_{CP} = 2.2 Hz), 129.02, 128.86 (d, J_{CP} = 8.8 Hz), 128.48 (d, J_{CP} = 2.1 Hz), 128.20, 127.96, 127.58 (d, J_{CP} = 5.3 Hz), 126.62, 126.36, 126.16, 53.43 (dd, J_{CP} = 7.0, 120.9 Hz), 46.68 (d, J_{CP} = 135.8 Hz), 36.07 (d, J_{CP} = 2.7 Hz), 33.92, 24.25.

^{31}P NMR (243 MHz, CDCl$_3$) δ 30.50.

IR (neat): 2955, 1600, 1508, 1461, 1363, 1245, 1184, 1053, 1030, 861, 759, 650, 575, 545, 503, 476, 456 cm^{-1}.

$[\alpha]^{20}$ D: + 97.7° (c 1.00, $CHCl_3$)

HRMS (+p APCl) calcd. for $C_{23}H_{27}O_3P$ $[M + H]^+$ 383.1771, found 383.1777

HPLC analysis: (Chiralcel ADH, 25 cm x 4.6 mm, 3.0% *i*-PrOH/hexanes, 1.0 mL/min, 230 nm) indicated 94% ee: t_R (minor enantiomer) = 25.4 min, t (major enantiomer) = 39.5 min.

Dimethyl (1-(3,4-dichlorophenyl)-2-(4-isopropylphenyl)ethyl)phosphonate (13e)

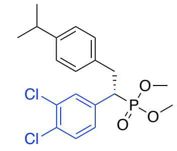

13e

The material was purified by flash chromatography (SiO_2; hexanes/EtOAc, 10:0 to 1:1 gradient; R_f = 0.35 in 1:1 hexanes/EtOAc), which afforded **13e** as a colorless oil (68 mg, 85%).

^{1}H NMR (600 MHz, CDCl$_3$) δ 7.33 (d, J = 8.5 Hz, 2H), 7.16 – 7.11 (m, 1H), 7.04 (d, J = 7.8 Hz, 2H), 6.90 (d, J = 7.8 Hz, 2H), 3.72 (d, J_{HP} = 10.6 Hz, 3H), 3.58 (d, J_{HP} = 10.6 Hz, 3H), 3.40 (ddd, J = 4.0, 9.3, 13.7 Hz, 1H), 3.27 (ddd, J = 4.0, 11.1, 22.5 Hz, 1H), 3.12 – 3.02 (m, 1H), 2.82 (hept, J = 6.9 Hz, 1H), 1.18 (d, J = 6.9 Hz, 6H). (Coupling constants are uncorrected)

^{13}C NMR (151 MHz, CDCl$_3$) δ 136.37 (d, J_{CP} = 6.2 Hz), 135.70 (d, J_{CP} = 15.7 Hz), 132.80 (d, J_{CP} = 2.7 Hz), 131.68 (d, J_{CP} = 3.8 Hz), 131.60 (d, J_{CP} = 7.2 Hz), 130.71 (d, J_{CP} = 2.5 Hz), 129.04 (d, J_{CP} = 6.8 Hz), 128.94, 126.81, 53.54 (dd, J_{CP} = 7.0, 84.3 Hz), 45.81 (d, J_{CP} = 137.1 Hz), 35.93 (d, J_{CP} = 2.8 Hz), 33.98, 24.27 (d, J_{CP} = 2.4 Hz).

^{31}P NMR (243 MHz, CDCl$_3$) δ 29.26.

IR (neat): 2956, 1514, 1468, 1398, 1239, 1239, 1184, 1133, 1029, 894, 823, 761, 731, 704, 678, 596, 565, 506, 494, 478, 470, 459, 441, 422, 411, 406 cm^{-1}.

[α]^{20} D: + 145.1° (c 1.00, CHCl$_3$)

HRMS (+p APCl) calcd. for $C_{19}H_{23}Cl_2O_3P$ $[M + H]^+$ 401.0835, found 401.0842.

HPLC analysis: (Chiralcel ADH, 25 cm x 4.6 mm, 4.0% *i*-PrOH/hexanes, 1.0 mL/min, 230 nm) indicated 98% ee: t_R (minor enantiomer) = 13.5 min, t (major enantiomer) = 16.4 min.

Bis(2,2,2-trifluoroethyl) (*S*)-(1-(4-bromophenyl)-2-(4-isopropylphenyl)ethyl)phosphonate (15)

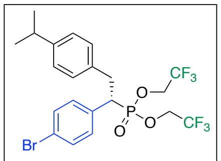

15

The material was purified by flash chromatography (SiO$_2$; hexanes/EtOAc, 10:0 to 1:1 gradient; R_f = 0.55 in 1:1 hexanes/EtOAc), which afforded **15** as a colorless oil (92 mg, 84%). **m.p.** 75-77 °C.

^{1}H NMR (400 MHz, CDCl$_3$) δ 7.47 – 7.42 (m, 2H), 7.20 – 7.14 (m, 2H), 7.09 – 7.04 (m, 2H), 6.97 – 6.92 (m, 2H), 4.34 – 4.15 (m, 2H), 4.08 (ddq, J = 8.1, 9.3, 12.2 Hz, 1H), 3.89 – 3.76 (m,

1H), 3.52 – 3.34 (m, 2H), 3.23 – 3.09 (m, 1H), 2.82 (hept, $J = 7.0$ Hz, 1H), 1.19 (d, $J = 7.0$ Hz, 6H). (Coupling constants are uncorrected)

^{13}C NMR (151 MHz, CDCl$_3$) δ 147.9, 135.0 (d, $J_{CP} = 15.3$ Hz), 133.2 (d, $J_{CP} = 6.7$ Hz), 132.3 (d, $J_{CP} = 2.7$ Hz), 131.3 (d, $J_{CP} = 7.3$ Hz), 129.0, 123.7 (qd, $J_{CPF} = 4.2$, 278.24 Hz), 123.6 (qd, $J_{CPF} = 4.2$, 278.24 Hz), 126.9, 63.0 (qd, $J_{CPF} = 6.5$, 37.8 Hz), 62.3 (qd, J $J_{CPF} = 6.5$, 37.8 Hz), 46.3 (d, $J_{CP} = 136.7$ Hz), 35.6 (d, $J_{CP} = 2.6$ Hz), 34.0, 24.3 (d, $J_{CP} = 3.1$ Hz).

^{19}F NMR (376 MHz, CDCl$_3$) δ -75.23 (t, $J = 8.1$ Hz), -75.51 (t, $J = 8.0$ Hz). (Coupling constants are uncorrected)

^{31}P NMR (162 MHz, CDCl$_3$) δ 30.43.

IR (neat): 2963, 1489, 1456, 1418, 1290, 1251, 1167, 1102, 1070, 1012, 962, 870, 835, 710, 652, 540, 481 cm-^{1}.

[α]^{20} D: + 82.6° (c 0.50, CHCl$_3$)

HRMS (+p APCl) calcd. for $C_{19}H_{23}Cl_2O_3P$ 401.0835, found 401.0842.

HPLC analysis: (Chiralcel ODH, 25 cm x 4.6 mm, 1.0% *i*-PrOH/hexanes, 1.0 mL/min, 230 nm) indicated 99% ee: t_R (minor enantiomer) = 11.2 min, *t* (major enantiomer) = 15.8 min.

The crystal structure information for compound (**15**) is located in the crystallography section.

5. *References.*

1. Davies, H. M. L.; Bruzinski, P. R.; Lake, D. H.; Kong, N.; Fall, M. J., Asymmetric Cyclopropanations by Rhodium(II) N-(Arylsulfonyl)prolinate Catalyzed Decomposition of Vinyldiazomethanes in the Presence of Alkenes. Practical Enantioselective Synthesis of the Four Stereoisomers of 2-Phenylcyclopropan-1-amino Acid. *J. Am. Chem. Soc.* **1996,** *118* (29), 6897-6907.
2. Callot, H. J.; Metz, F., Rhodium(II)2,4,6-triarylbenzoates : improved catalysts for the syn cyclopropanation of z-olefins. *Tetrahedron* **1985,** *41* (20), 4495-4501.
3. Reddy, R. P.; Lee, G. H.; Davies, H. M. L., Dirhodium Tetracarboxylate Derived from Adamantylglycine as a Chiral Catalyst for Carbenoid Reactions. *Org. Lett.* **2006,** *8* (16), 3437-3440.
4. Fu, J.; Ren, Z.; Bacsa, J.; Musaev, D. G.; Davies, H. M. L., Desymmetrization of cyclohexanes by site- and stereoselective C–H functionalization. *Nature* **2018,** *564* (7736), 395-399.
5. Reddy, R. P.; Davies, H. M. L., Dirhodium Tetracarboxylates Derived from Adamantylglycine as Chiral Catalysts for Enantioselective C−H Aminations. *Org. Lett.* **2006,** *8* (22), 5013-5016.
6. Ghanem, A.; Gardiner, M. G.; Williamson, R. M.; Müller, P., First X-ray Structure of a N-Naphthaloyl-Tethered Chiral Dirhodium(II) Complex: Structural Basis for Tether Substitution Improving Asymmetric Control in Olefin Cyclopropanation. *Chem. Eur. J.* **2010,** *16* (11), 3291-3295.
7. Qin, C.; Boyarskikh, V.; Hansen, J. H.; Hardcastle, K. I.; Musaev, D. G.; Davies, H. M. L., D2-Symmetric Dirhodium Catalyst Derived from a 1,2,2-Triarylcyclopropanecarboxylate Ligand: Design, Synthesis and Application. *J. Am. Chem. Soc.* **2011,** *133* (47), 19198-19204.
8. Garlets, Z. J.; Boni, Y. T.; Sharland, J. C.; Kirby, R. P.; Fu, J.; Bacsa, J.; Davies, H. M. L., Design, Synthesis, and Evaluation of Extended C4–Symmetric Dirhodium Tetracarboxylate Catalysts. *ACS Catal.* **2022,** *12* (17), 10841-10848.
9. Han, B.; Ren, C.; Jiang, M.; Wu, L., Titanium-Catalyzed Exhaustive Reduction of Oxo-Chemicals. *Angew. Chem. Int. Ed.* **2022,** *61* (46), e202209232.
10. Yanagisawa, A.; Nezu, T.; Mohri, S.-i., Brønsted Acid-Promoted Hydrocyanation of Arylalkenes. *Org. Lett.* **2009,** *11* (22), 5286-5289.
11. Rono, L. J.; Yayla, H. G.; Wang, D. Y.; Armstrong, M. F.; Knowles, R. R., Enantioselective Photoredox Catalysis Enabled by Proton-Coupled Electron Transfer: Development of an Asymmetric Aza-Pinacol Cyclization. *J. Am. Chem. Soc.* **2013,** *135* (47), 17735-17738.
12. Qin, C.; Davies, H. M. L., Role of Sterically Demanding Chiral Dirhodium Catalysts in Site-Selective C–H Functionalization of Activated Primary C–H Bonds. *J. Am. Chem. Soc.* **2014,** *136* (27), 9792-9796.
13. Jaladi, A. K.; Choi, H. S.; An, D. K., Catalyst-free and solvent-free hydroboration of alkynes. *New J. Chem.* **2020,** *44* (32), 13626-13632.
14. Burke, C. P.; Shi, Y., Enantioselective Epoxidation of Nonconjugated cis-Olefins by Chiral Dioxirane. *Org. Lett.* **2009,** *11* (22), 5150-5153.

15. Feng, B.; Yang, Y.; You, J., A methylation platform of unconventional inert aryl electrophiles: trimethylboroxine as a universal methylating reagent. *Chem. Sci.* **2020,** *11* (23), 6031-6035.
16. Adeniji, A.; Uddin, M. J.; Zang, T.; Tamae, D.; Wangtrakuldee, P.; Marnett, L. J.; Penning, T. M., Discovery of (R)-2-(6-Methoxynaphthalen-2-yl)butanoic Acid as a Potent and Selective Aldo-keto Reductase 1C3 Inhibitor. *J. Med. Chem.* **2016,** *59* (16), 7431-7444.
17. Lovrek, M.; Takac, M. J.; Zorc, B.; Boneschans, B., Gemfibrozil ester and amide derivatives--synthesis, spectroscopic characterisation and QSPR. *Die Pharmazie* **2000,** *55* (11), 811-816.
18. Basu, S.; Barawkar, D. A.; Thorat, S.; Shejul, Y. D.; Patel, M.; Naykodi, M.; Jain, V.; Salve, Y.; Prasad, V.; Chaudhary, S.; Ghosh, I.; Bhat, G.; Quraishi, A.; Patil, H.; Ansari, S.; Menon, S.; Unadkat, V.; Thakare, R.; Seervi, M. S.; Meru, A. V.; De, S.; Bhamidipati, R. K.; Rouduri, S. R.; Palle, V. P.; Chug, A.; Mookhtiar, K. A., Design, Synthesis of Novel, Potent, Selective, Orally Bioavailable Adenosine A2A Receptor Antagonists and Their Biological Evaluation. *J. Med. Chem.* **2017,** *60* (2), 681-694.
19. Luan, Y.-Y.; Gou, X.-Y.; Shi, W.-Y.; Liu, H.-C.; Chen, X.; Liang, Y.-M., Three-Component Ruthenium-Catalyzed meta-C–H Alkylation of Phenol Derivatives. *Org. Lett.* **2022,** *24* (5), 1136-1140.
20. Haydl, A. M.; Hartwig, J. F., Palladium-Catalyzed Methylation of Aryl, Heteroaryl, and Vinyl Boronate Esters. *Org. Lett.* **2019,** *21* (5), 1337-1341.
21. Dhameja, M.; Pandey, J., Bestmann–Ohira Reagent: A Convenient and Promising Reagent in the Chemical World. *Asian Journal of Org. Chem.* **2018,** *7* (8), 1502-1523.
22. Ohira, S., Methanolysis of Dimethyl (1-Diazo-2-oxopropyl) Phosphonate: Generation of Dimethyl (Diazomethyl) Phosphonate and Reaction with Carbonyl Compounds. *Synth. Commun.* **1989,** *19* (3-4), 561-564.
23. Müller, S.; Liepold, B.; Roth, G. J.; Bestmann, H. J., An Improved One-pot Procedure for the Synthesis of Alkynes from Aldehydes. *Synlett* **1996,** *1996* (06), 521-522.
24. Zhou, Y.; Zhang, Y.; Wang, J., Geminal difunctionalization of α-diazo arylmethylphosphonates: synthesis of fluorinated phosphonates. *Org. Biomol. Chem.* **2016,** *14* (44), 10444-10453.
25. Ye, F.; Wang, C.; Zhang, Y.; Wang, J., Synthesis of Aryldiazoacetates through Palladium(0)-Catalyzed Deacylative Cross-Coupling of Aryl Iodides with Acyldiazoacetates. *Angew. Chem. Int. Ed.* **2014,** *53* (43), 11625-11628.
26. Wu, C.; Ye, F.; Wu, G.; Xu, S.; Deng, G.; Zhang, Y.; Wang, J., Synthesis of Allenylphosphonates through Cu(I)-Catalyzed Coupling- of Terminal Alkynes with Diazophosphonates. *Synthesis* **2016,** *48* (05), 751-760.
27. Tomioka, H.; Hirai, K.; Tanimoto, Y., Chemistry and kinetics of α- and β-naphthyl(phosphonyl)carbenes. Effects of positions on neighbouring phosphonate participation. *J. Chem. Soc., PERKIN TRANS. 2* **1994,** (3), 633-641.
28. Al-Riyami, L.; Pineda, M. A.; Rzepecka, J.; Huggan, J. K.; Khalaf, A. I.; Suckling, C. J.; Scott, F. J.; Rodgers, D. T.; Harnett, M. M.; Harnett, W., Designing Anti-inflammatory Drugs from Parasitic Worms: A Synthetic Small Molecule Analogue of the Acanthocheilonema viteae Product ES-62 Prevents Development of Collagen-Induced Arthritis. *J. Med. Chem.* **2013,** *56* (24), 9982-10002.

29. Knoll, S.; Streb, C., Initial Steps of the Acid-Catalyzed Polyoxometalate-Functionalization with Phosphonic Acid Esters. *Inorg. Chem.* **2023,** *62* (3), 1218-1225.

8. ^{1}H NMR, ^{13}C NMR, ^{19}F NMR, and ^{31}P NMR

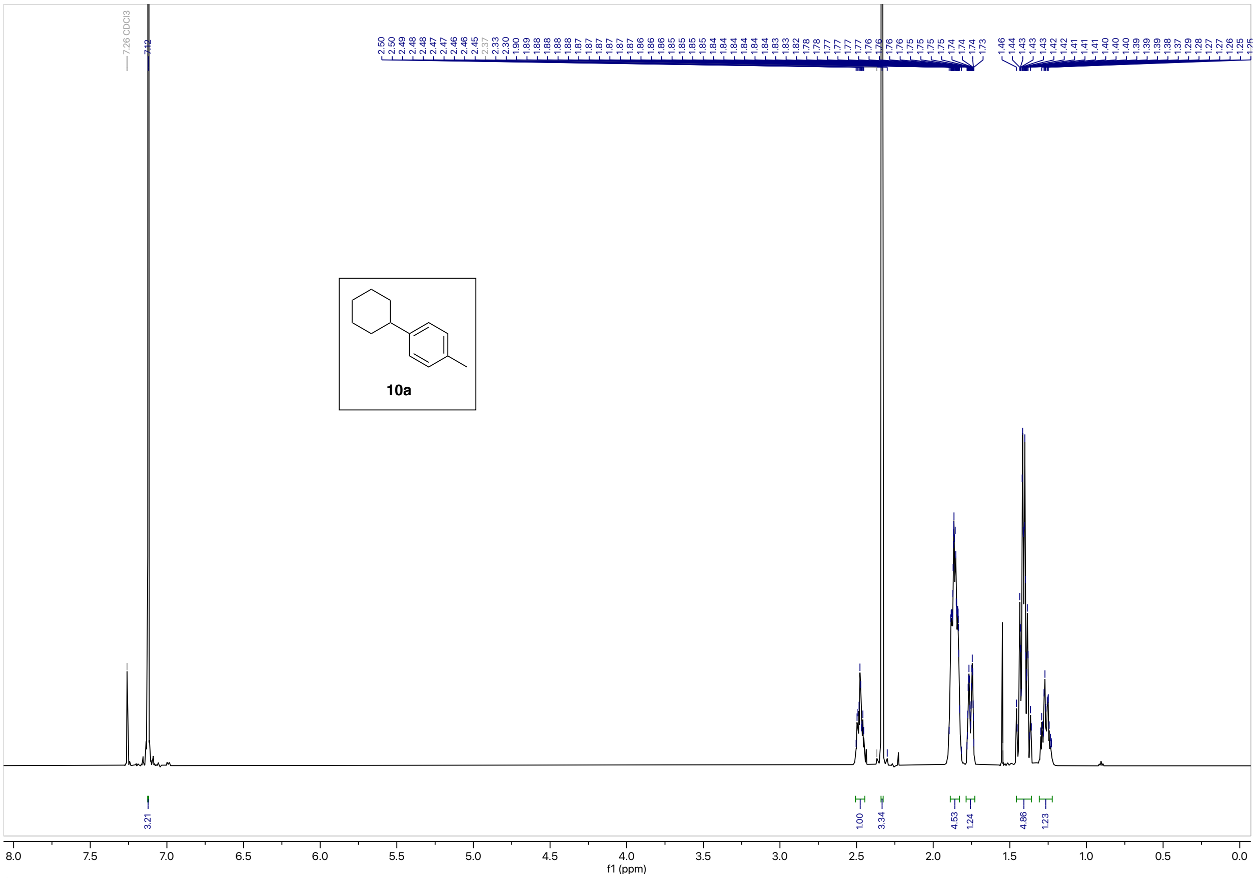

^{1}H NMR spectrum (600 MHz, Chloroform-*d*) (s, 7.26 ppm) of **10a**.

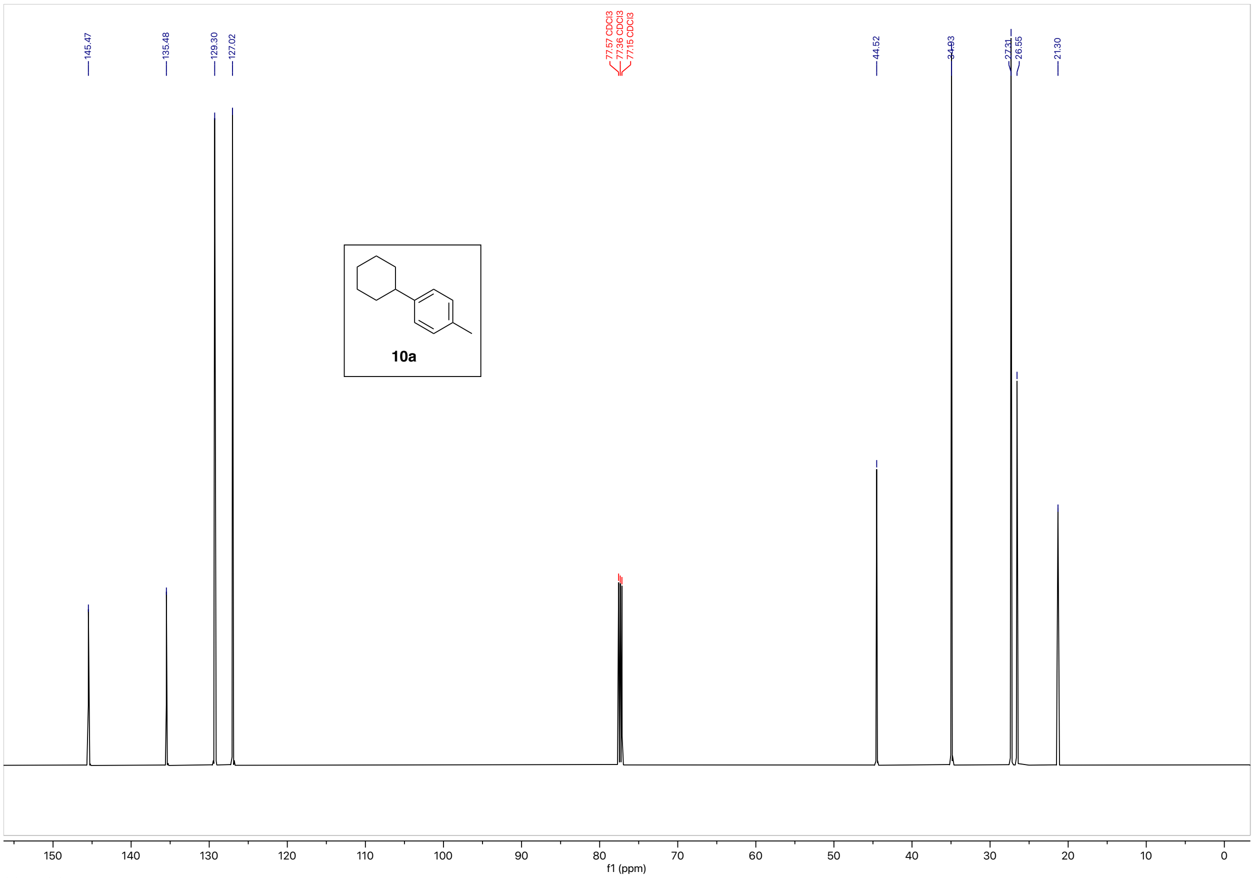

$^{13}C^{11}$ NMR spectrum (151 MHz, Chloroform-*d*) (t, 77.36 ppm) of **10a**.

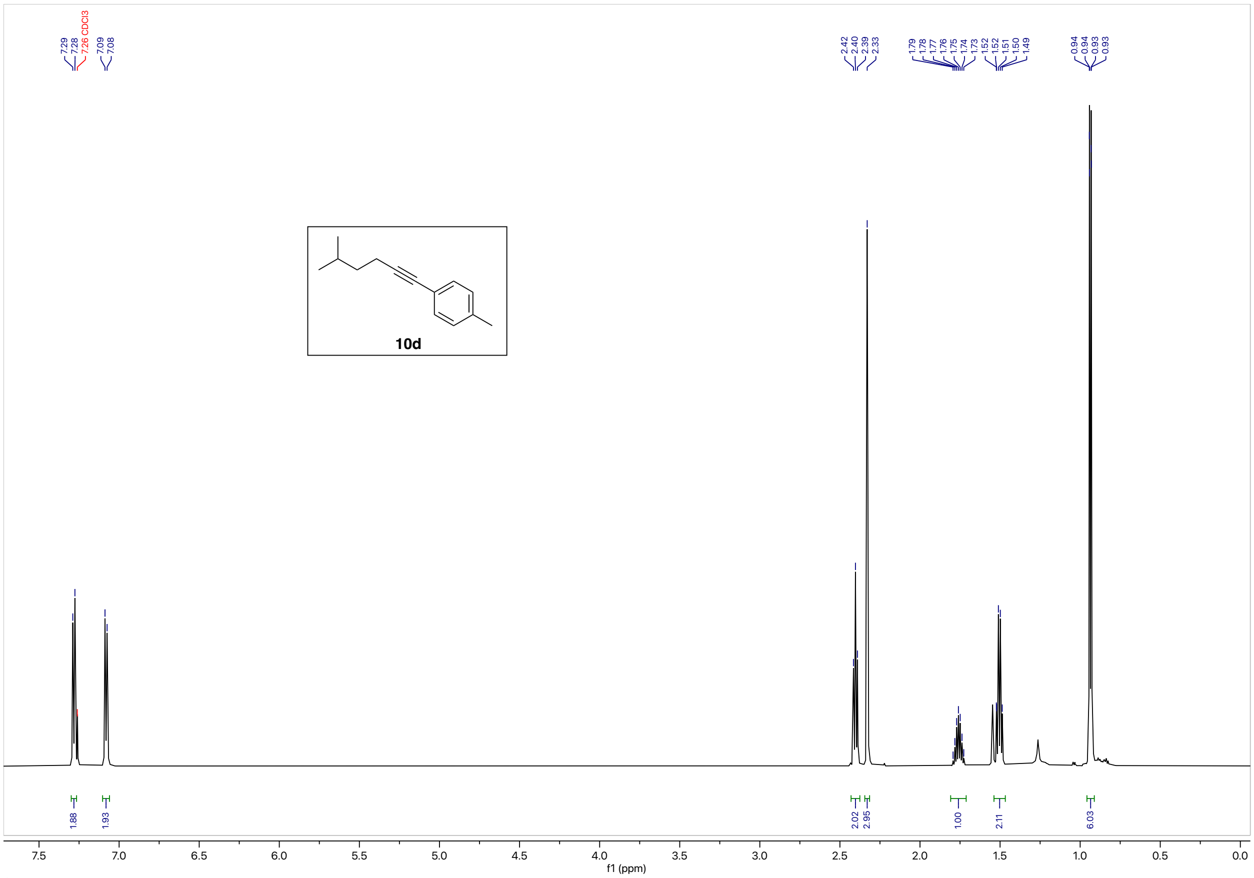

^{1}H NMR spectrum (600 MHz, Chloroform-*d*) (s, 7.26 ppm) of **10d**.

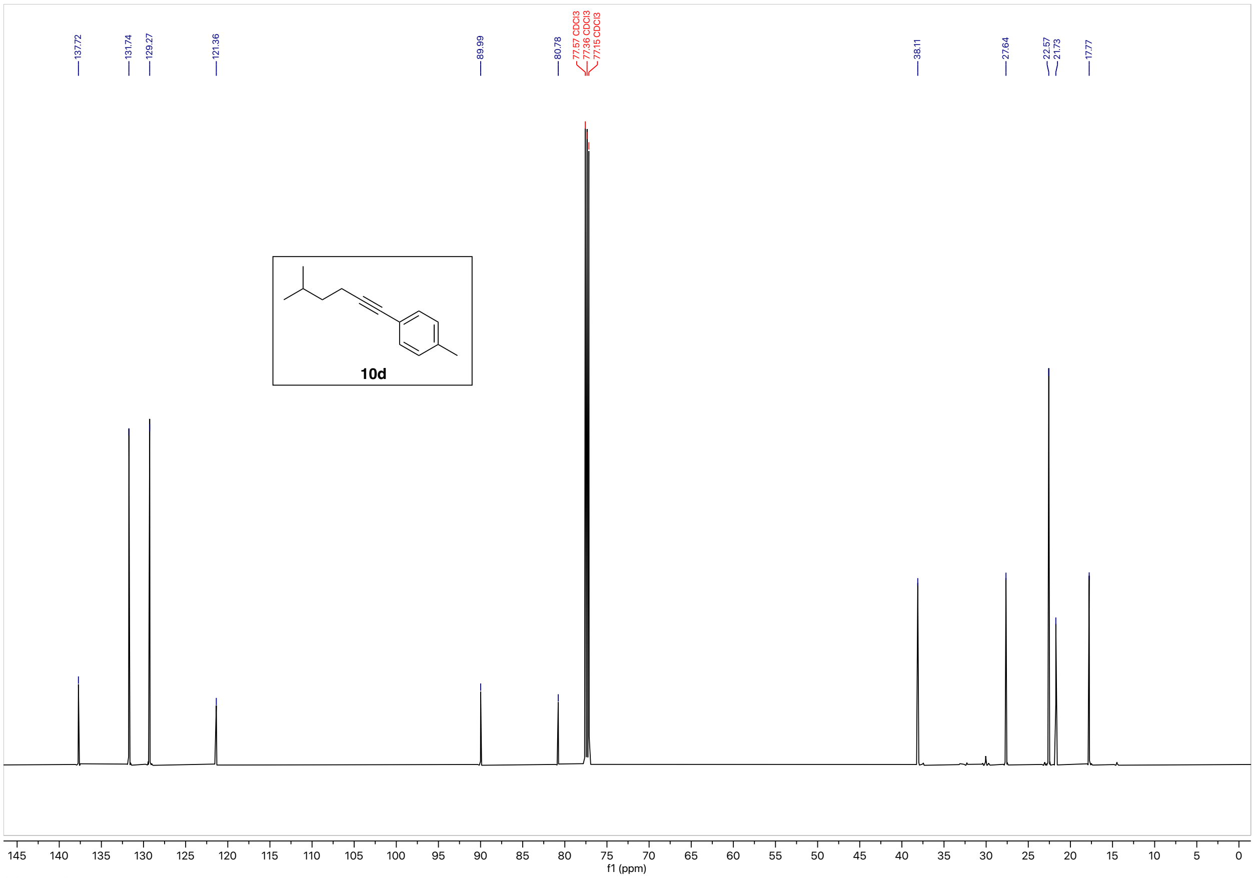

$^{13}C\{^{1}H\}$ NMR spectrum (151 MHz, Chloroform-*d*) (t, 77.36 ppm) of **10d**.

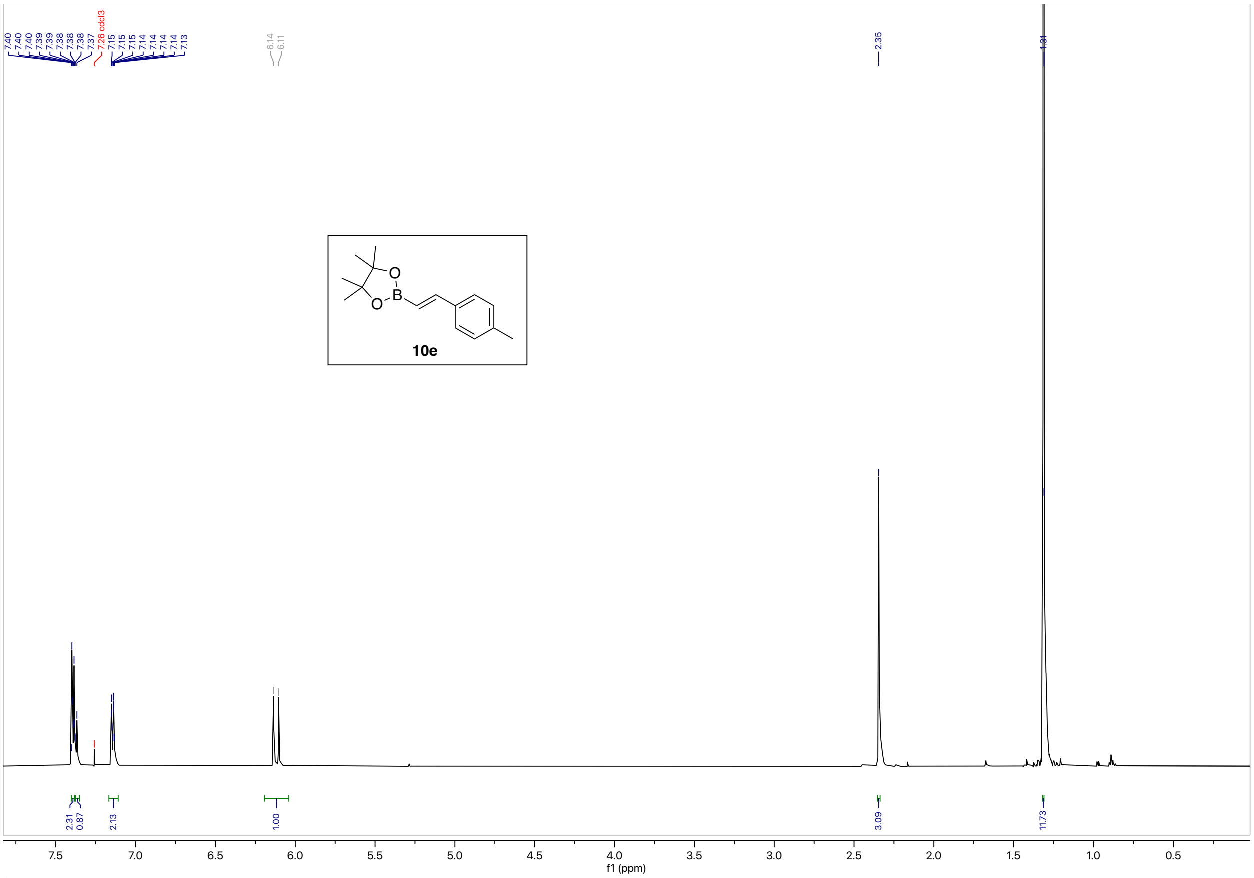

^{1}H NMR spectrum (600 MHz, Chloroform-*d*) (s, 7.26 ppm) of **10e**.

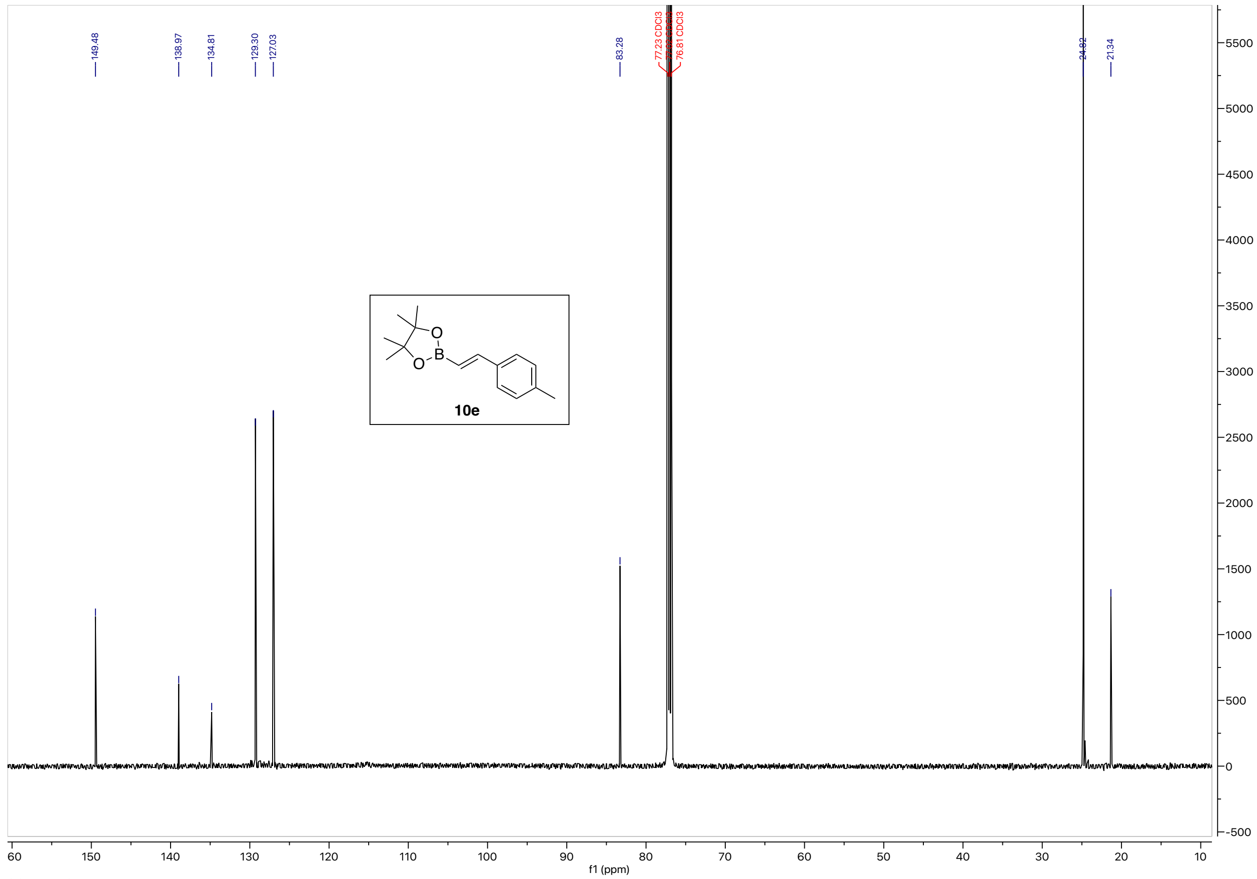

$^{13}C\{^{1}H\}$ NMR spectrum (151 MHz, Chloroform-*d*) (t, 77.36 ppm) of **7e**.

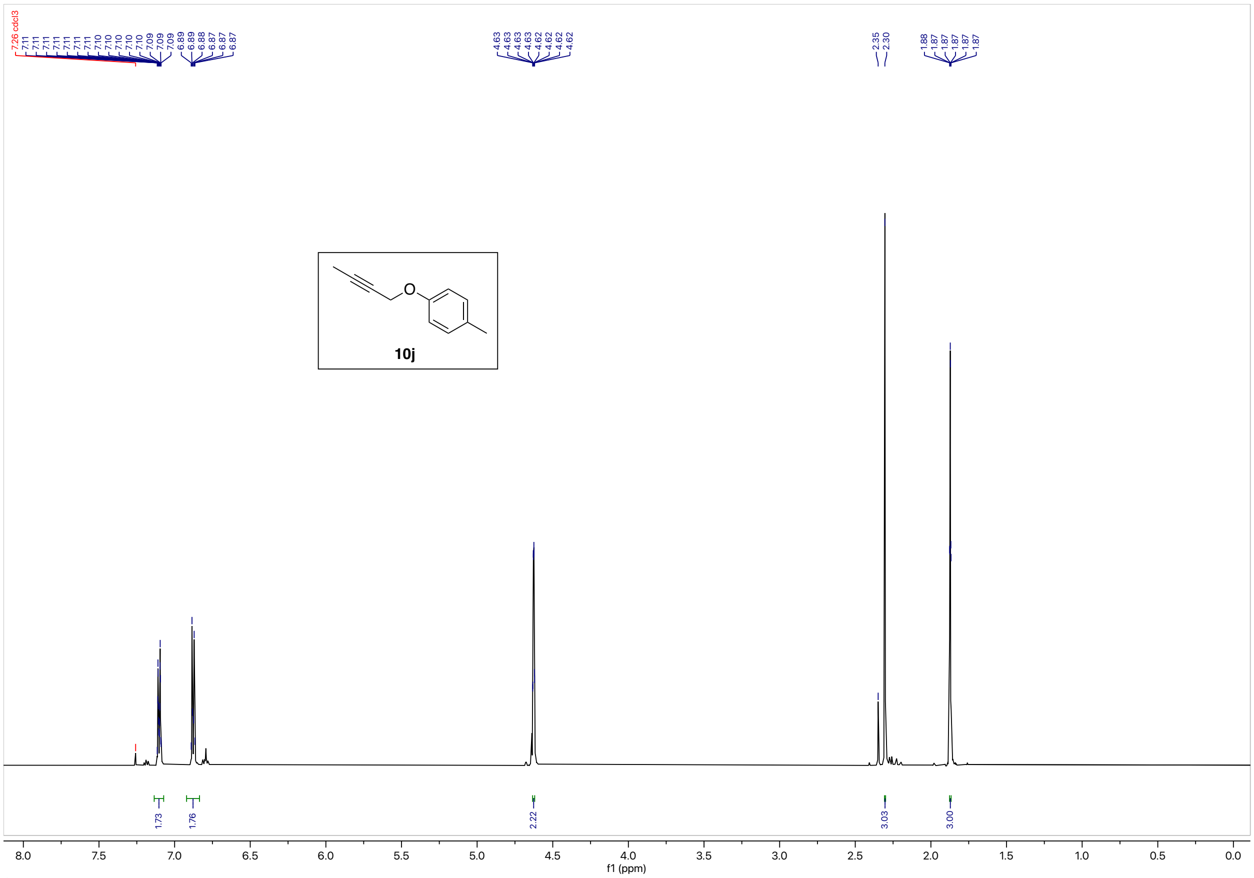

^{1}H NMR spectrum (600 MHz, Chloroform-*d*) (s, 7.26 ppm) of **10j**.

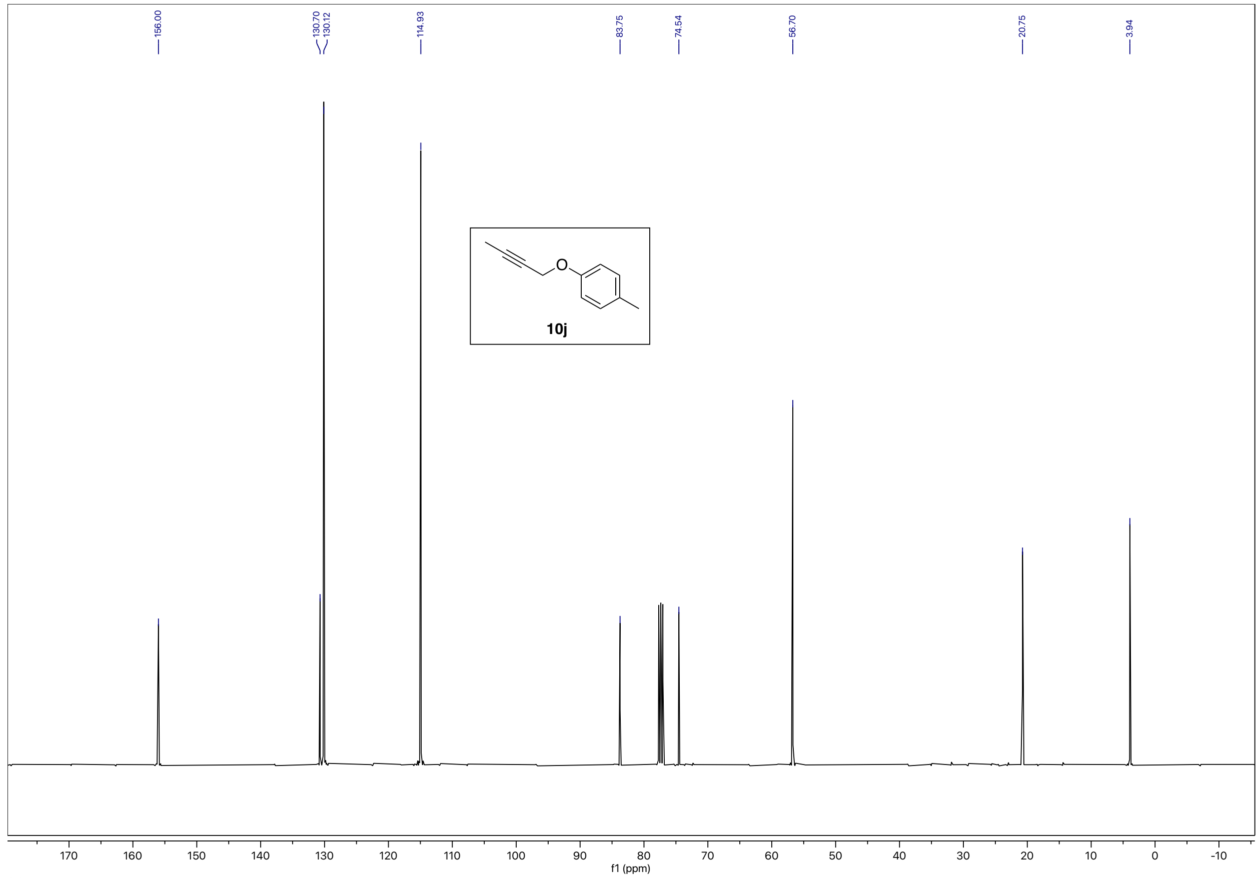

^{13}C NMR spectrum (101 MHz, Chloroform-*d*) (t, 77.36 ppm) of **10j**.

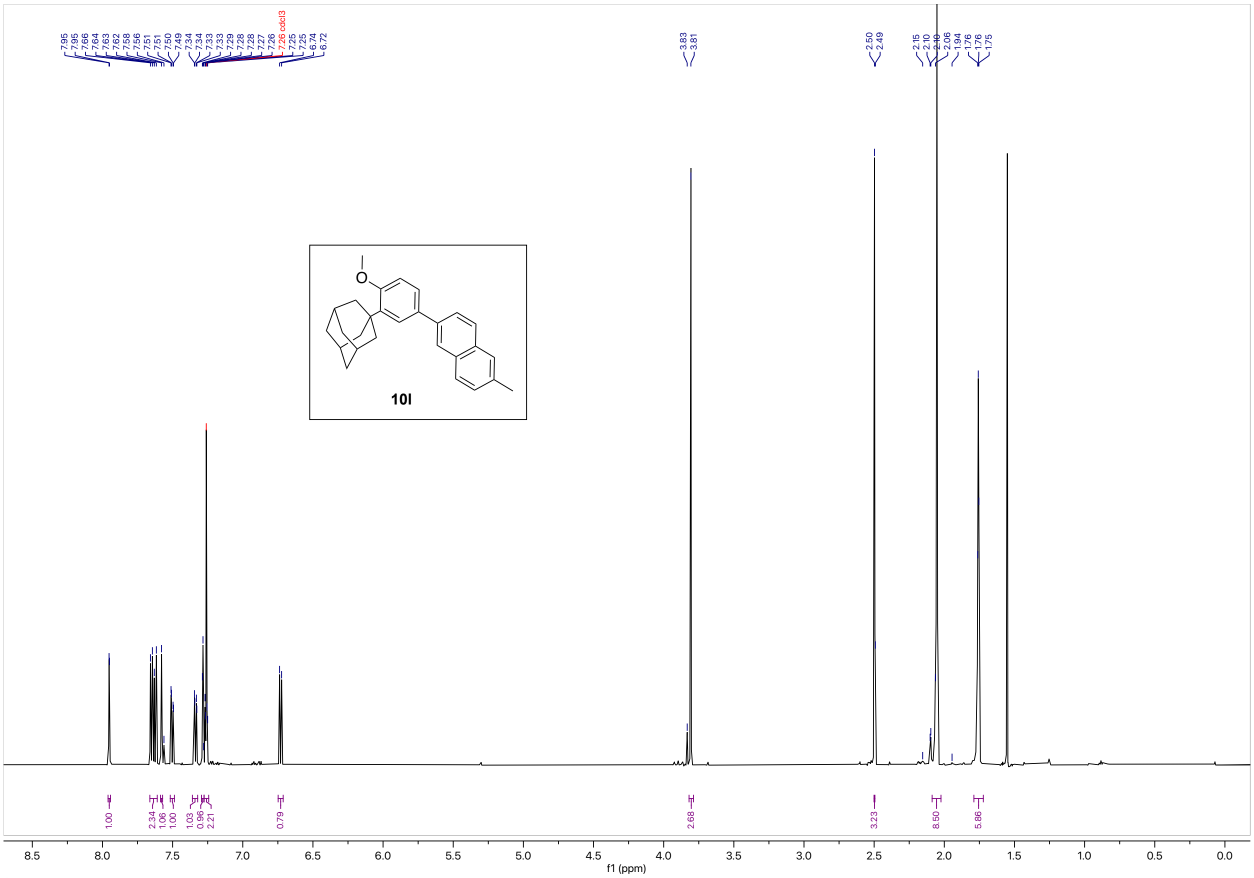

^{1}H NMR spectrum (600 MHz, Chloroform-*d*) (s, 7.26 ppm) of **10l**.

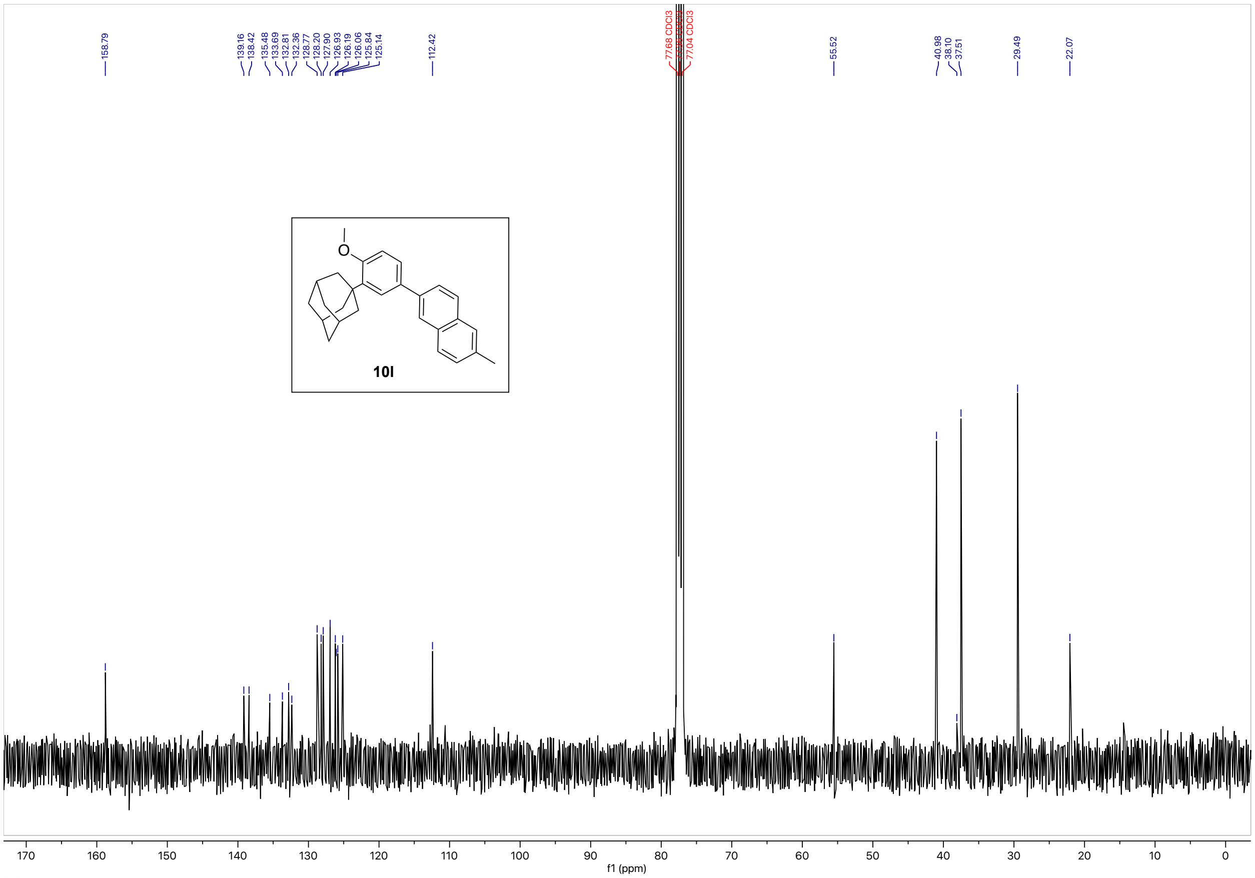

^{13}C NMR spectrum (101 MHz, Chloroform-*d*) (t, 77.36 ppm) of **10l**.

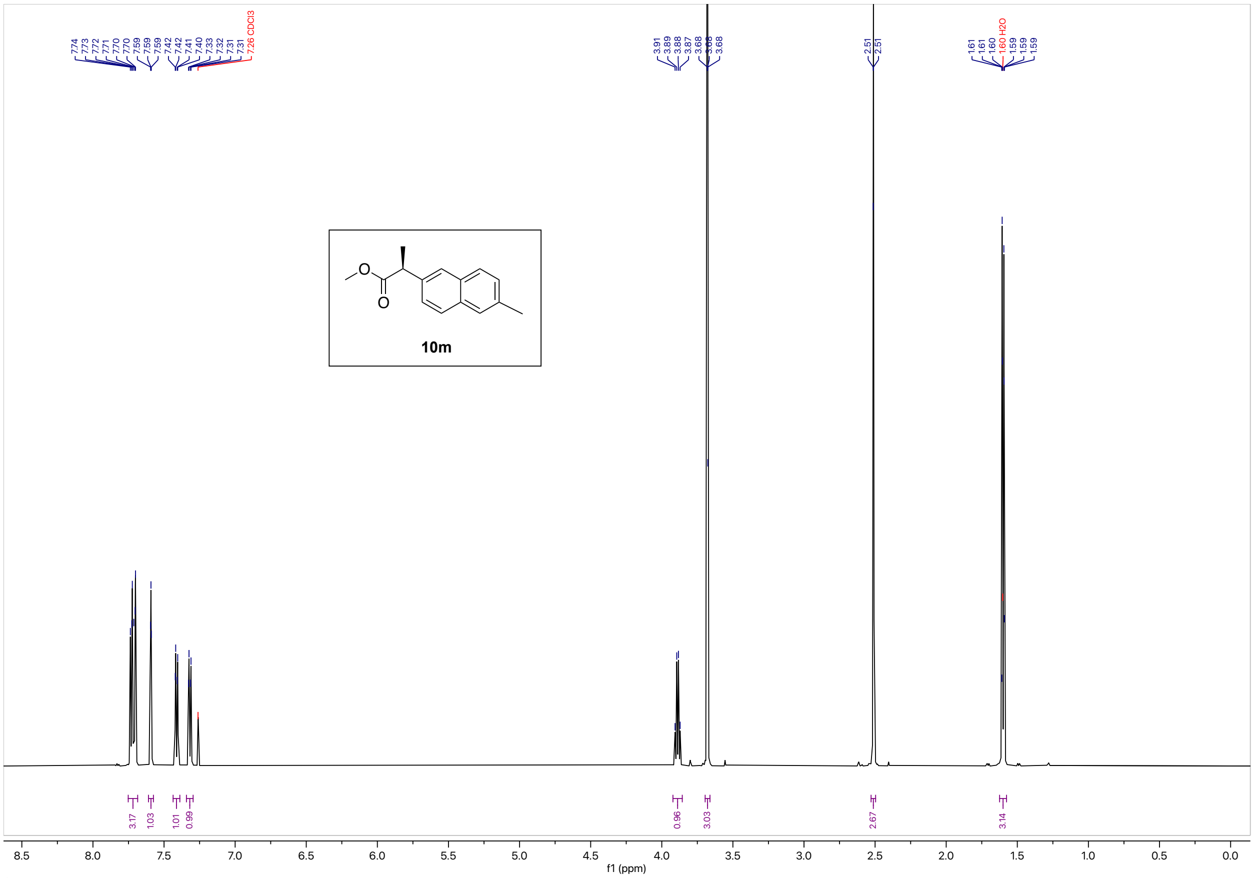

^{1}H NMR spectrum (600 MHz, Chloroform-*d*) (s, 7.26 ppm) of **10m**.

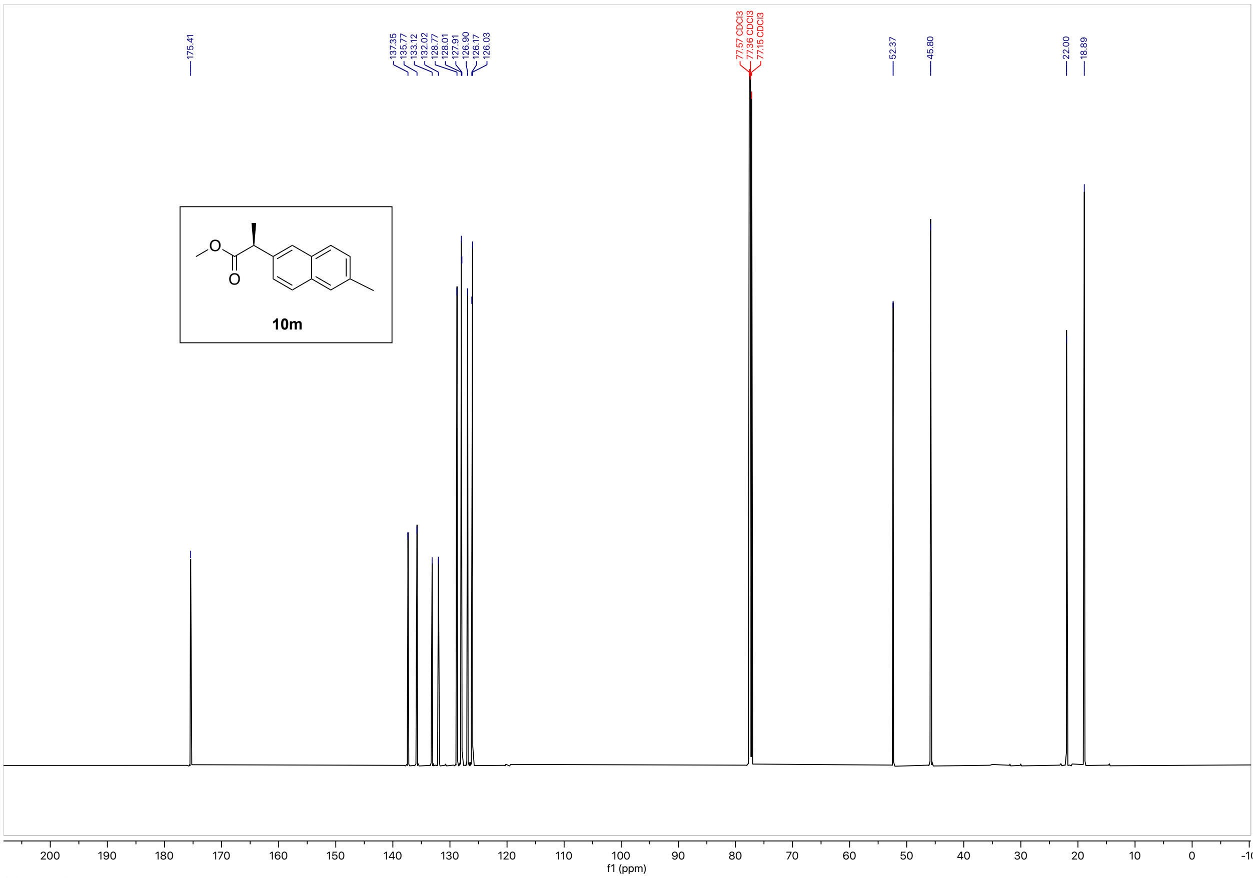

$^{13}C\{^{1}H\}$ NMR spectrum (151 MHz, Chloroform-*d*) (t, 77.36 ppm) of **10m**.

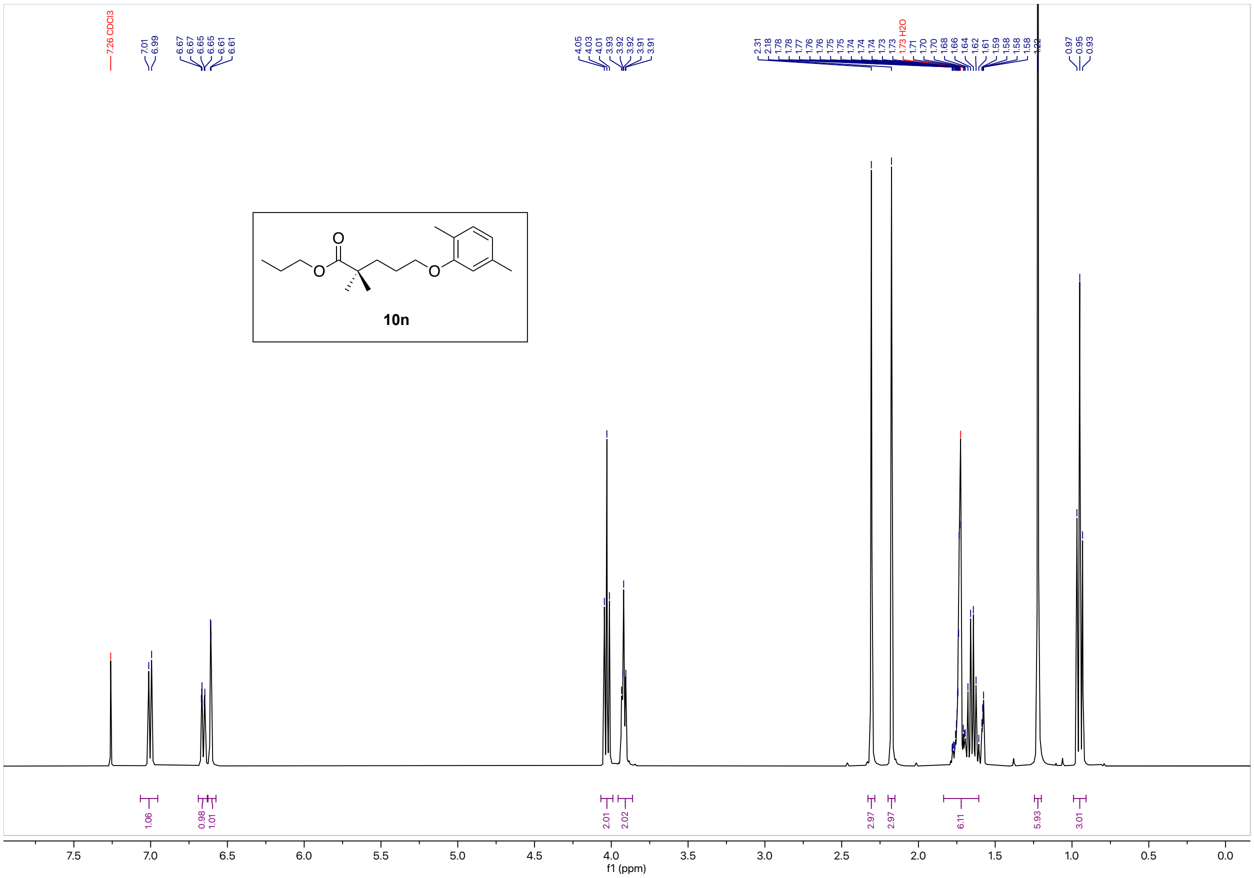

^{1}H NMR spectrum (600 MHz, Chloroform-*d*) (s, 7.26 ppm) of **10n**.

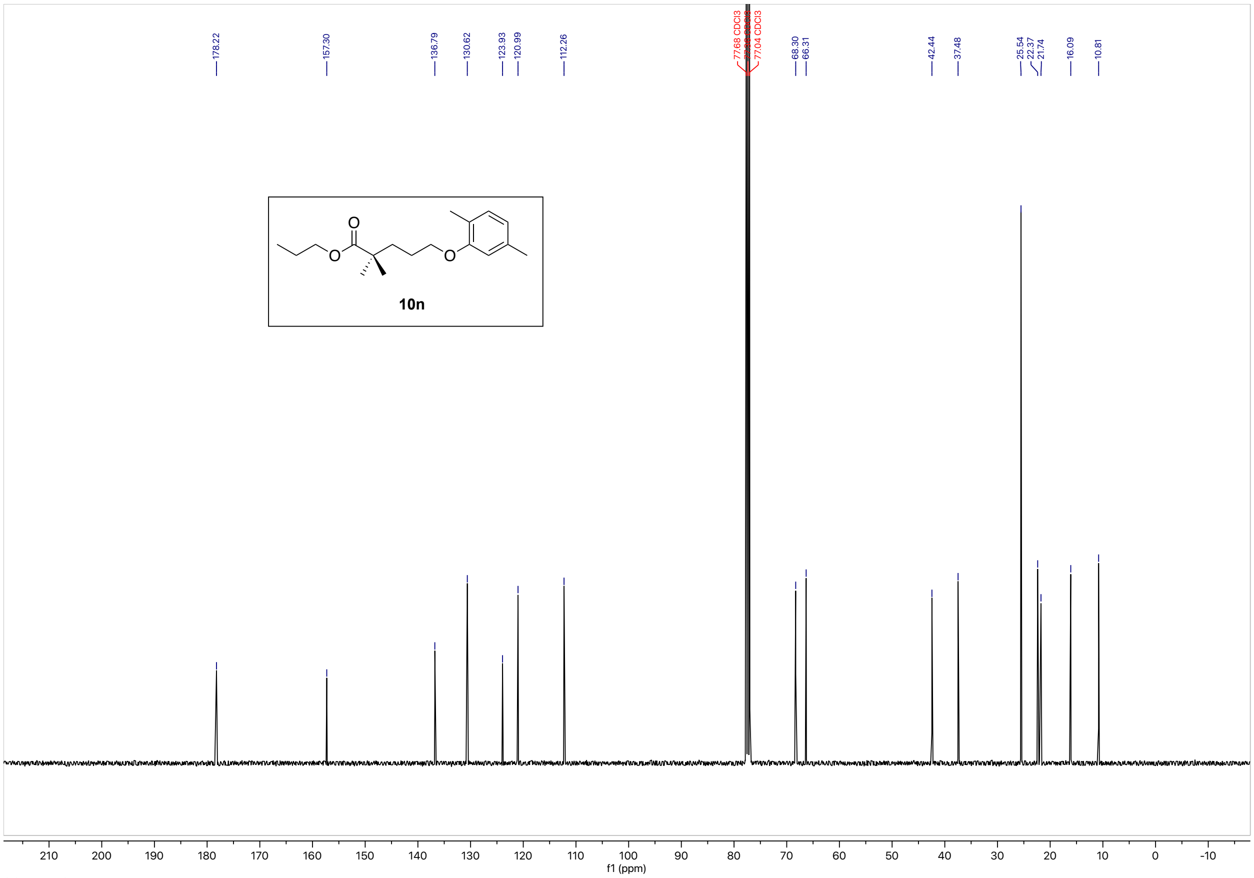

$^{13}C\{^1H\}$ NMR spectrum (151 MHz, Chloroform-*d*) (t, 77.36 ppm) of **10n**.

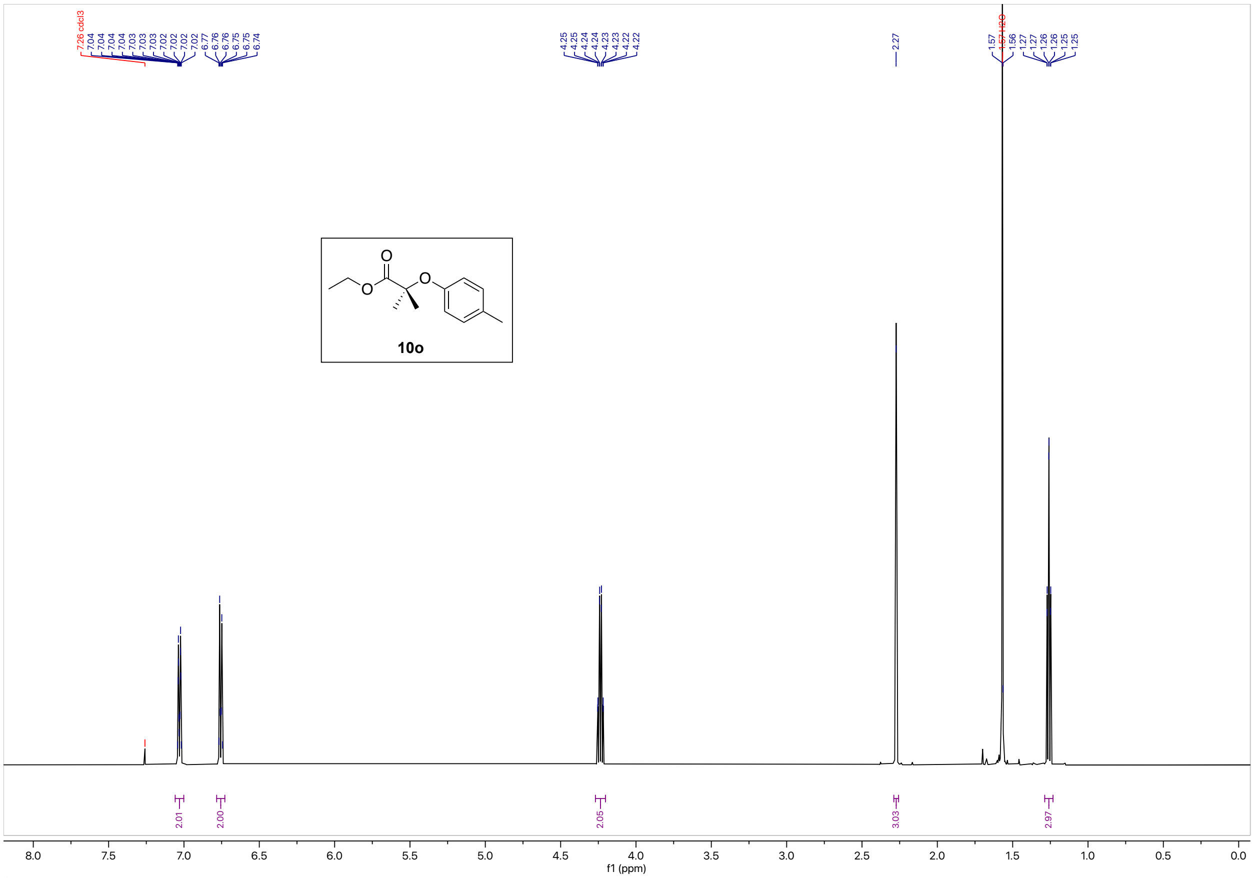

^{1}H NMR spectrum (600 MHz, Chloroform-*d*) (s, 7.26 ppm) of **10o**.

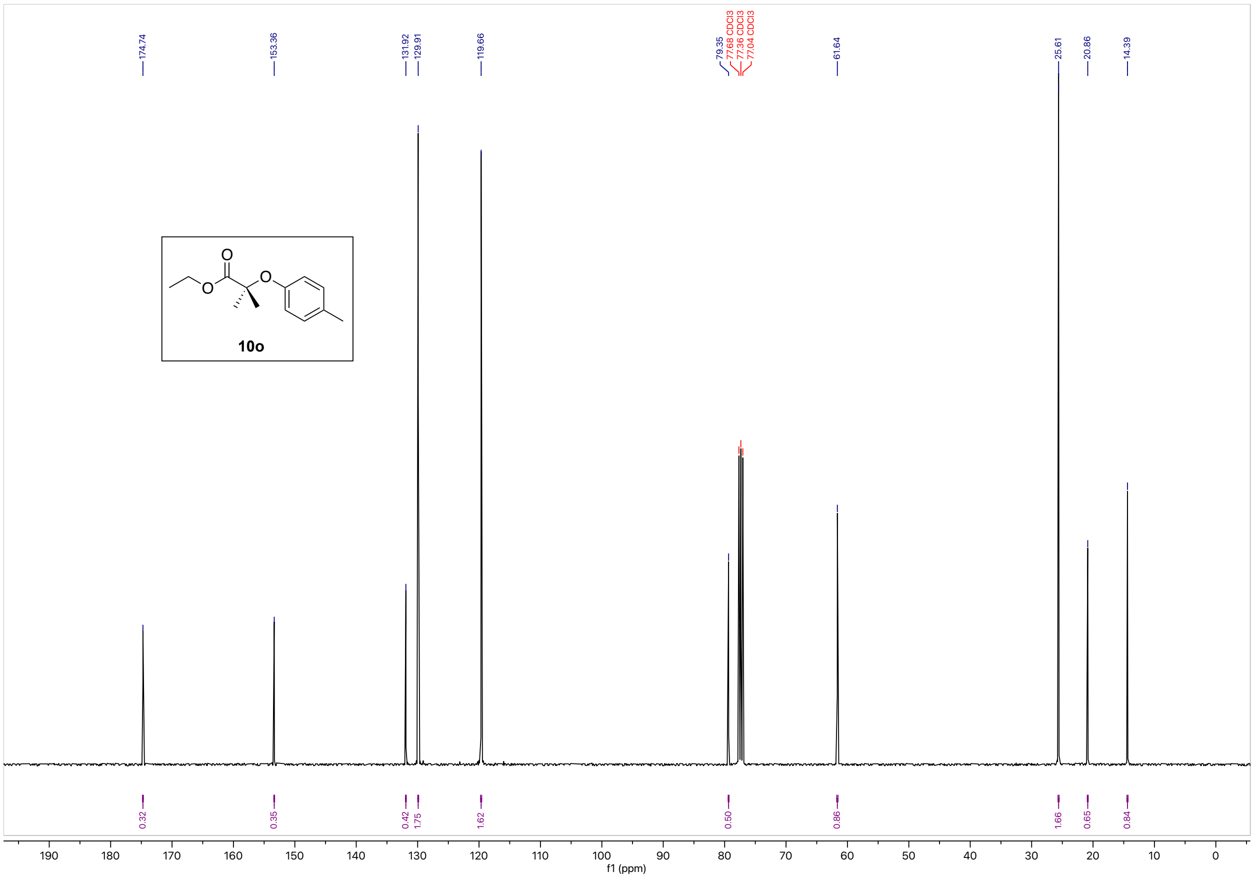

$^{13}C\{^{1}H\}$ NMR spectrum (101 MHz, Chloroform-*d*) (t, 77.36 ppm) of **10o**.

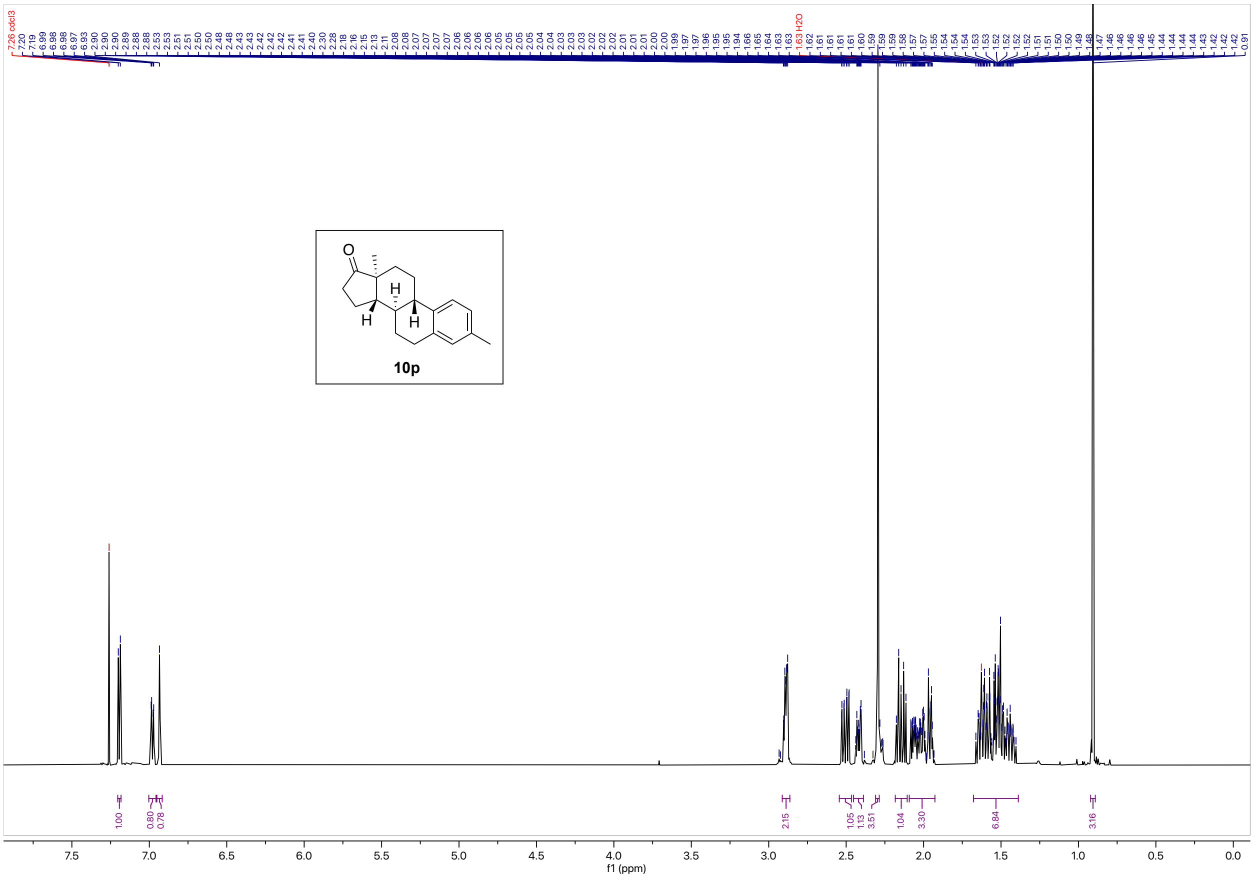

^{1}H NMR spectrum (600 MHz, Chloroform-*d*) (s, 7.26 ppm) of **10p**.

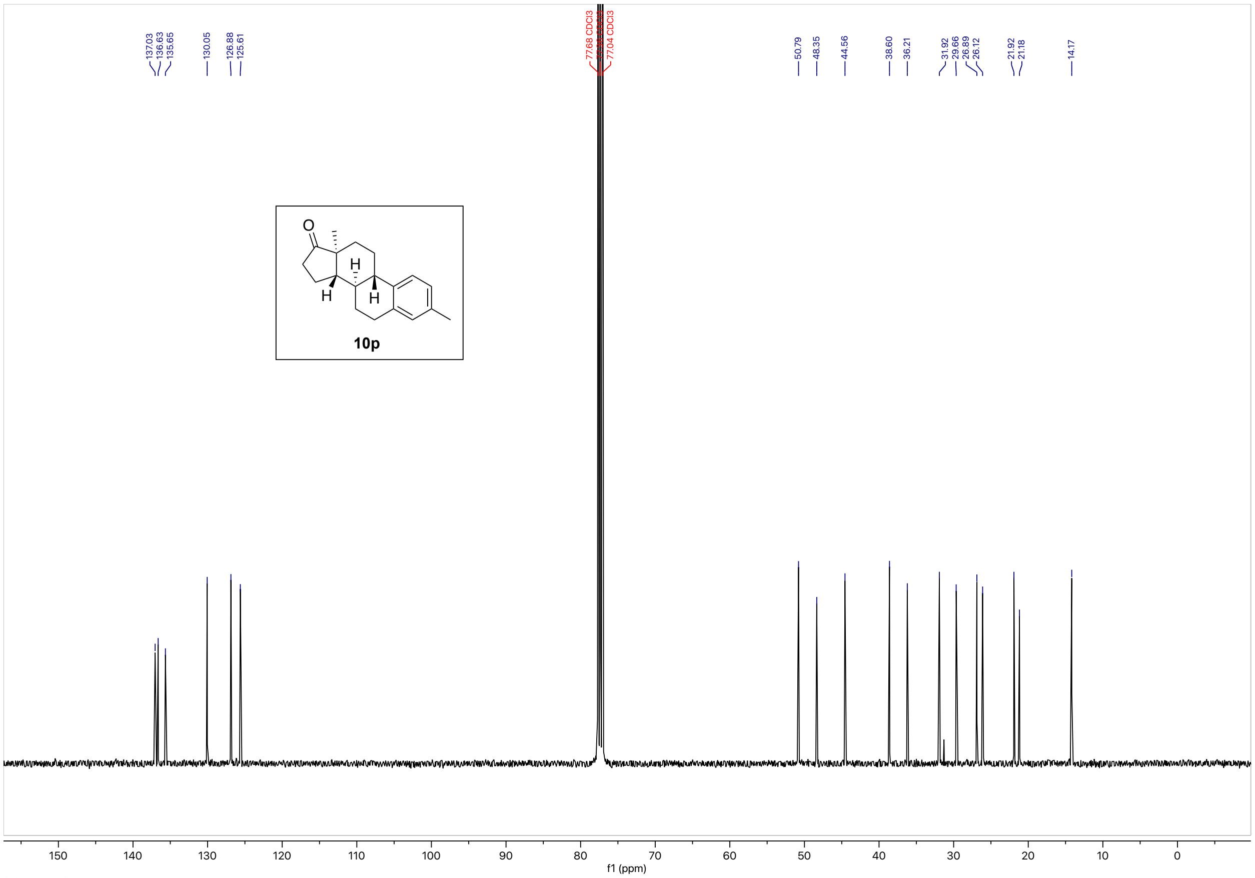

$^{13}C\{^{1}H\}$ NMR spectrum (101 MHz, Chloroform-*d*) (t, 77.36 ppm) of **10p**.

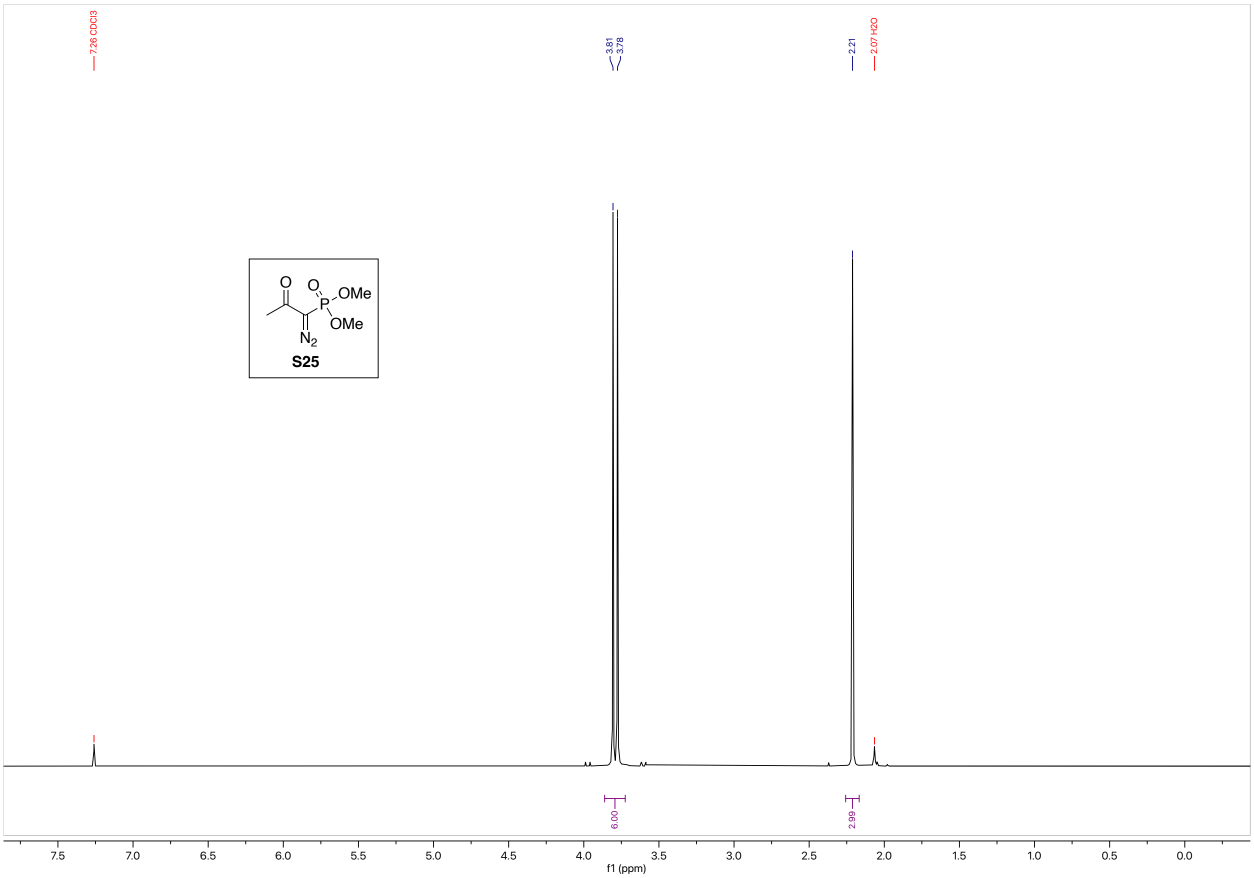

^{1}H NMR spectrum (400 MHz, Chloroform-*d*) (s, 7.26 ppm) of **S25**.

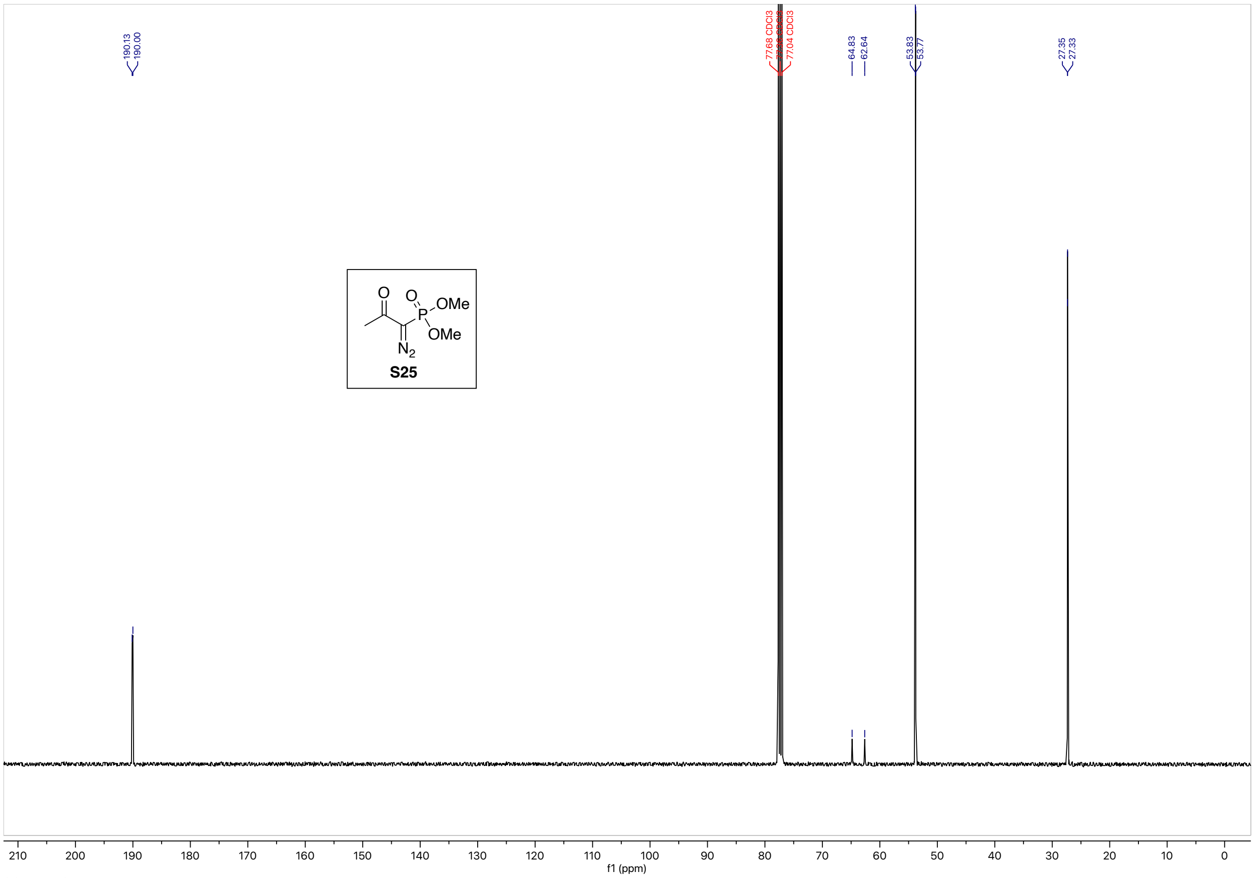

$^{13}C\{^{1}H\}$ NMR spectrum (101 MHz, Chloroform-*d*) (t, 77.36 ppm) of **S25**.

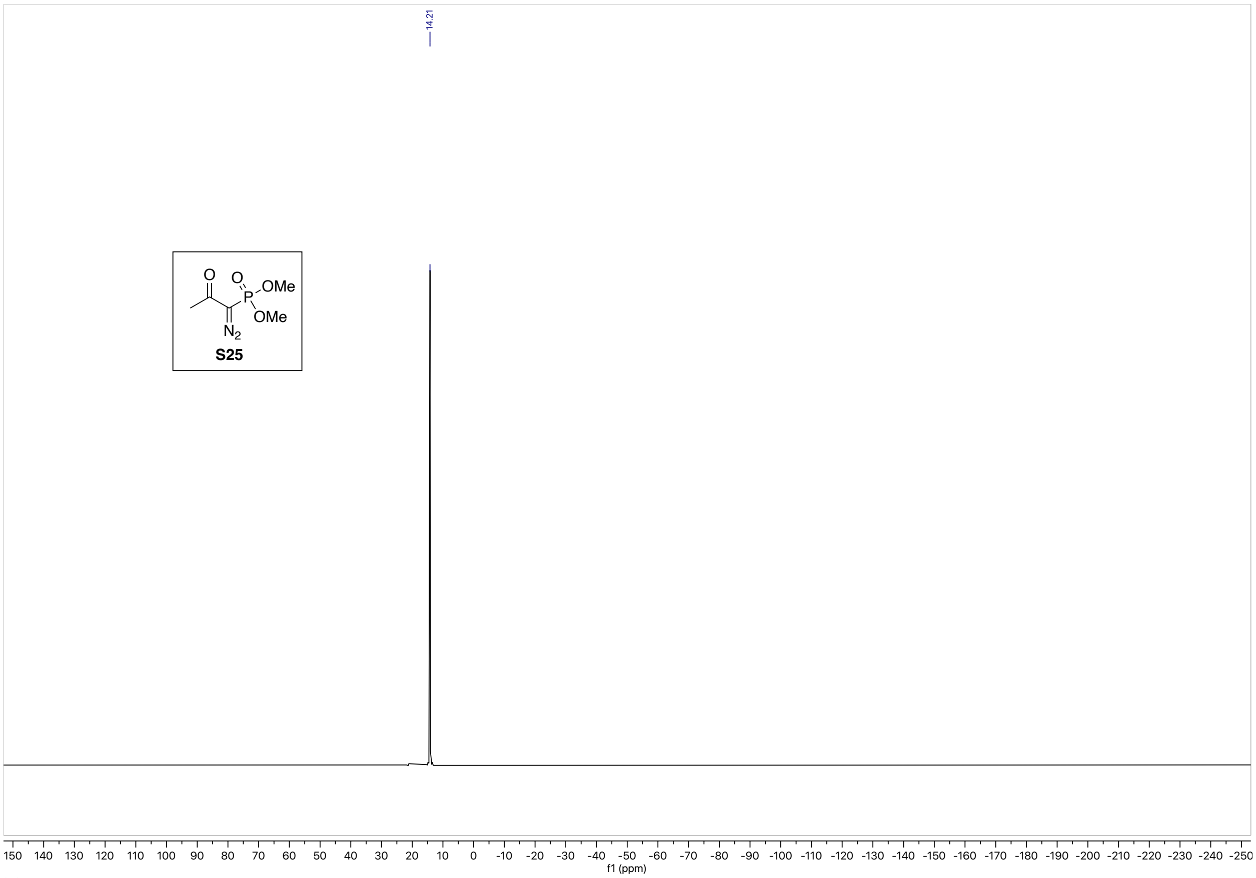

^{31}P NMR spectrum (162 MHz, Chloroform-d) of **S25**.

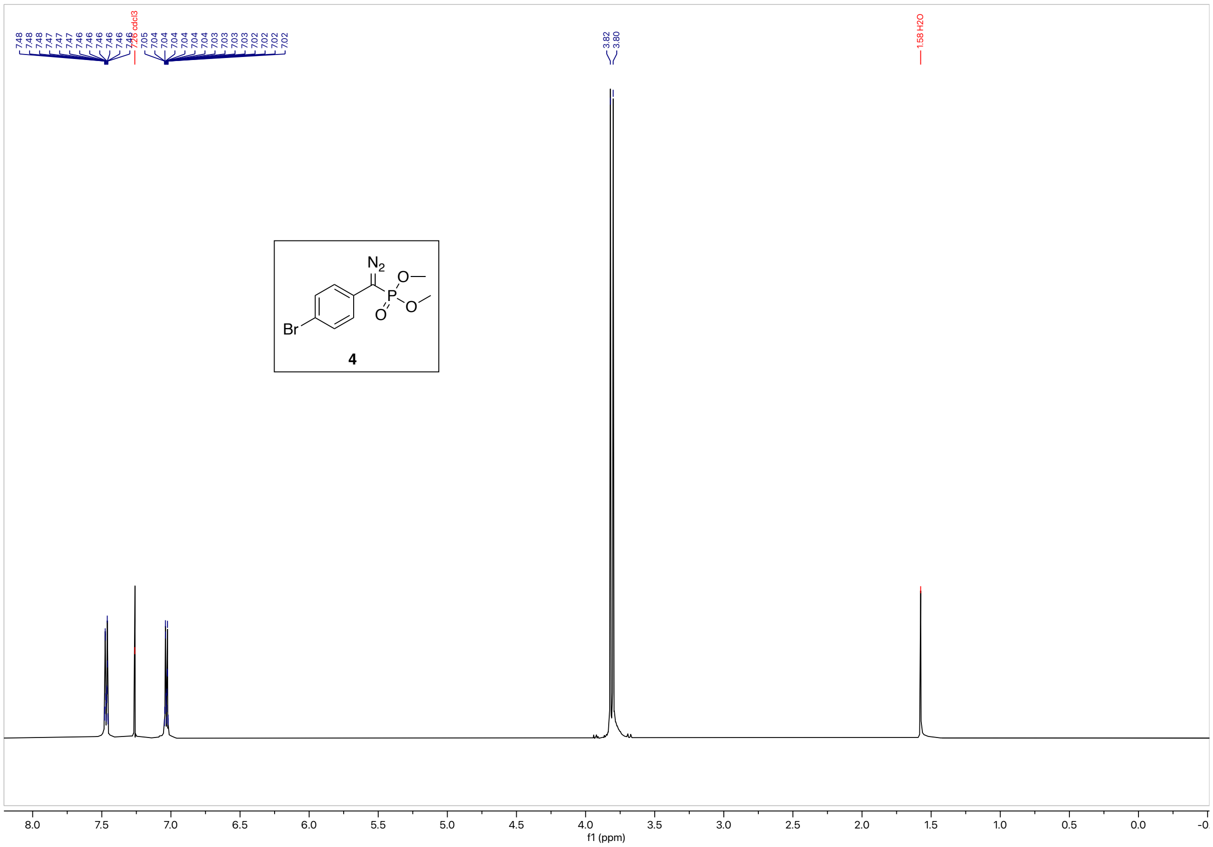

^{1}H NMR spectrum (600 MHz, Chloroform-*d*) (s, 7.26 ppm) of **4**.

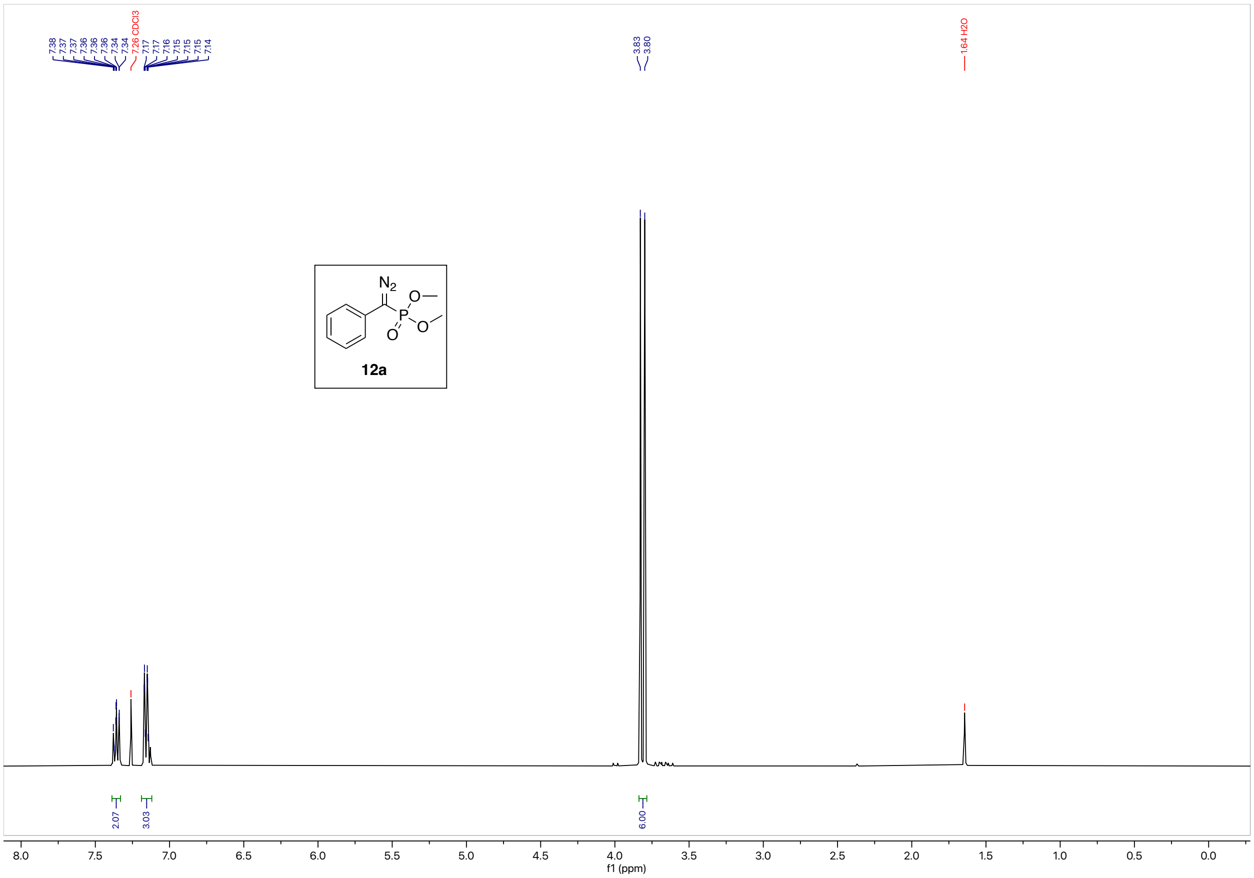

^{1}H NMR spectrum (400 MHz, Chloroform-*d*) (s, 7.26 ppm) of **12a**.

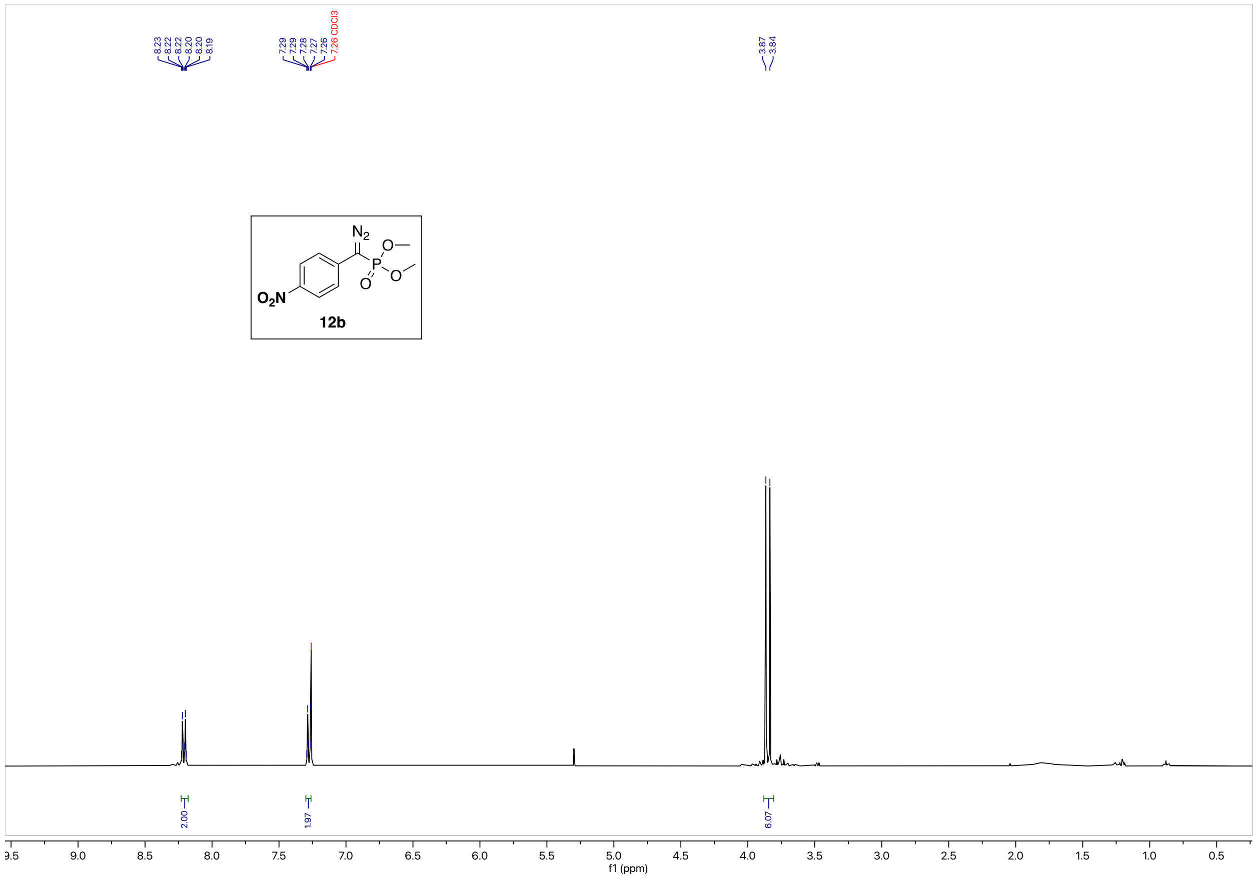

^{1}H NMR spectrum (400 MHz, Chloroform-*d*) (s, 7.26 ppm) of **12b**.

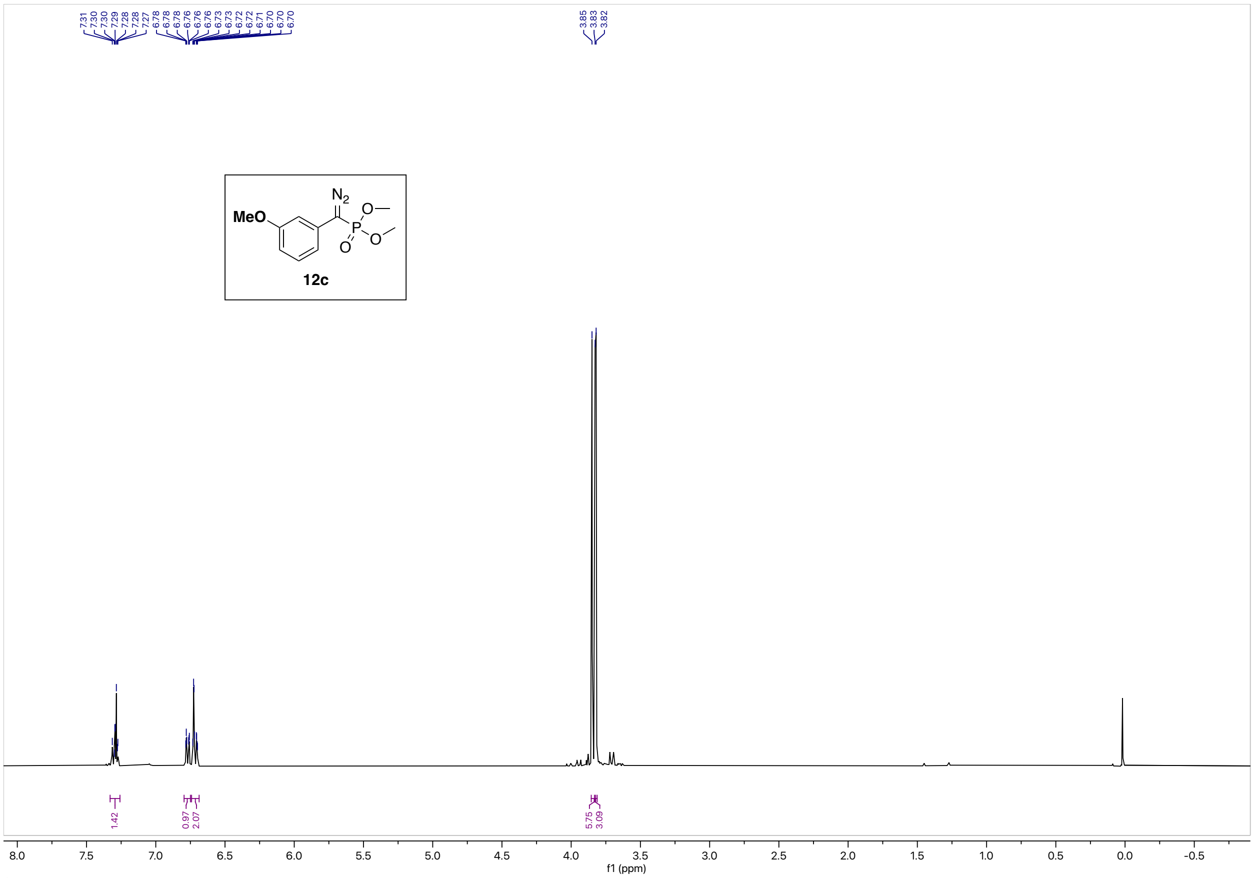

^{1}H NMR spectrum (400 MHz, Chloroform-*d*) (s, 7.26 ppm) of **12c**.

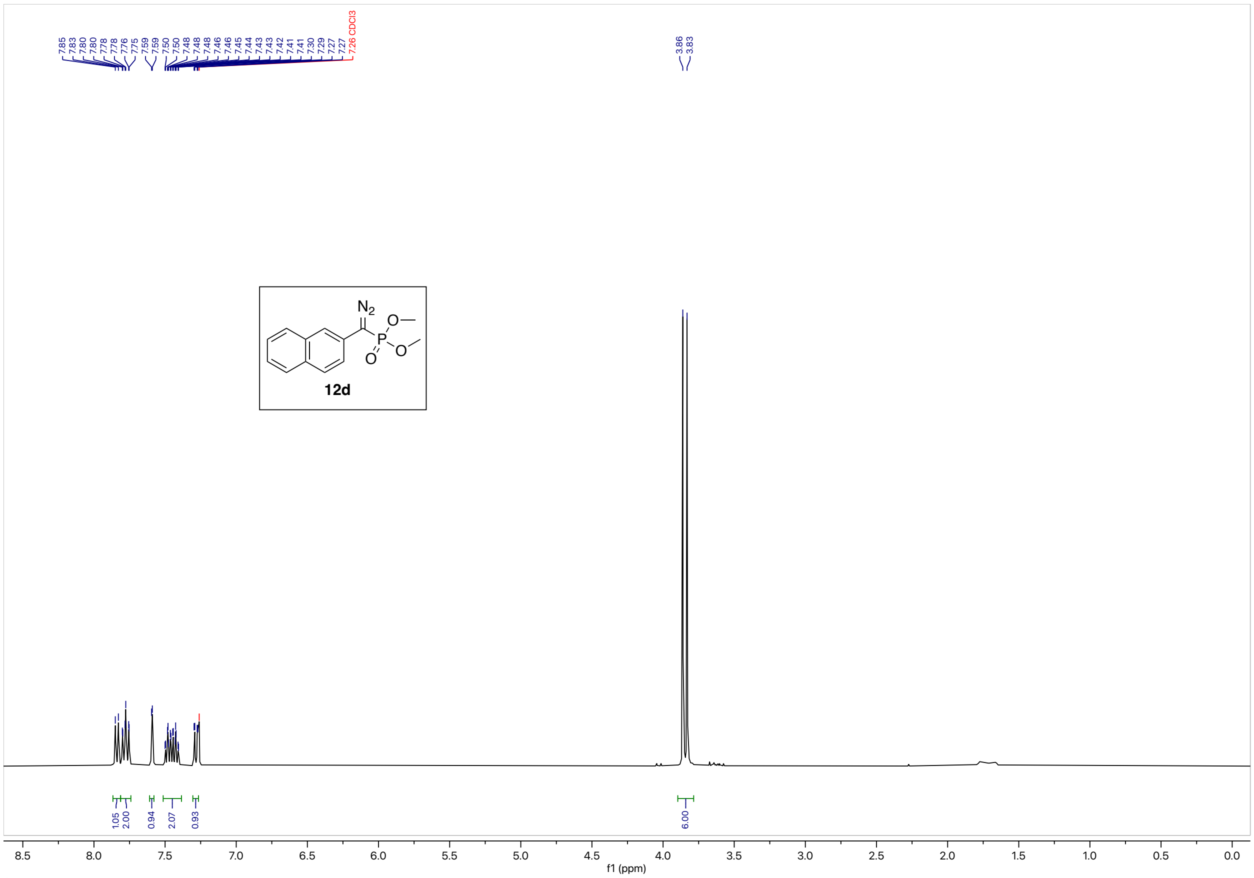

^{1}H NMR spectrum (400 MHz, Chloroform-*d*) (s, 7.26 ppm) of **12d**.

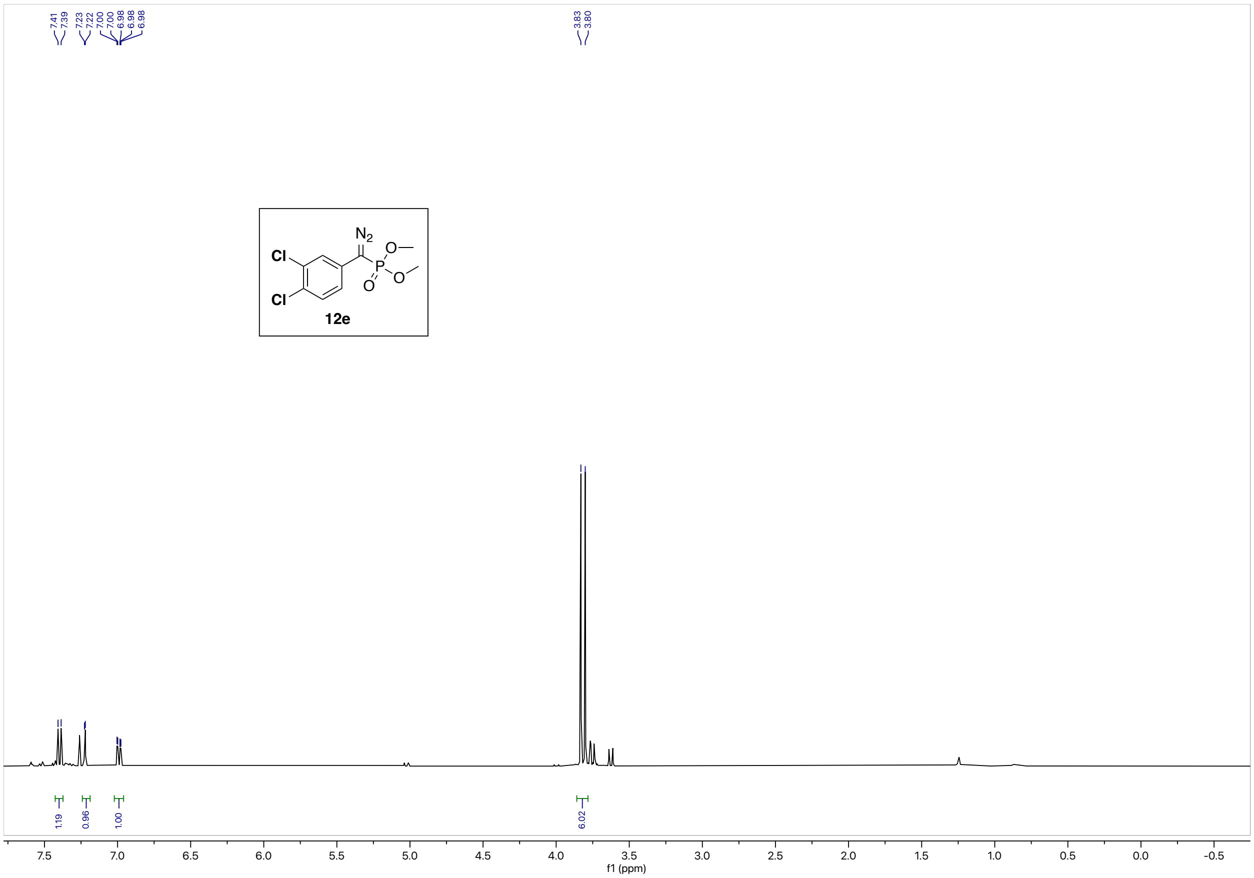

^{1}H NMR spectrum (400 MHz, Chloroform-*d*) (s, 7.26 ppm) of **12e**.

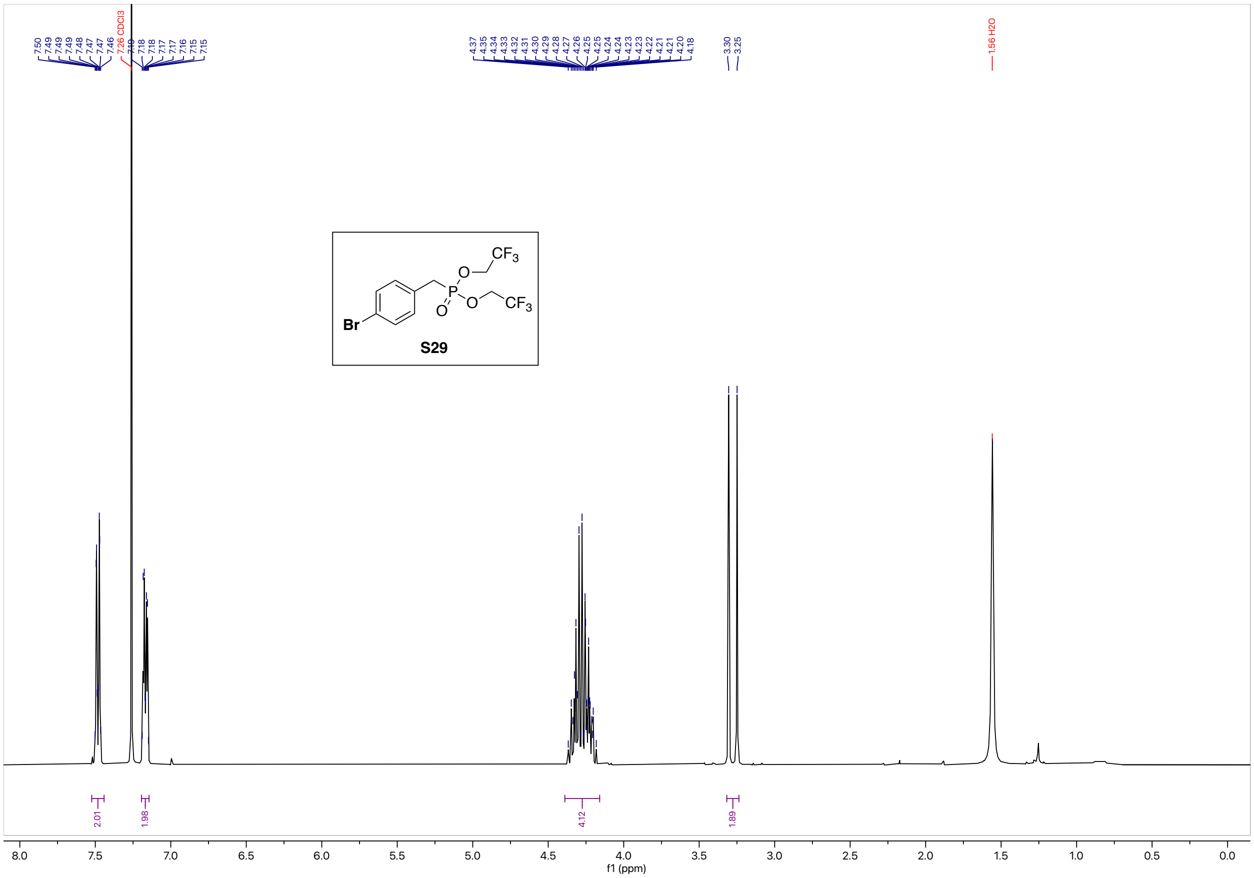

^{1}H NMR spectrum (400 MHz, Chloroform-*d*) (s, 7.26 ppm) of **S29**.

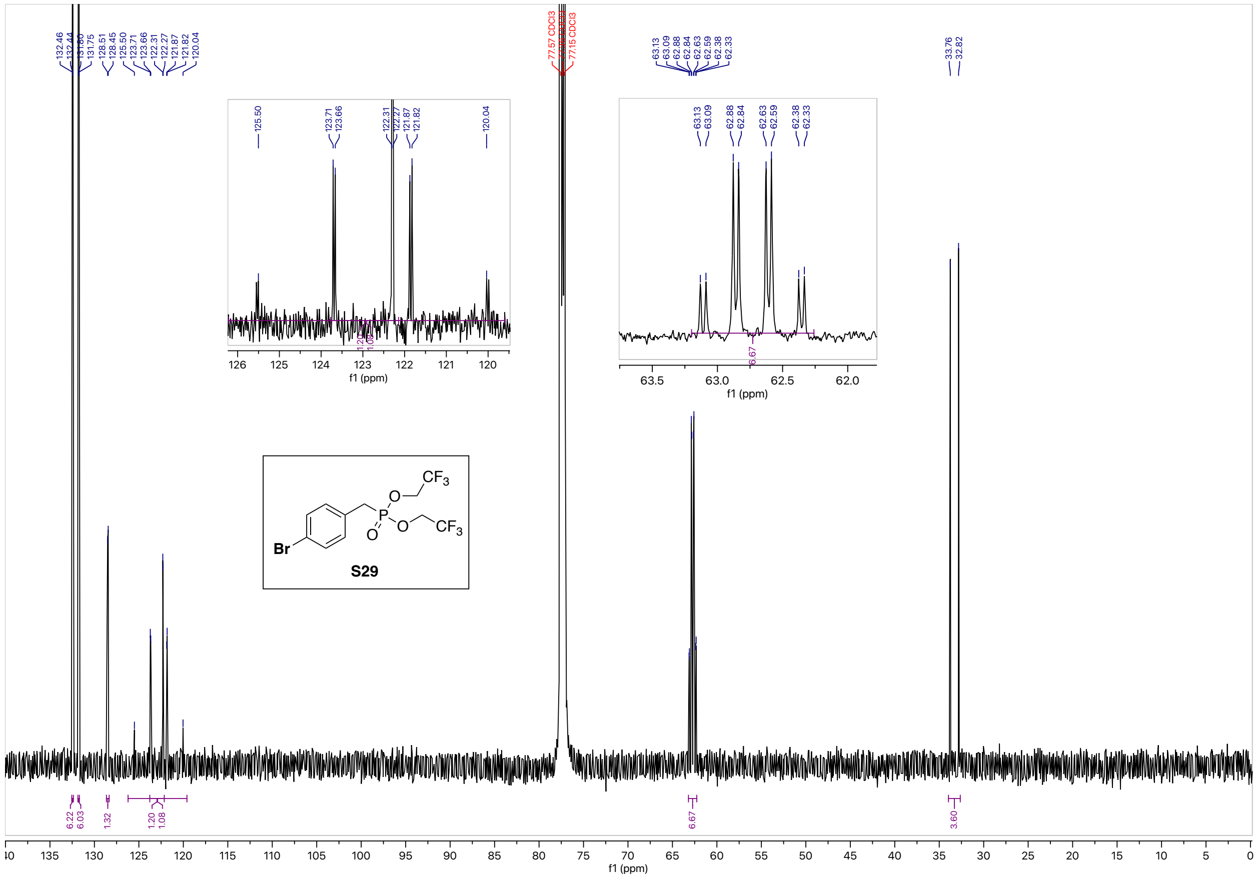

$^{13}C\{^{1}H\}$ NMR spectrum (101 MHz, Chloroform-*d*) (t, 77.36 ppm) of **S29**.

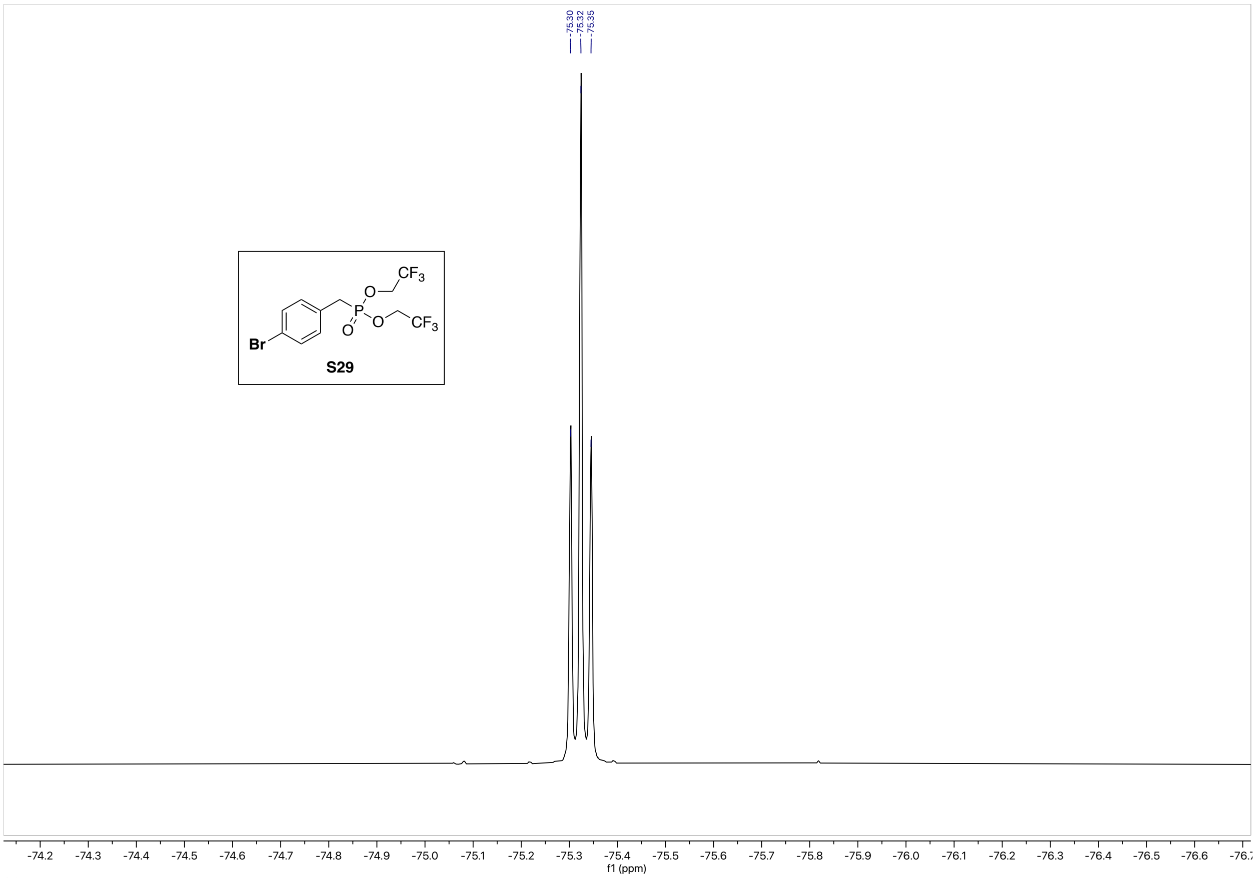

^{19}F NMR spectrum (376 MHz, Chloroform-d) of **S29**.

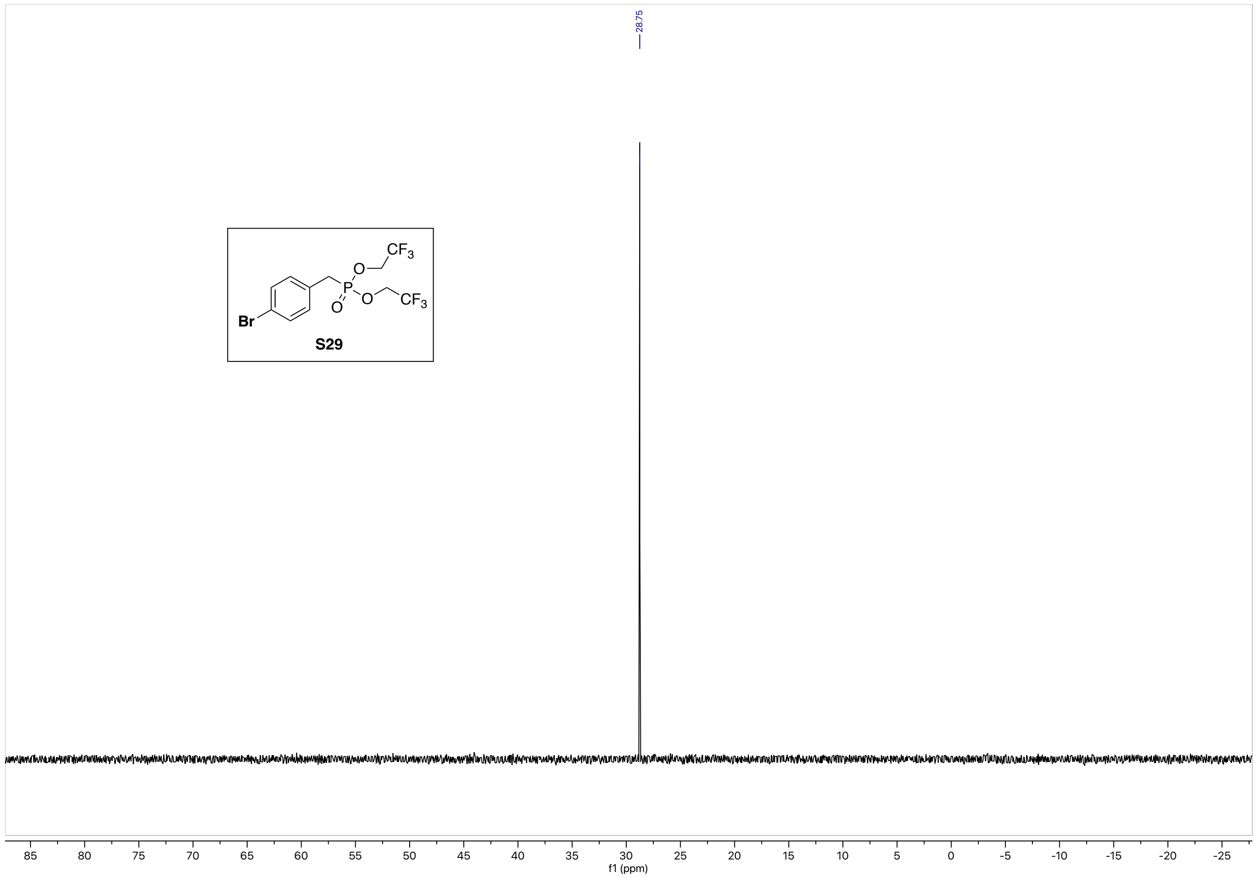

^{31}P NMR spectrum (162 MHz, Chloroform-d) of **S29**.

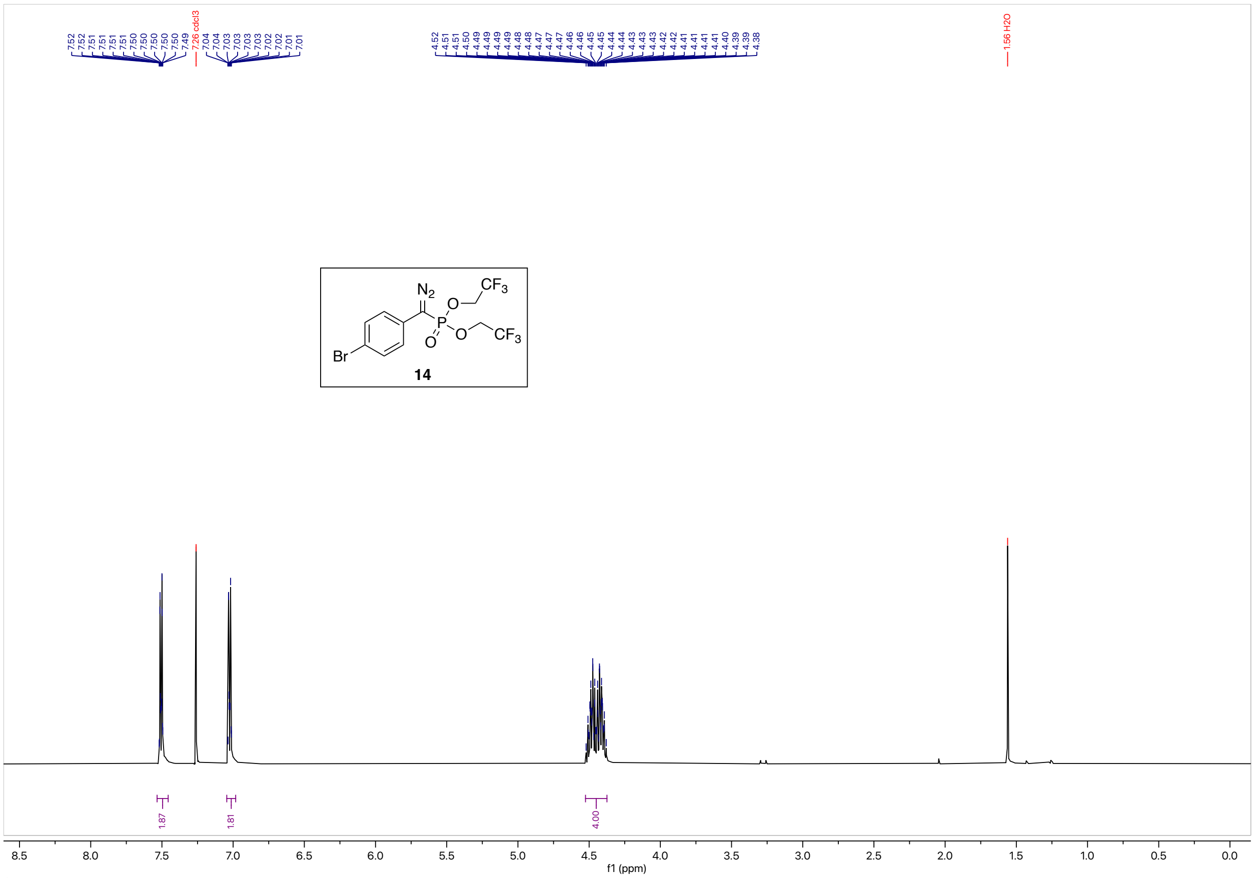

^{1}H NMR spectrum (600 MHz, Chloroform-*d*) (s, 7.26 ppm) of **14**.

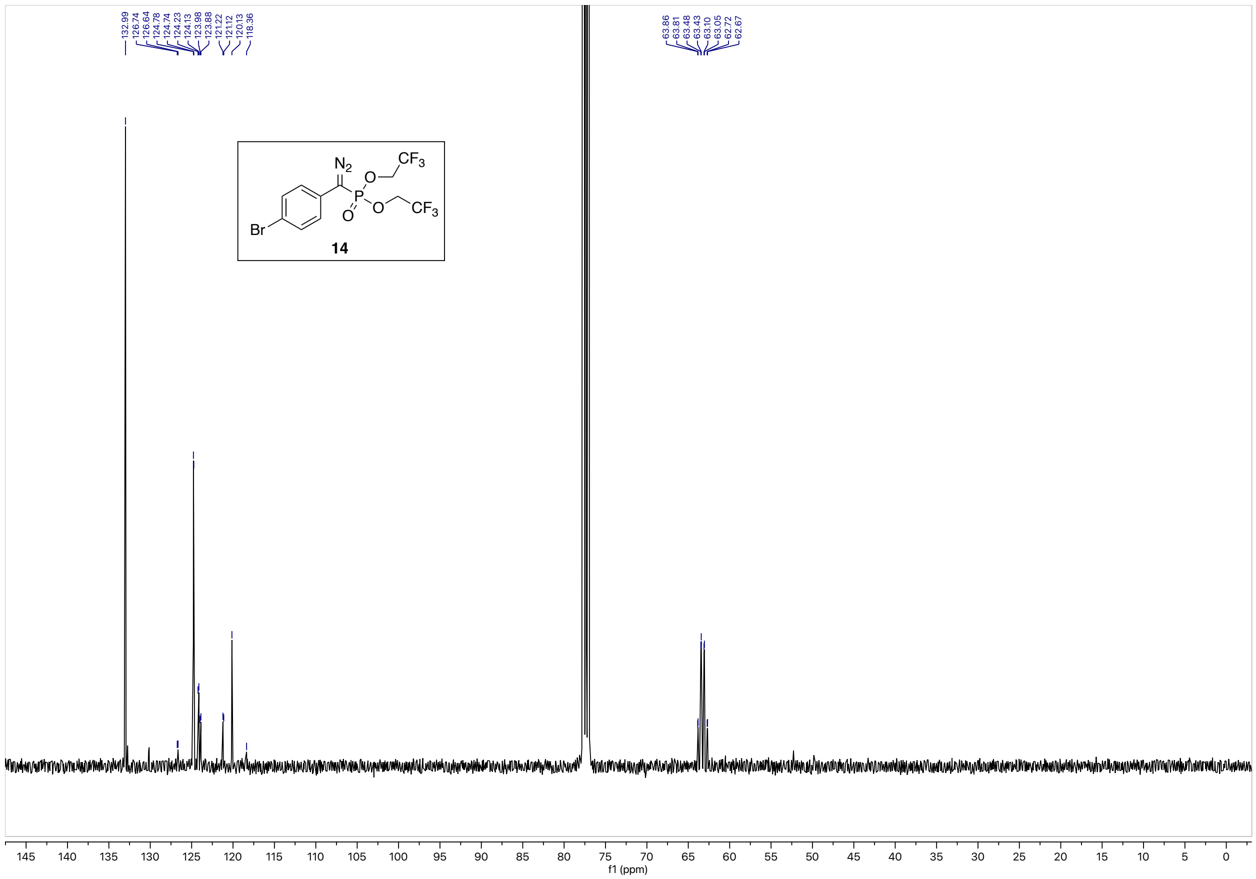

$^{13}C\{^1H\}$ NMR spectrum (101 MHz, Chloroform-*d*) (t, 77.36 ppm) of **14**.

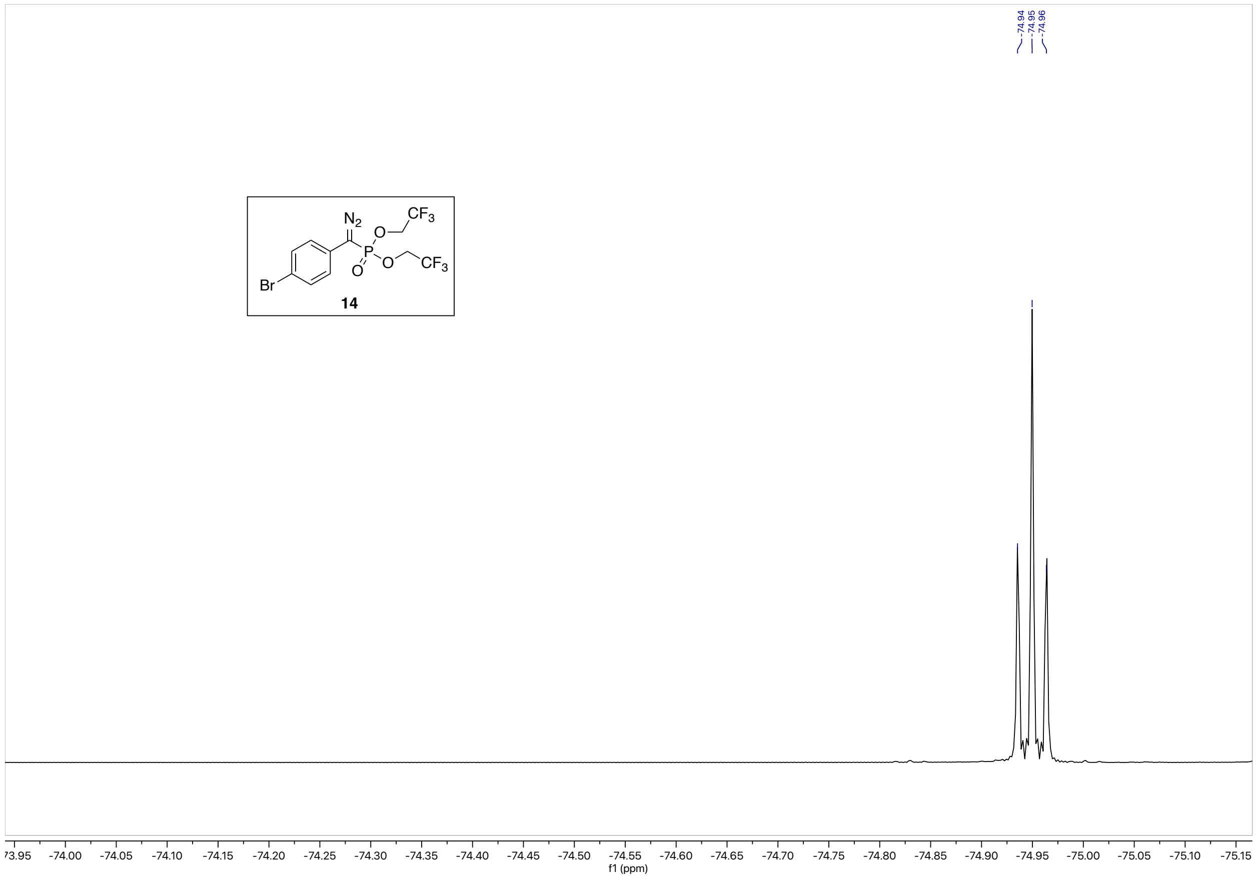

^{19}F NMR spectrum (565 MHz, Chloroform-d) of **14**.

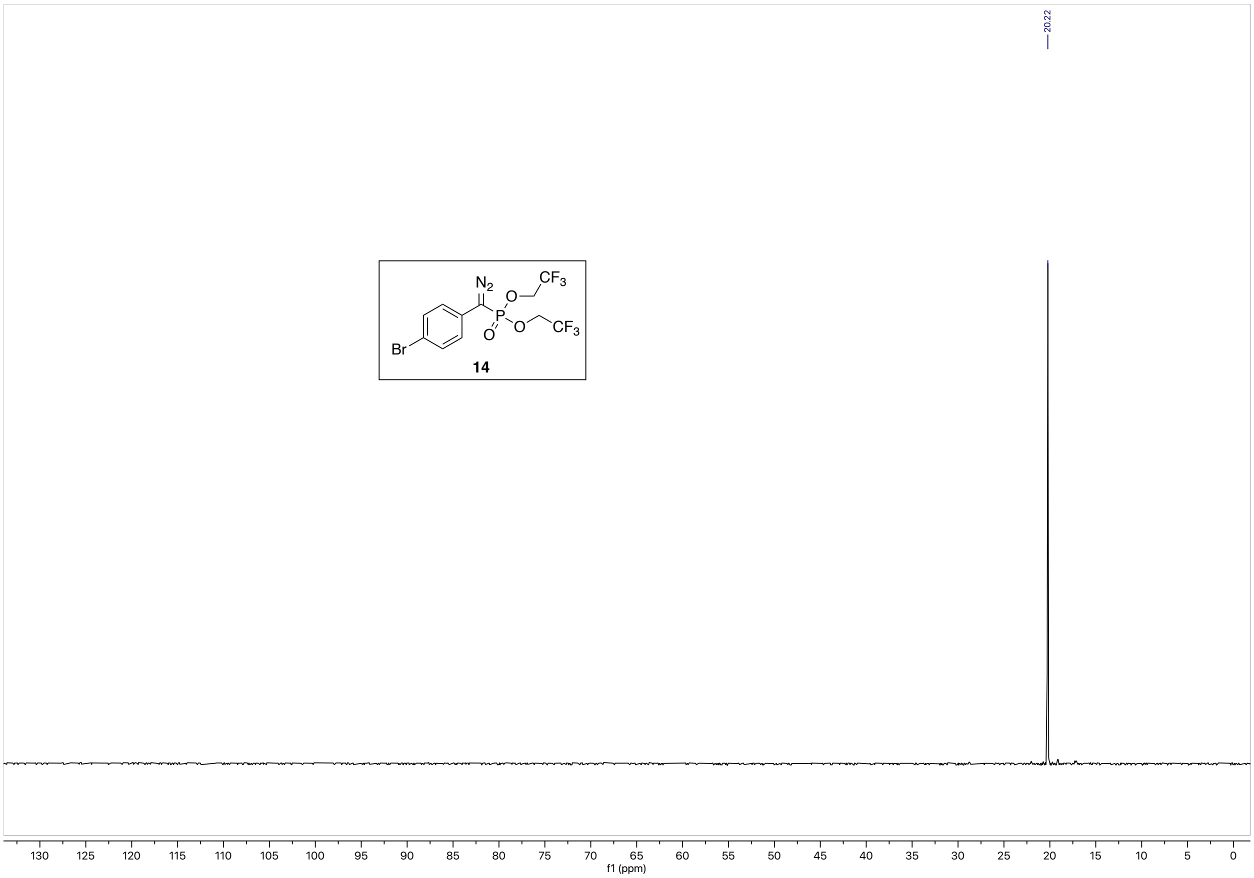

^{31}P NMR spectrum (243 MHz, Chloroform-d) of **14**.

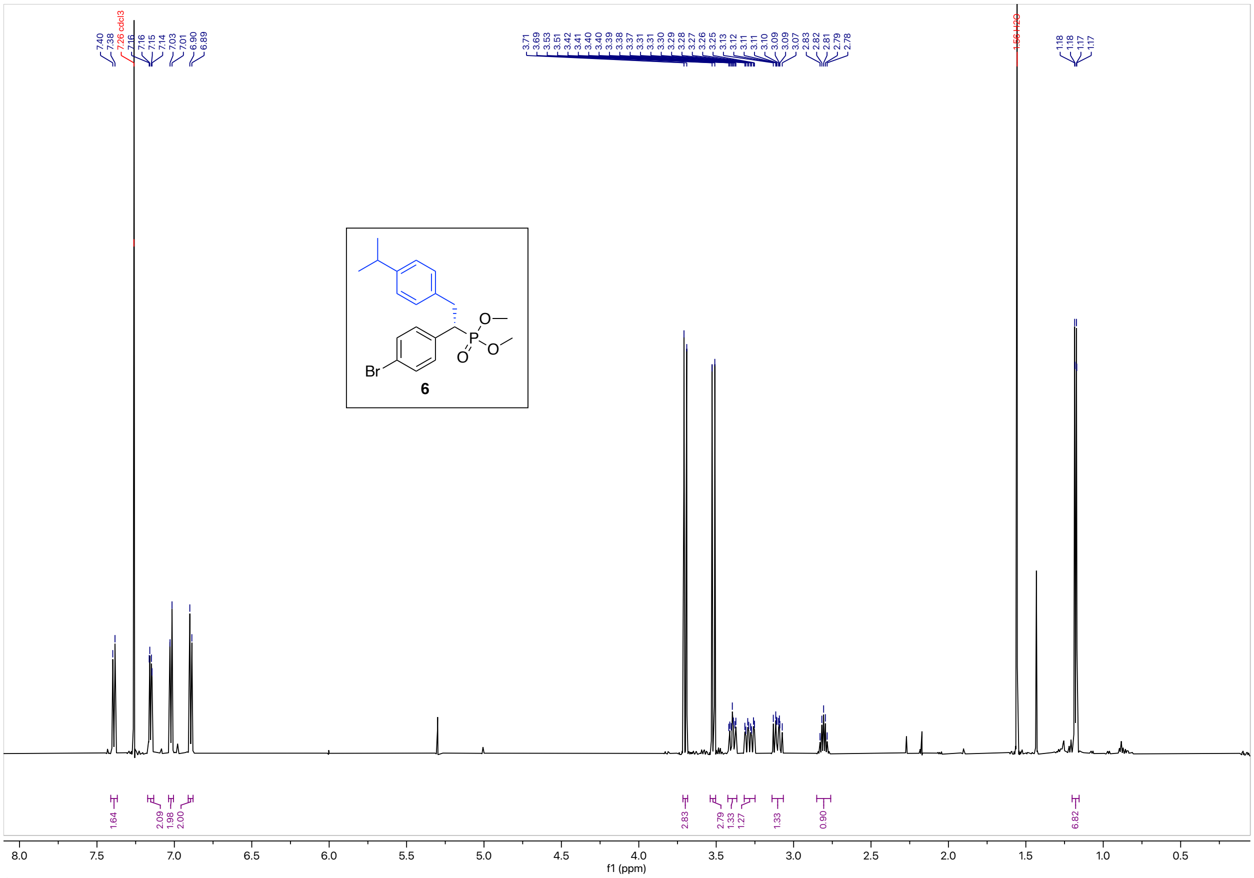

^{1}H NMR spectrum (600 MHz, Chloroform-*d*) (s, 7.26 ppm) of **6**.

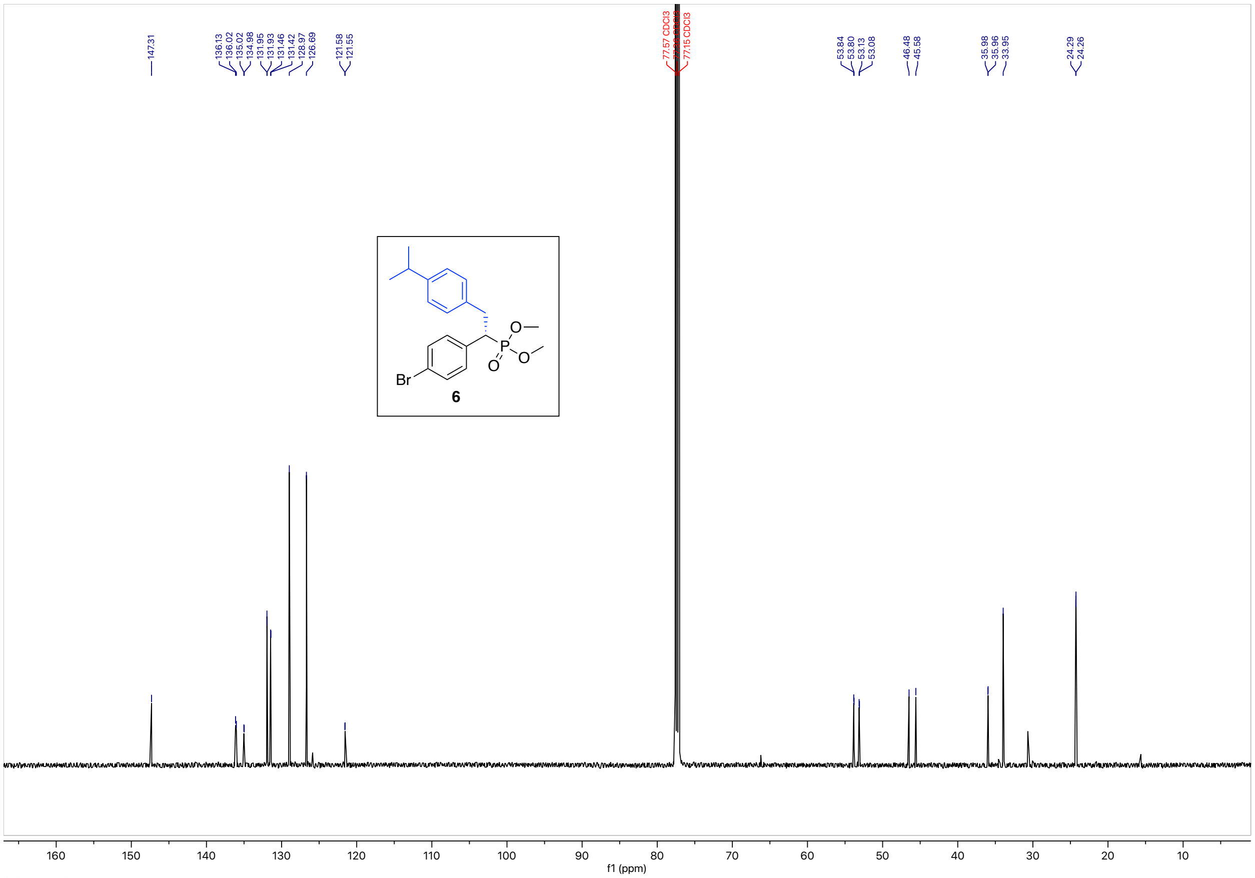

$^{13}C\{^{1}H\}$ NMR spectrum (151 MHz, Chloroform-*d*) (t, 77.36 ppm) of **6**.

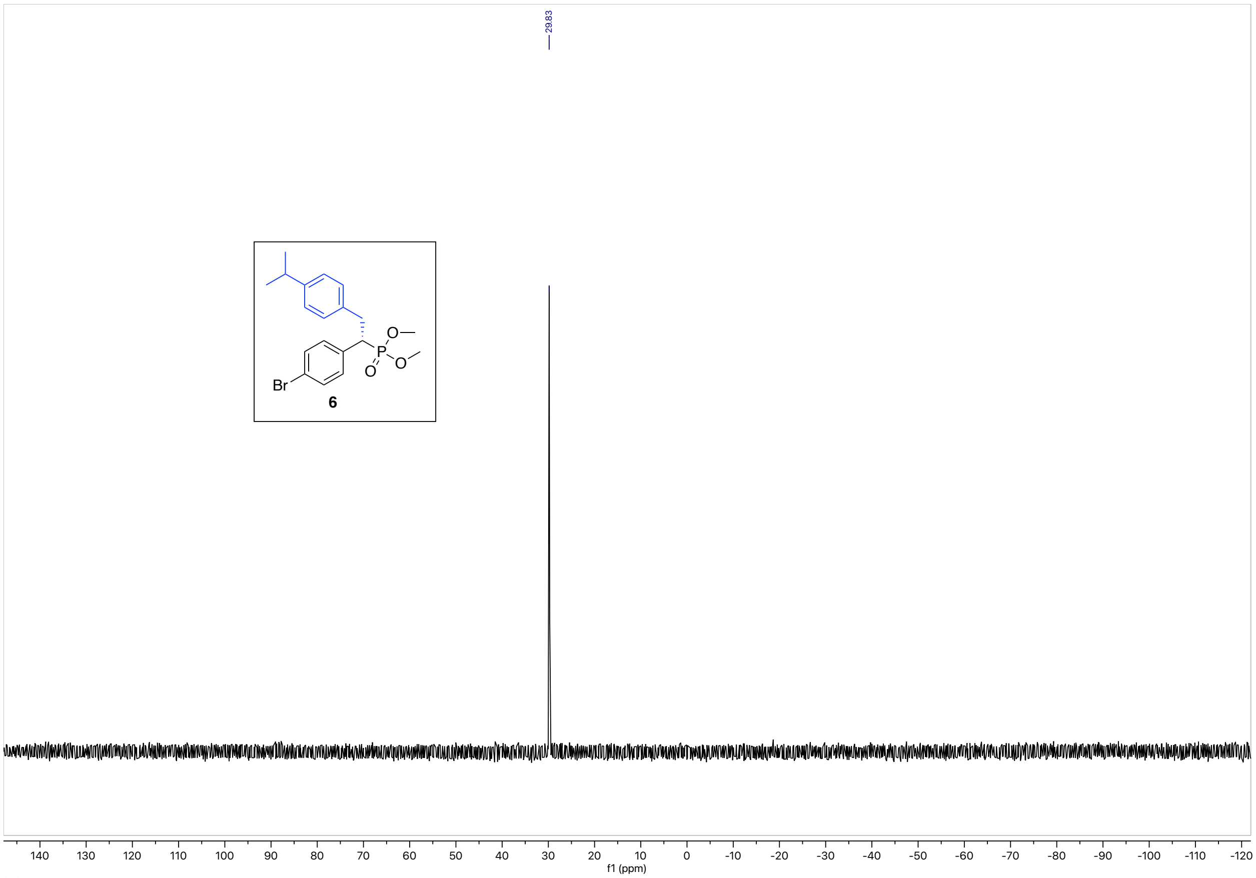

^{31}P NMR spectrum (243 MHz, Chloroform-*d*) of **6**.

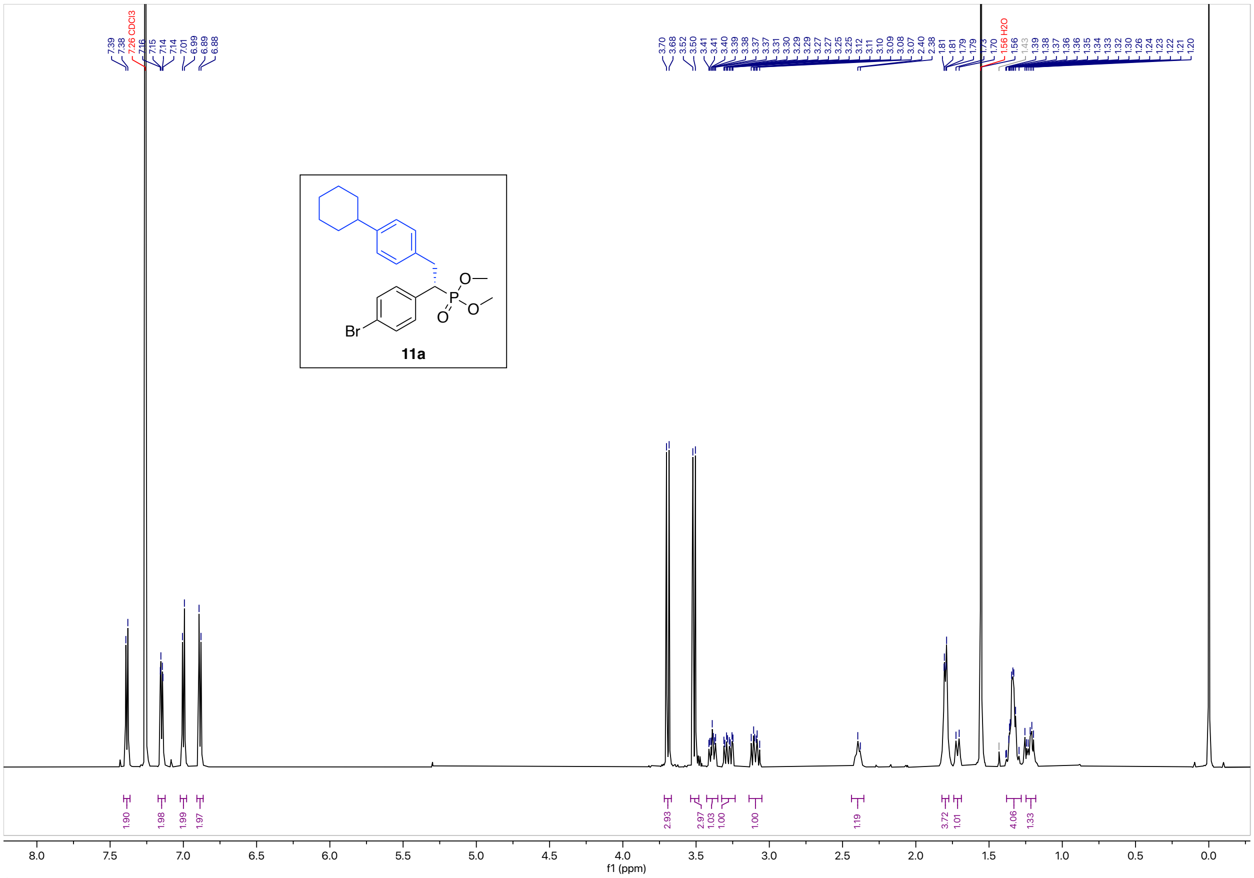

^{1}H NMR spectrum (600 MHz, Chloroform-*d*) (s, 7.26 ppm) of **11a**.

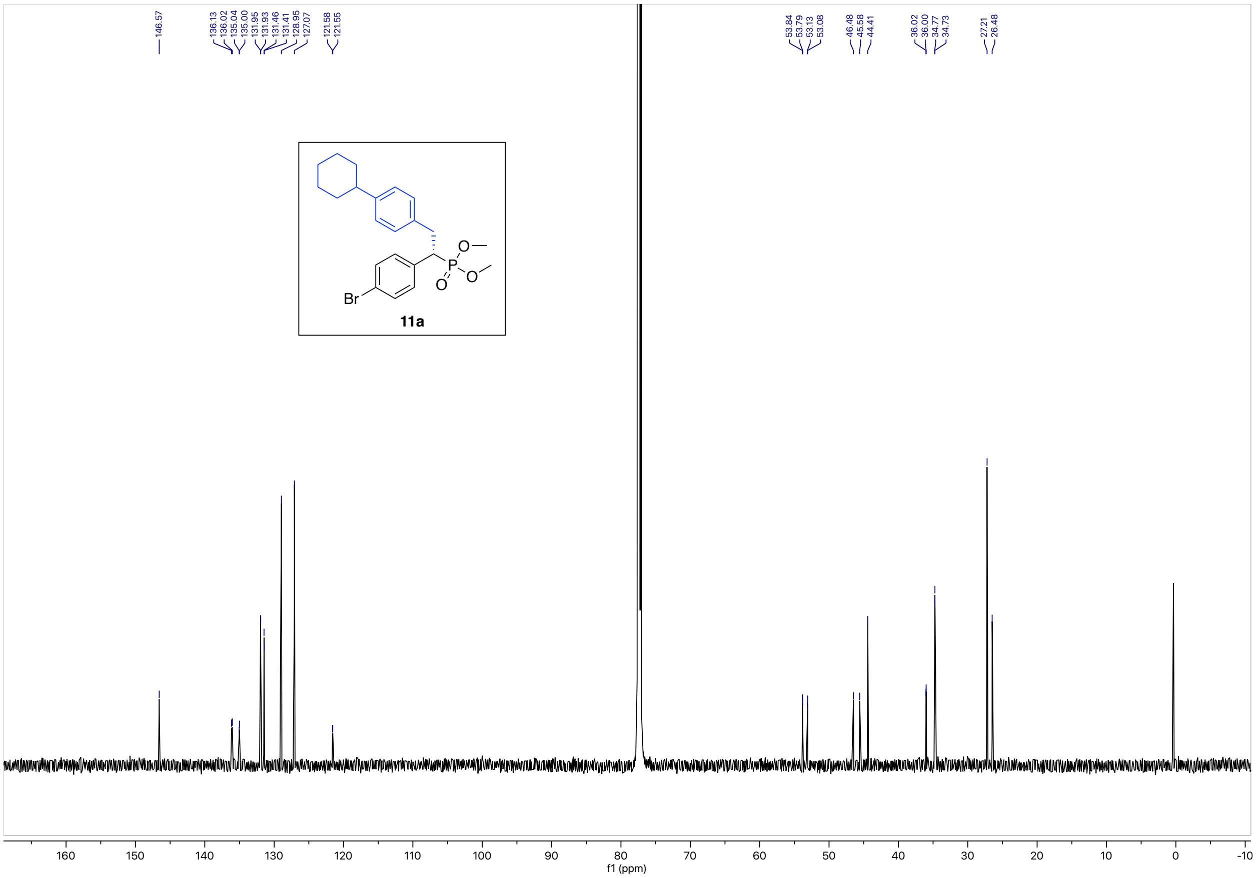

$^{13}C\{^{1}H\}$ NMR spectrum (151 MHz, Chloroform-*d*) (t, 77.36 ppm) of **11a**.

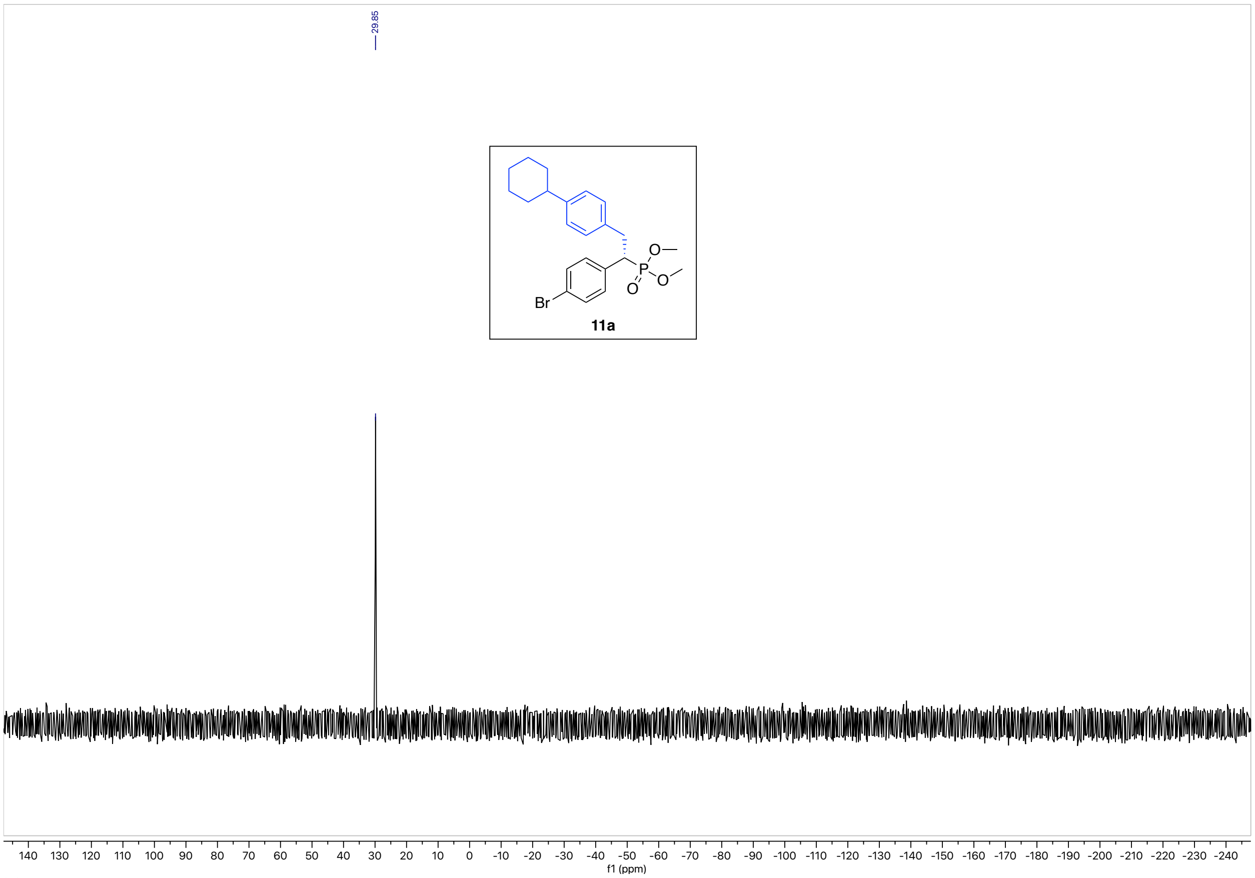

^{31}P NMR spectrum (243 MHz, Chloroform-*d*) of **11a**.

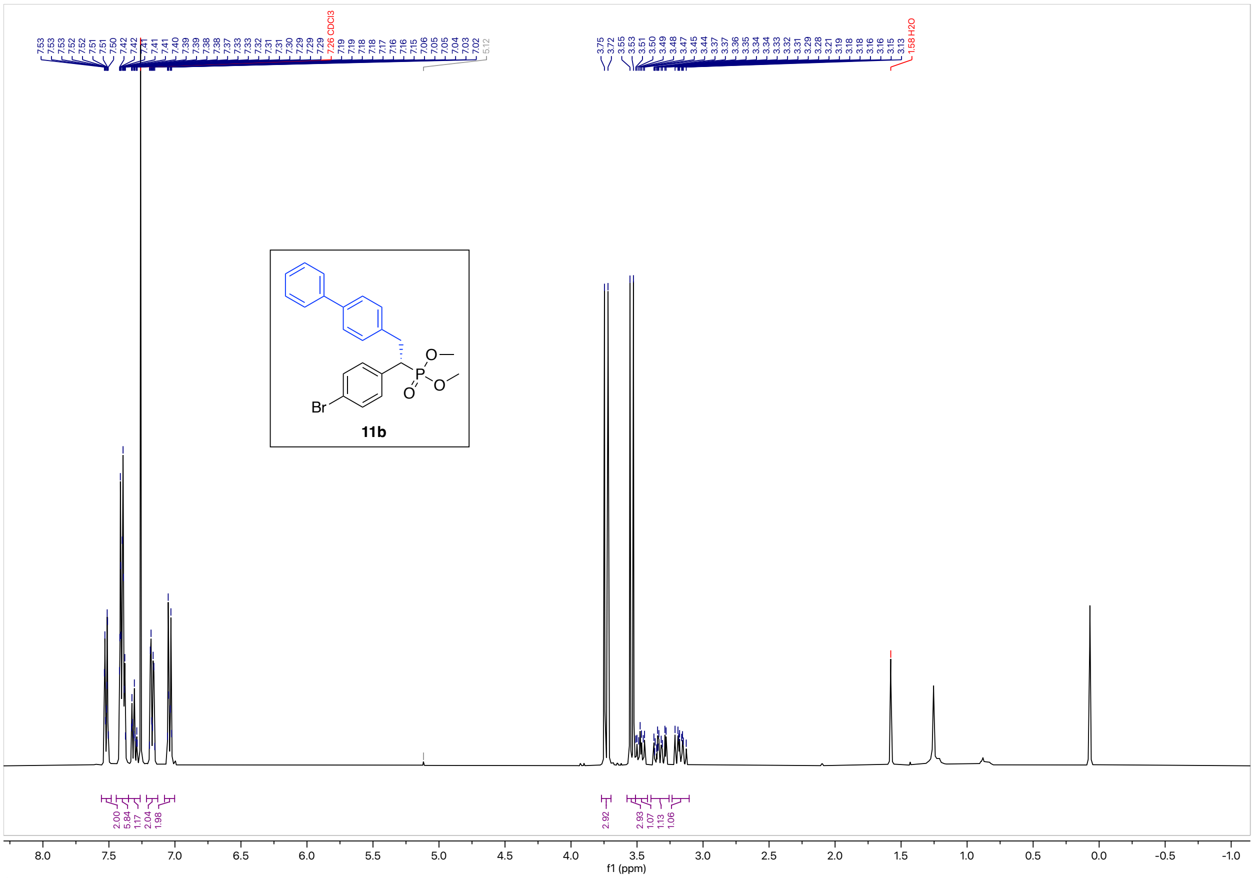

^{1}H NMR spectrum (400 MHz, Chloroform-*d*) (s, 7.26 ppm) of **11b**.

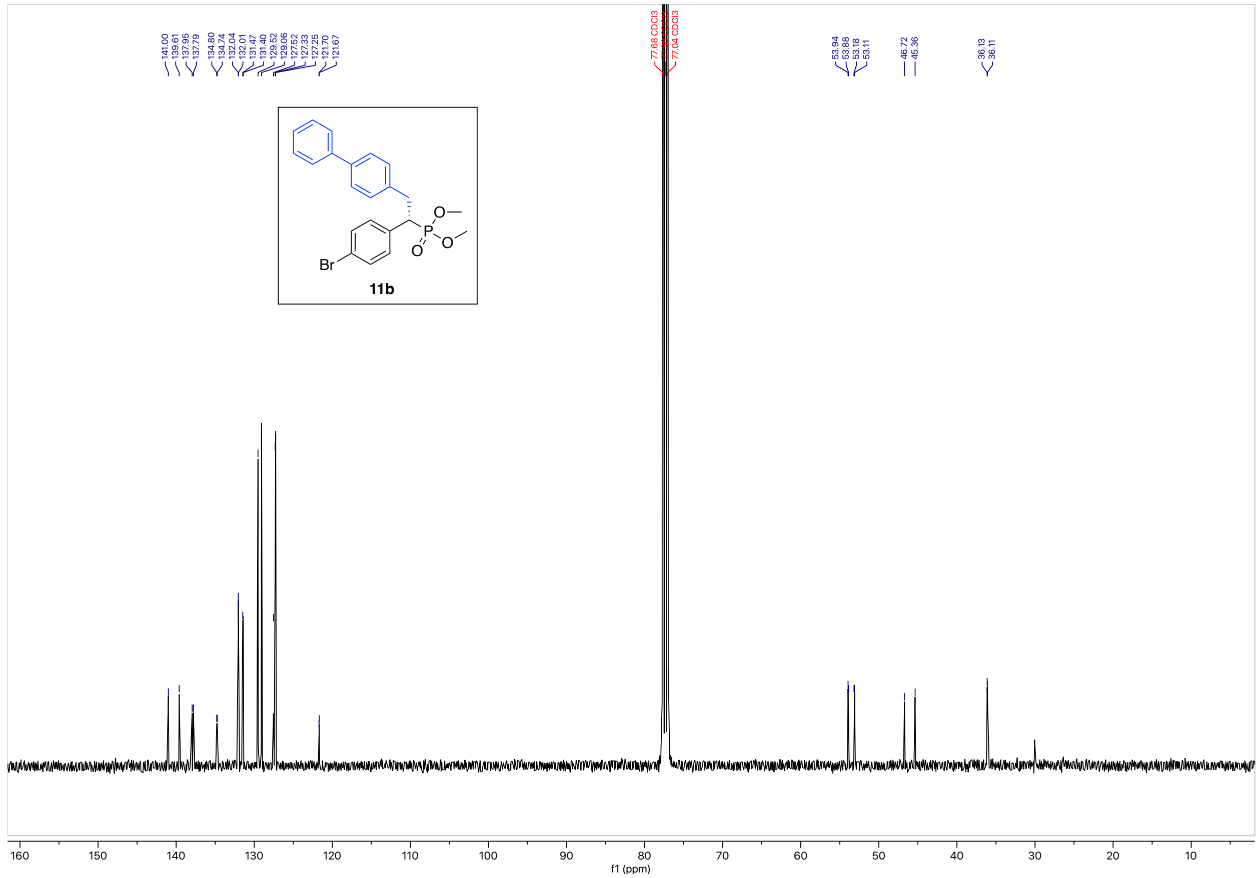

$^{13}C\{^1H\}$ NMR spectrum (101 MHz, Chloroform-*d*) (t, 77.36 ppm) of **11b**.

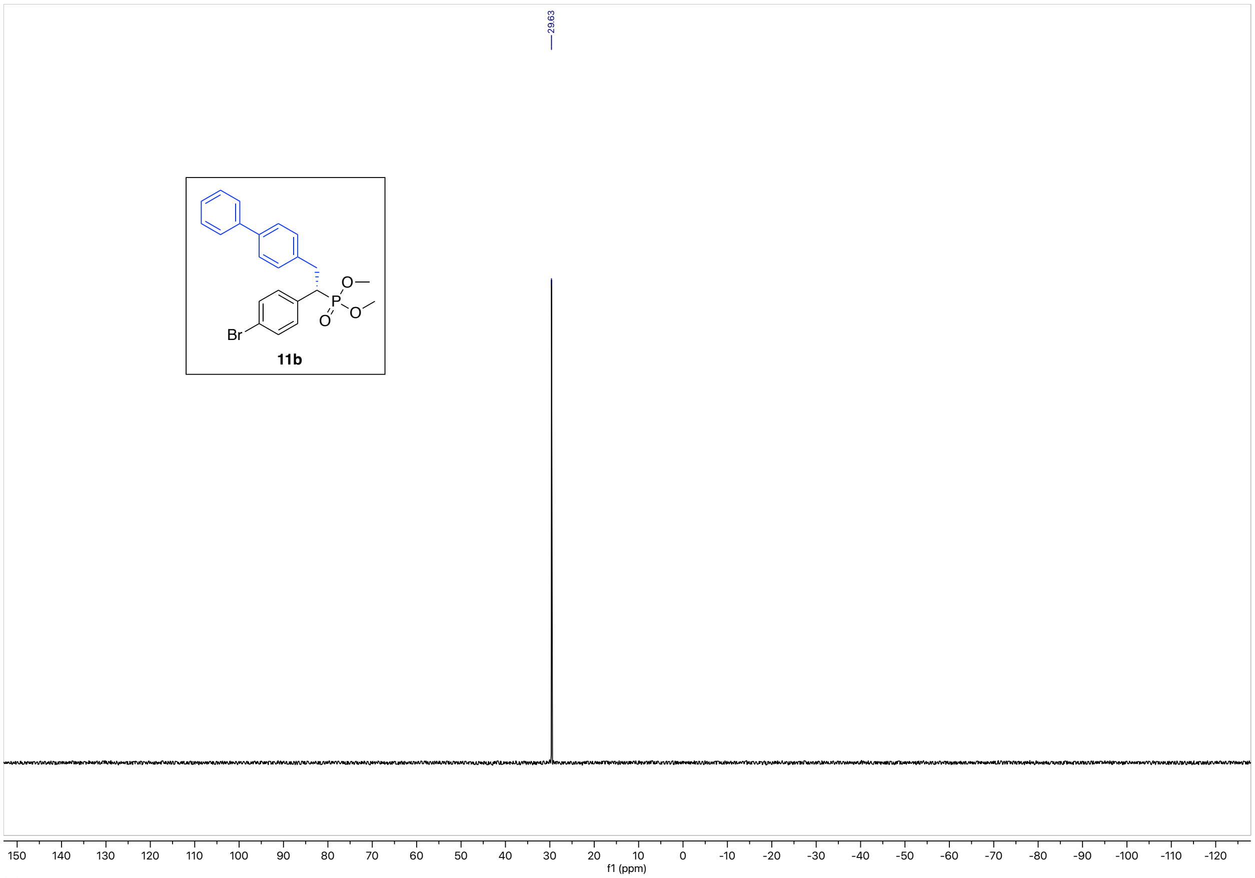

^{31}P NMR spectrum (162 MHz, Chloroform-*d*) of **11b**.

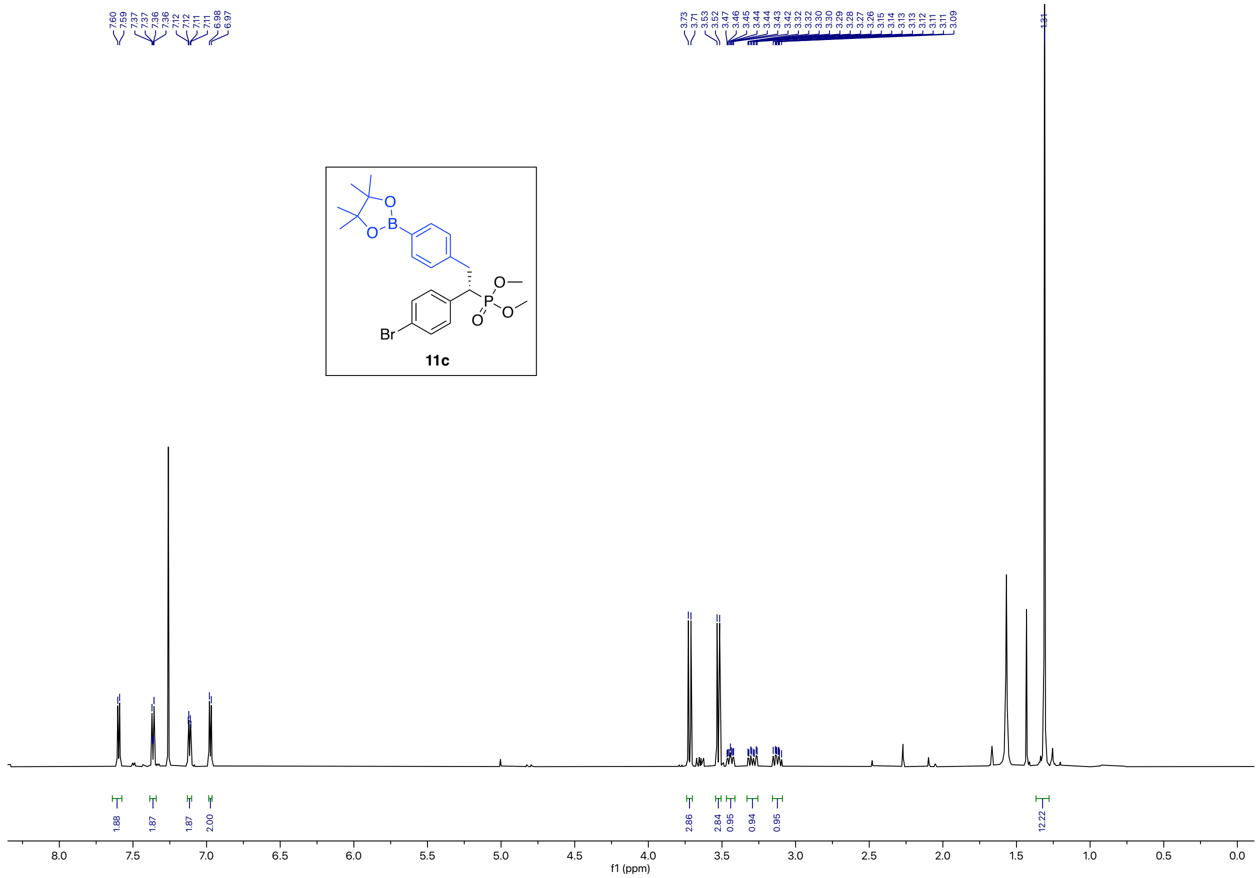

^{1}H NMR spectrum (600 MHz, Chloroform-*d*) (s, 7.26 ppm) of **11c**.

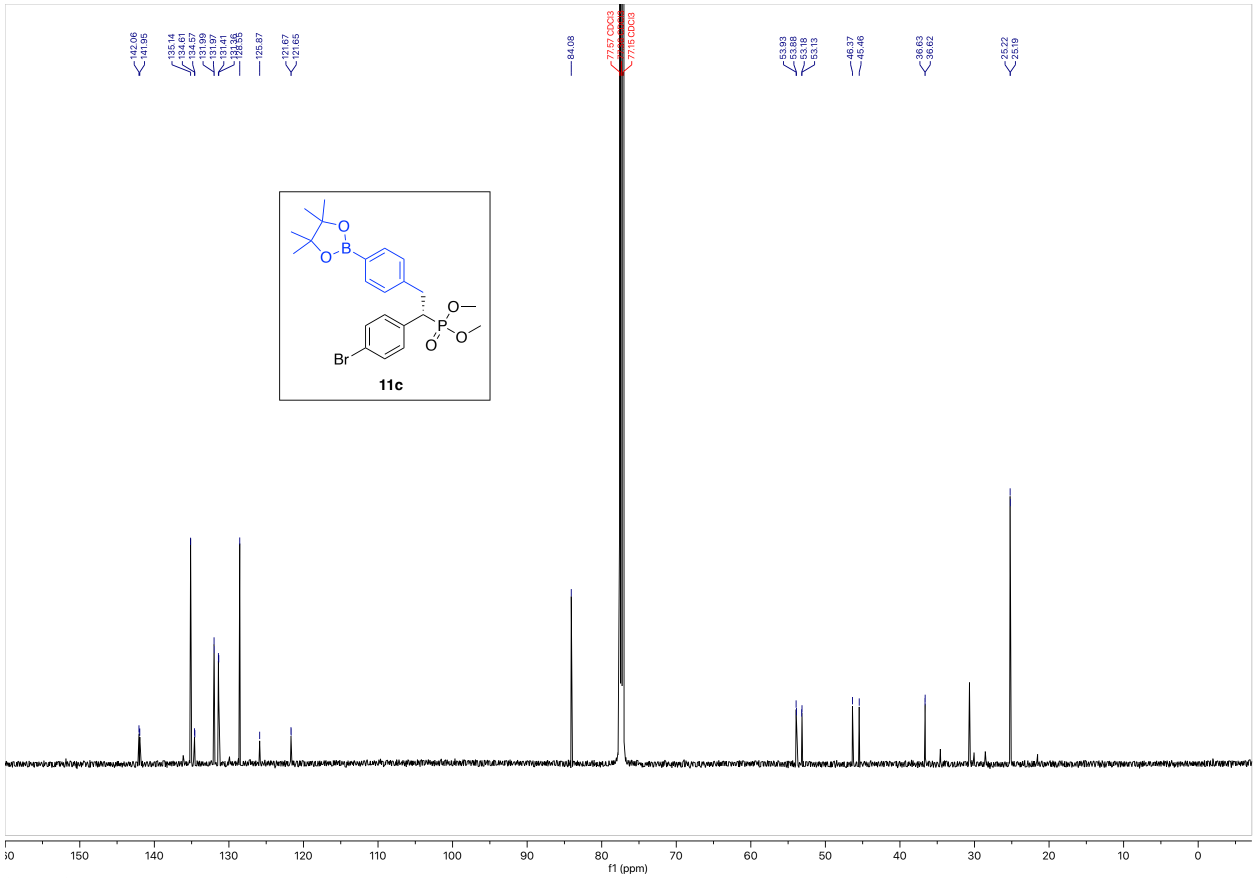

$^{13}C\{^1H\}$ NMR spectrum (151 MHz, Chloroform-*d*) (t, 77.36 ppm) of **11c**.

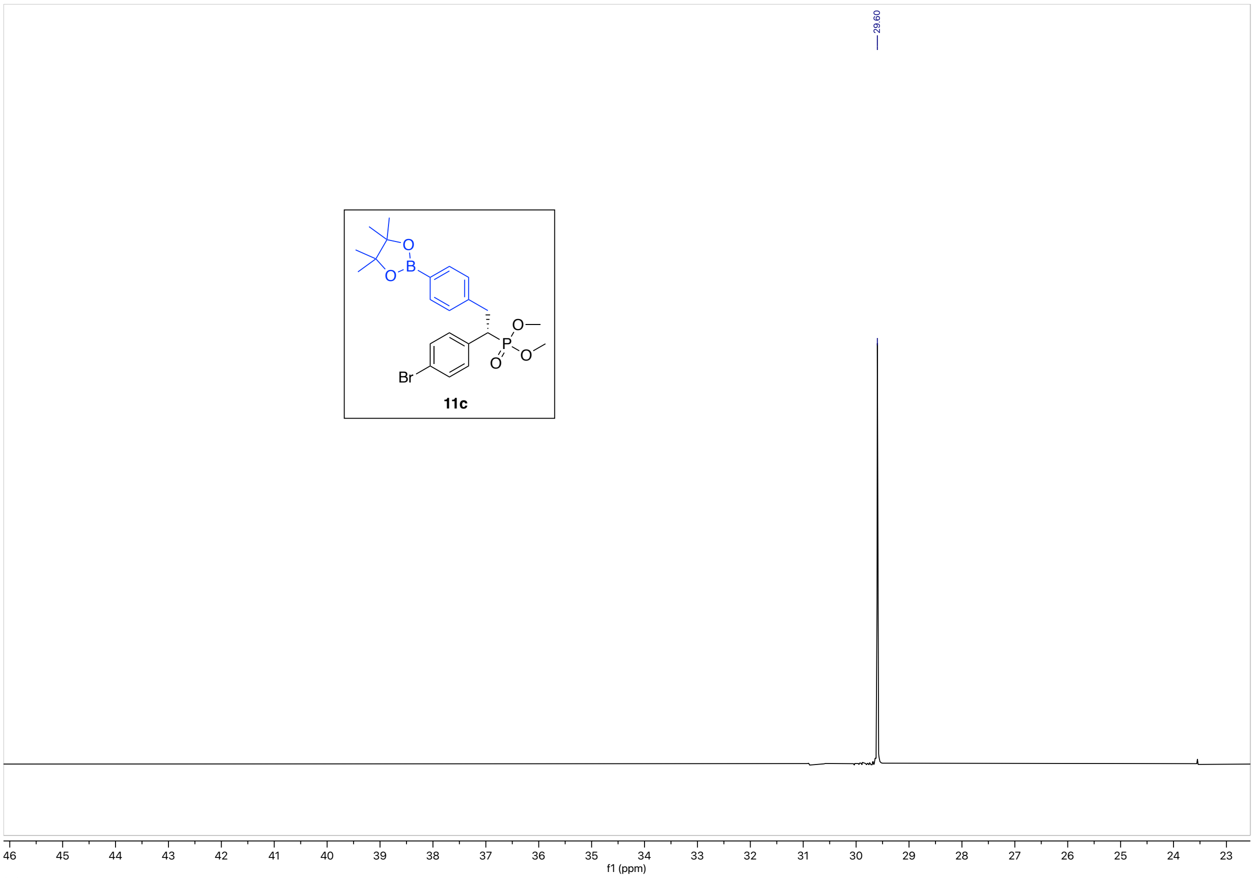

^{31}P NMR spectrum (243 MHz, Chloroform-*d*) of **11b**.

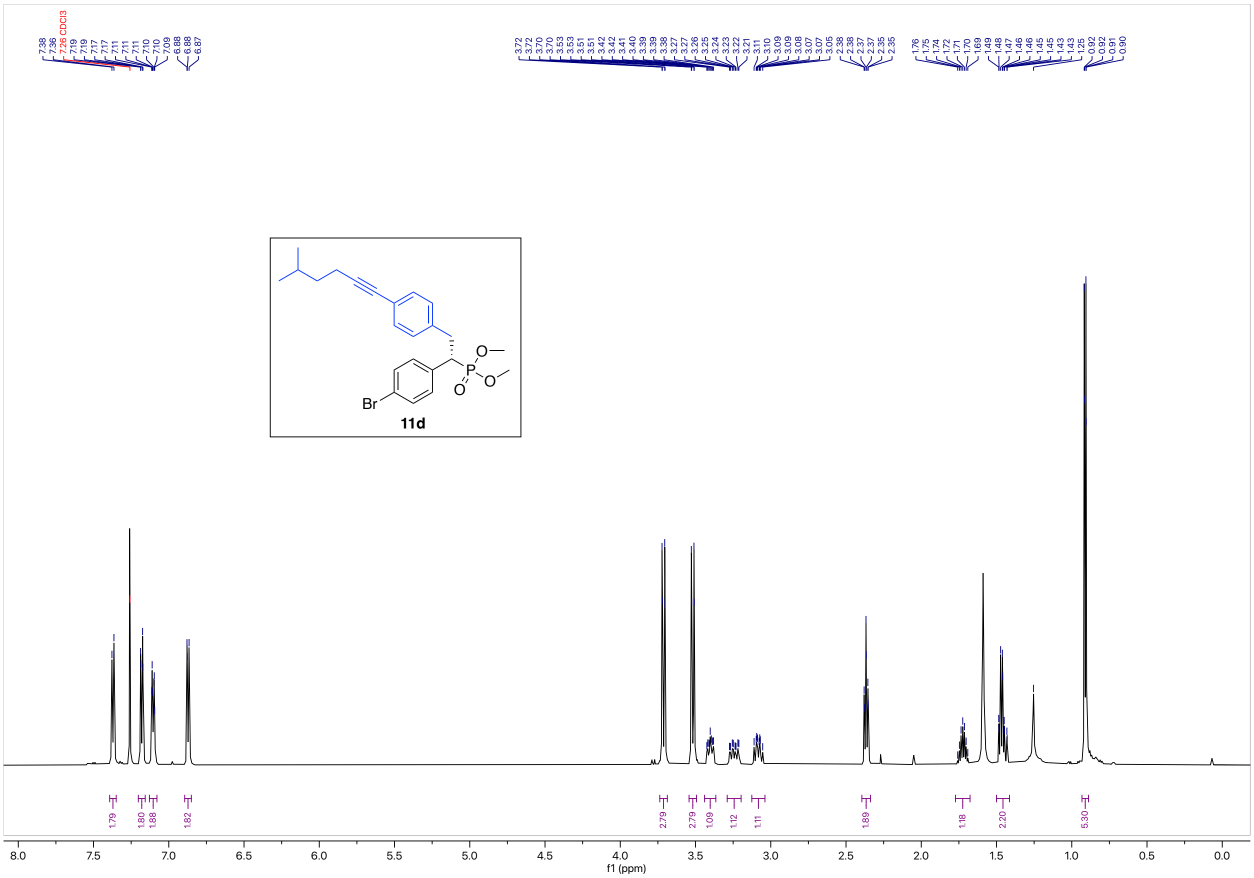

^{1}H NMR spectrum (600 MHz, Chloroform-*d*) (s, 7.26 ppm) of **11d**.

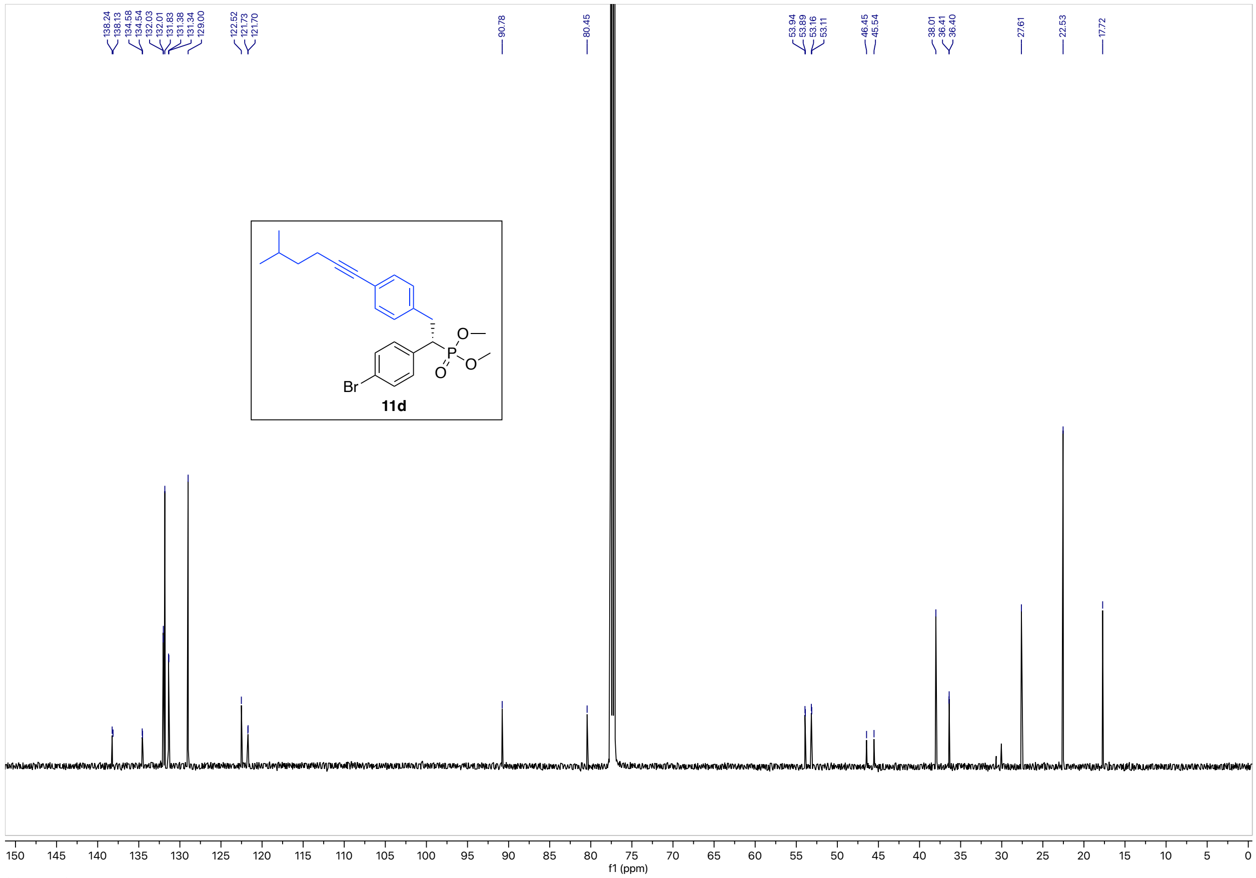

$^{13}C\{^{1}H\}$ NMR spectrum (151 MHz, Chloroform-*d*) (t, 77.36 ppm) of **11d**.

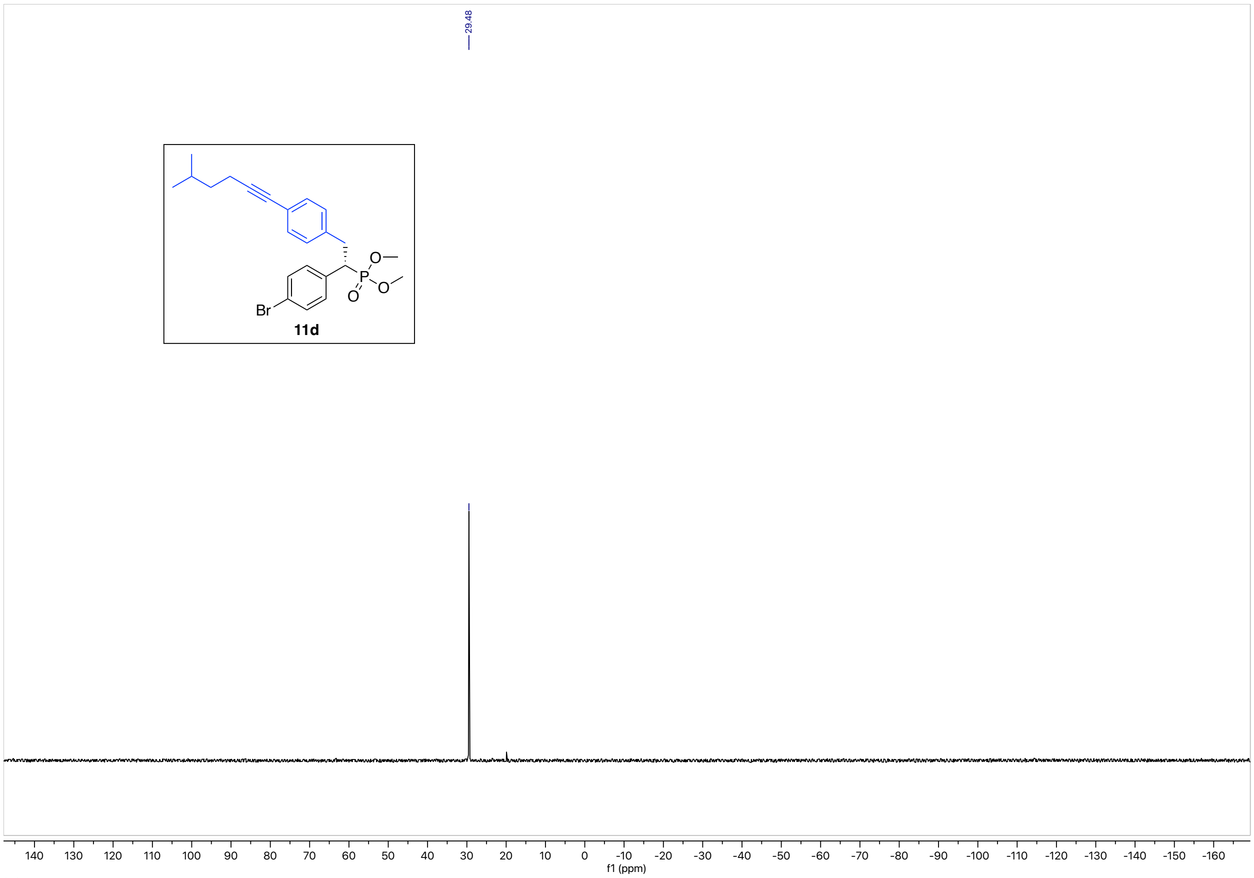

^{31}P NMR spectrum (243 MHz, Chloroform-*d*) of **11d**.

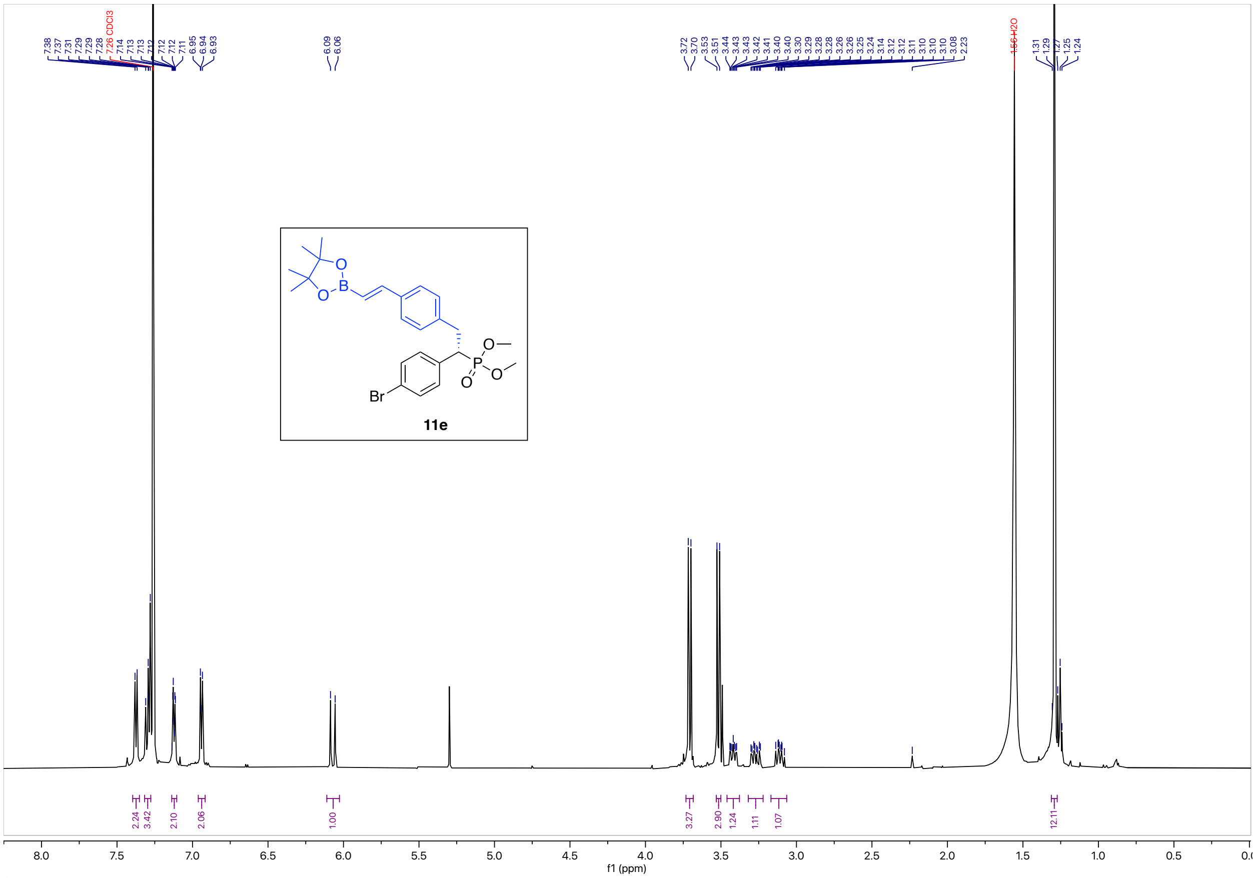

^{1}H NMR spectrum (600 MHz, Chloroform-*d*) (s, 7.26 ppm) of **11e**.

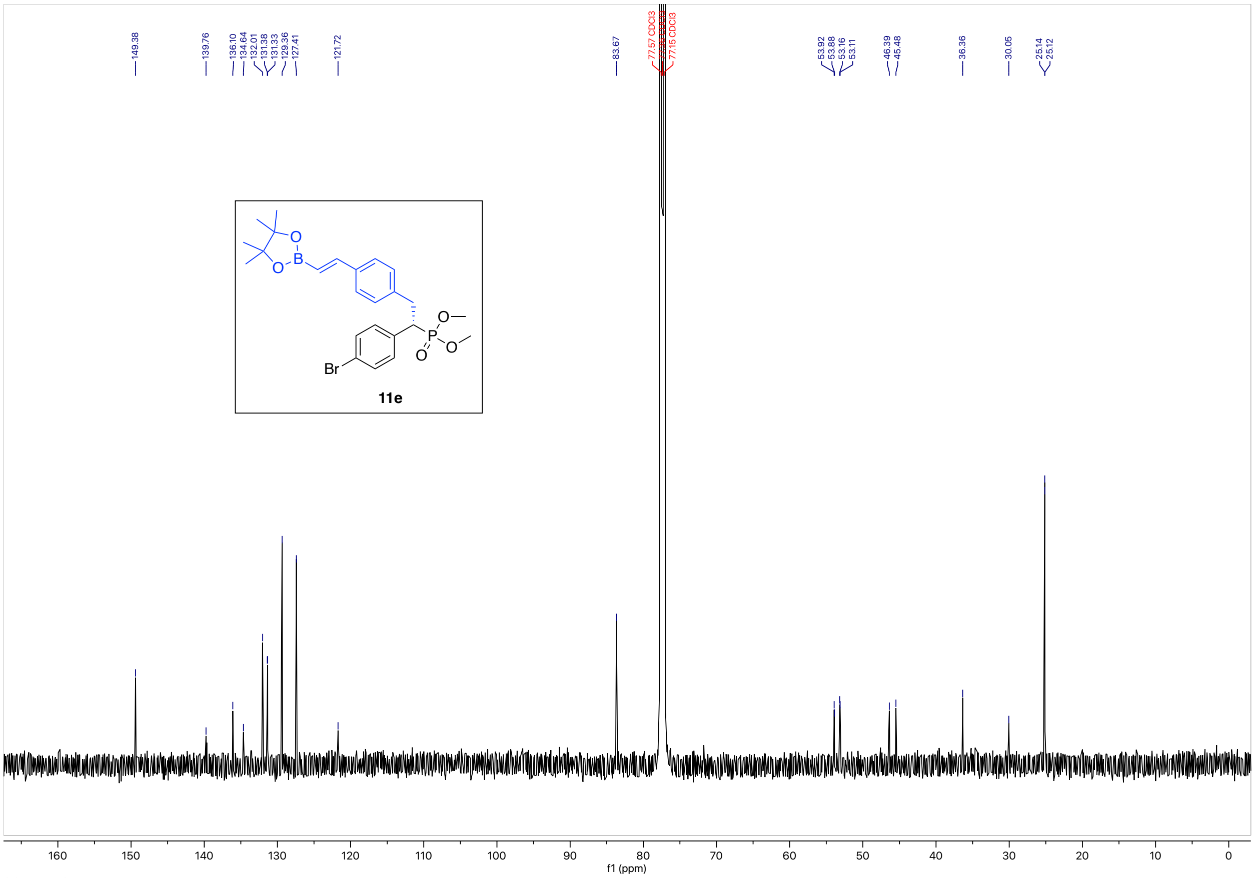

$^{13}C\{^{1}H\}$ NMR spectrum (151 MHz, Chloroform-*d*) (t, 77.36 ppm) of **11e**.

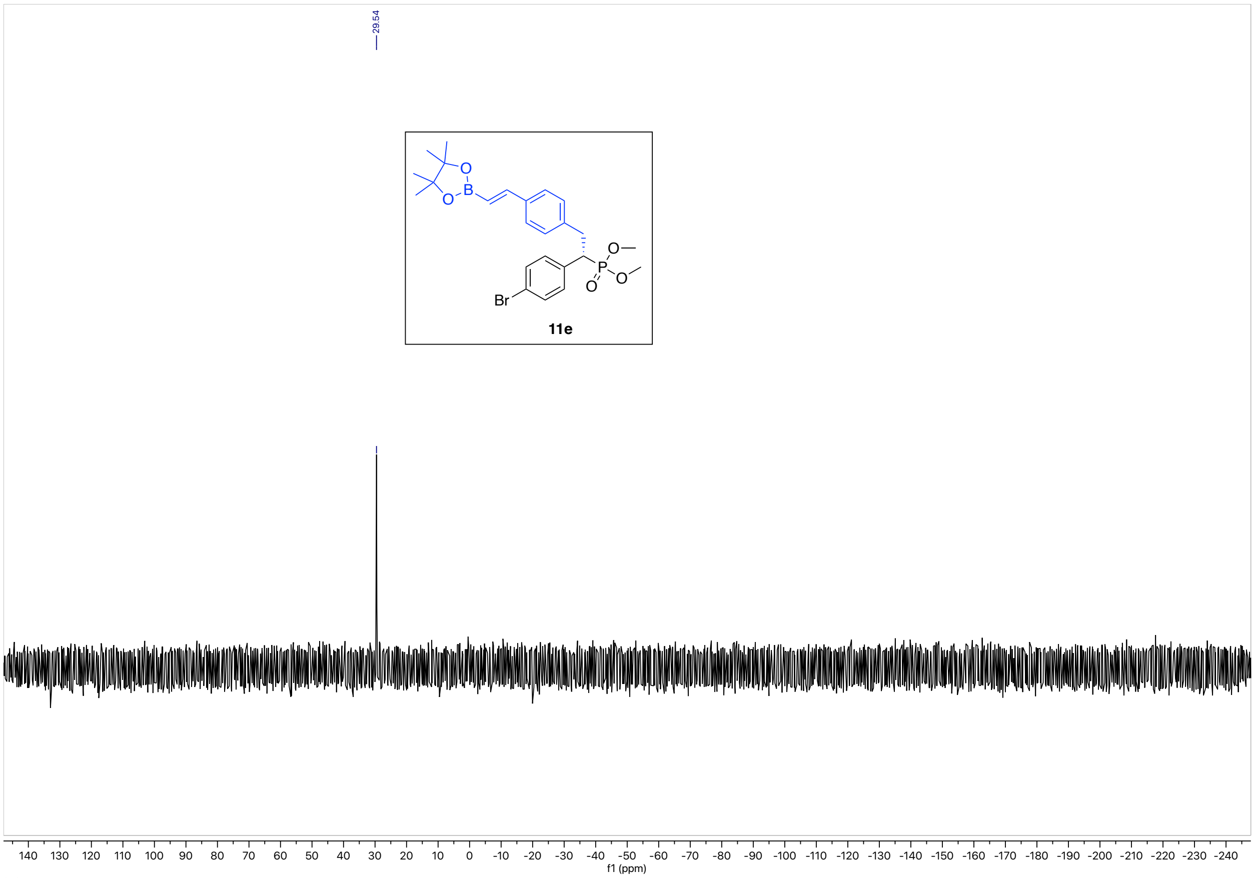

^{31}P NMR spectrum (243 MHz, Chloroform-*d*) of **11e**.

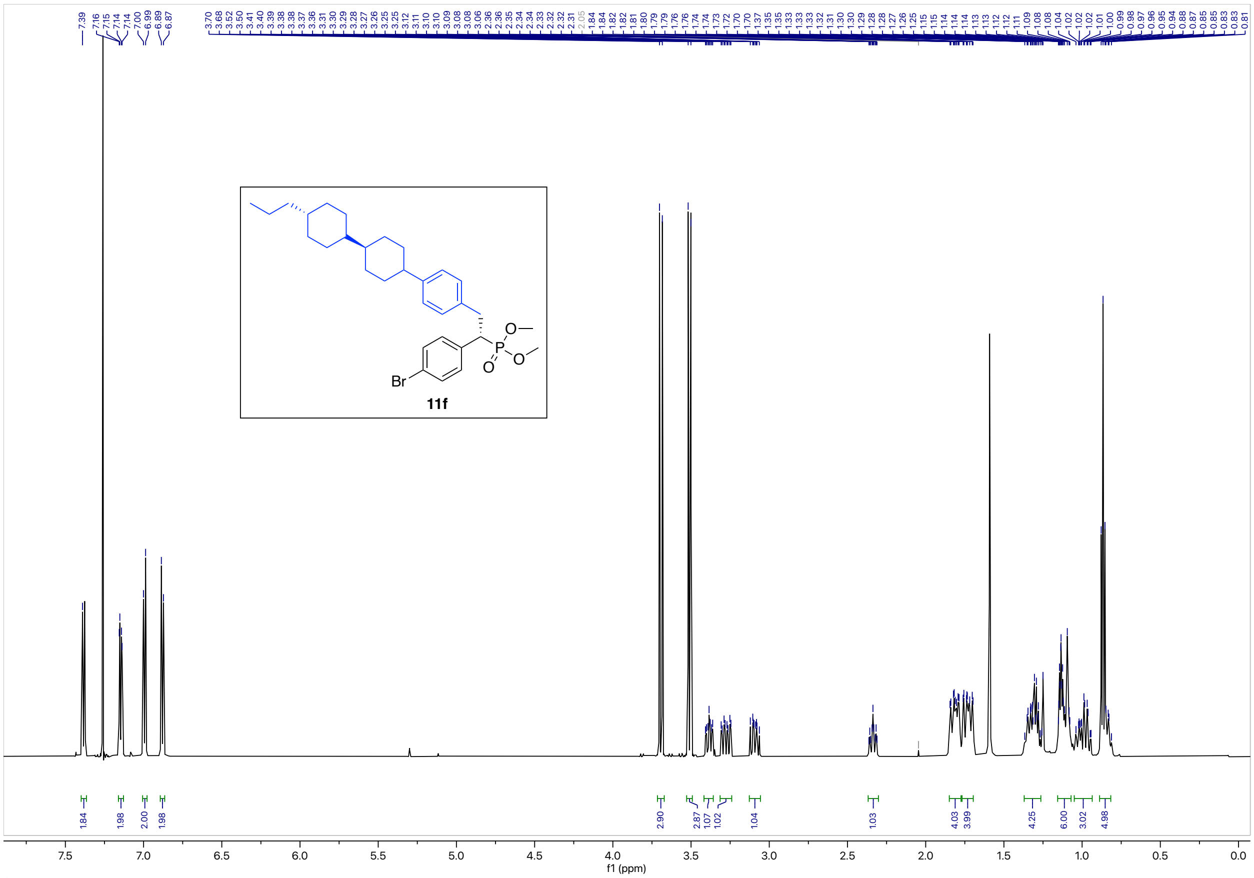

^{1}H NMR spectrum (600 MHz, Chloroform-*d*) (s, 7.26 ppm) of **11f**.

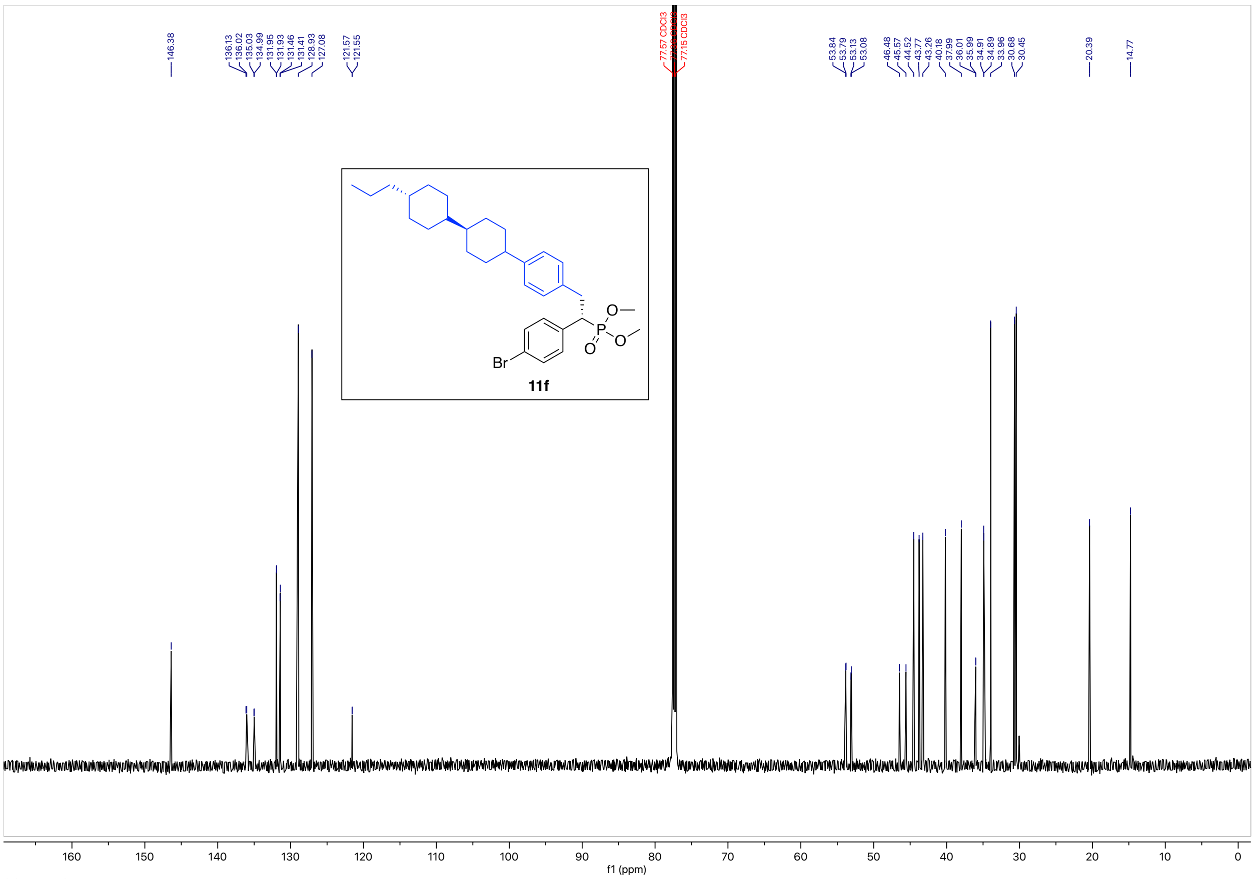

$^{13}C\{^{1}H\}$ NMR spectrum (151 MHz, Chloroform-*d*) (t, 77.36 ppm) of **11f**.

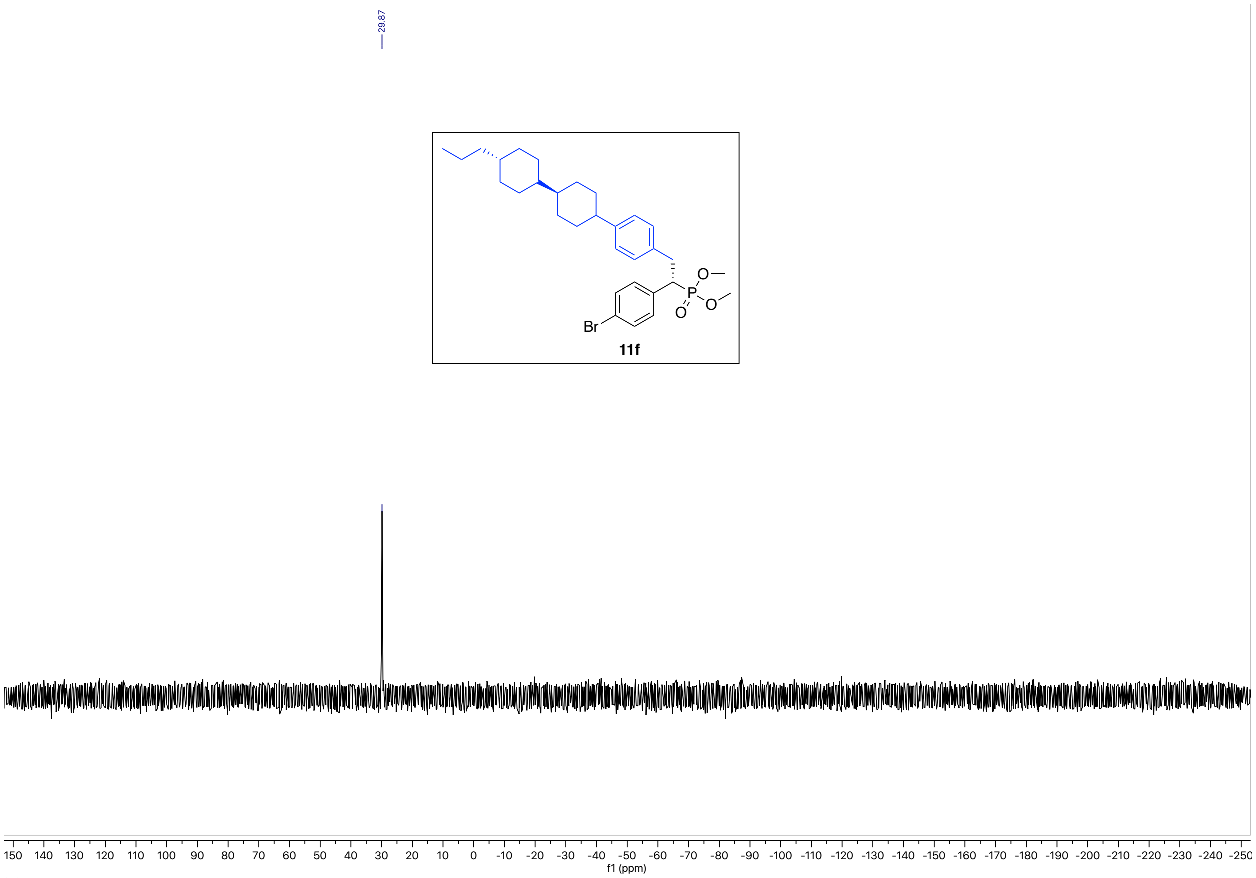

^{31}P NMR spectrum (243 MHz, Chloroform-*d*) of **11f**.

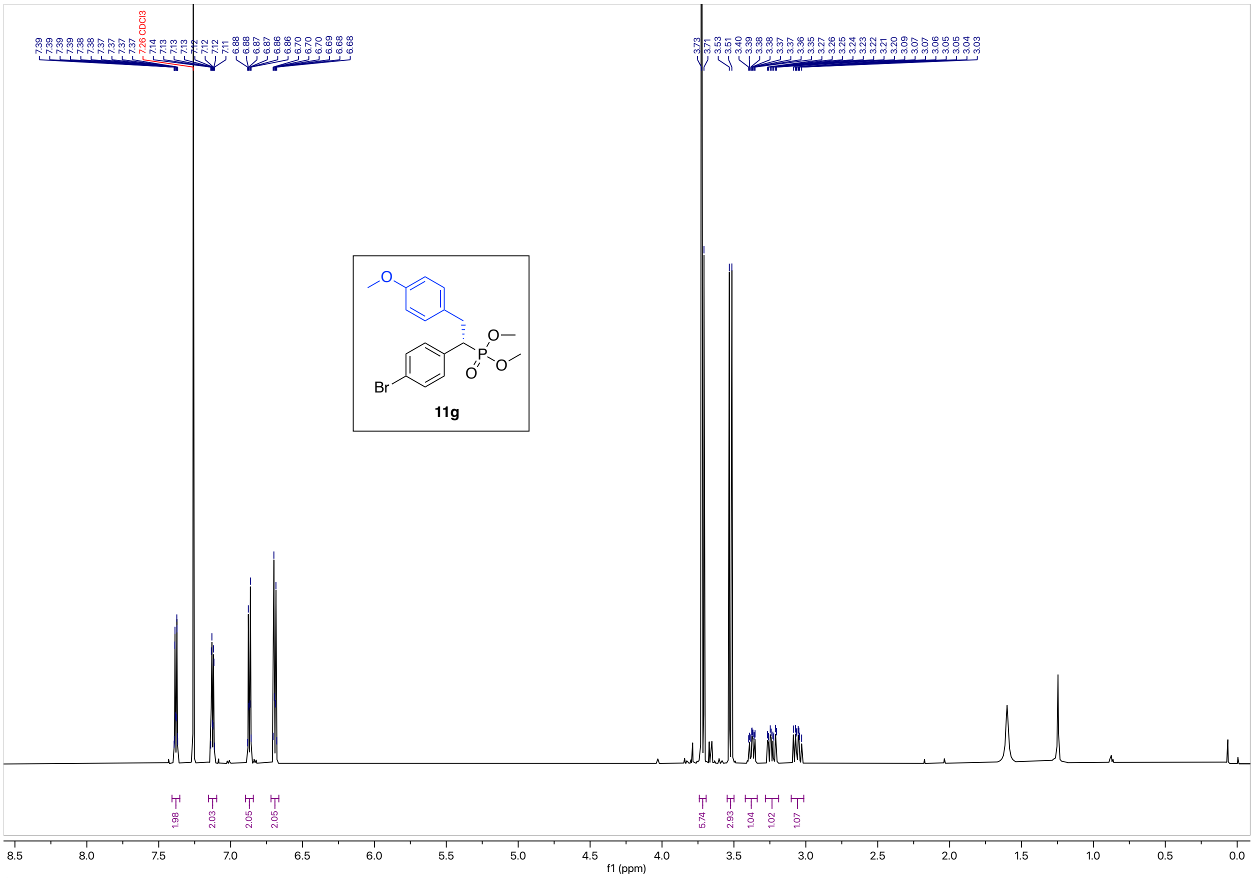

^{1}H NMR spectrum (600 MHz, Chloroform-*d*) (s, 7.26 ppm) of **11g**.

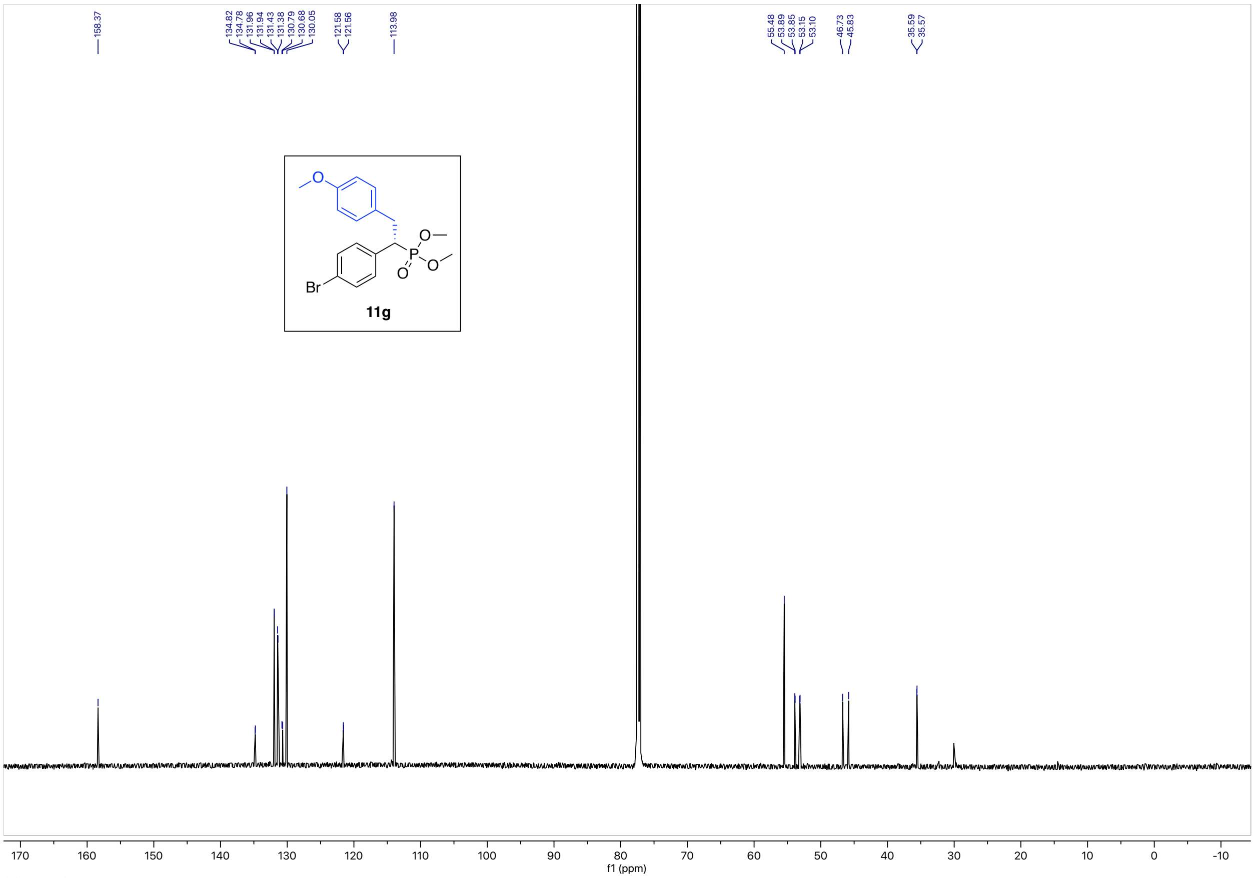

$^{13}C\{^1H\}$ NMR spectrum (151 MHz, Chloroform-*d*) (t, 77.36 ppm) of **11g**.

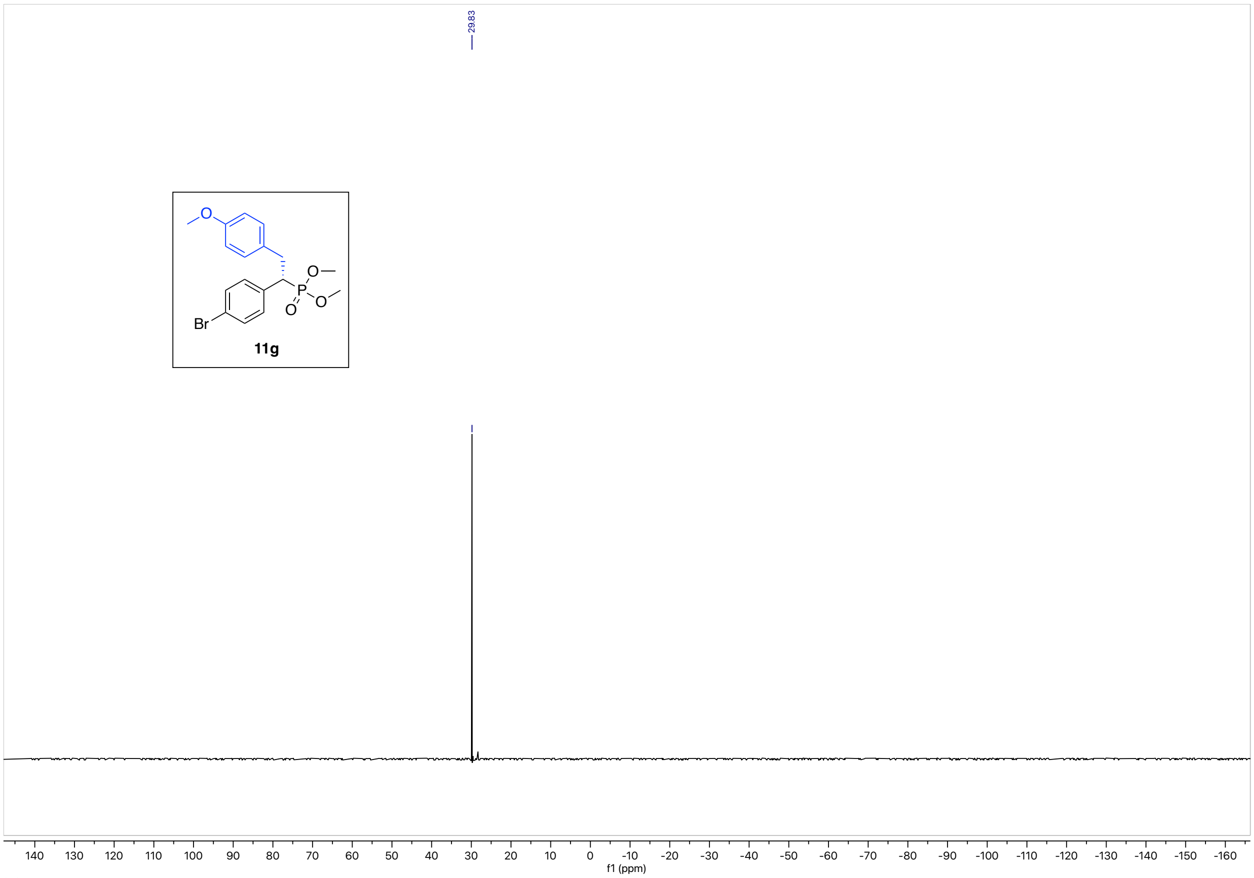

^{31}P NMR spectrum (243 MHz, Chloroform-*d*) of **11g**.

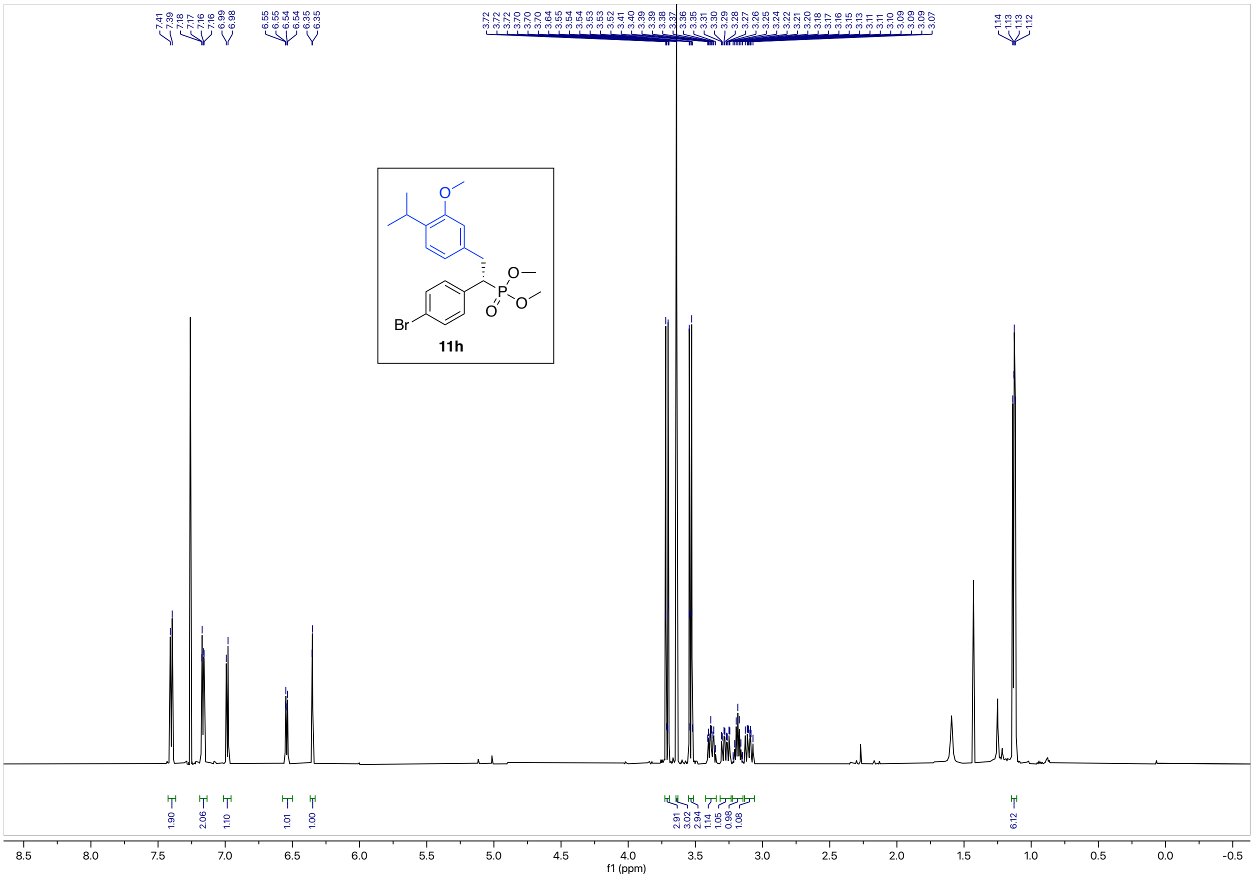

^{1}H NMR spectrum (600 MHz, Chloroform-*d*) (s, 7.26 ppm) of **11h**.

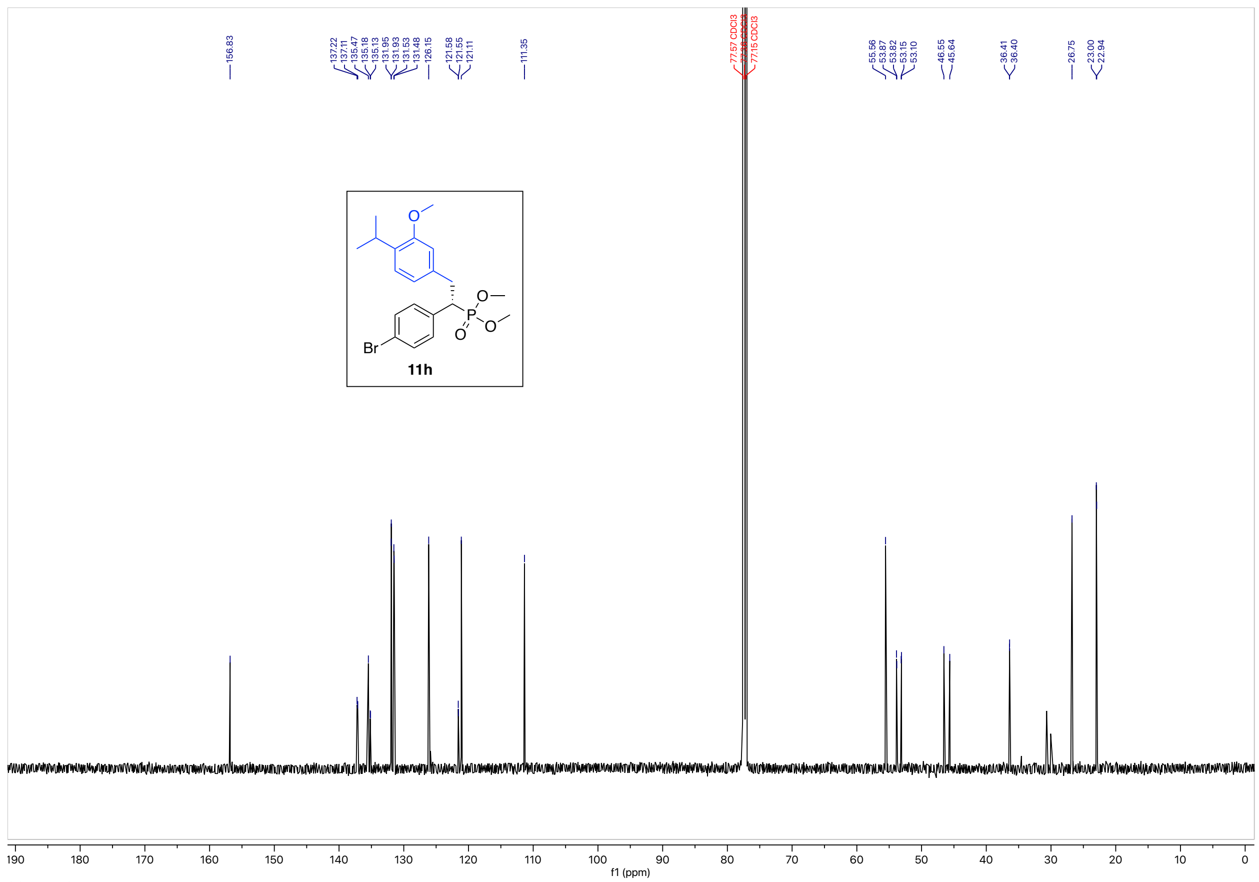

$^{13}C\{^{1}H\}$ NMR spectrum (151 MHz, Chloroform-*d*) (t, 77.36 ppm) of **11h**.

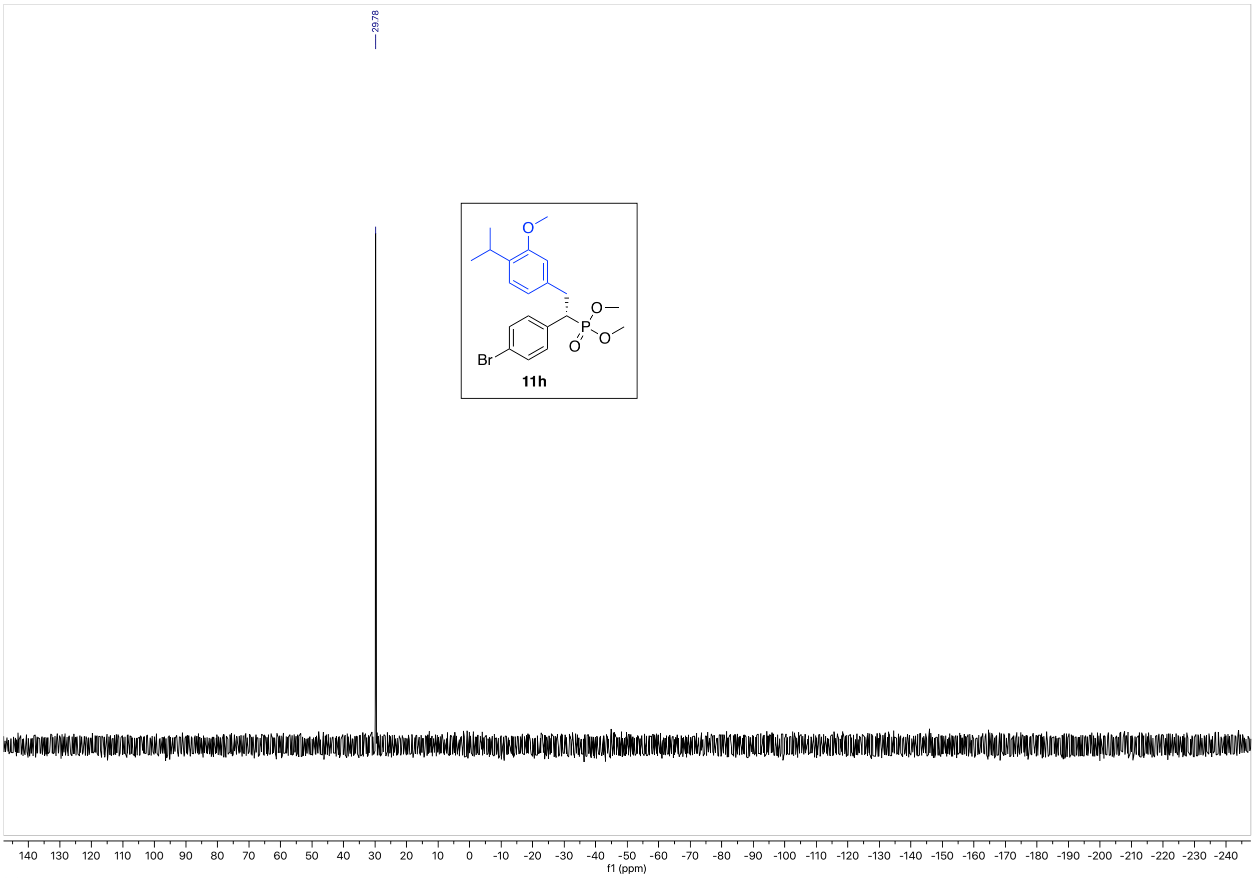

^{31}P NMR spectrum (243 MHz, Chloroform-*d*) of **11h**.

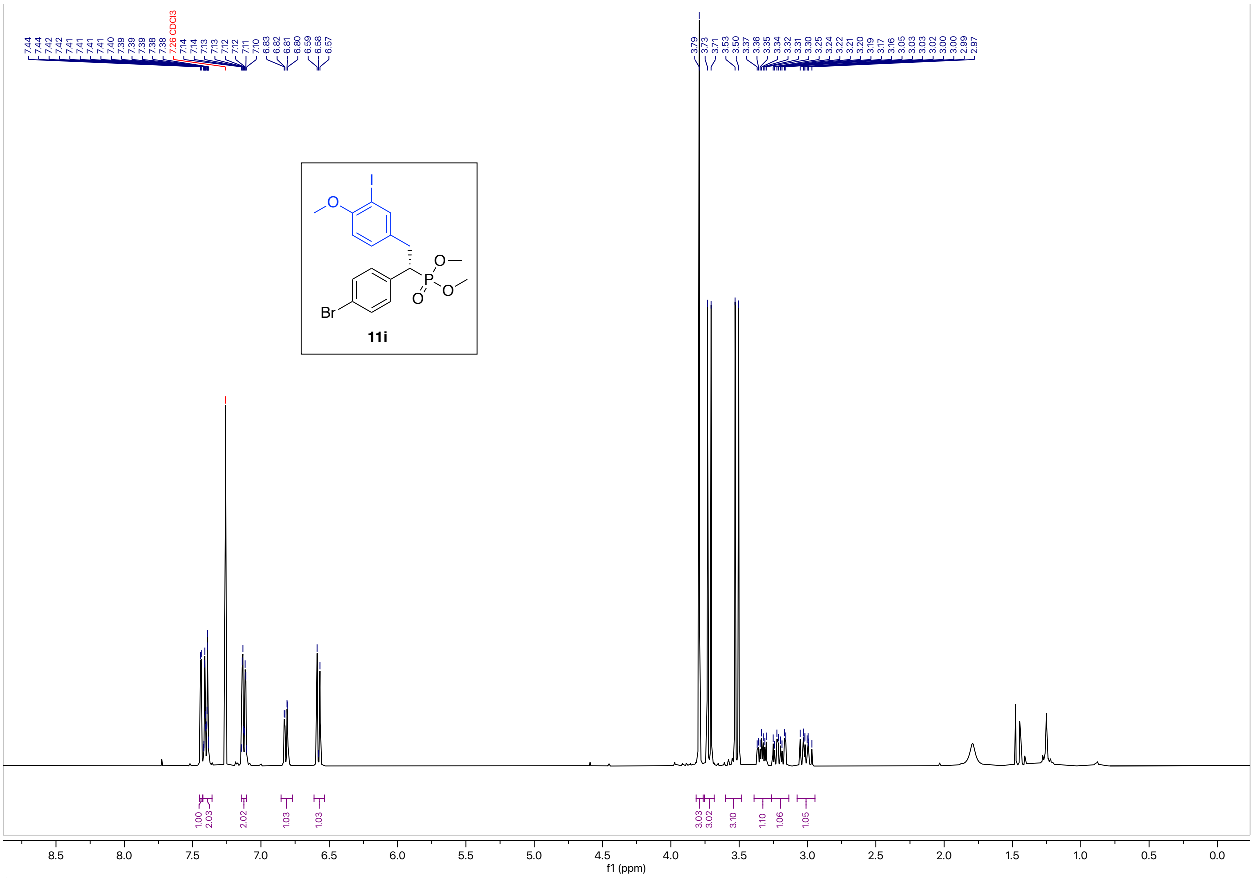

^{1}H NMR spectrum (400 MHz, Chloroform-*d*) (s, 7.26 ppm) of **11i**.

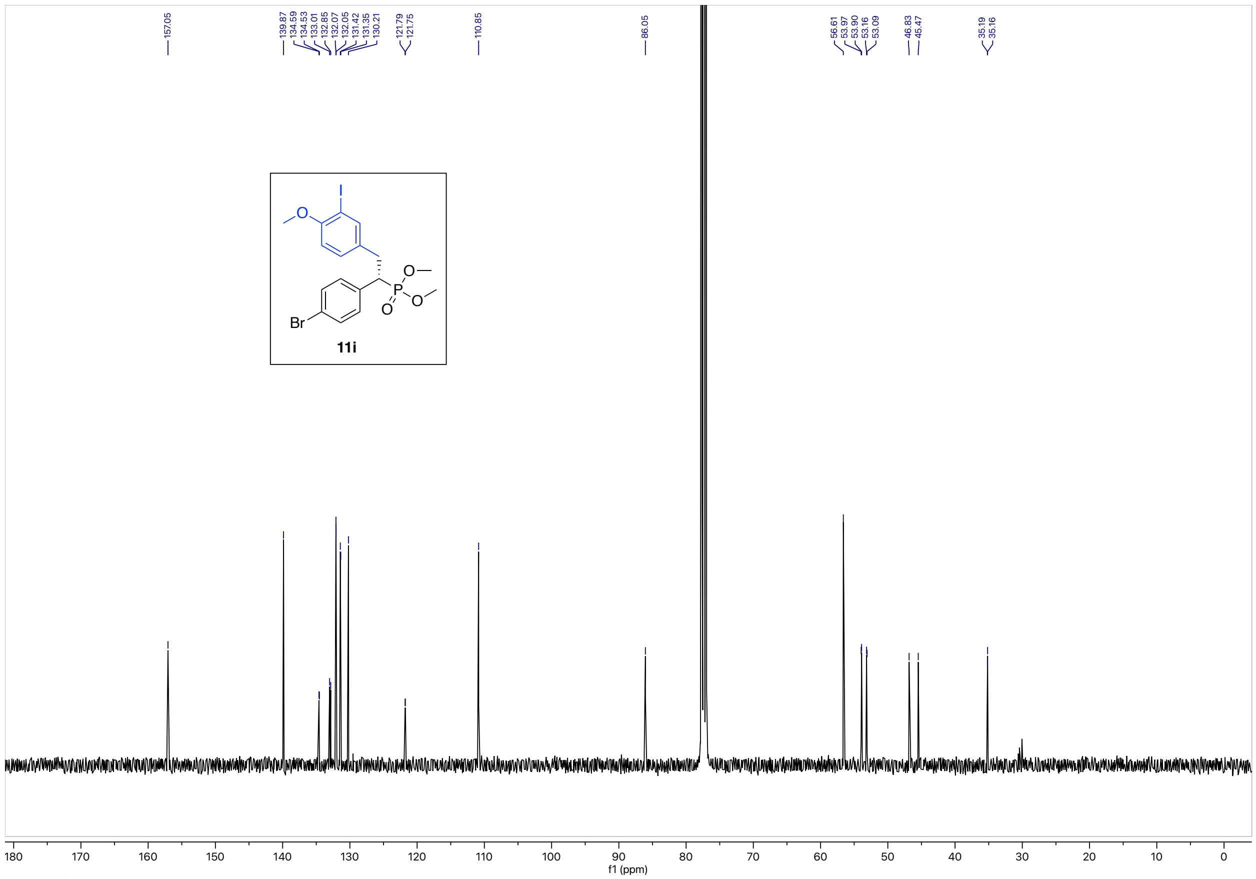

$^{13}C\{^{1}H\}$ NMR spectrum (101 MHz, Chloroform-*d*) (t, 77.36 ppm) of **11i**.

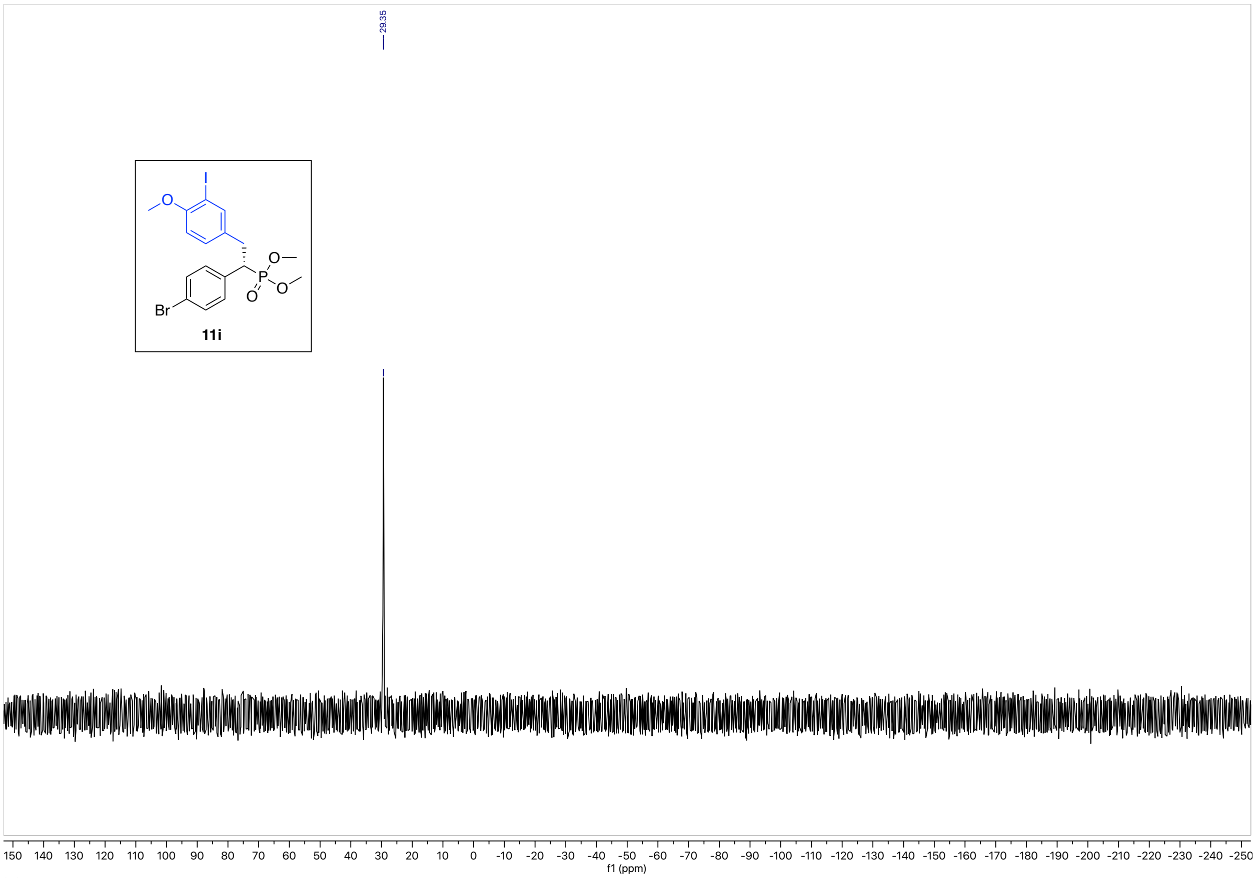

^{31}P NMR spectrum (162 MHz, Chloroform-*d*) of **11i**.

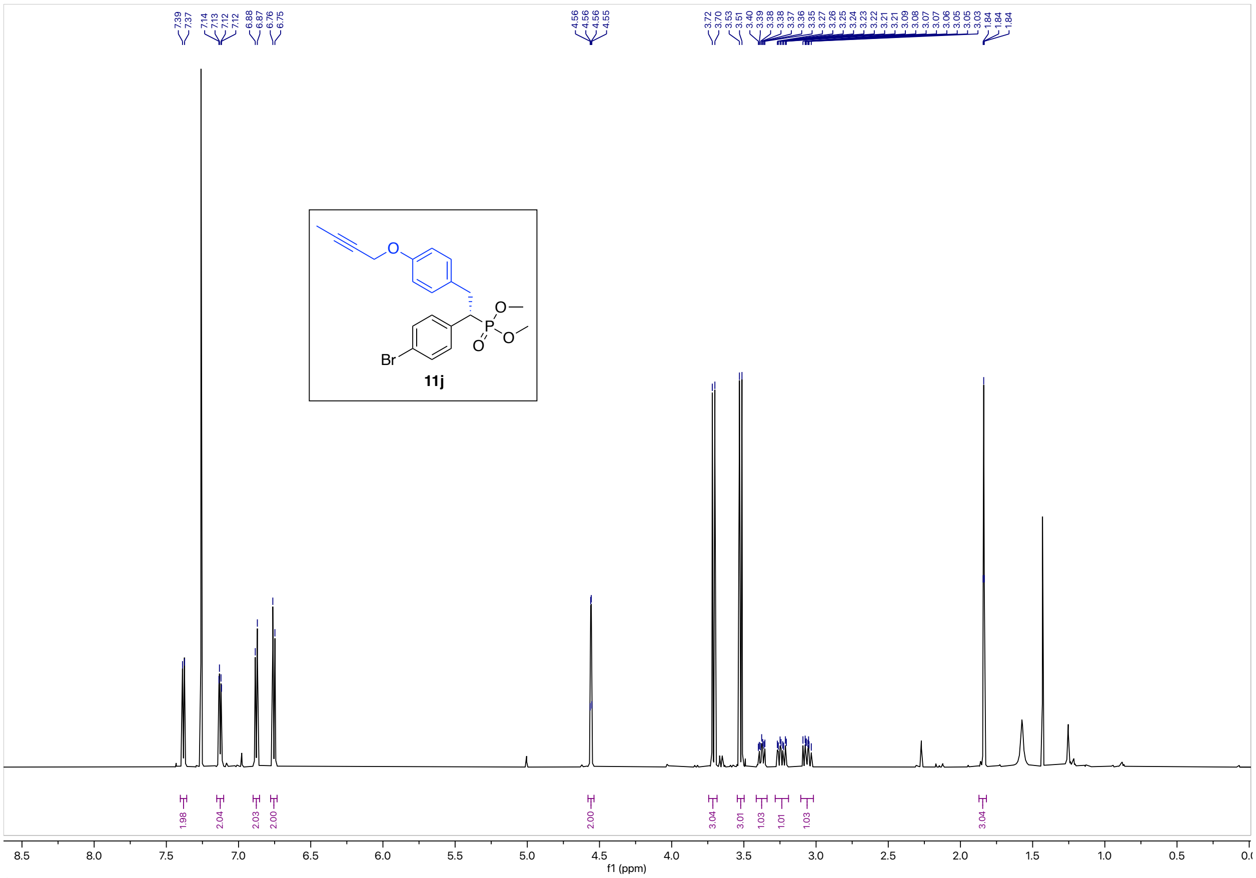

^{1}H NMR spectrum (600 MHz, Chloroform-*d*) (s, 7.26 ppm) of **11j**.

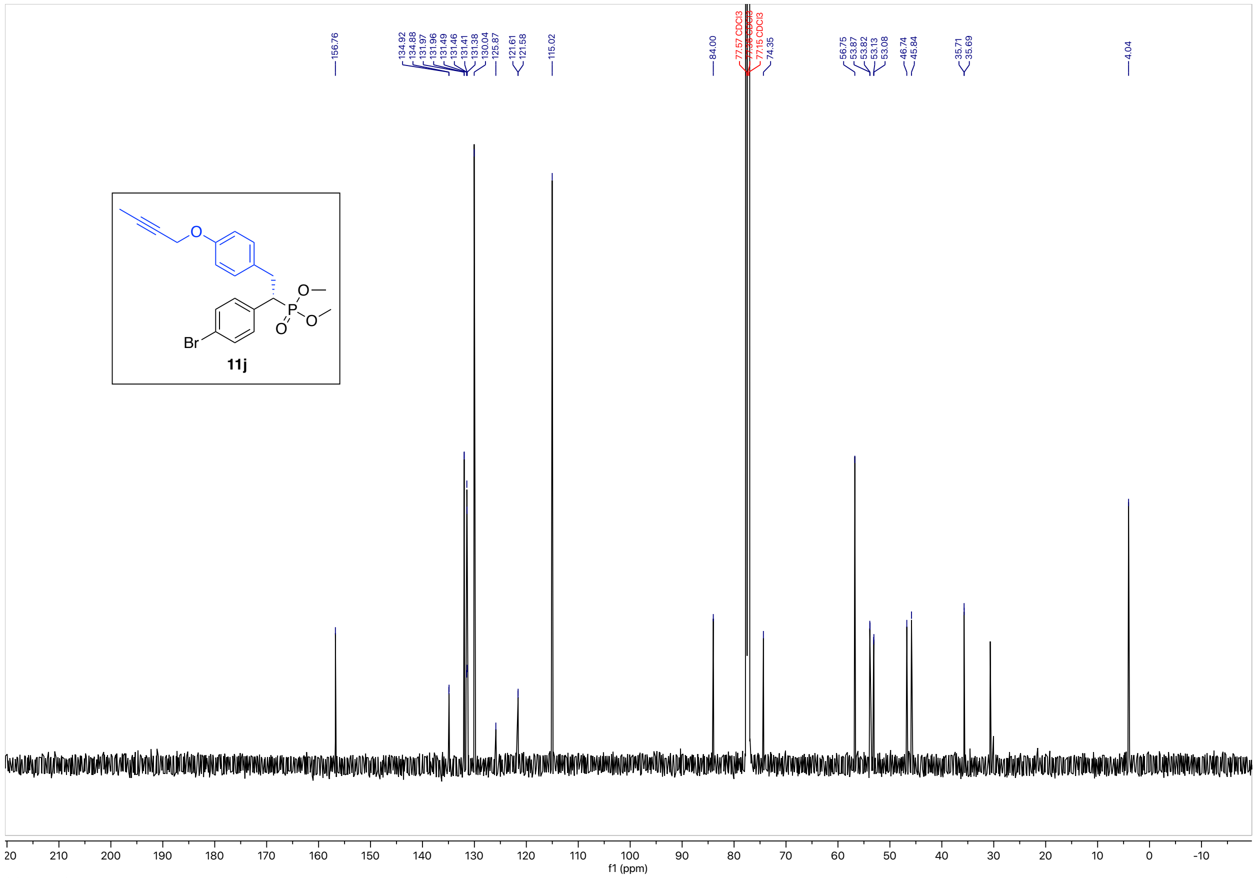

$^{13}C\{^{1}H\}$ NMR spectrum (151 MHz, Chloroform-*d*) (t, 77.36 ppm) of **11j**.

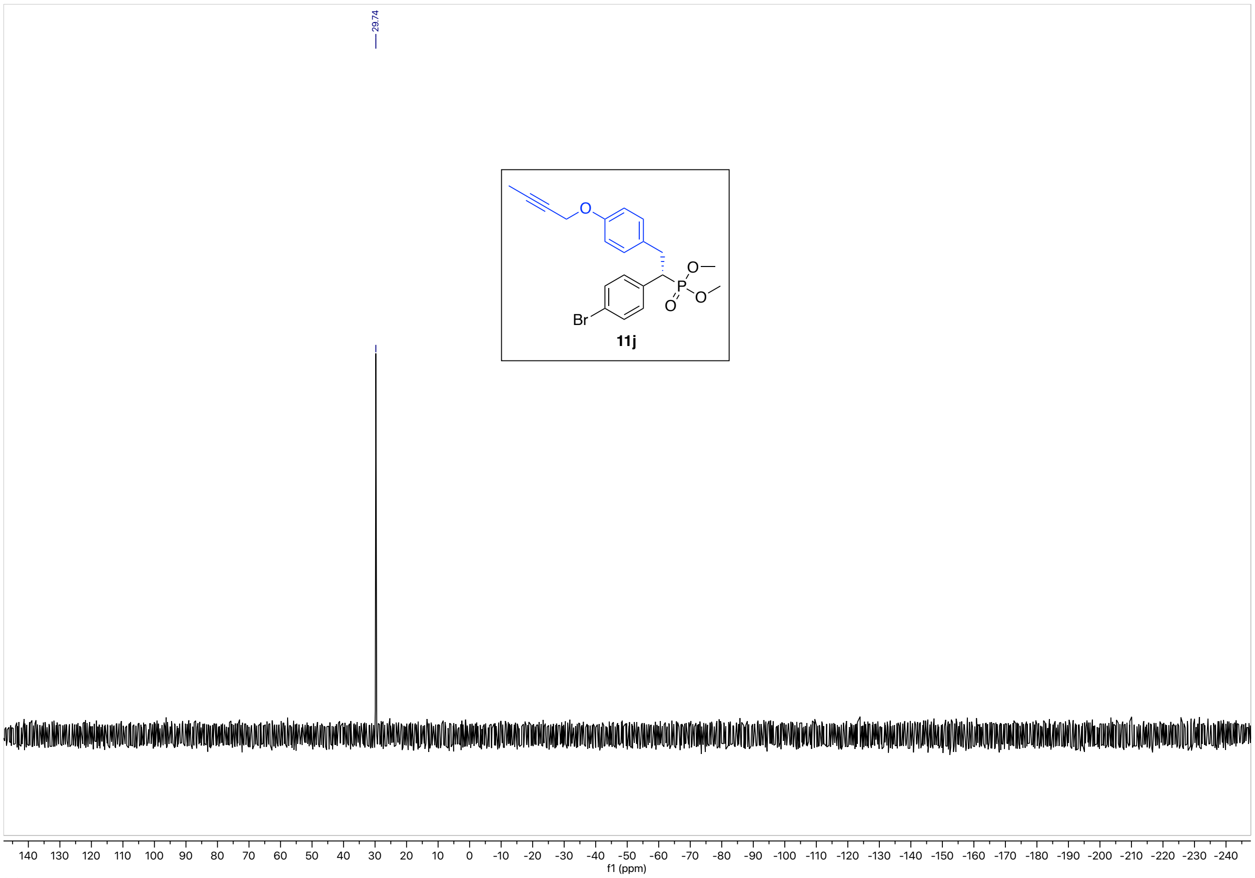

^{31}P NMR spectrum (243 MHz, Chloroform-*d*) of **11j**.

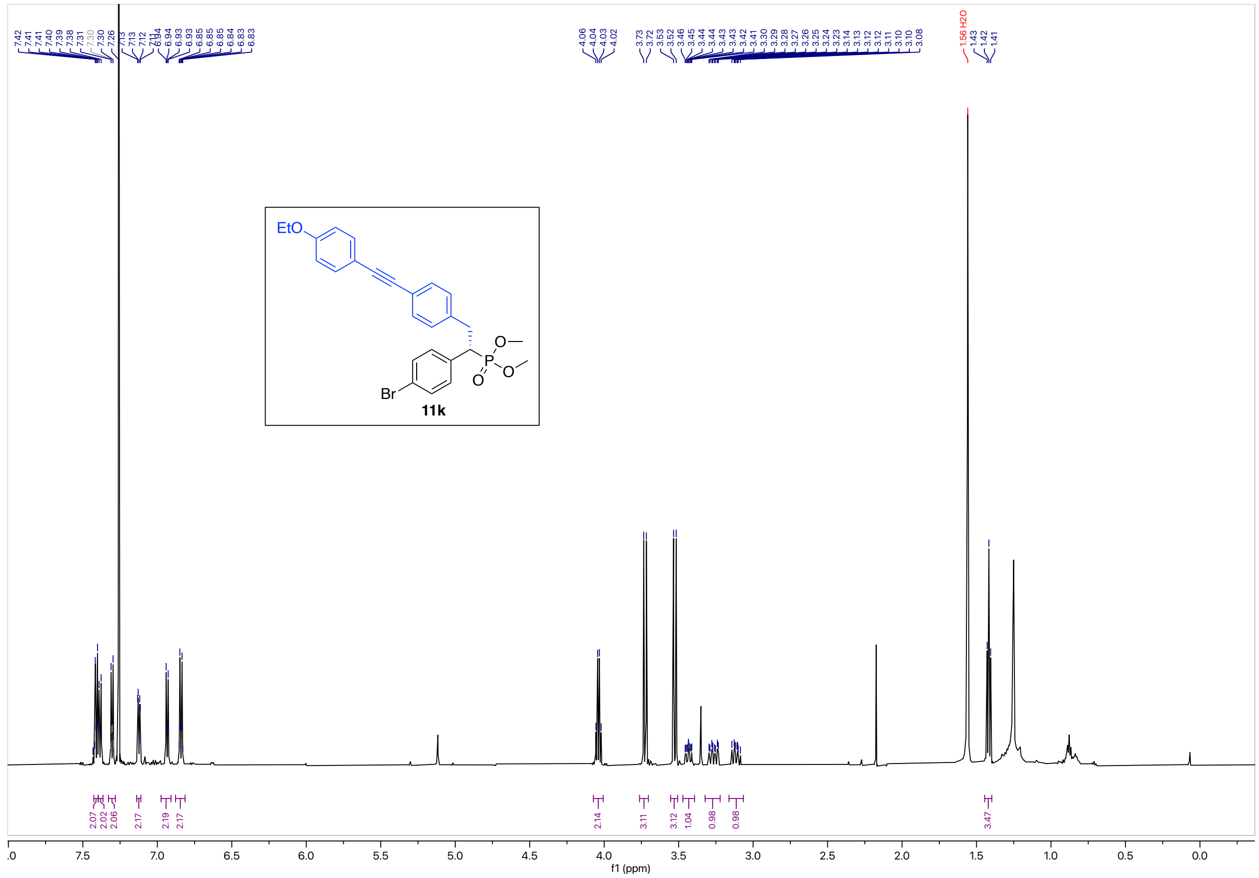

^{1}H NMR spectrum (600 MHz, Chloroform-*d*) (s, 7.26 ppm) of **11k**.

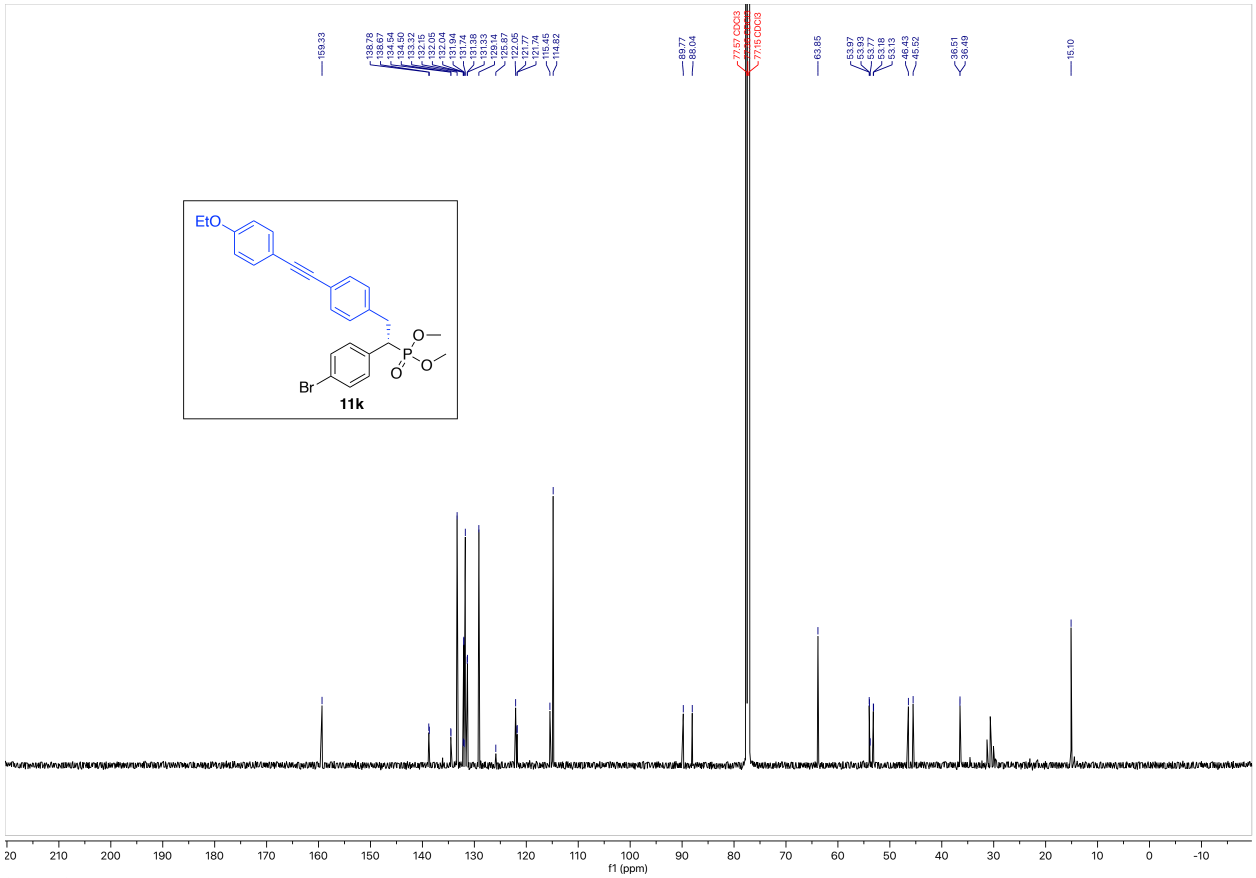

$^{13}C\{^{1}H\}$ NMR spectrum (151 MHz, Chloroform-*d*) (t, 77.36 ppm) of **11k**.

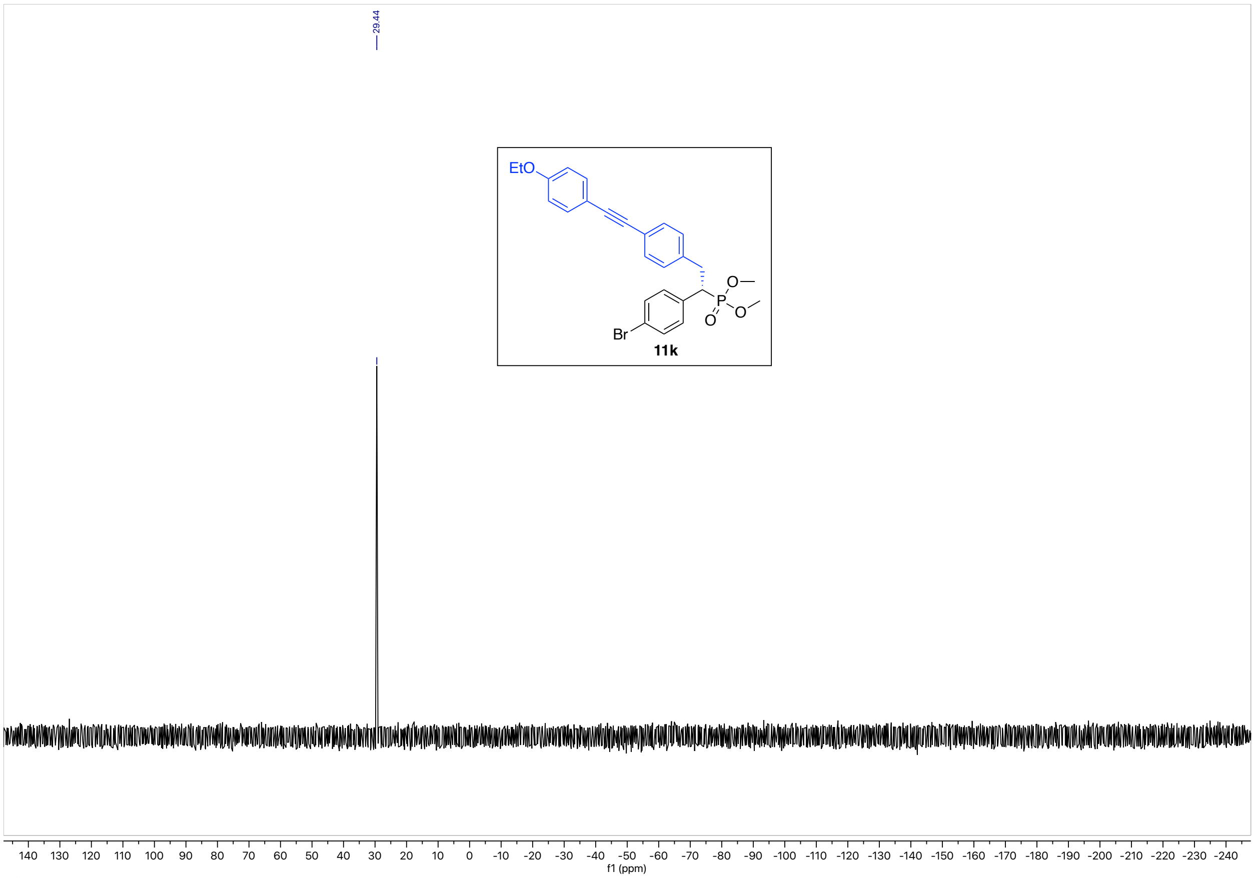

^{31}P NMR spectrum (243 MHz, Chloroform-*d*) of **11k**.

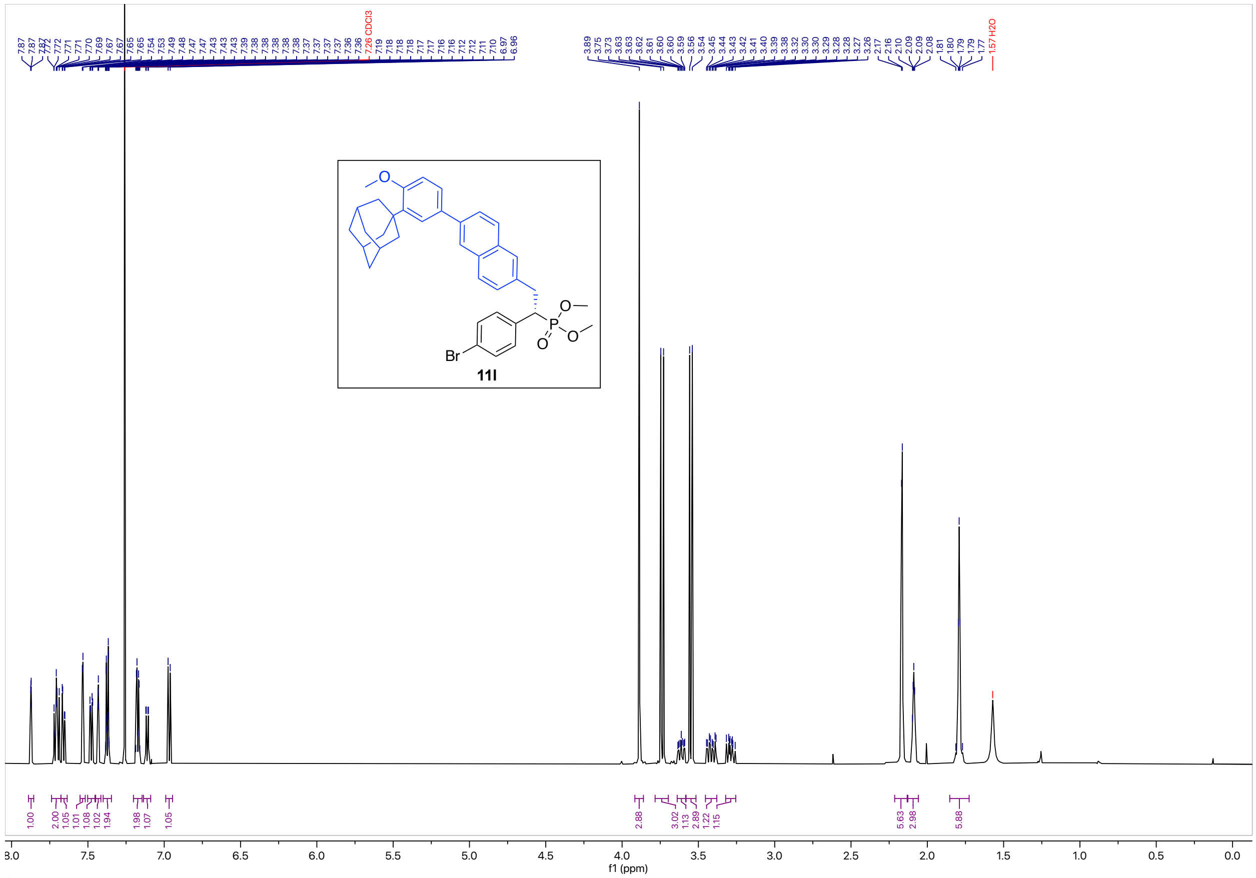

^{1}H NMR spectrum (600 MHz, Chloroform-*d*) (s, 7.26 ppm) of **11l**.

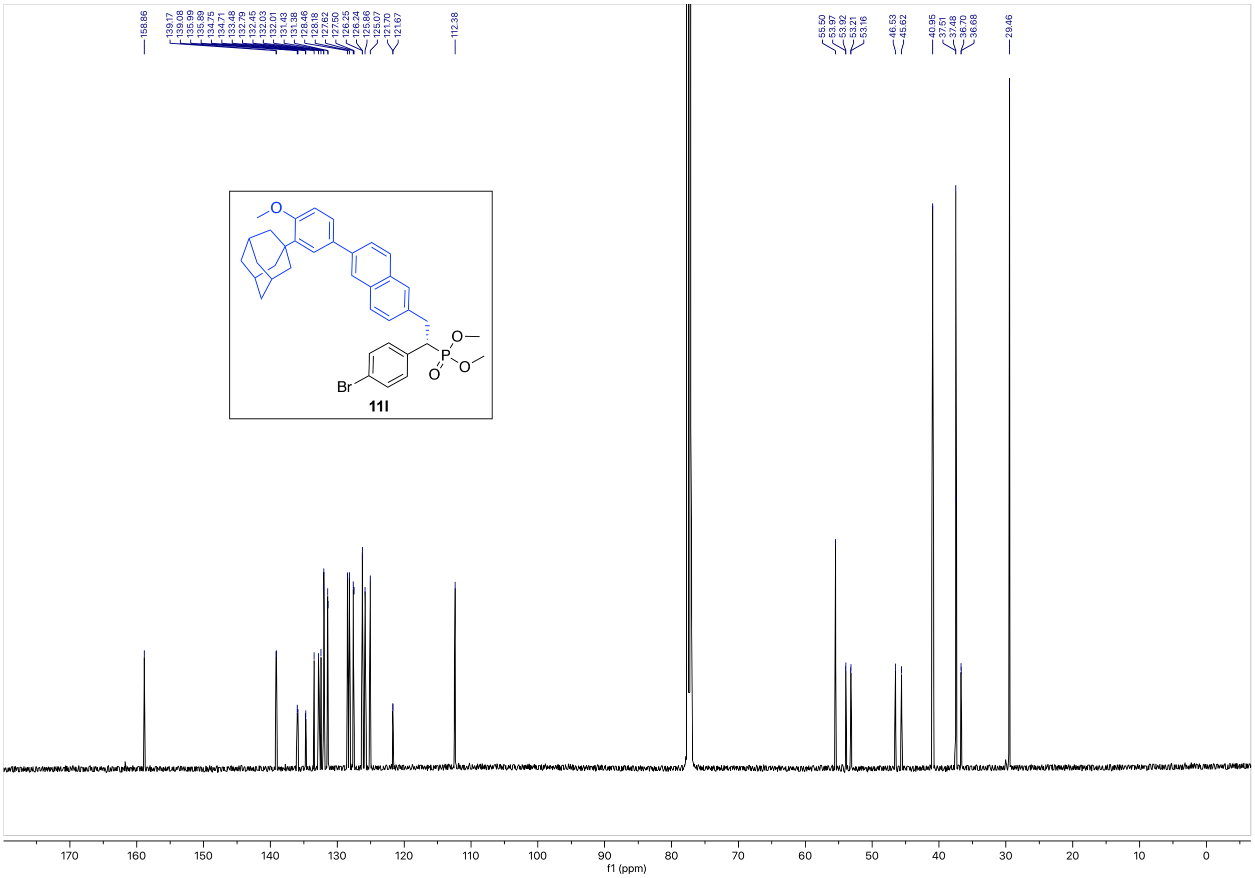

$^{13}C\{^{1}H\}$ NMR spectrum (151 MHz, Chloroform-*d*) (t, 77.36 ppm) of **11l**.

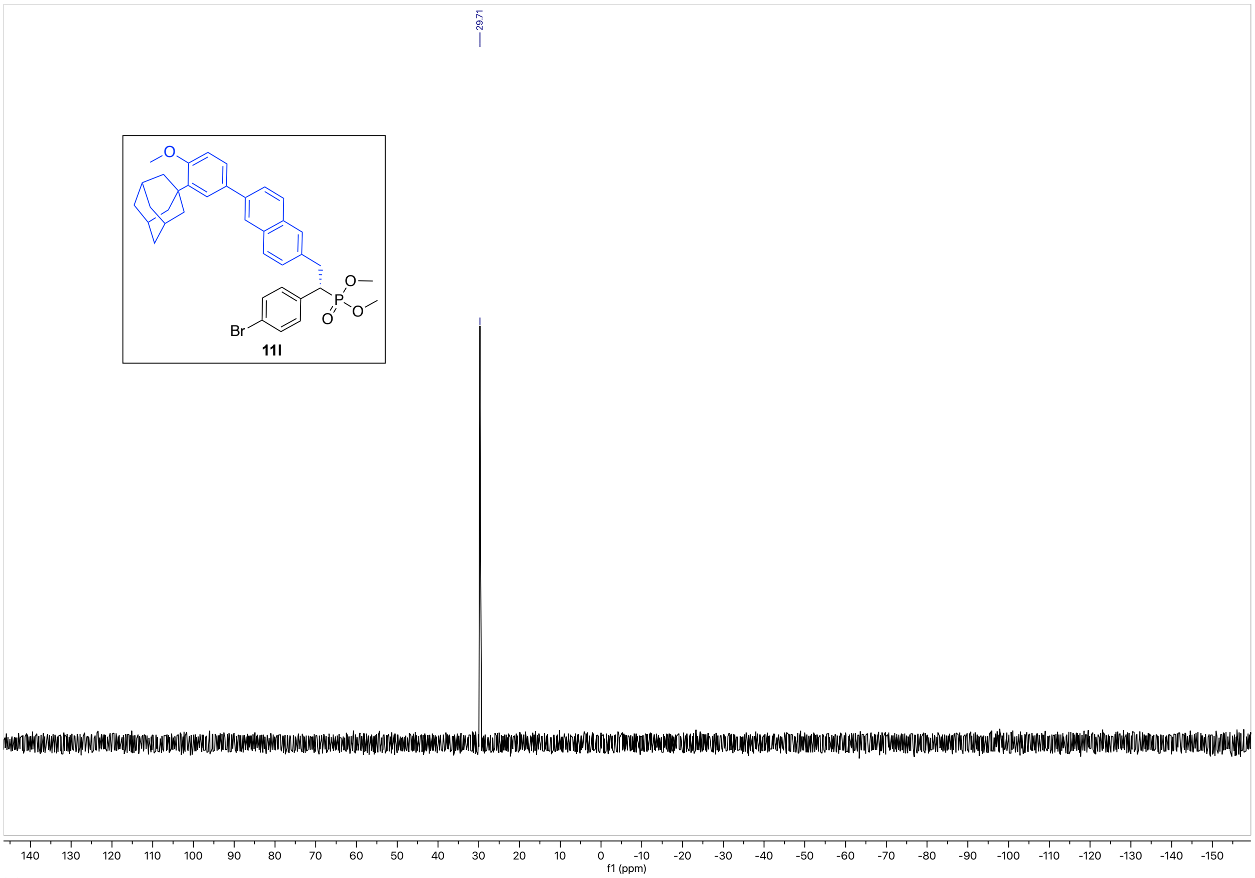

^{31}P NMR spectrum (243 MHz, Chloroform-*d*) of **11l**.

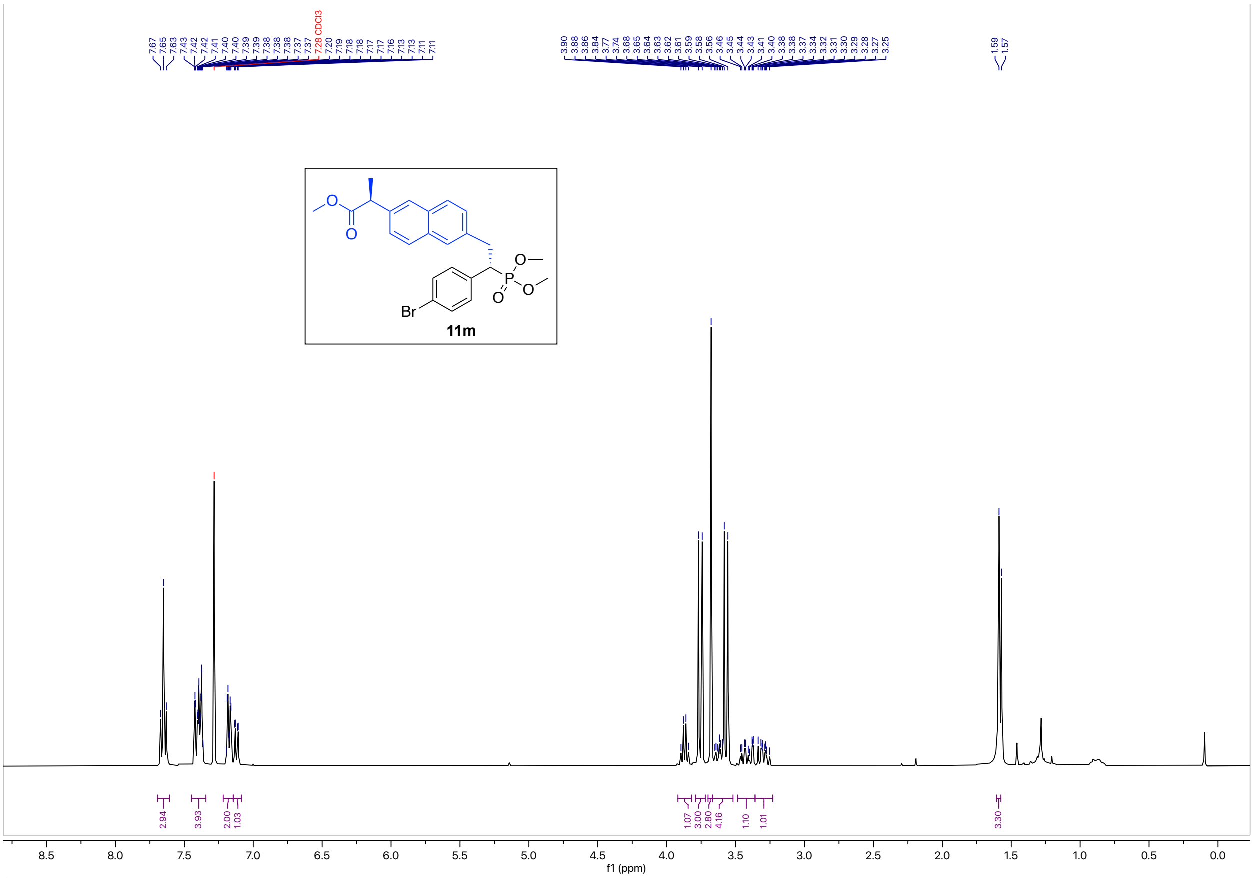

^{1}H NMR spectrum (400 MHz, Chloroform-*d*) (s, 7.26 ppm) of **11m**.

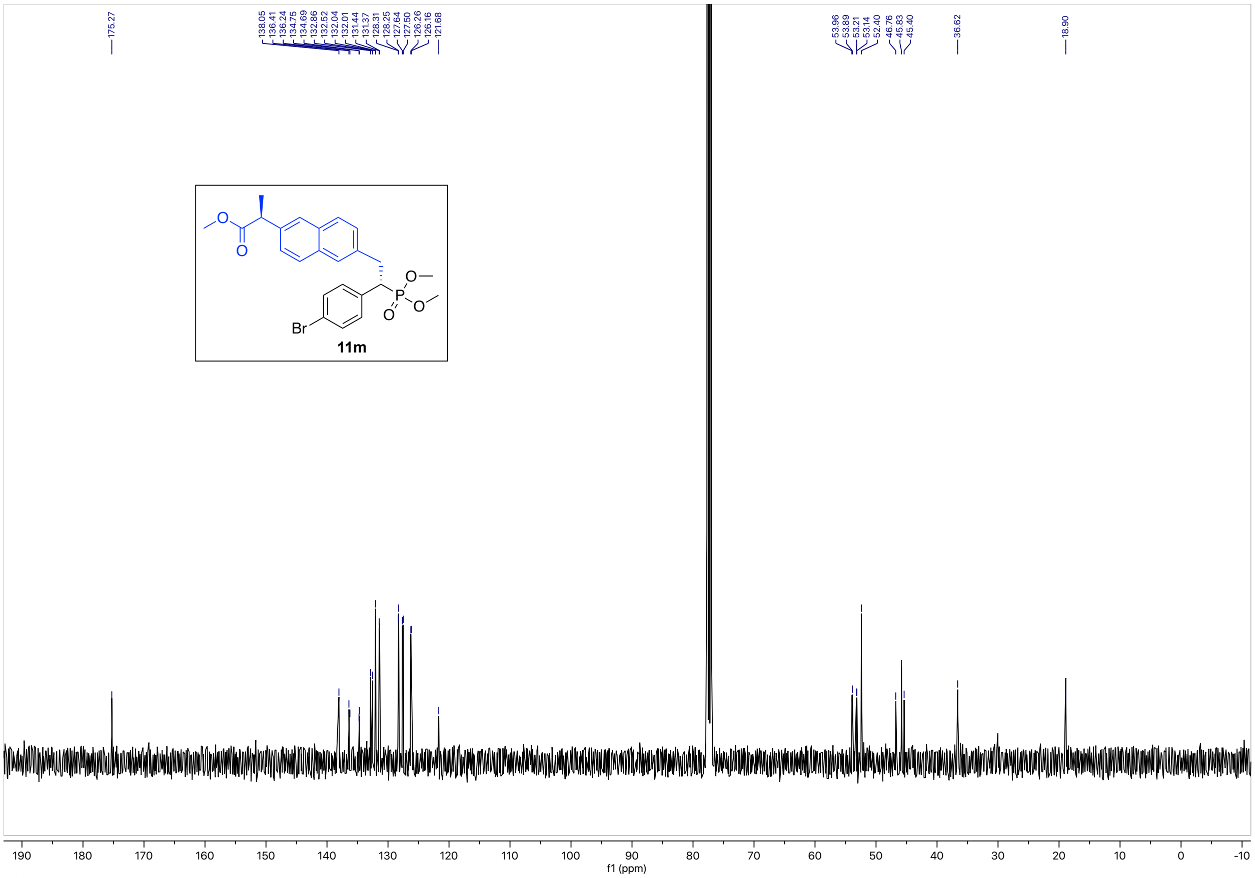

$^{13}C\{^1H\}$ NMR spectrum (101 MHz, Chloroform-*d*) (t, 77.36 ppm) of **11m**.

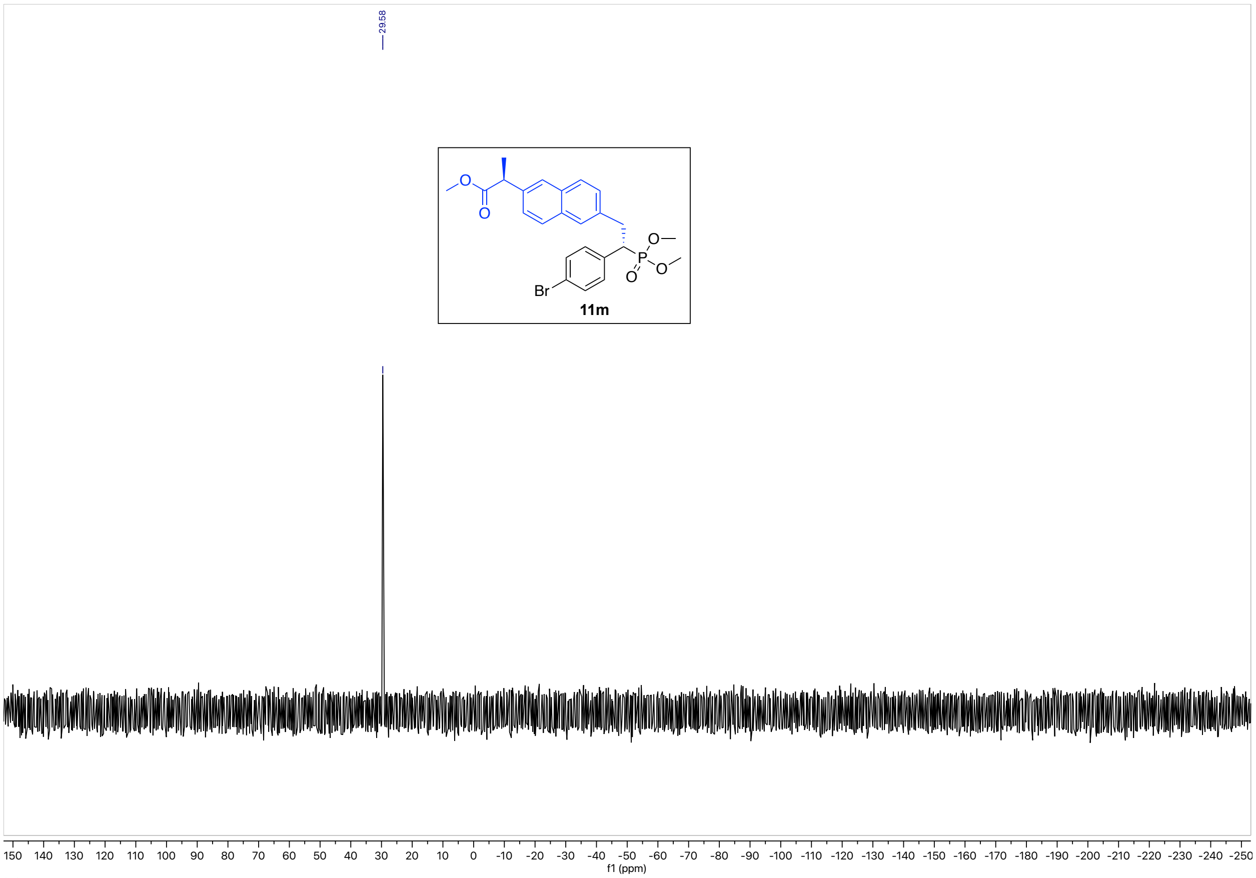

^{31}P NMR spectrum (162 MHz, Chloroform-*d*) of **11m**.

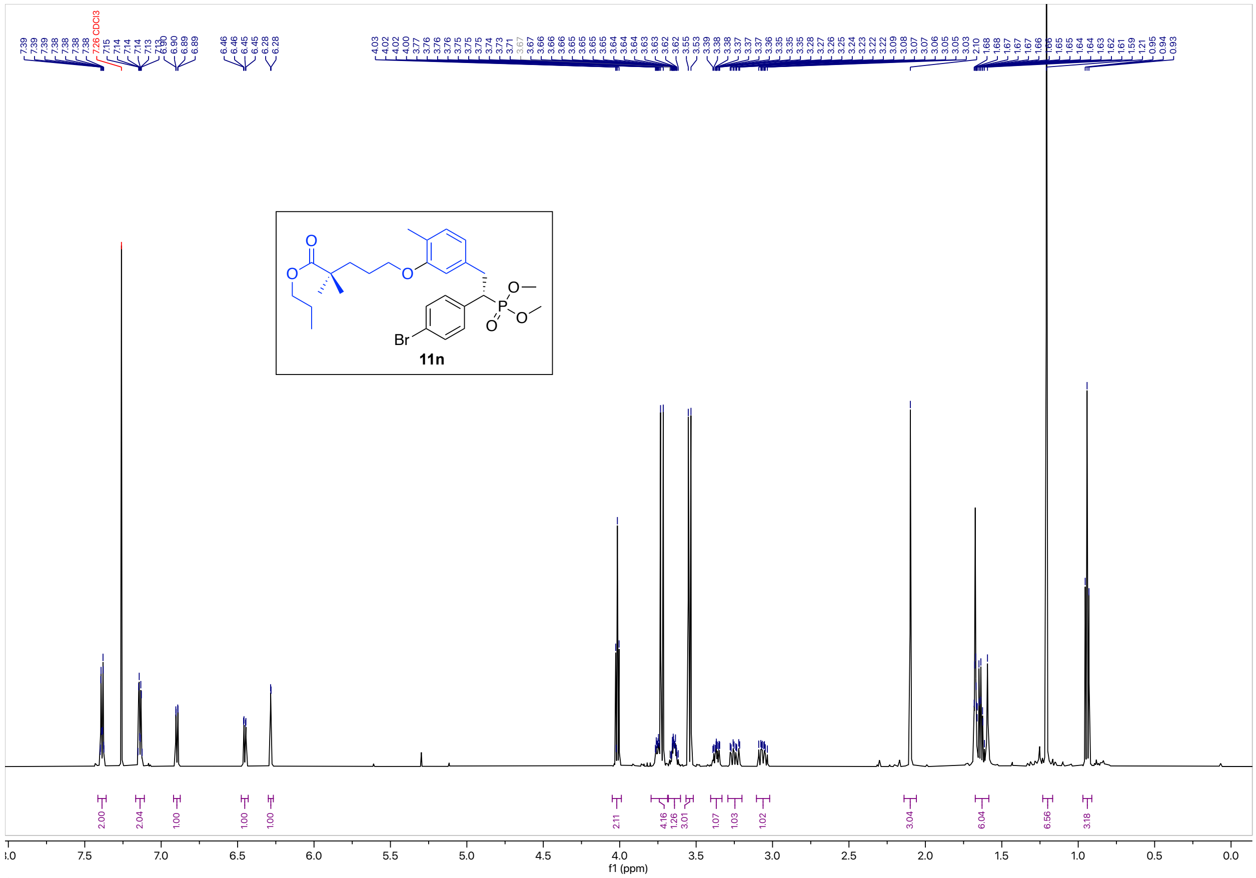

^{1}H NMR spectrum (600 MHz, Chloroform-*d*) (s, 7.26 ppm) of **11n**.

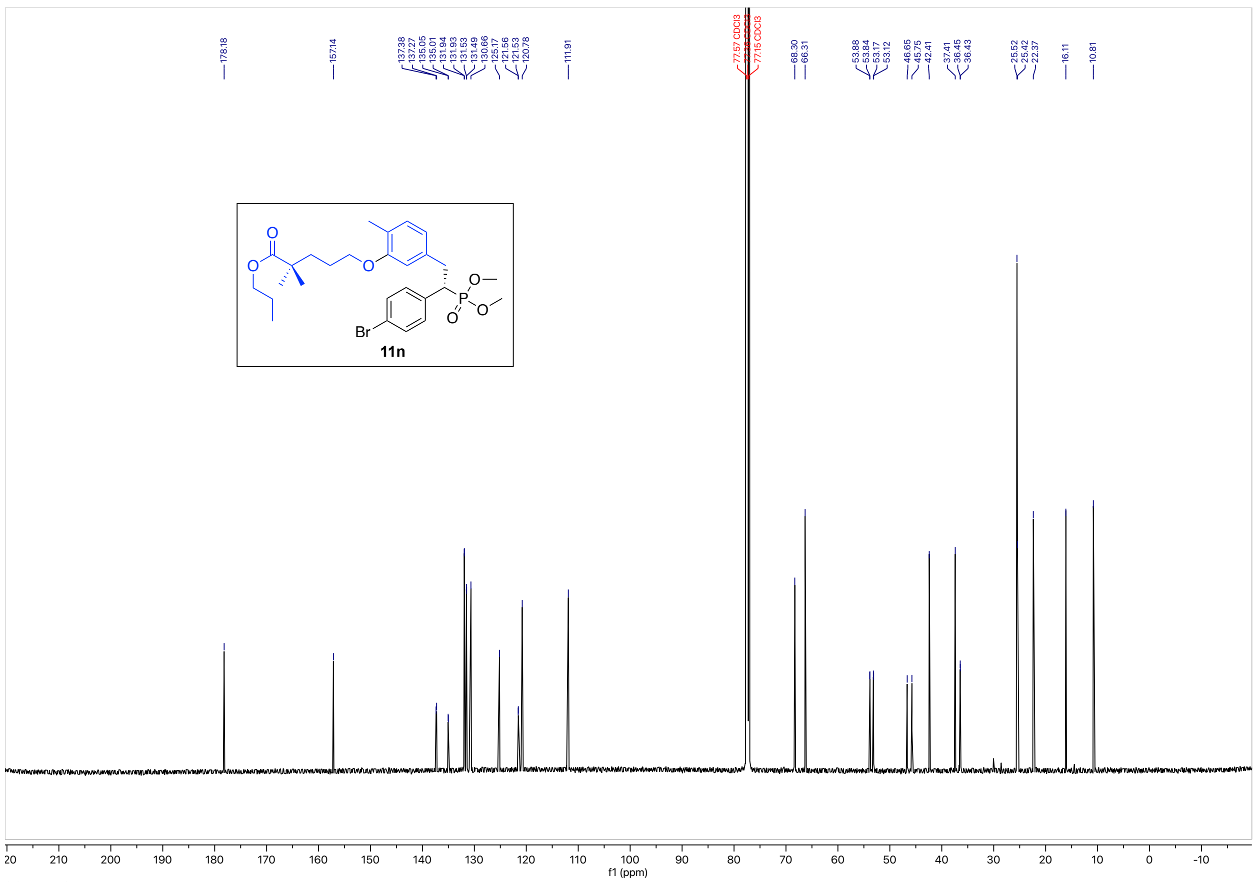

$^{13}C\{^{1}H\}$ NMR spectrum (151 MHz, Chloroform-*d*) (t, 77.36 ppm) of **11n**.

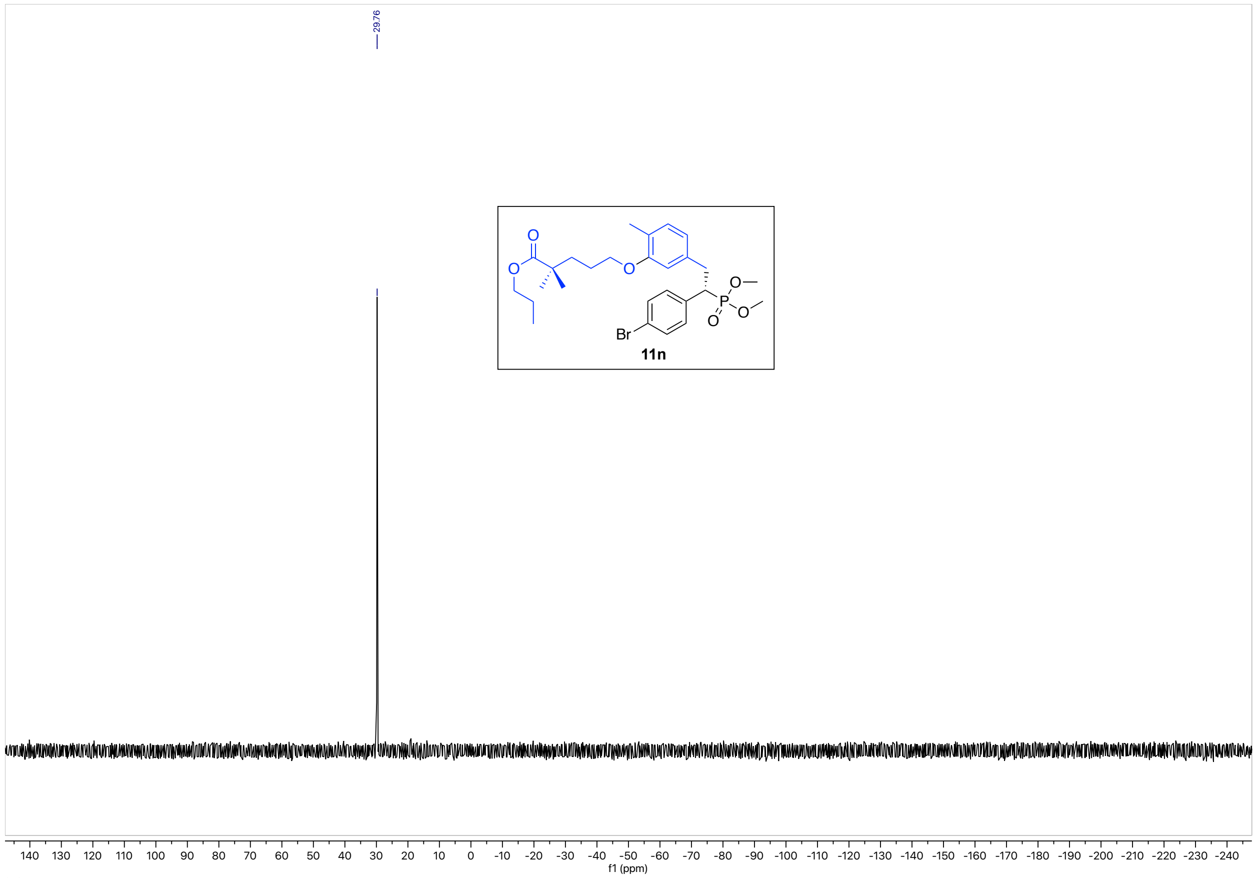

^{31}P NMR spectrum (243 MHz, Chloroform-*d*) of **11n**.

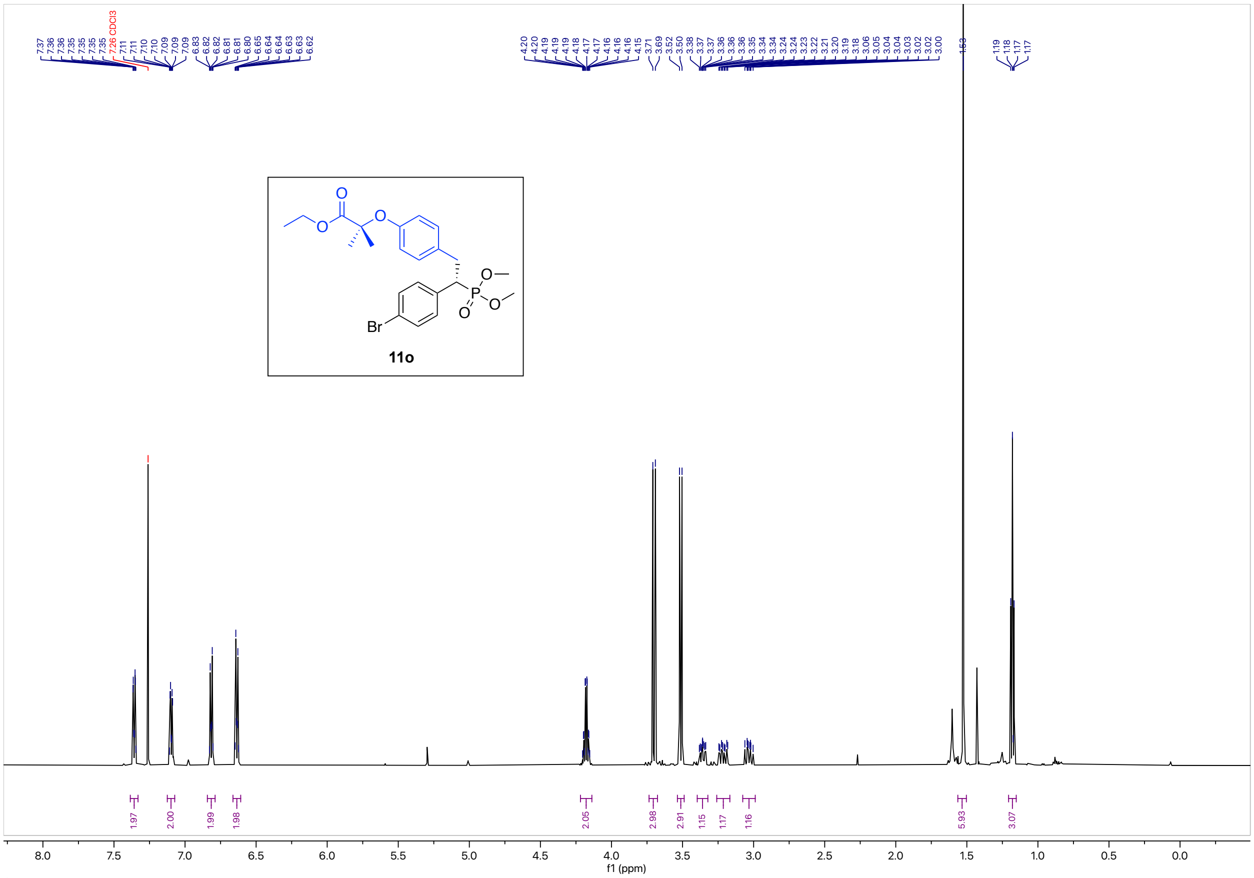

^{1}H NMR spectrum (600 MHz, Chloroform-*d*) (s, 7.26 ppm) of **11o**.

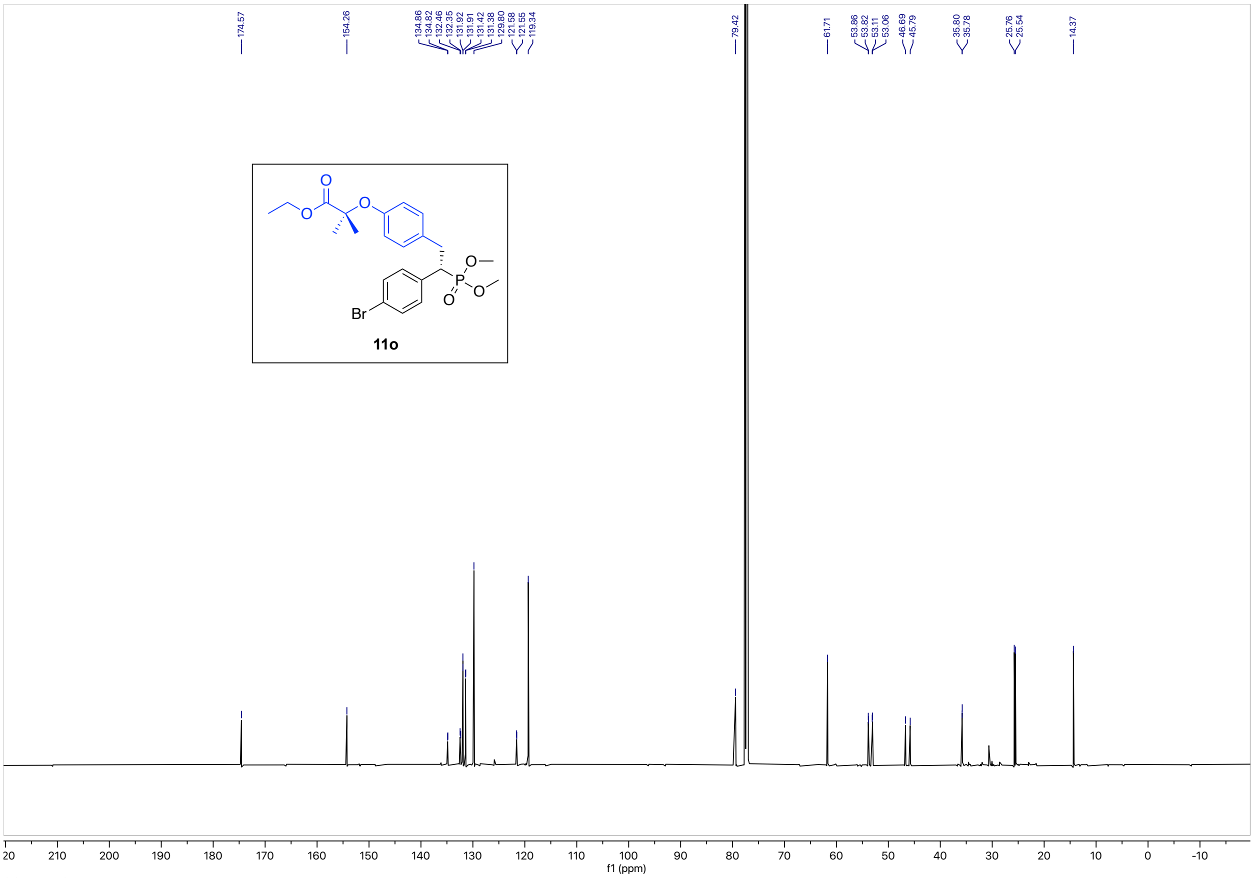

$^{13}C\{^{1}H\}$ NMR spectrum (151 MHz, Chloroform-*d*) (t, 77.36 ppm) of **11o**.

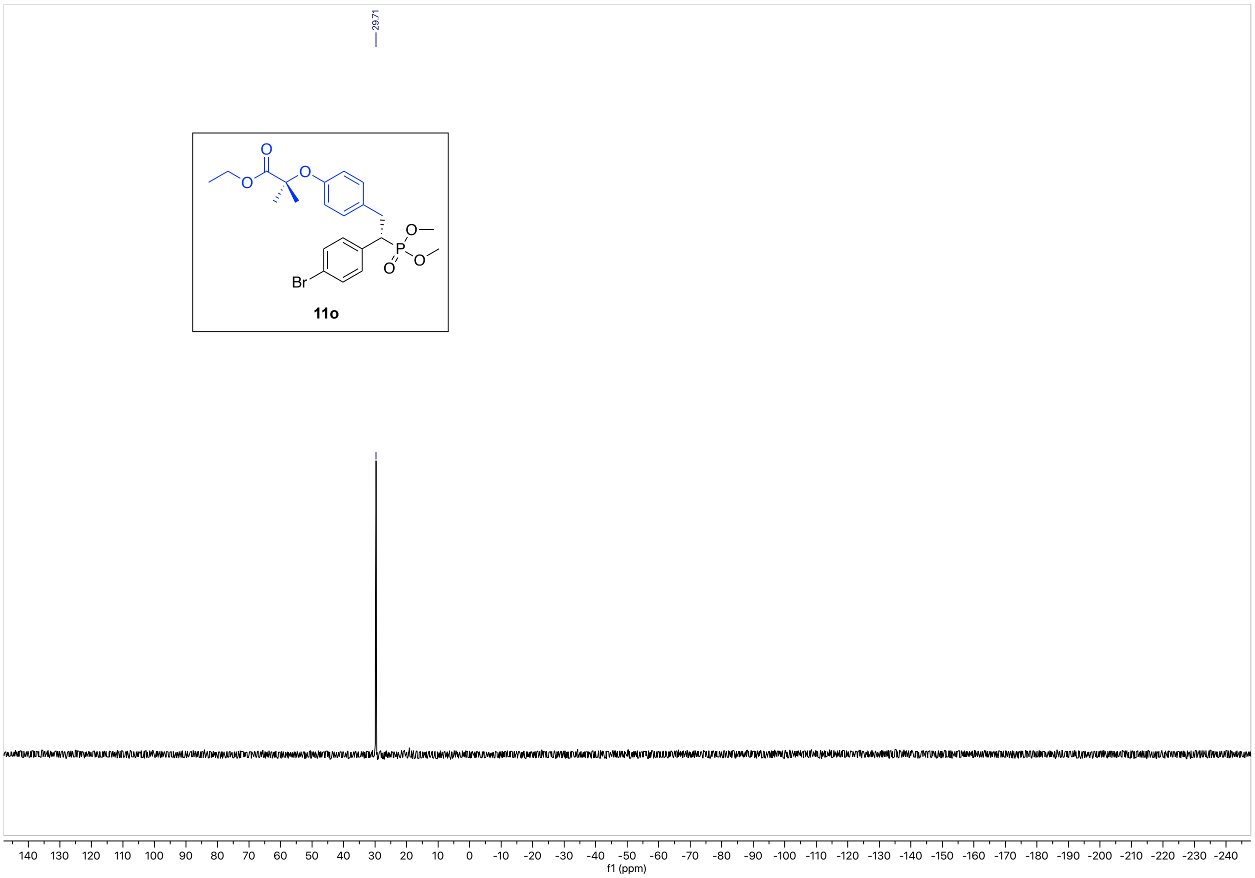

^{31}P NMR spectrum (243 MHz, Chloroform-*d*) of **11o**.

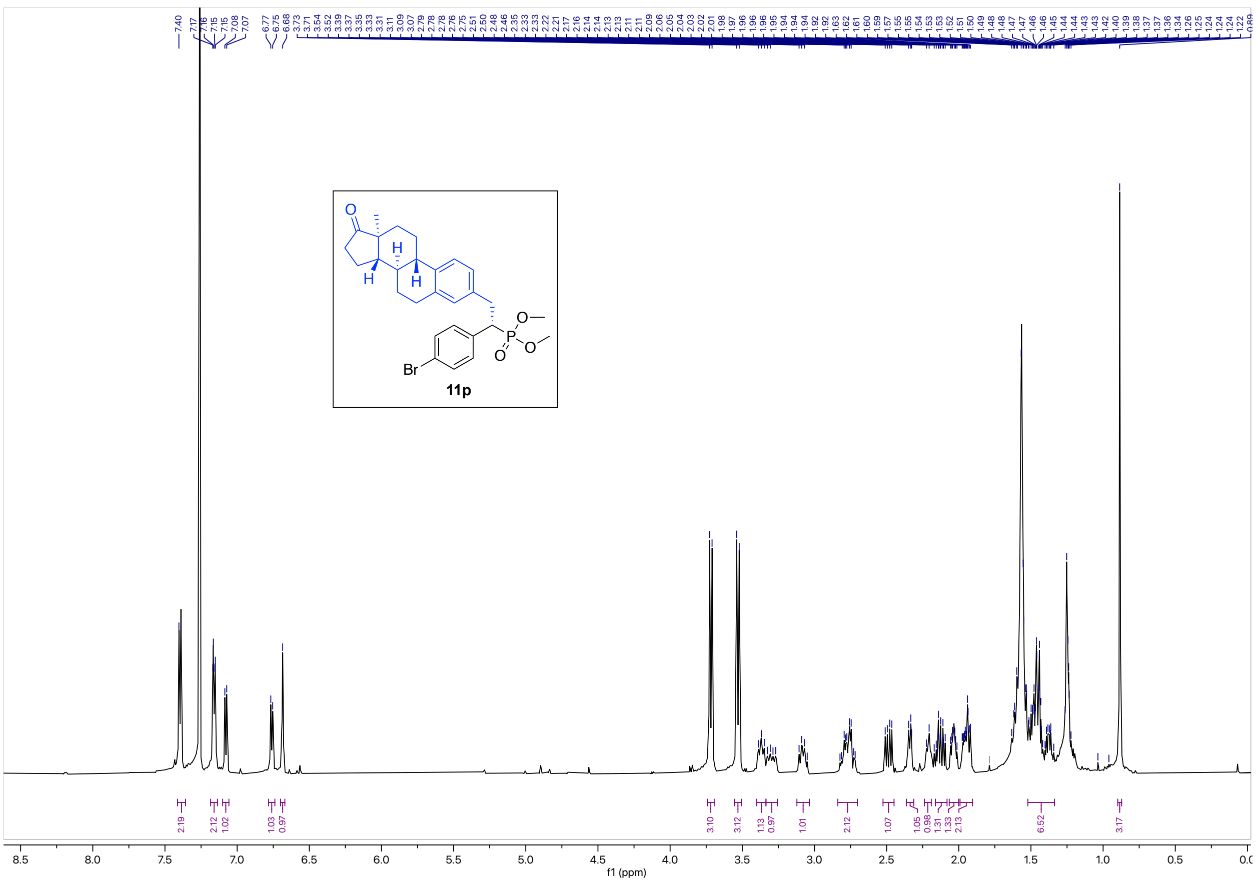

^{1}H NMR spectrum (600 MHz, Chloroform-*d*) (s, 7.26 ppm) of **11p**.

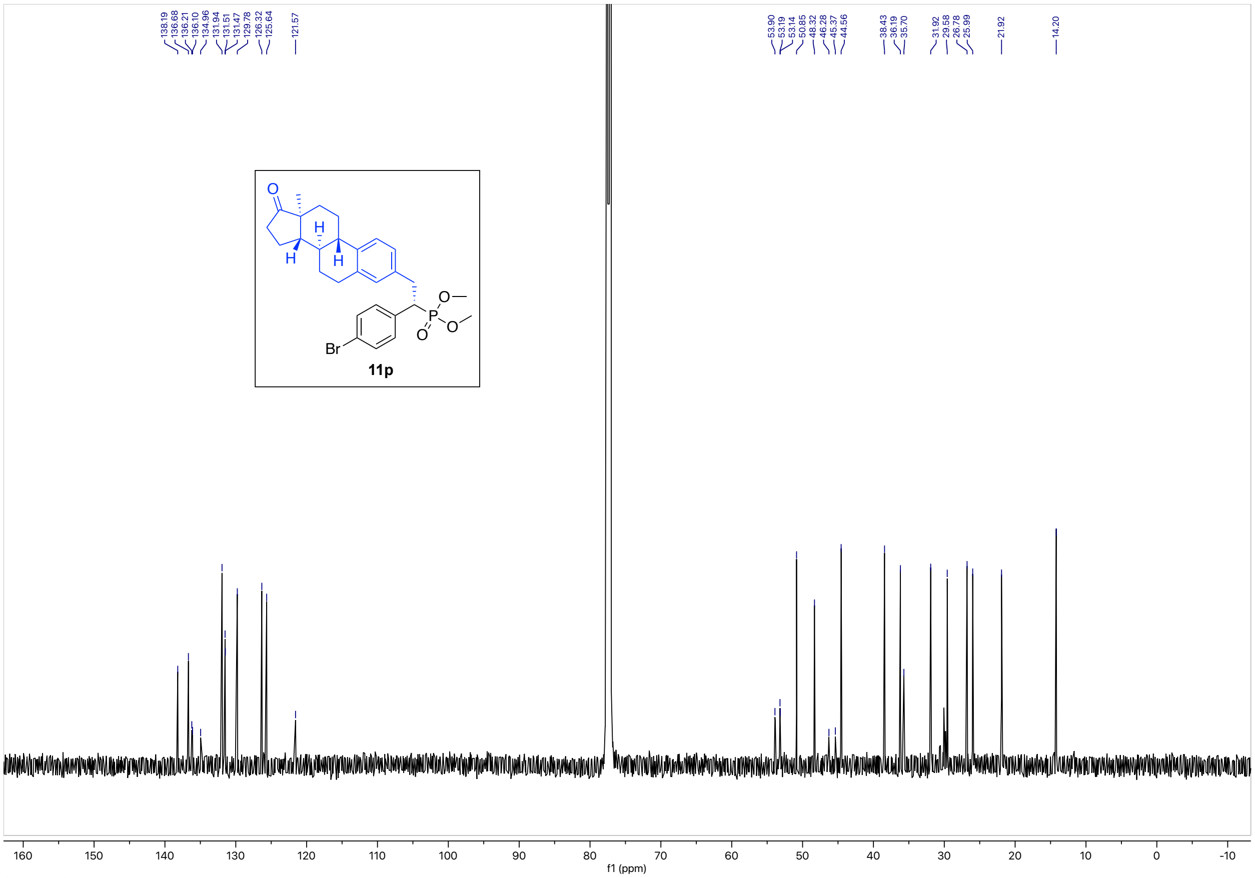

$^{13}C\{^{1}H\}$ NMR spectrum (151 MHz, Chloroform-*d*) (t, 77.36 ppm) of **11p**.

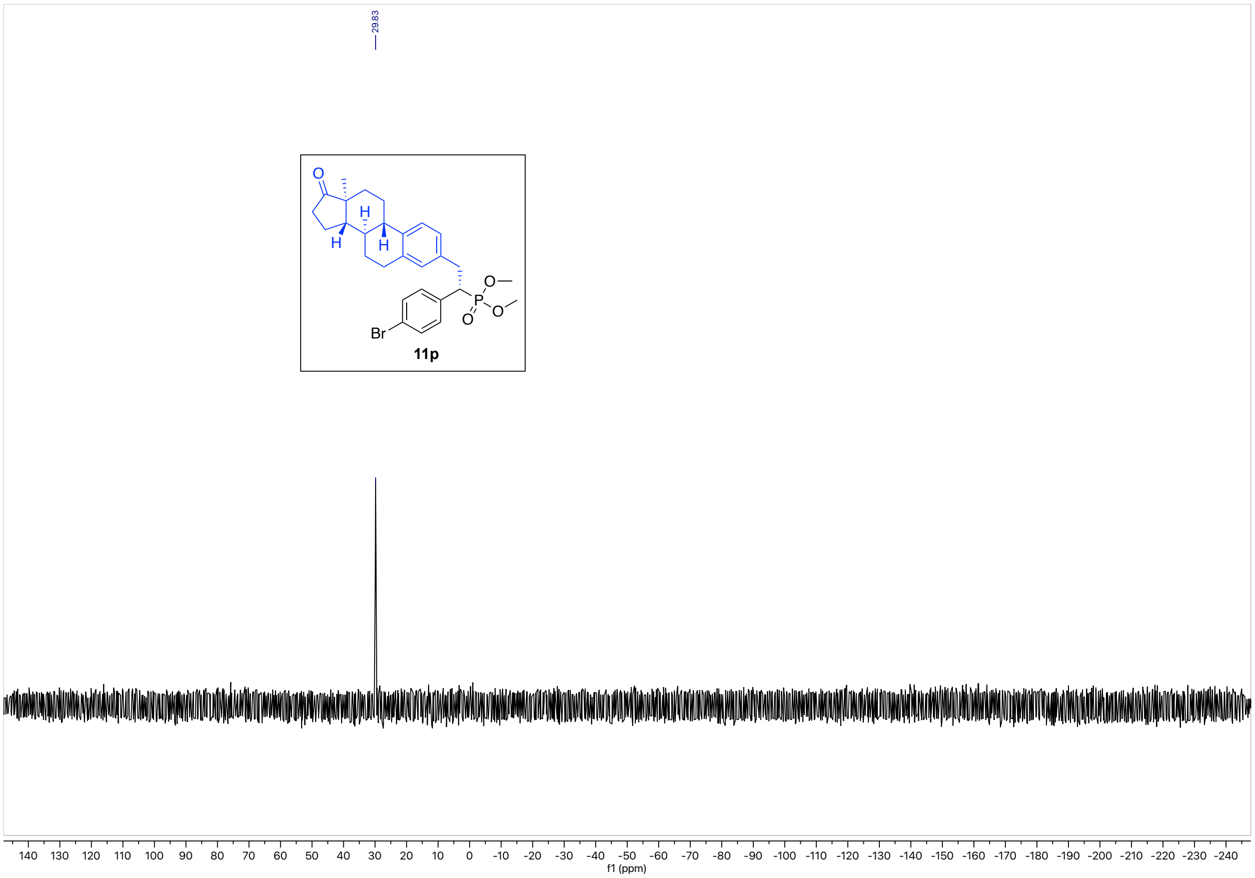

^{31}P NMR spectrum (243 MHz, Chloroform-*d*) of **11p**.

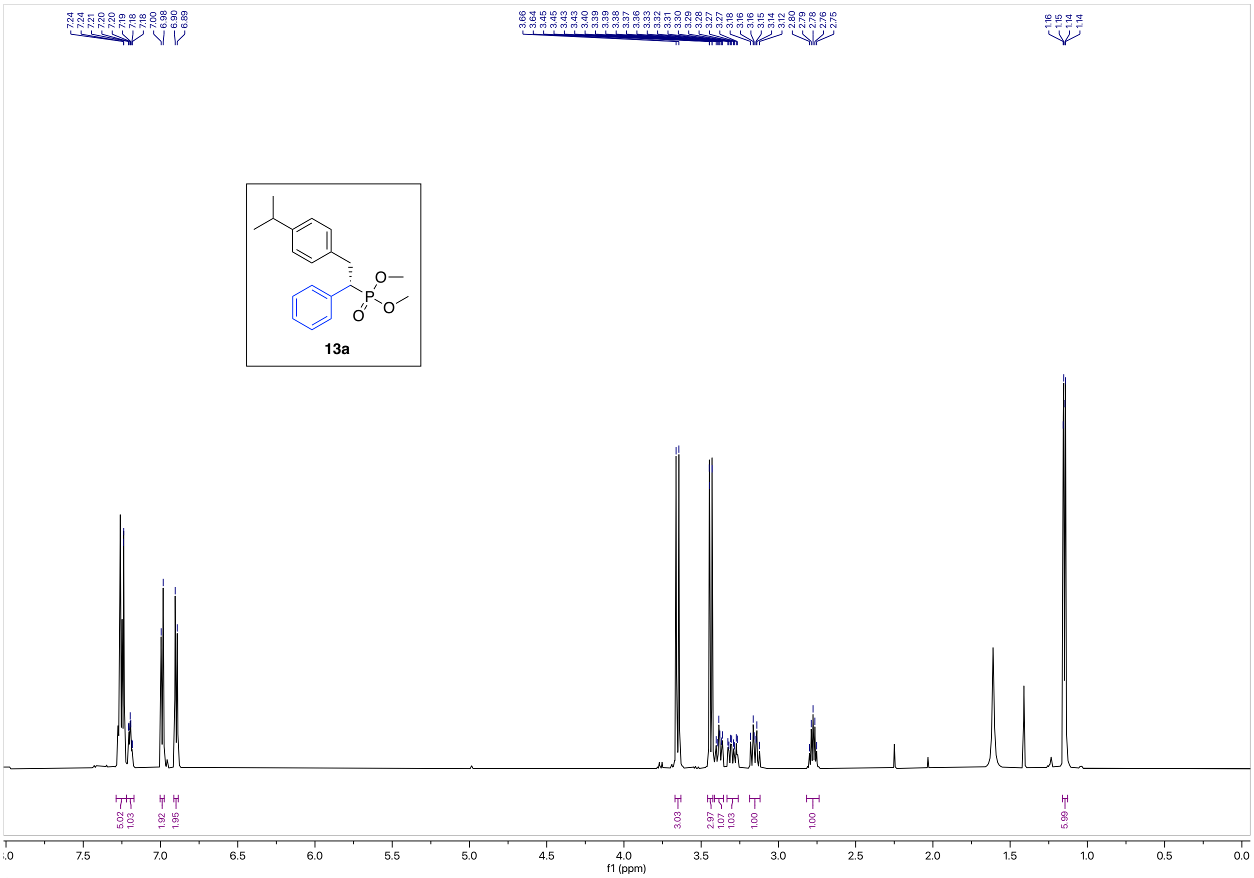

^{1}H NMR spectrum (600 MHz, Chloroform-*d*) (s, 7.26 ppm) of **13a**.

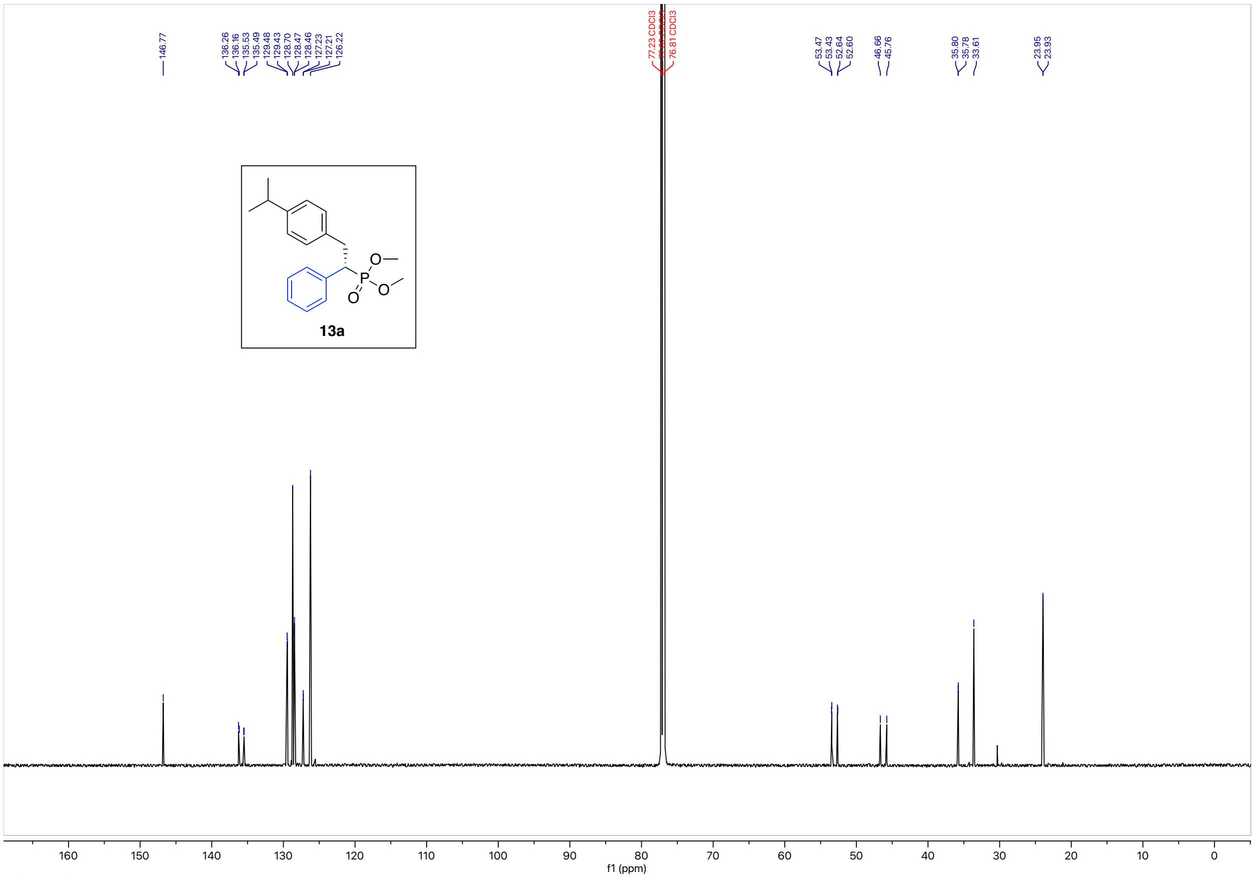

$^{13}C\{^{1}H\}$ NMR spectrum (151 MHz, Chloroform-*d*) (t, 77.36 ppm) of **13a**.

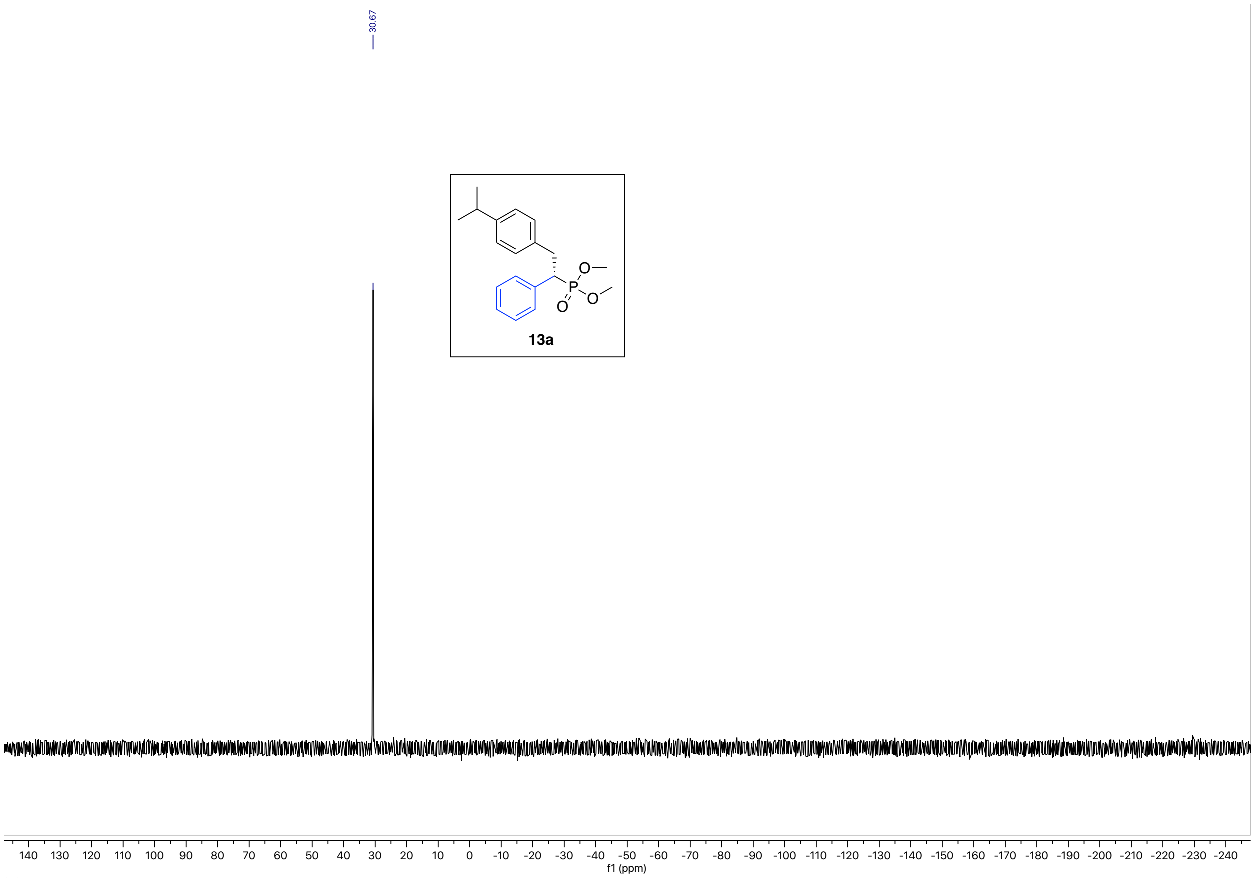

^{31}P NMR spectrum (243 MHz, Chloroform-*d*) of **13a**.

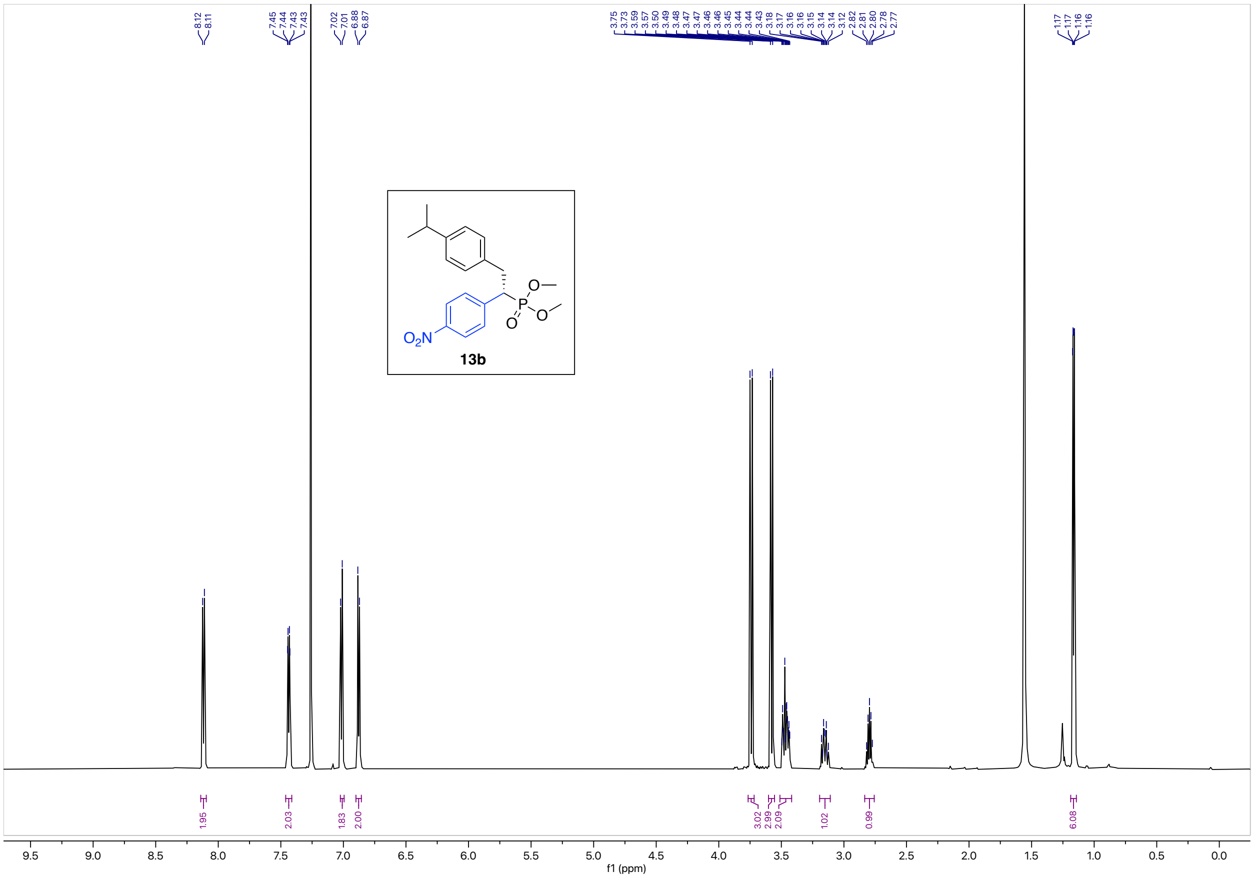

^{1}H NMR spectrum (600 MHz, Chloroform-*d*) (s, 7.26 ppm) of **13b**.

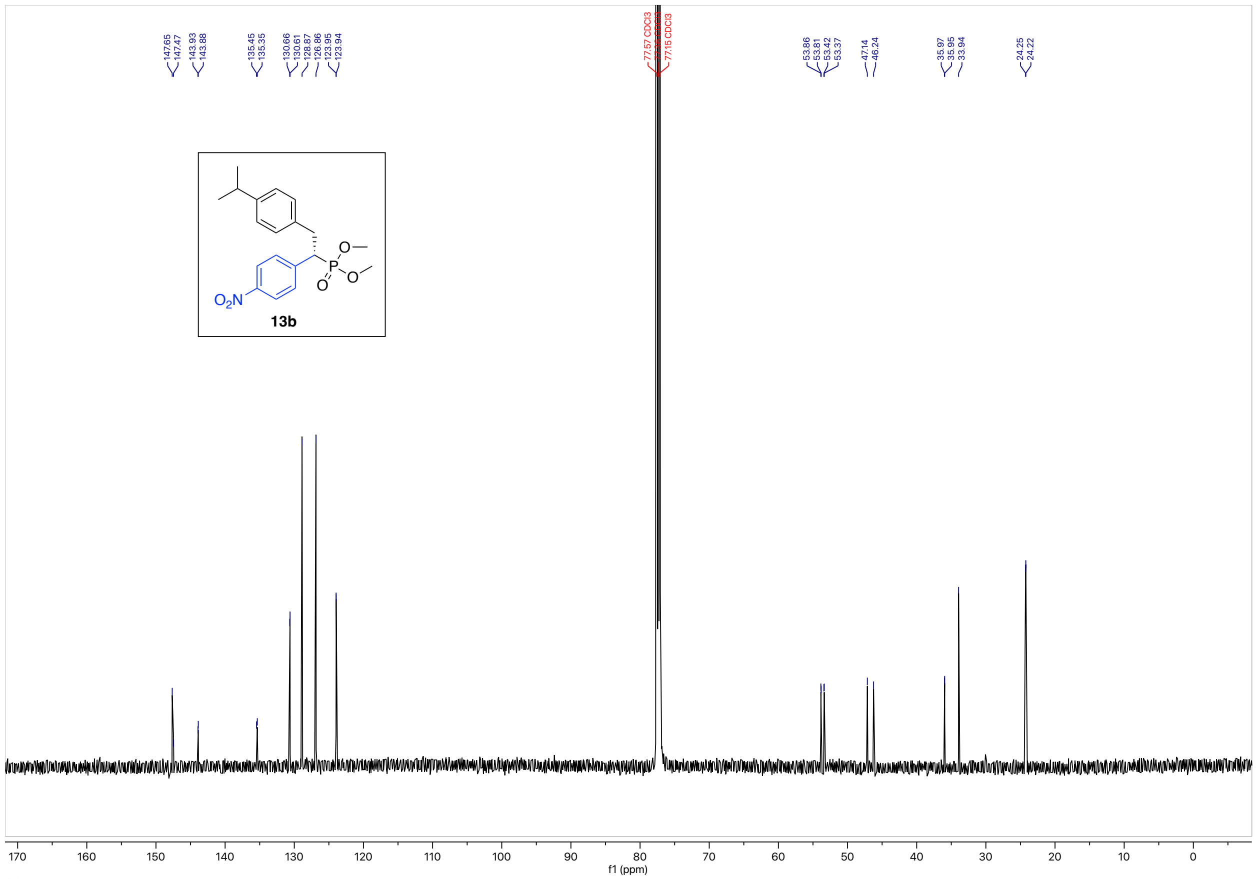

$^{13}C\{^1H\}$ NMR spectrum (151 MHz, Chloroform-*d*) (t, 77.36 ppm) of **13b**.

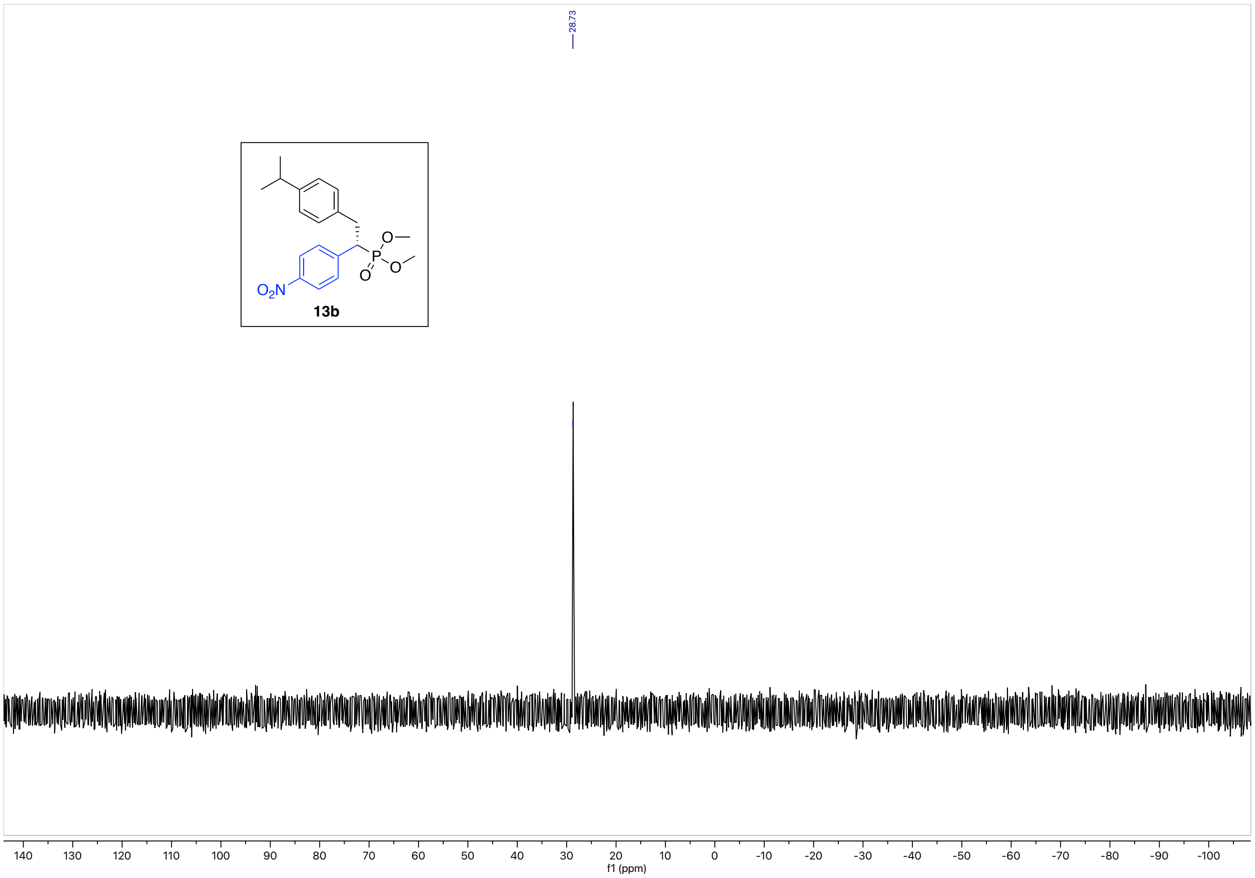

^{31}P NMR spectrum (243 MHz, Chloroform-*d*) of **13b**.

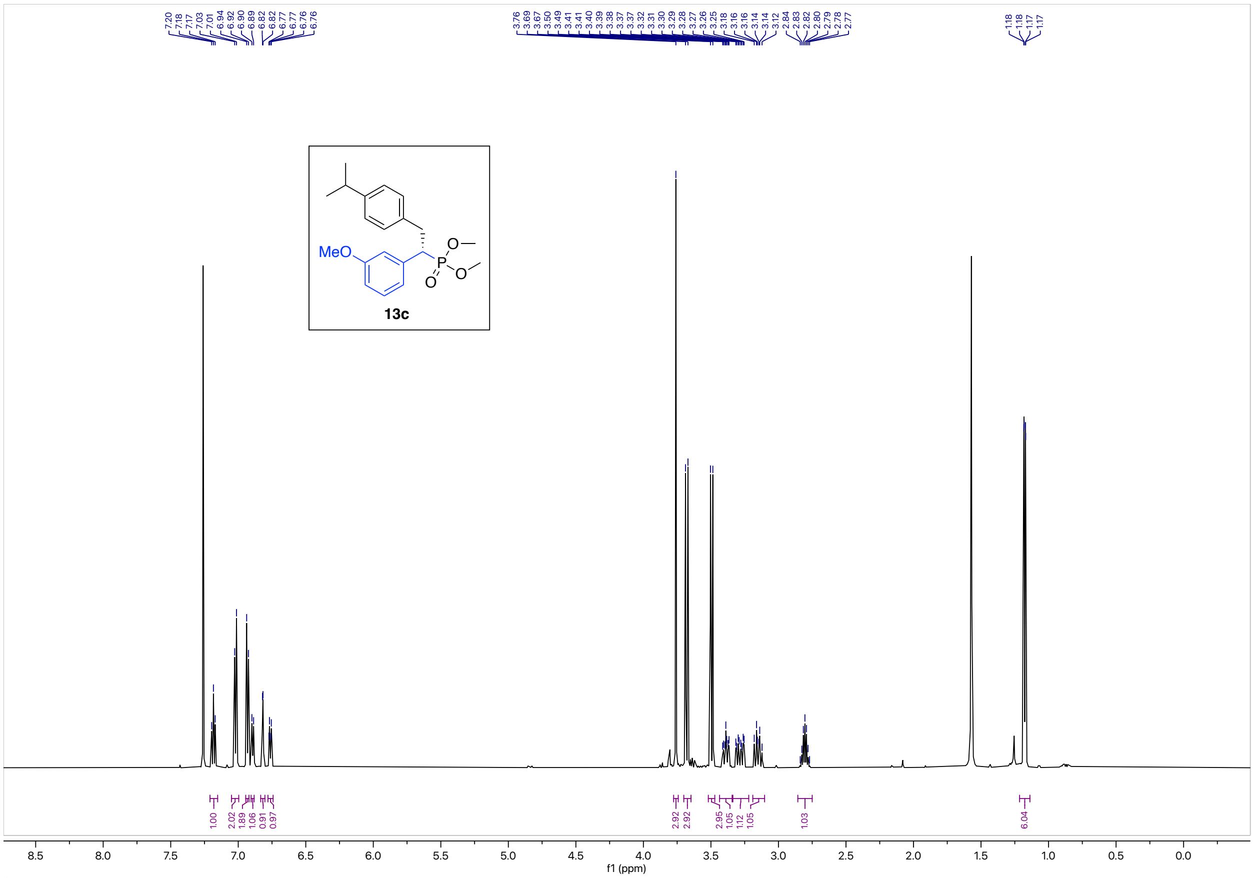

^{1}H NMR spectrum (600 MHz, Chloroform-*d*) (s, 7.26 ppm) of **13c**.

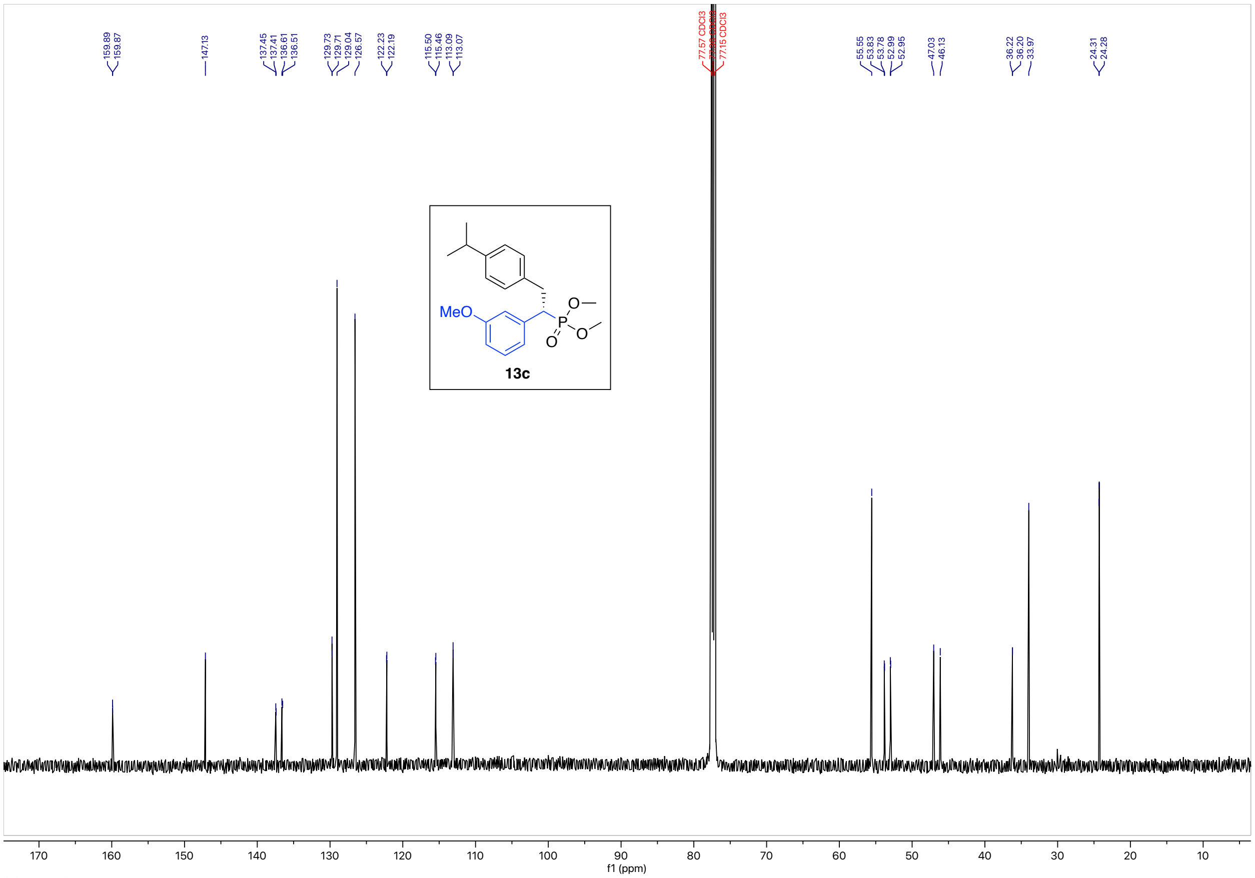

$^{13}C\{^1H\}$ NMR spectrum (151 MHz, Chloroform-*d*) (t, 77.36 ppm) of **13c**.

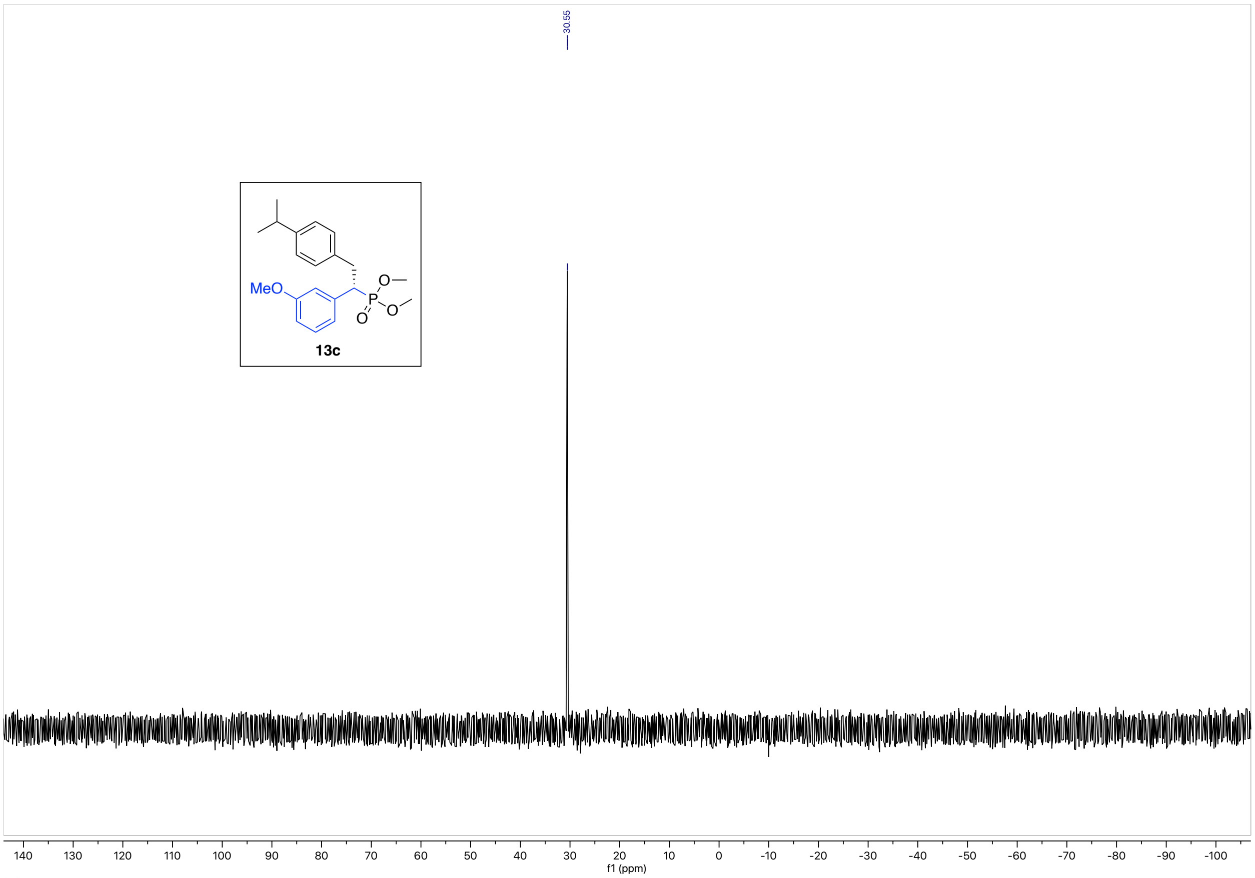

^{31}P NMR spectrum (243 MHz, Chloroform-*d*) of **13c**.

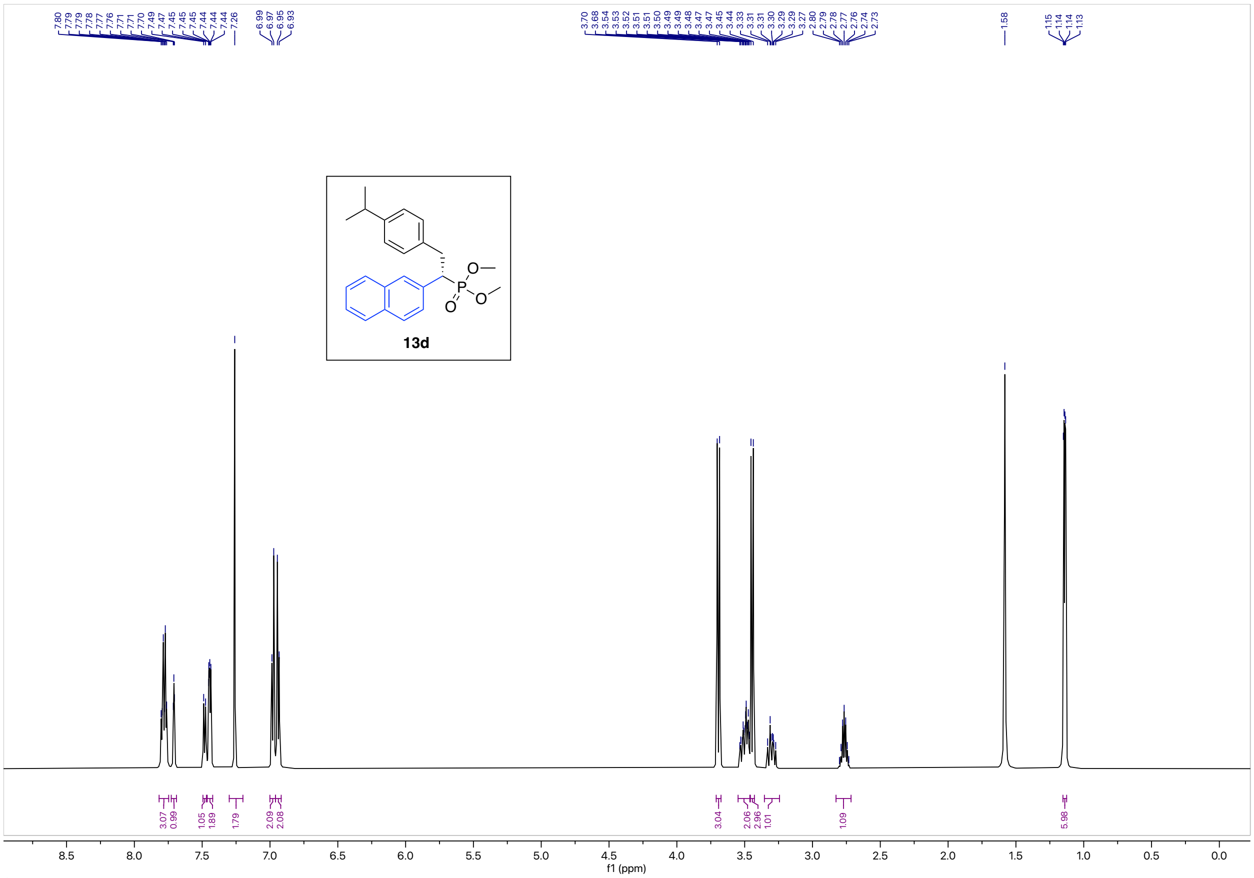

^{1}H NMR spectrum (600 MHz, Chloroform-*d*) (s, 7.26 ppm) of **13d**.

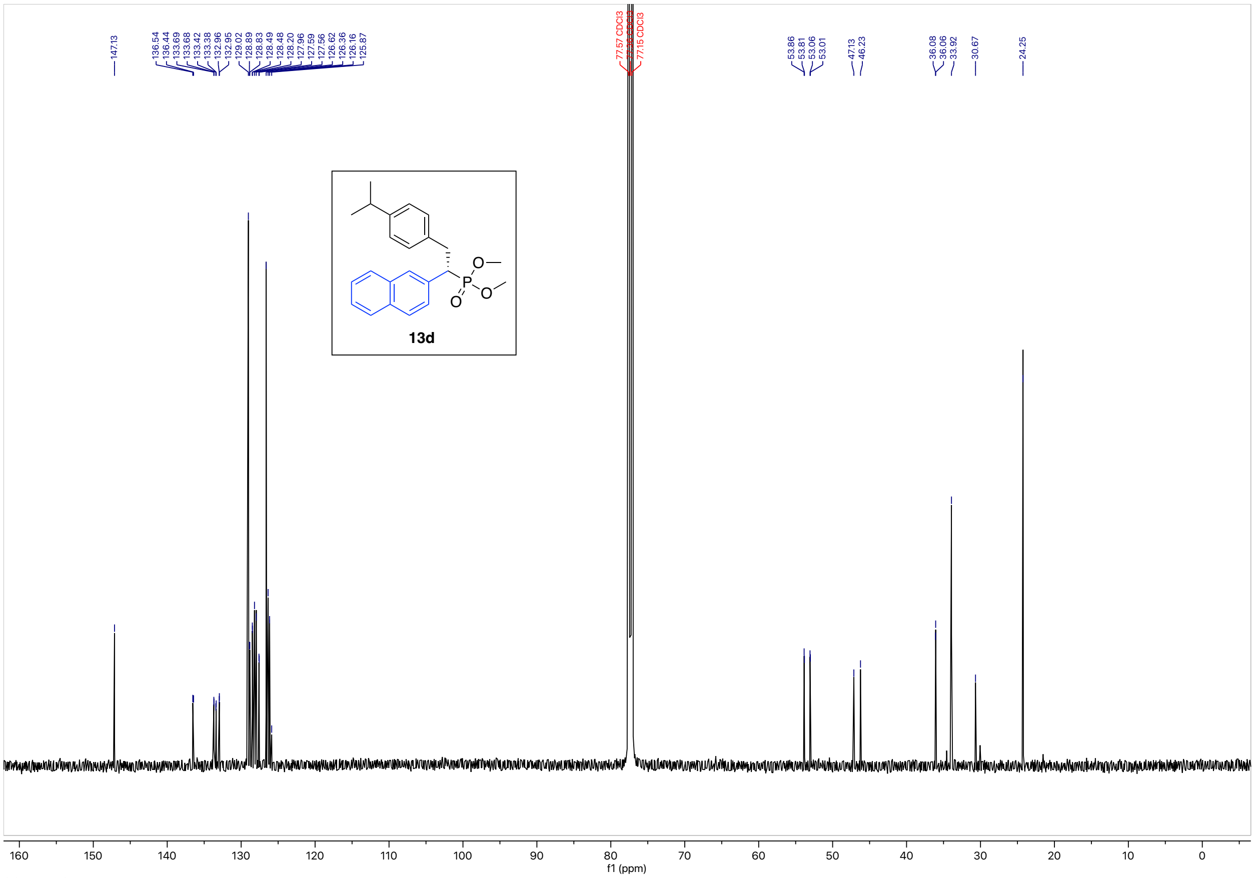

$^{13}C\{^{1}H\}$ NMR spectrum (151 MHz, Chloroform-*d*) (t, 77.36 ppm) of **13d**.

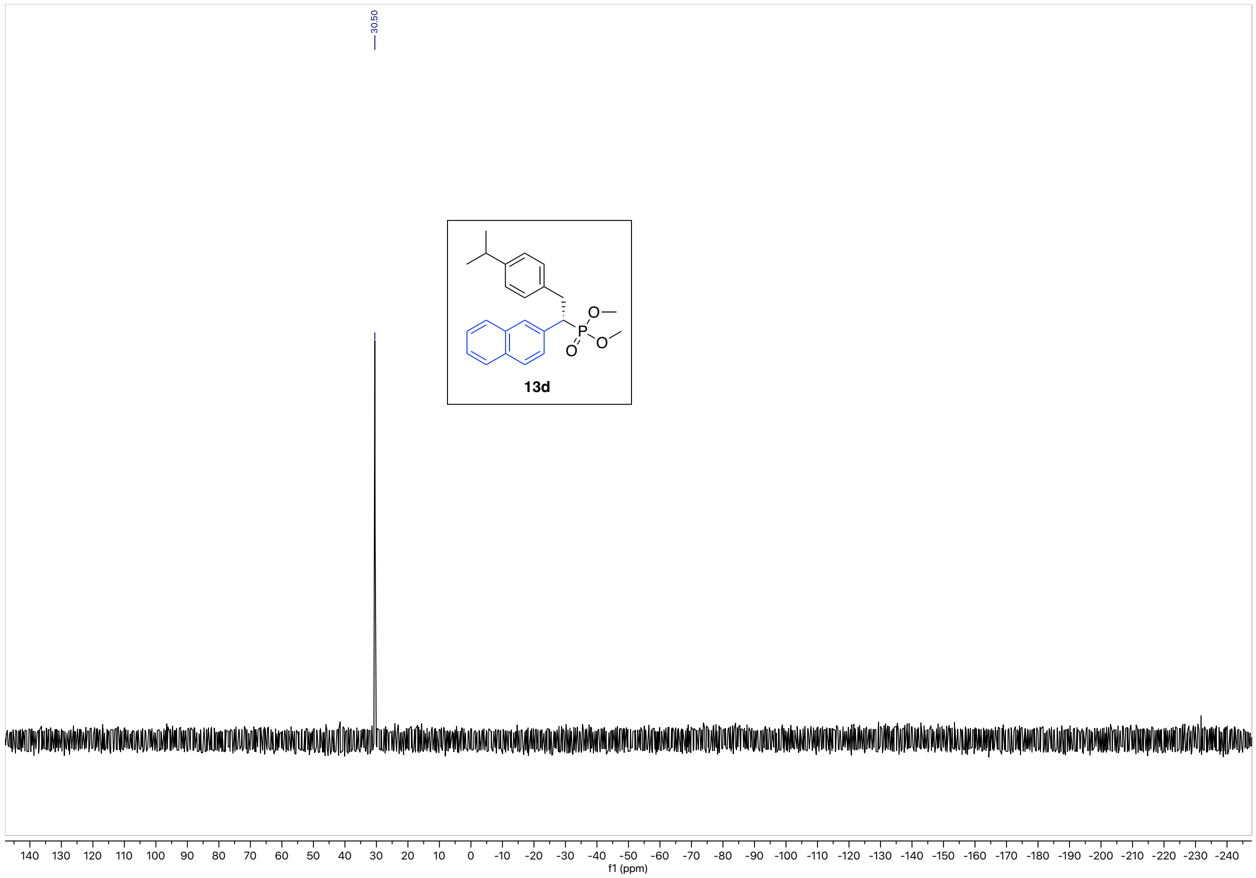

^{31}P NMR spectrum (243 MHz, Chloroform-*d*) of **13d**.

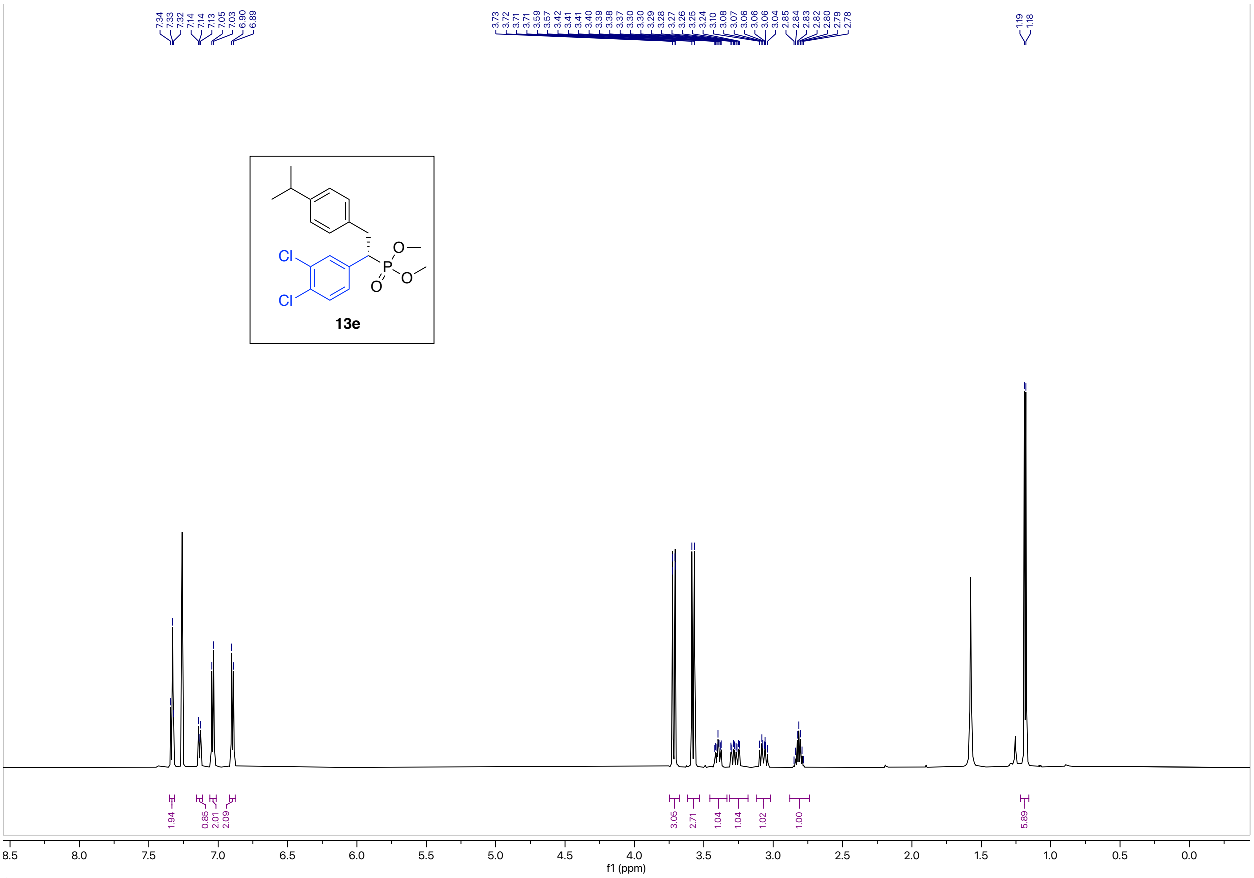

^{1}H NMR spectrum (600 MHz, Chloroform-*d*) (s, 7.26 ppm) of **13e**.

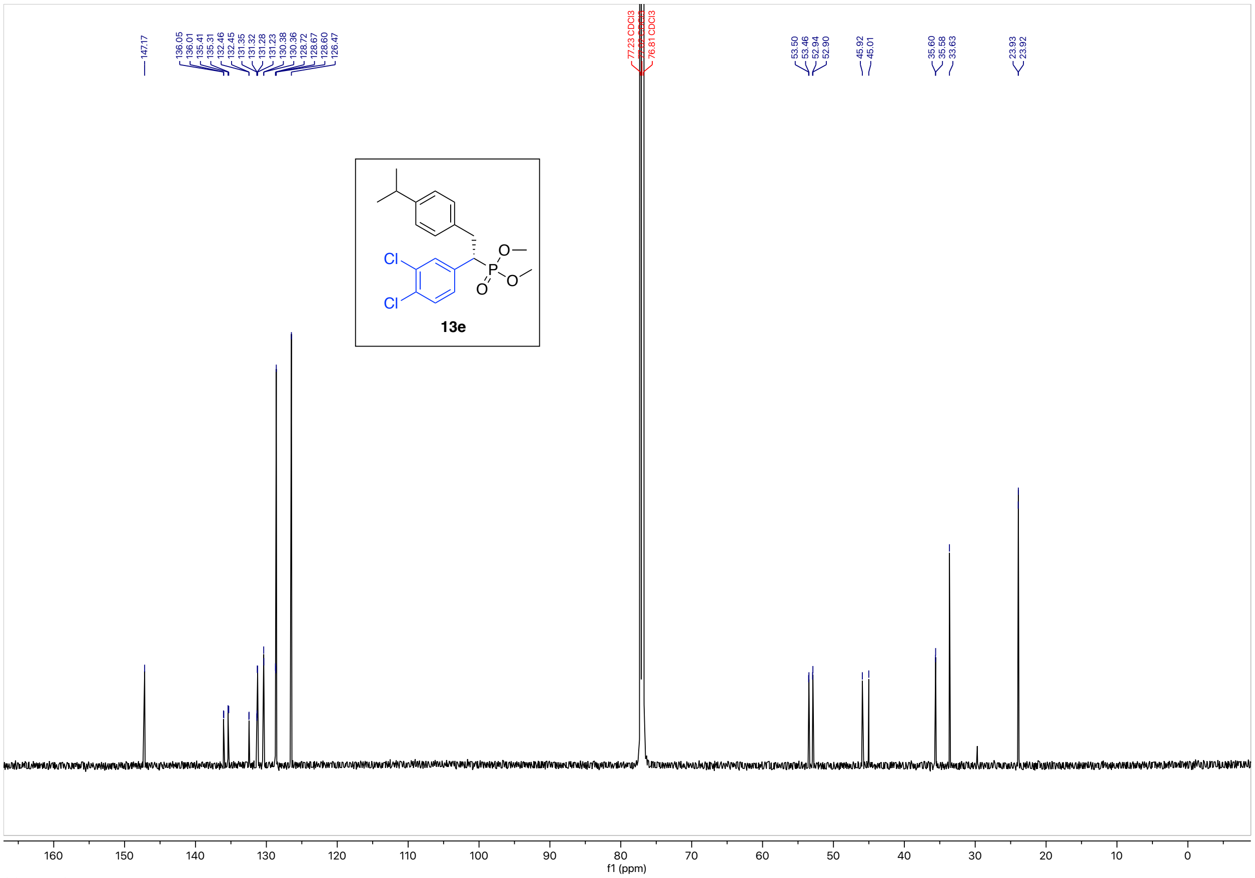

$^{13}C\{^{1}H\}$ NMR spectrum (151 MHz, Chloroform-*d*) (t, 77.36 ppm) of **13e**.

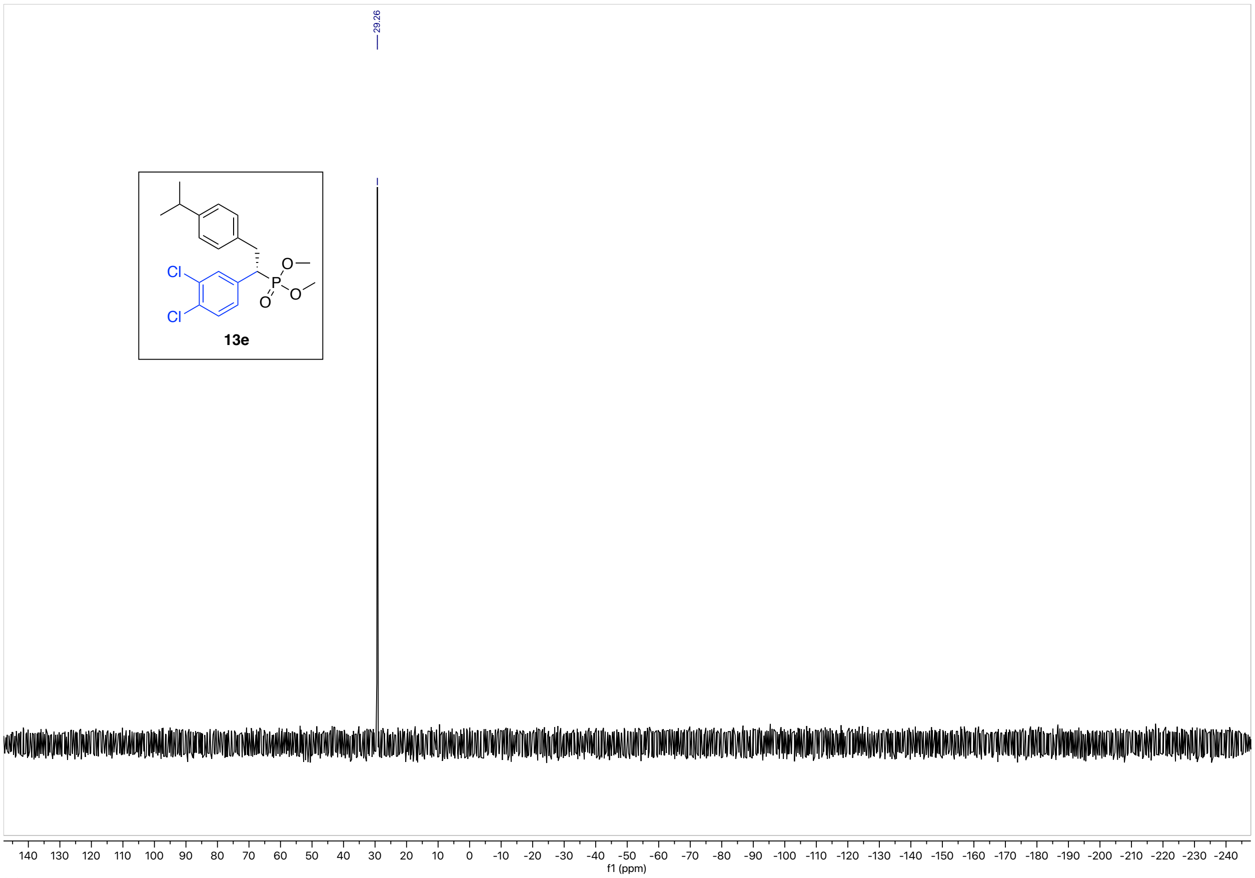

^{31}P NMR spectrum (243 MHz, Chloroform-*d*) of **13e**.

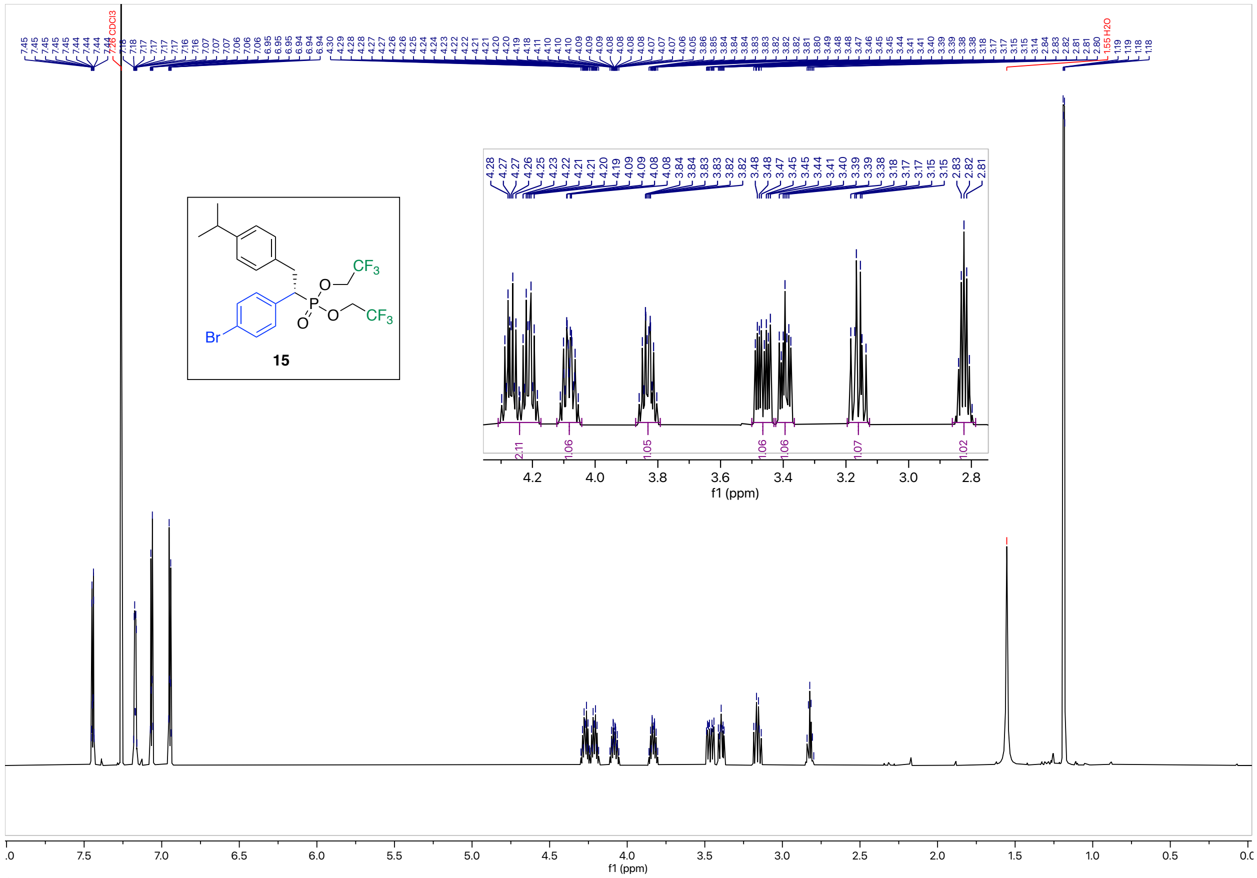

^{1}H NMR spectrum (600 MHz, Chloroform-d) of (s, 7.26 ppm) of **15**.

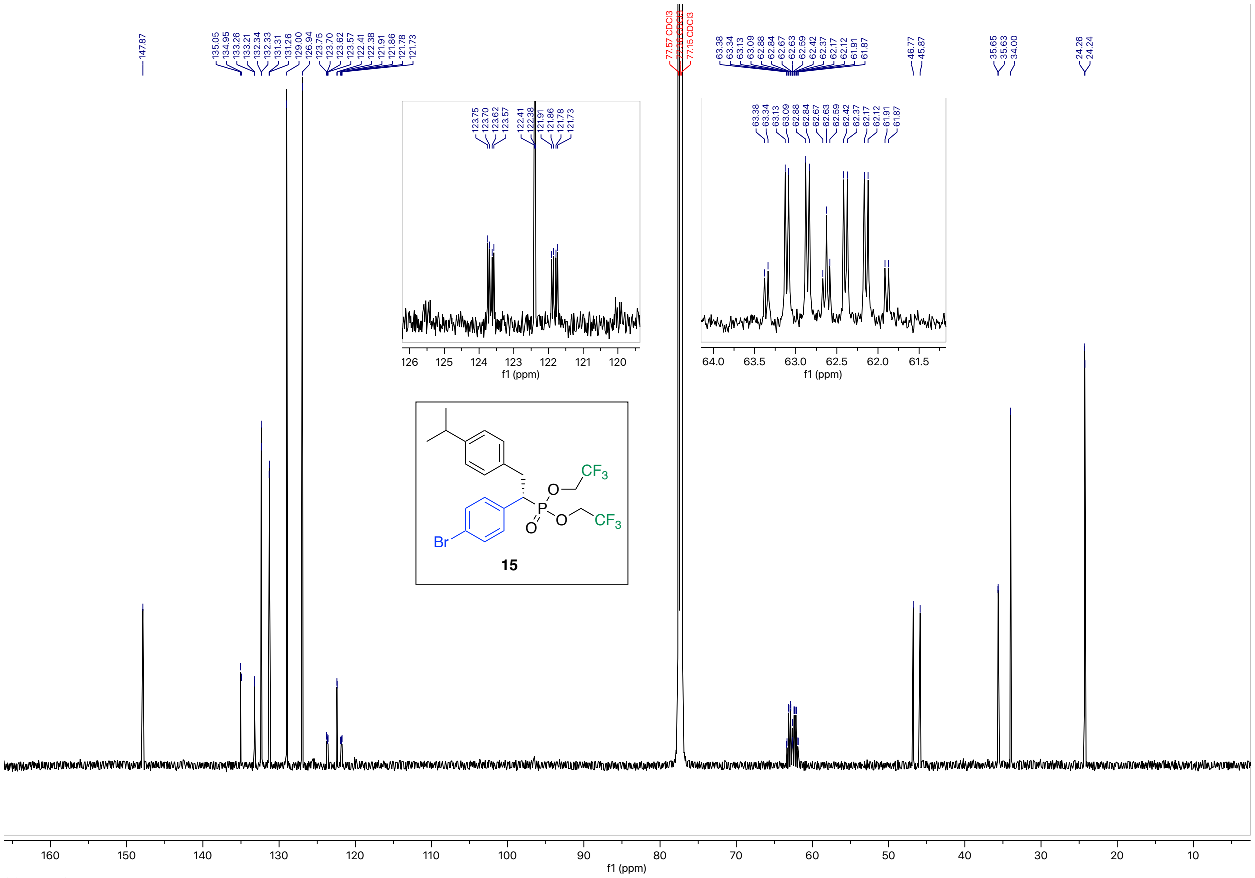

$^{13}C\{^{1}H\}$ NMR spectrum (151 MHz, Chloroform-d) of (t, 77.36 ppm) of **15**.

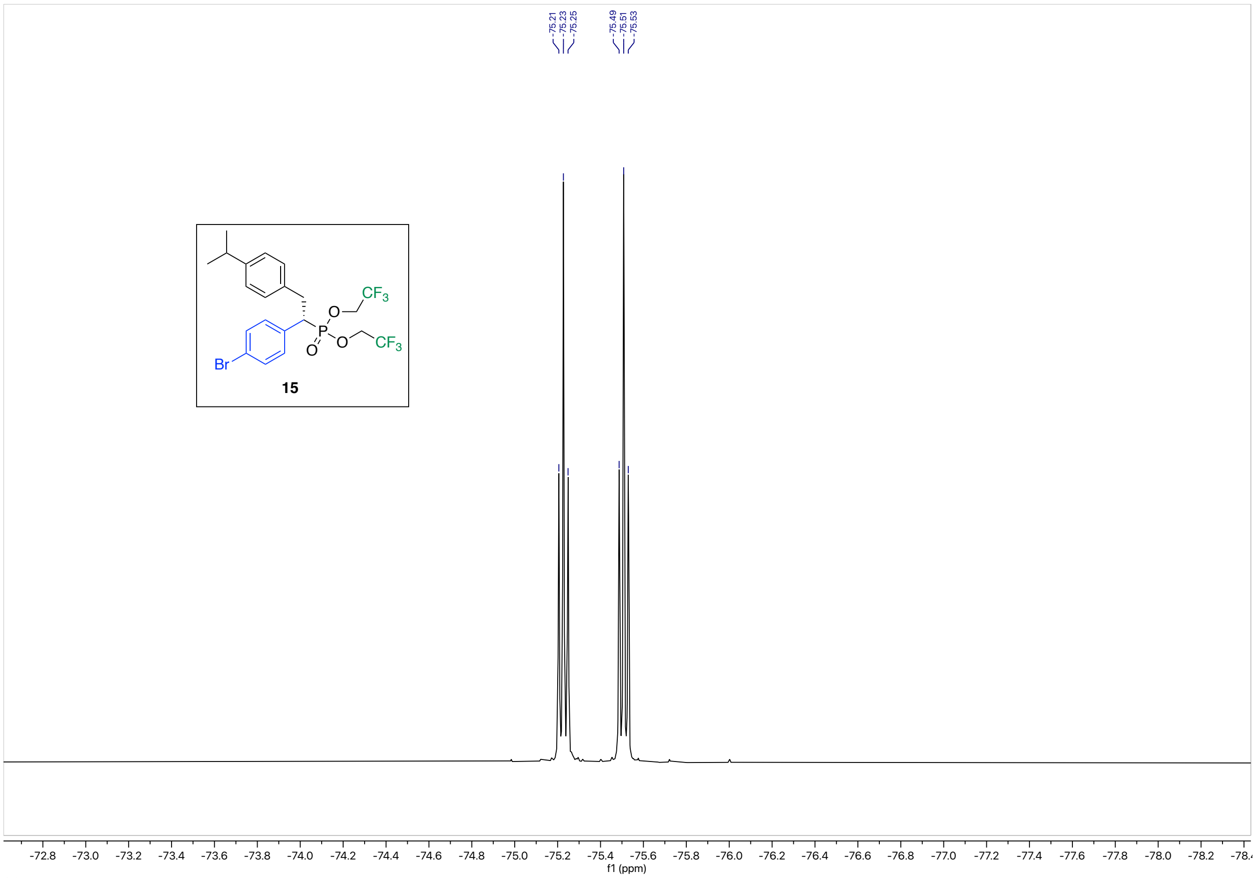

^{19}F NMR spectrum (376 MHz, Chloroform-d) of **15**.

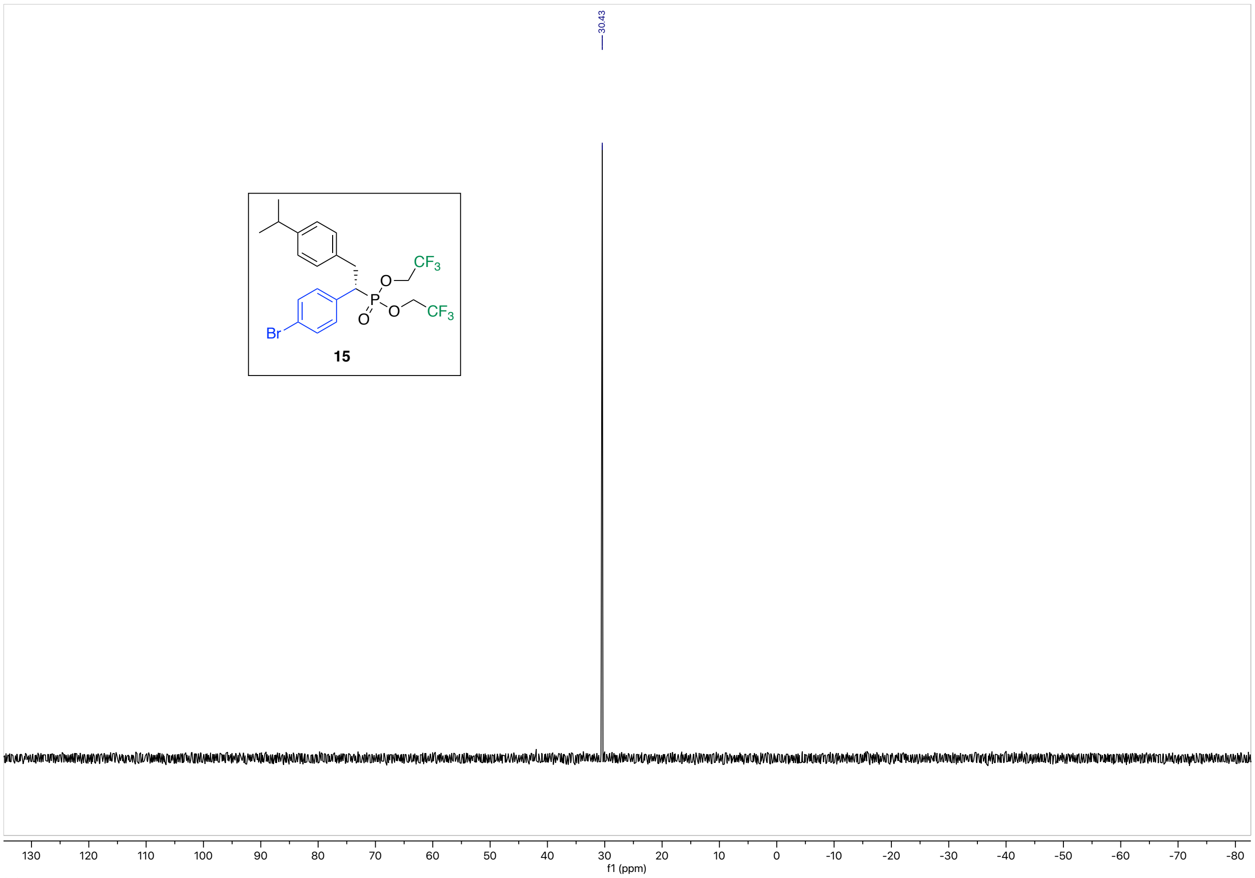

^{31}P NMR spectrum (162 MHz, Chloroform-d) of **15**.

9. Enantioselectivity Determination by HPLC or SFC

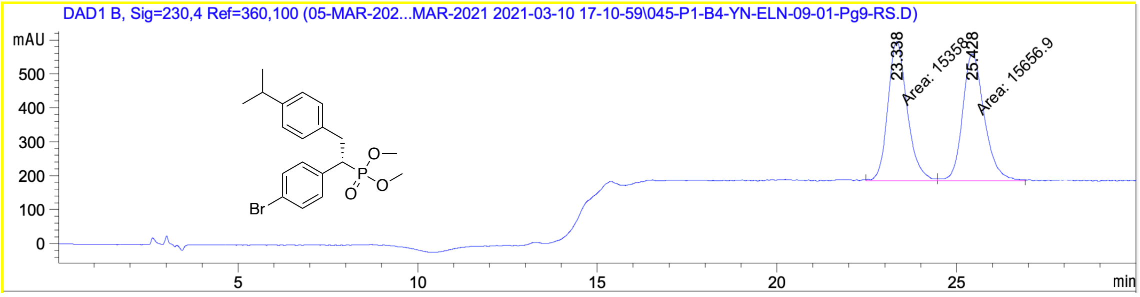

Peak #	RetTime [min]	Type	Width [min]	Area [mAU*s]	Height [mAU]	Area %
1	23.338	MF	0.6247	1.53580e4	409.71216	49.5182
2	25.428	FM	0.6982	1.56569e4	373.71921	50.4818
Totals :				3.10149e4	783.43137	

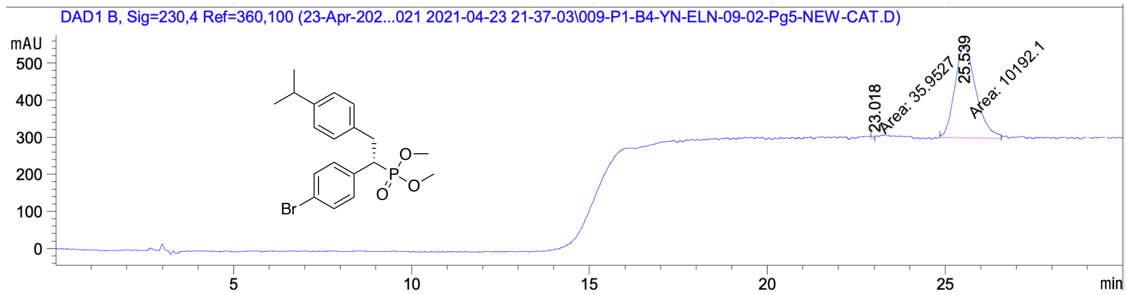

Peak #	RetTime [min]	Type	Width [min]	Area [mAU*s]	Height [mAU]	Area %
1	23.018	MM	0.1011	35.95274	5.92440	0.3515
2	25.539	MM	0.6825	1.01921e4	248.88594	99.6485
Totals :				1.02280e4	254.81034	

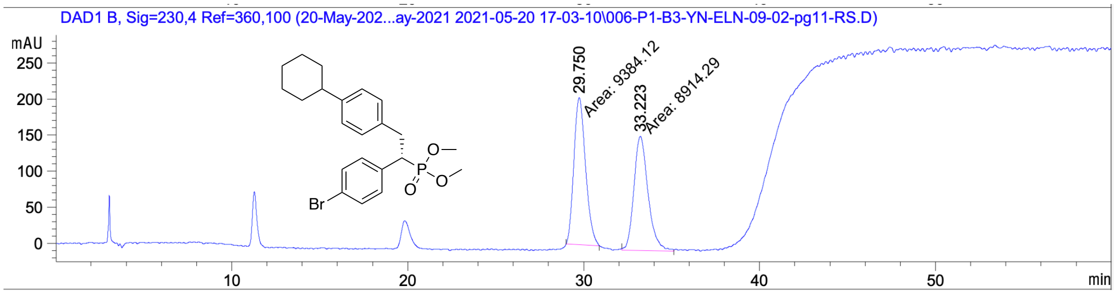

Peak #	RetTime [min]	Type	Width [min]	Area [mAU*s]	Height [mAU]	Area %
1	29.750	MM	0.7670	9384.11621	203.90833	51.2838
2	33.223	MM	0.9359	8914.29004	158.74568	48.7162

Totals : 1.82984e4 362.65401

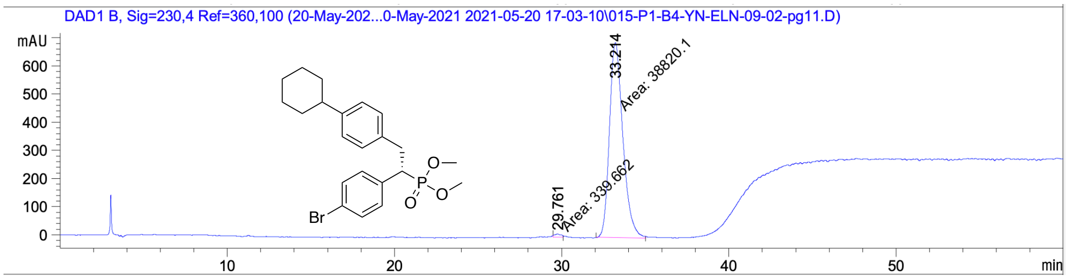

Peak #	RetTime [min]	Type	Width [min]	Area [mAU*s]	Height [mAU]	Area %
1	29.761	MM	0.4746	339.66165	11.92875	0.8674
2	33.214	MM	0.9334	3.88201e4	693.16809	99.1326

Totals : 3.91597e4 705.09684

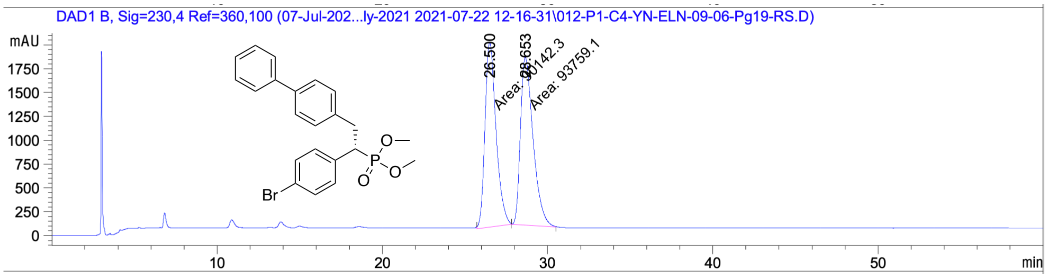

Peak #	RetTime [min]	Type	Width [min]	Area [mAU*s]	Height [mAU]	Area %
1	26.500	MM	0.7761	9.01423e4	1935.90735	49.0166
2	28.653	MM	0.8871	9.37591e4	1761.58472	50.9834

Totals : 1.83901e5 3697.49207

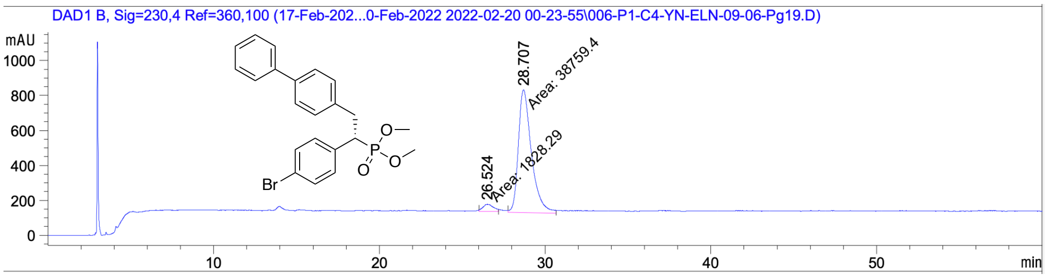

Peak #	RetTime [min]	Type	Width [min]	Area [mAU*s]	Height [mAU]	Area %
1	26.524	MM	0.7563	1828.28589	40.29083	4.5045
2	28.707	MM	0.9187	3.87594e4	703.18109	95.4955

Totals : 4.05877e4 743.47192

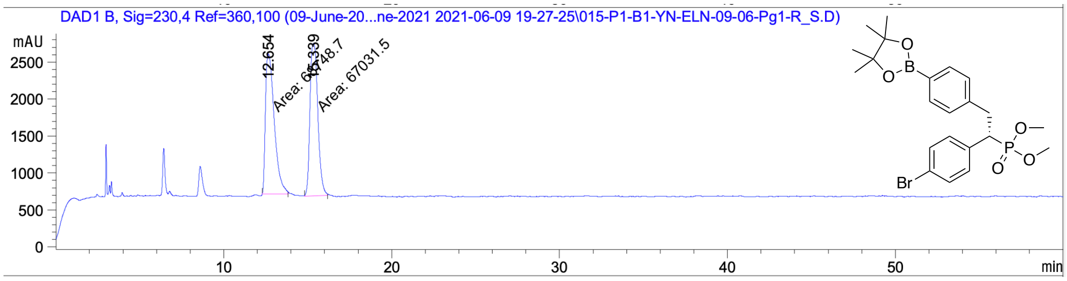

Peak #	RetTime [min]	Type	Width [min]	Area [mAU*s]	Height [mAU]	Area %
1	12.654	MM	0.6063	6.87487e4	1889.84363	50.6324
2	15.339	MM	0.5407	6.70315e4	2066.25659	49.3676

Totals : 1.35780e5 3956.10022

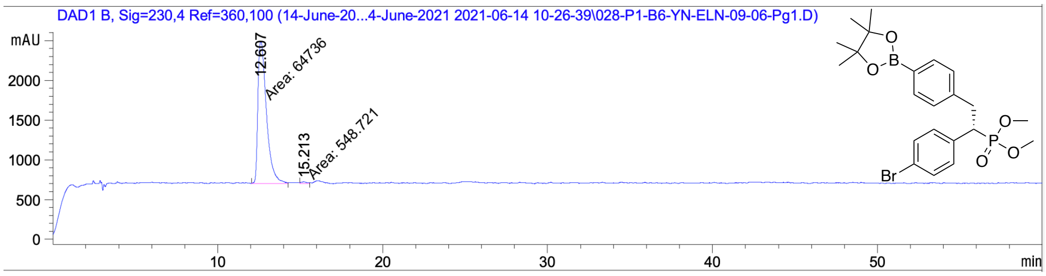

Peak #	RetTime [min]	Type	Width [min]	Area [mAU*s]	Height [mAU]	Area %
1	12.607	MM	0.6034	6.47360e4	1788.11292	99.1595
2	15.213	MM	0.4084	548.72058	22.39397	0.8405

Totals : 6.52847e4 1810.50688

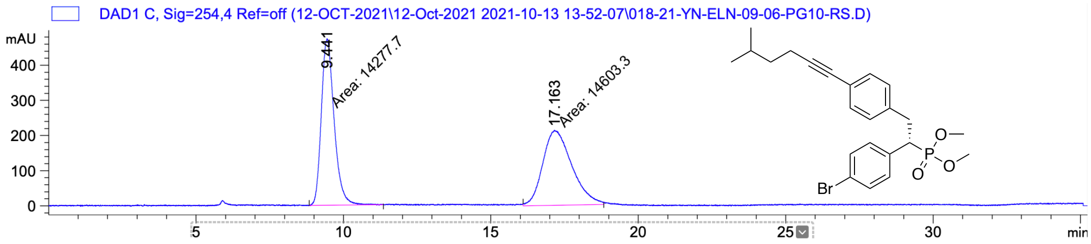

Peak #	RetTime [min]	Type	Width [min]	Area [mAU*s]	Height [mAU]	Area %
1	9.441	MM	0.5020	1.42777e4	474.00946	49.4364
2	17.163	MM	1.1377	1.46033e4	213.92677	50.5636
Totals :				2.88810e4	687.93623	

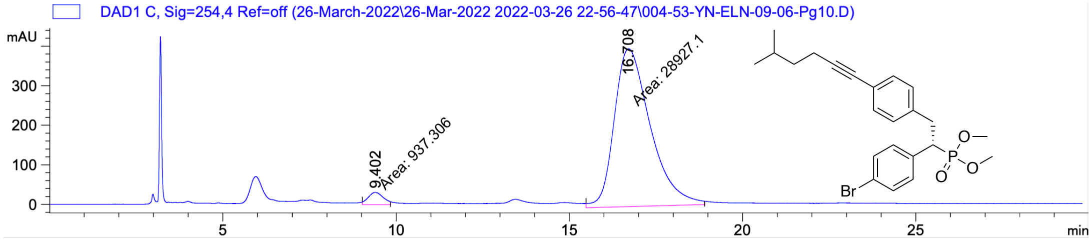

Peak #	RetTime [min]	Type	Width [min]	Area [mAU*s]	Height [mAU]	Area %
1	9.402	MM	0.5095	937.30646	30.66275	3.1385
2	16.708	MM	1.2074	2.89271e4	399.29587	96.8615
Totals :				2.98644e4	429.95862	

SFC

AS3_7%MeOH_IPA_0_2% Formic Acid_2.5mL/min_5min

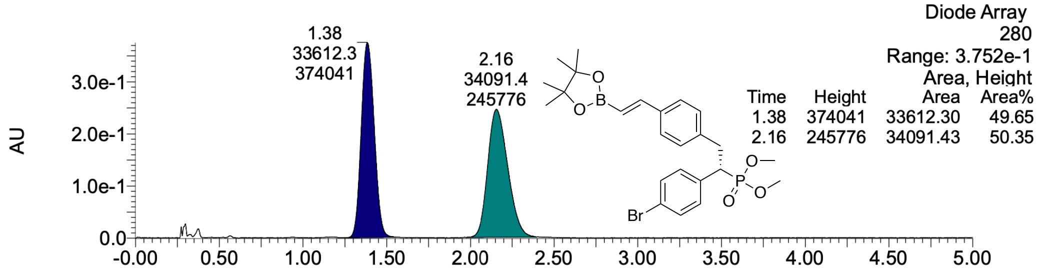

Time	Height	Area	Area%
1.38	374041	33612.30	49.65
2.16	245776	34091.43	50.35

AS3_7%MeOH_IPA_0_2% Formic Acid_2.5mL/min_5min

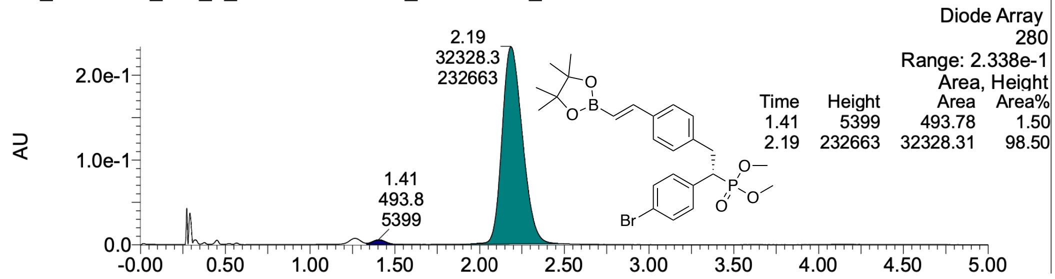

Time	Height	Area	Area%
1.41	5399	493.78	1.50
2.19	232663	32328.31	98.50

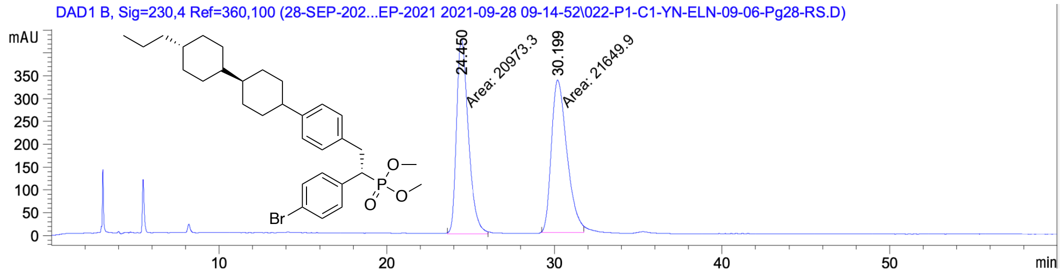

Peak #	RetTime [min]	Type	Width [min]	Area [mAU*s]	Height [mAU]	Area %
1	24.450	MM	0.8188	2.09733e4	426.92914	49.2063
2	30.199	MM	1.0779	2.16499e4	334.76395	50.7937

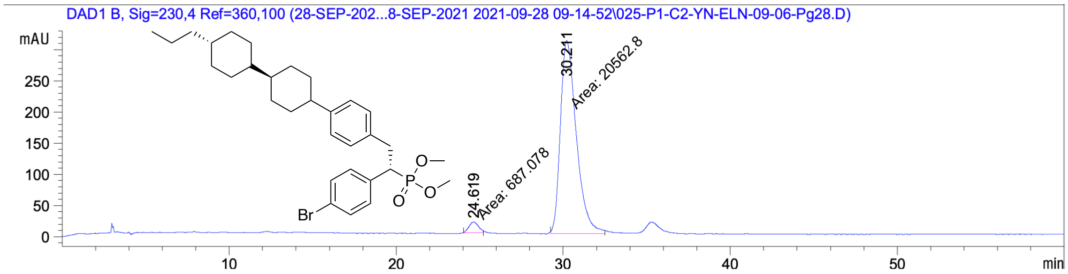

Peak #	RetTime [min]	Type	Width [min]	Area [mAU*s]	Height [mAU]	Area %
1	24.619	MM	0.6734	687.07770	17.00618	3.2333
2	30.211	MM	1.1054	2.05628e4	310.04095	96.7667

Totals : 2.12498e4 327.04713

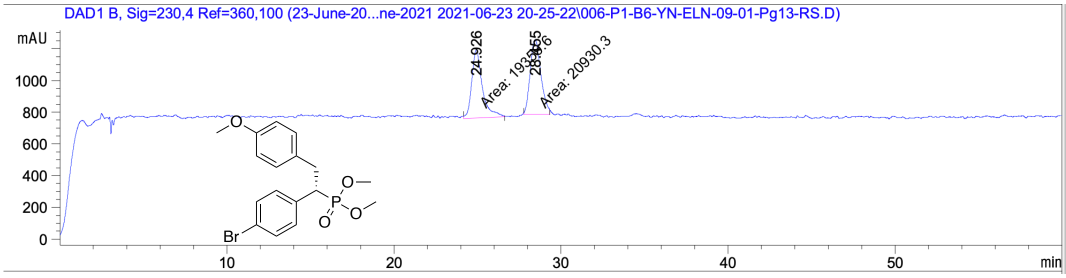

Peak #	RetTime [min]	Type	Width [min]	Area [mAU*s]	Height [mAU]	Area %
1	24.926	MM	0.7497	1.93596e4	430.39493	48.0508
2	28.455	MM	0.7438	2.09303e4	468.96695	51.9492

Totals : 4.02899e4 899.36188

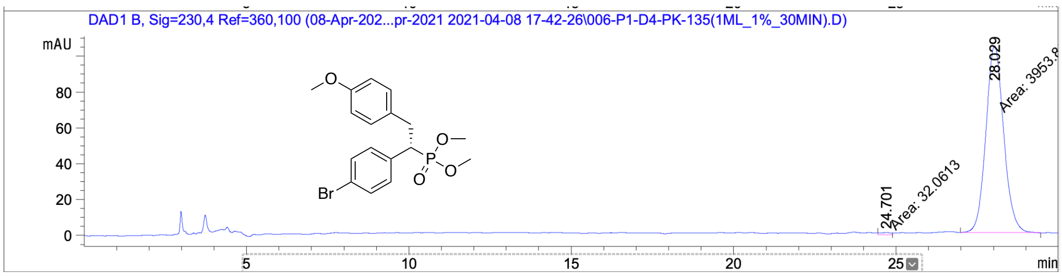

Peak #	RetTime [min]	Type	Width [min]	Area [mAU*s]	Height [mAU]	Area %
1	24.701	MM	0.4098	32.06128	1.30389	0.8044
2	28.029	MM	0.6325	3953.86230	104.19036	99.1956

Totals : 3985.92358 105.49425

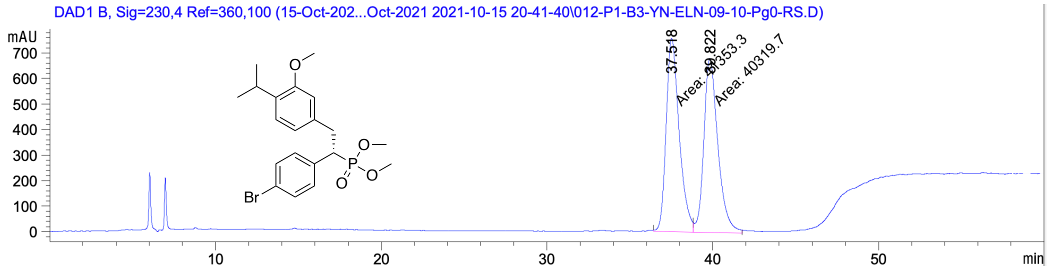

Peak #	RetTime [min]	Type	Width [min]	Area [mAU*s]	Height [mAU]	Area %
1	37.518	MF	0.9105	4.13533e4	756.97668	50.6328
2	39.822	FM	0.9853	4.03197e4	681.99347	49.3672

Totals : 8.16730e4 1438.97015

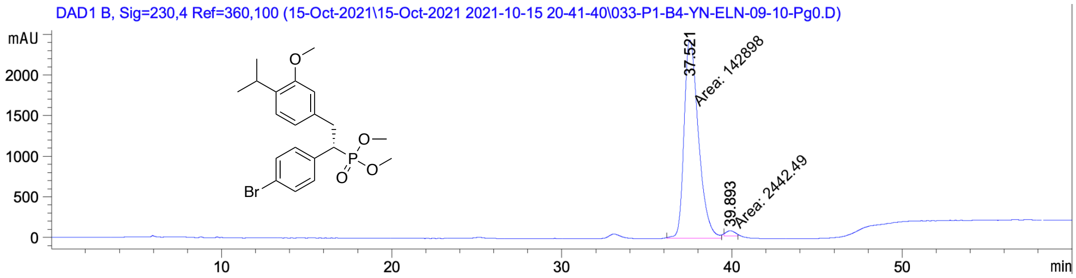

Peak #	RetTime [min]	Type	Width [min]	Area [mAU*s]	Height [mAU]	Area %
1	37.521	MM	0.9765	1.42898e5	2439.02441	98.3195
2	39.893	MM	0.6191	2442.48633	65.75668	1.6805

Totals : 1.45340e5 2504.78109

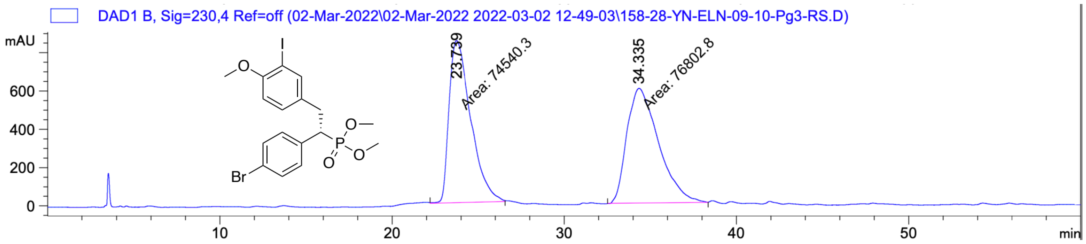

Peak #	RetTime [min]	Type	Width [min]	Area [mAU*s]	Height [mAU]	Area %
1	23.739	MM	1.4669	7.45403e4	846.90216	49.2525
2	34.335	MM	2.1364	7.68028e4	599.16113	50.7475

Totals : 1.51343e5 1446.06329

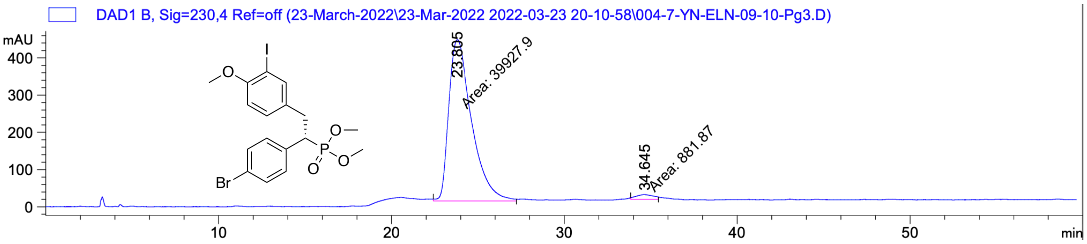

Peak #	RetTime [min]	Type	Width [min]	Area [mAU*s]	Height [mAU]	Area %
1	23.805	MM	1.5338	3.99279e4	433.86063	97.8391
2	34.645	MM	1.1665	881.87042	12.59987	2.1609

Totals : 4.08098e4 446.46049

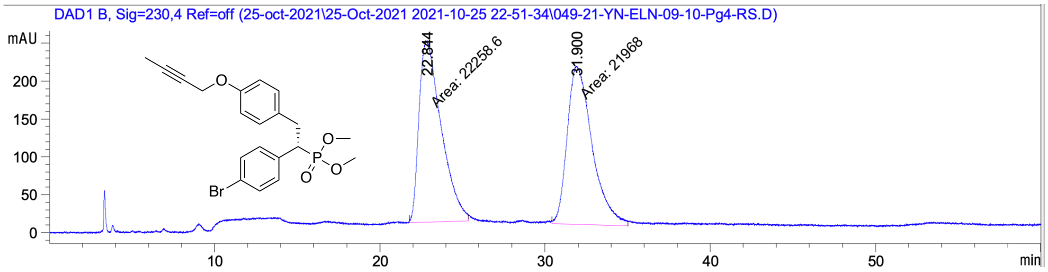

Peak #	RetTime [min]	Type	Width [min]	Area [mAU*s]	Height [mAU]	Area %
1	22.844	MM	1.5467	2.22586e4	239.84695	50.3286
2	31.900	MM	1.7526	2.19680e4	208.91402	49.6714
Totals :				4.42266e4	448.76097	

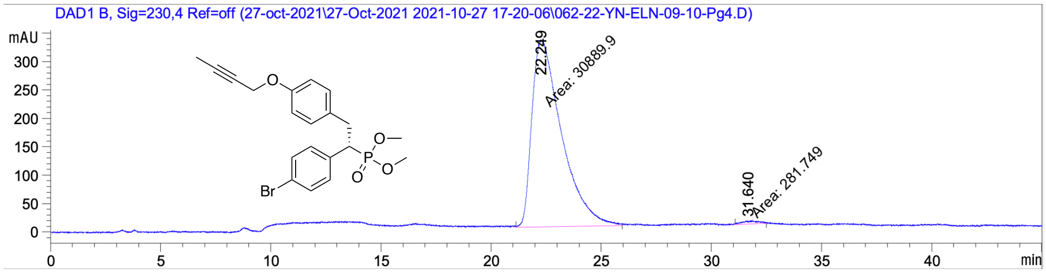

Peak #	RetTime [min]	Type	Width [min]	Area [mAU*s]	Height [mAU]	Area %
1	22.249	MM	1.5584	3.08899e4	330.35114	99.0961
2	31.640	MM	0.7416	281.74908	6.33182	0.9039
Totals :				3.11717e4	336.68296	

SFC

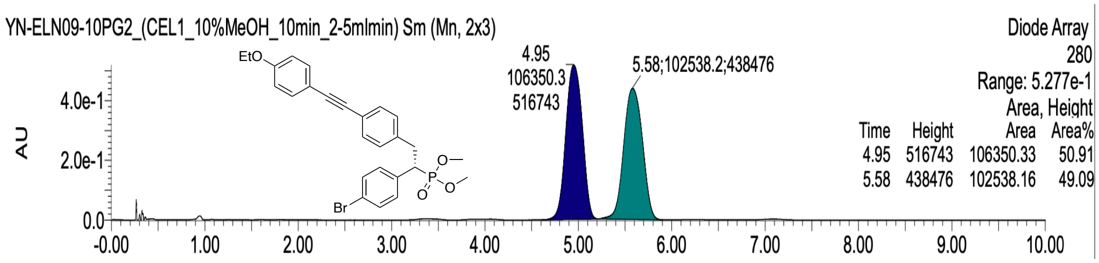
YN-ELN09-10PG2_(CEL1_10%MeOH_10min_2-5mlmin) Sm (Mn, 2x3)
Diode Array
280
Range: 5.277e-1
Area, Height
4.95
106350.3
516743
5.58;102538.2;438476
Time Height Area Area%
4.95 516743 106350.33 50.91
5.58 438476 102538.16 49.09
EtO
Br
AU
4.0e-1
2.0e-1
0.0
-0.00 1.00 2.00 3.00 4.00 5.00 6.00 7.00 8.00 9.00 10.00

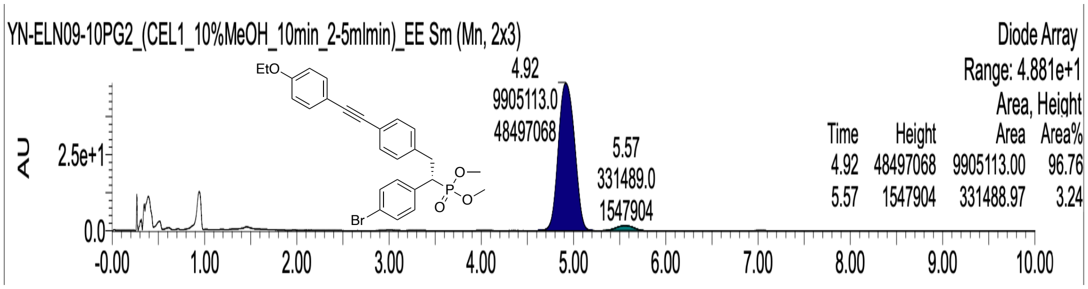
YN-ELN09-10PG2_(CEL1_10%MeOH_10min_2-5mlmin)_EE Sm (Mn, 2x3)
Diode Array
Range: 4.881e+1
Area, Height
4.92
9905113.0
48497068
5.57
331489.0
1547904
Time Height Area Area%
4.92 48497068 9905113.00 96.76
5.57 1547904 331488.97 3.24
EtO
Br
AU
2.5e+1
0.0
-0.00 1.00 2.00 3.00 4.00 5.00 6.00 7.00 8.00 9.00 10.00

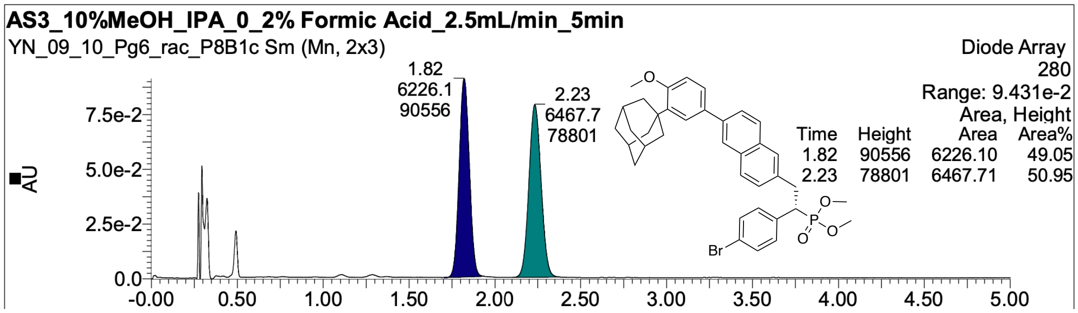
AS3_10%MeOH_IPA_0_2% Formic Acid_2.5mL/min_5min
YN_09_10_Pg6_rac_P8B1c Sm (Mn, 2x3)
Diode Array
280
Range: 9.431e-2
Area, Height
1.82
6226.1
90556
2.23
6467.7
78801
Time Height Area Area%
1.82 90556 6226.10 49.05
2.23 78801 6467.71 50.95
Br
AU
7.5e-2
5.0e-2
2.5e-2
0.0
-0.00 0.50 1.00 1.50 2.00 2.50 3.00 3.50 4.00 4.50 5.00

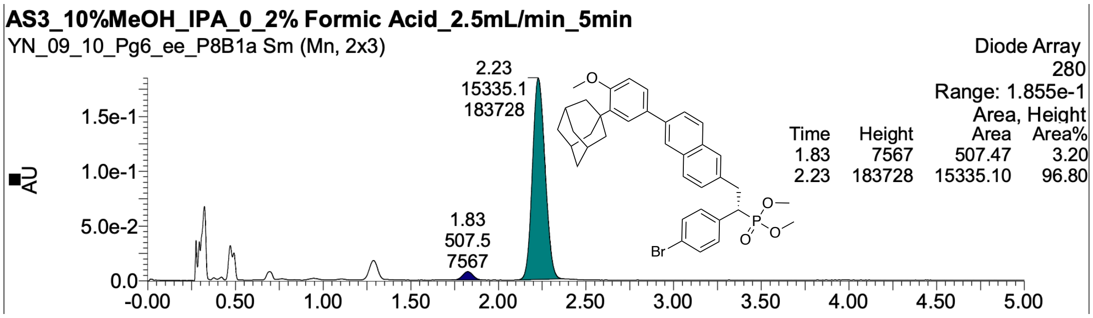
AS3_10%MeOH_IPA_0_2% Formic Acid_2.5mL/min_5min
YN_09_10_Pg6_ee_P8B1a Sm (Mn, 2x3)
Diode Array
280
Range: 1.855e-1
Area, Height
2.23
15335.1
183728
1.83
507.5
7567
Time Height Area Area%
1.83 7567 507.47 3.20
2.23 183728 15335.10 96.80
Br
AU
1.5e-1
1.0e-1
5.0e-2
0.0
-0.00 0.50 1.00 1.50 2.00 2.50 3.00 3.50 4.00 4.50 5.00

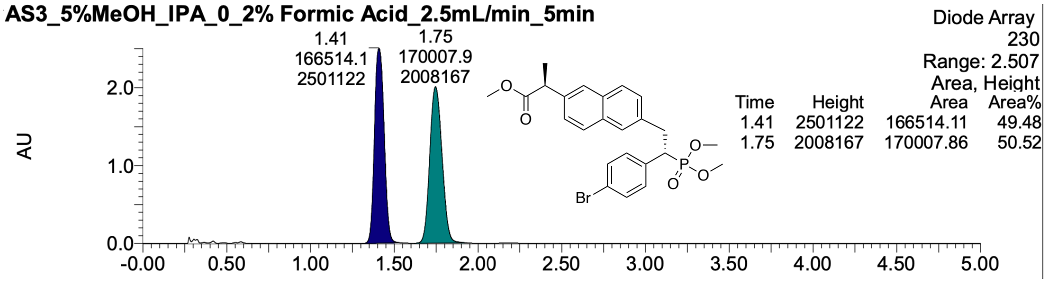
AS3_5%MeOH_IPA_0_2% Formic Acid_2.5mL/min_5min
Diode Array
230
Range: 2.507
Area, Height
Time Height Area Area%
1.41 2501122 166514.11 49.48
1.75 2008167 170007.86 50.52
1.41
166514.1
2501122
1.75
170007.9
2008167
AU
Br

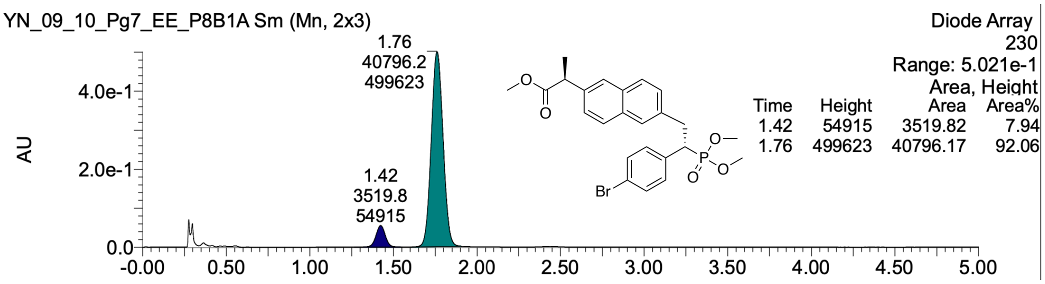
YN_09_10_Pg7_EE_P8B1A Sm (Mn, 2x3)
Diode Array
230
Range: 5.021e-1
Area, Height
Time Height Area Area%
1.42 54915 3519.82 7.94
1.76 499623 40796.17 92.06
1.76
40796.2
499623
1.42
3519.8
54915
AU
Br

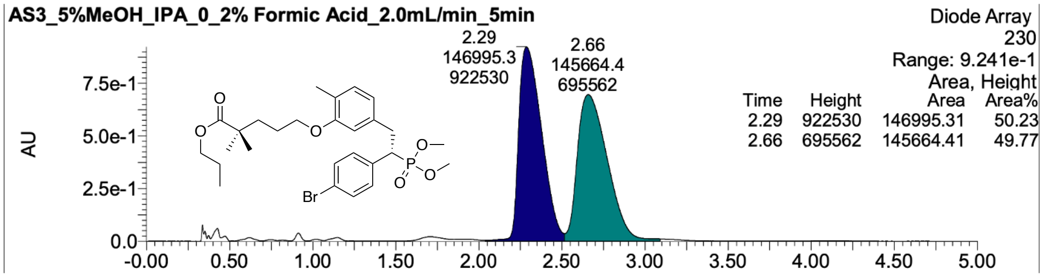
AS3_5%MeOH_IPA_0_2% Formic Acid_2.0mL/min_5min
Diode Array
230
Range: 9.241e-1
Area, Height
Time Height Area Area%
2.29 922530 146995.31 50.23
2.66 695562 145664.41 49.77
2.29
146995.3
922530
2.66
145664.4
695562
AU
Br

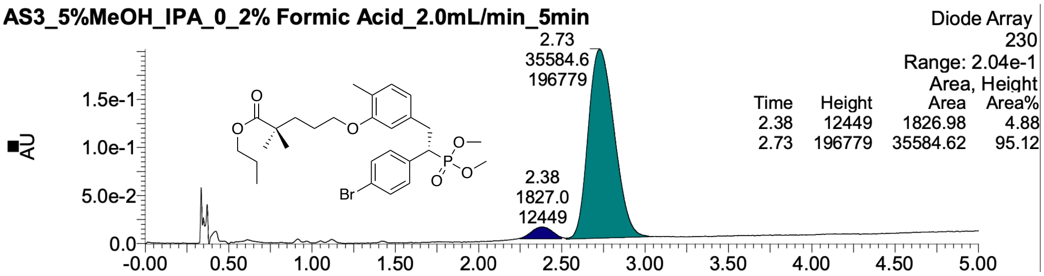
AS3_5%MeOH_IPA_0_2% Formic Acid_2.0mL/min_5min
Diode Array
230
Range: 2.04e-1
Area, Height
Time Height Area Area%
2.38 12449 1826.98 4.88
2.73 196779 35584.62 95.12
2.73
35584.6
196779
2.38
1827.0
12449
AU
Br

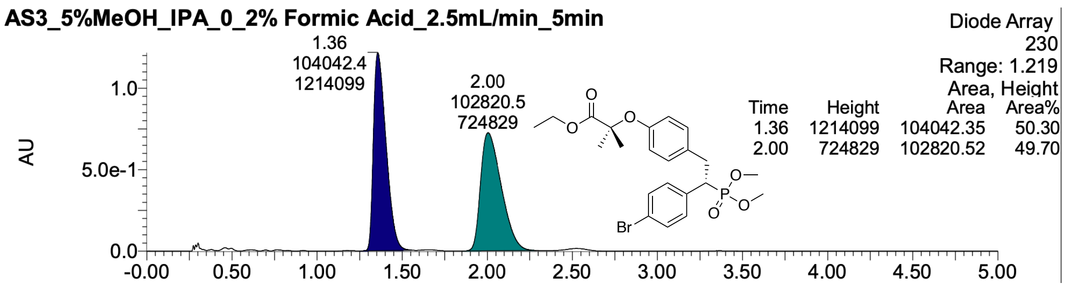

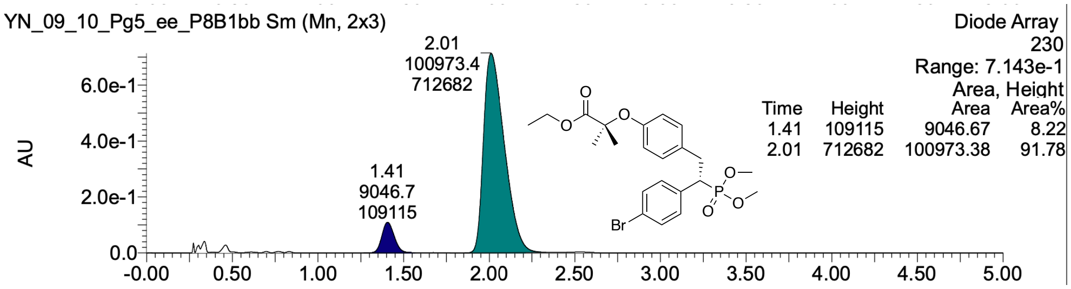

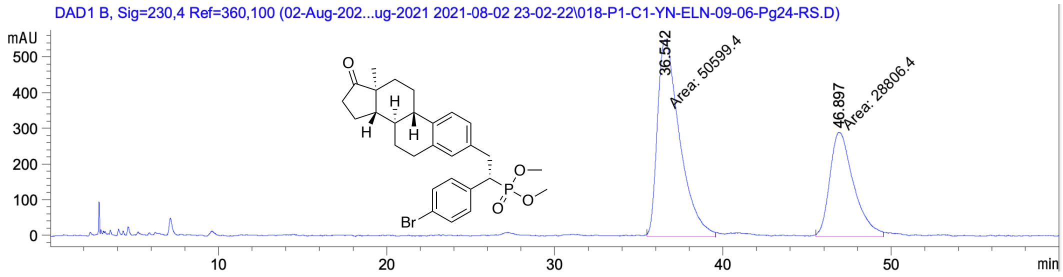

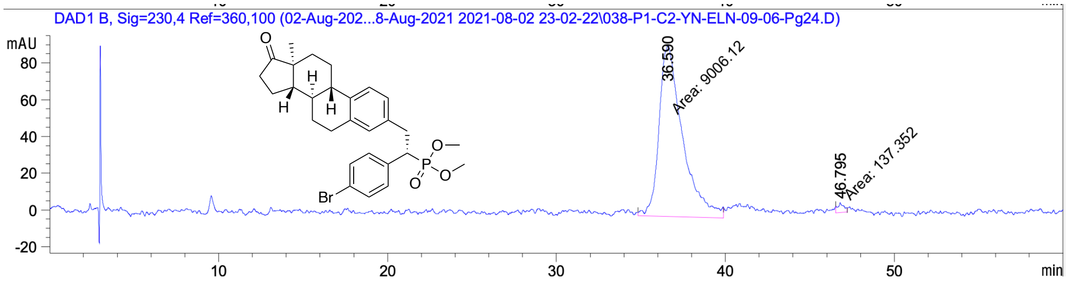

Peak #	RetTime [min]	Type	Width [min]	Area [mAU*s]	Height [mAU]	Area %
1	36.590	MM	1.6095	9006.12012	93.26187	98.4978
2	46.795	MM	0.4318	137.35229	5.30115	1.5022
Totals :				9143.47241	98.56303	

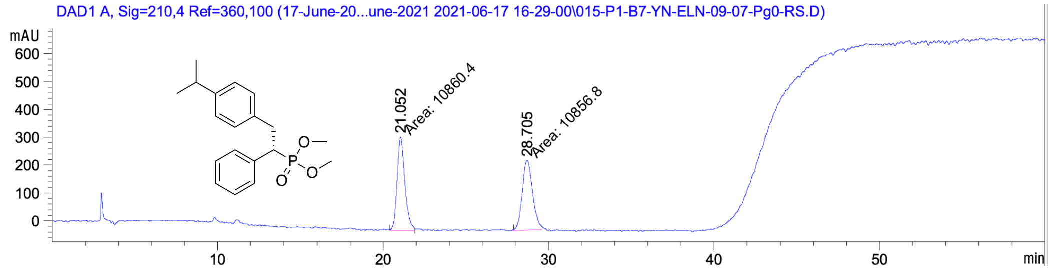

Peak #	RetTime [min]	Type	Width [min]	Area [mAU*s]	Height [mAU]	Area %
1	21.052	MM	0.5436	1.08604e4	332.99515	50.0083
2	28.705	MM	0.7235	1.08568e4	250.09103	49.9917

Totals : 2.17171e4 583.08618

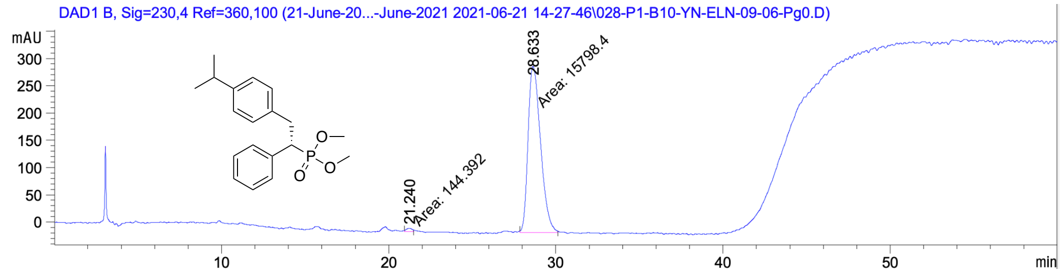

Peak #	RetTime [min]	Type	Width [min]	Area [mAU*s]	Height [mAU]	Area %
1	21.240	MM	0.4317	144.39218	5.57509	0.9057
2	28.633	MM	0.8646	1.57984e4	304.54202	99.0943

Totals : 1.59428e4 310.11711

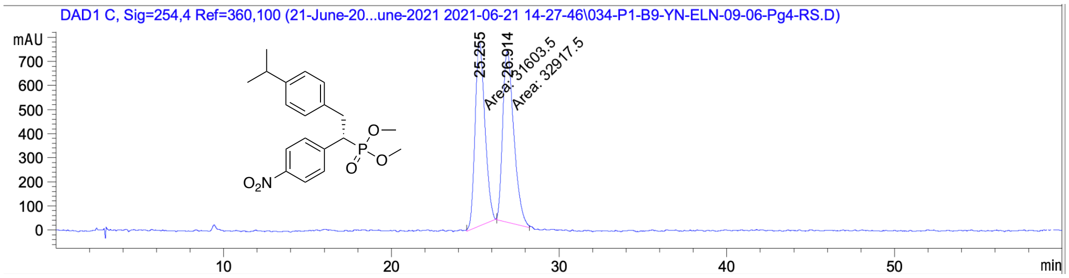

Peak #	RetTime [min]	Type	Width [min]	Area [mAU*s]	Height [mAU]	Area %
1	25.255	MM	0.6873	3.16035e4	766.32025	48.9817
2	26.914	MM	0.7733	3.29175e4	709.44983	51.0183

Totals : 6.45210e4 1475.77008

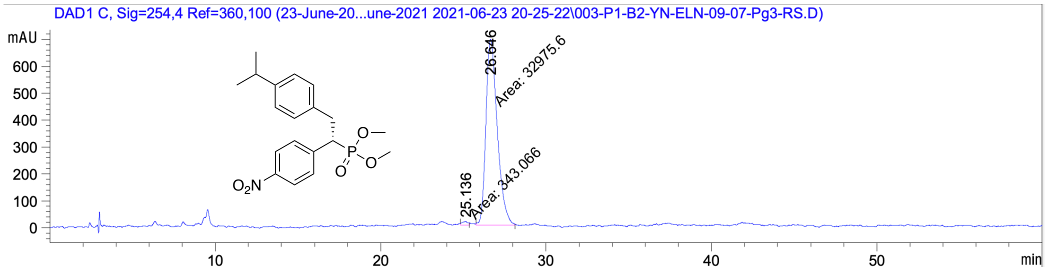

Peak #	RetTime [min]	Type	Width [min]	Area [mAU*s]	Height [mAU]	Area %
1	25.136	MM	0.4261	343.06598	13.41953	1.0297
2	26.646	MM	0.7956	3.29756e4	690.74866	98.9703

Totals : 3.33187e4 704.16819

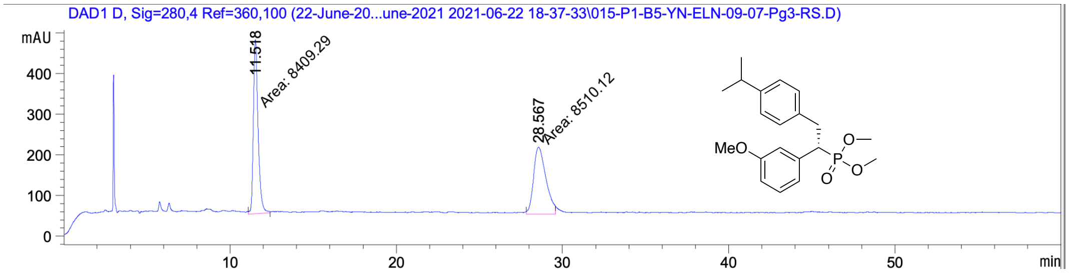

Peak #	RetTime [min]	Type	Width [min]	Area [mAU*s]	Height [mAU]	Area %
1	11.518	MM	0.3274	8409.29492	428.14645	49.7020
2	28.567	MM	0.8622	8510.12402	164.49858	50.2980

Totals : 1.69194e4 592.64503

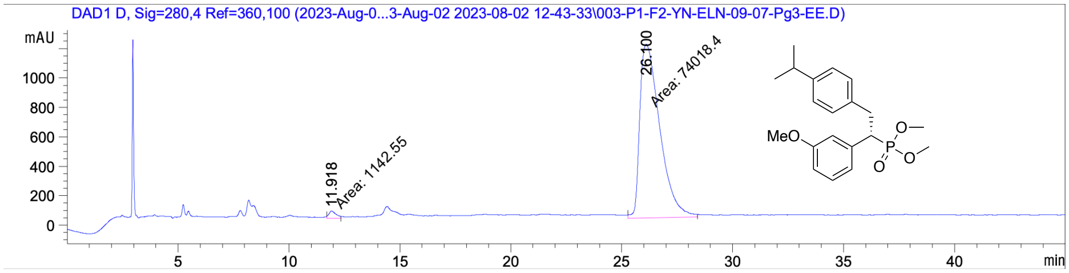

Peak #	RetTime [min]	Type	Width [min]	Area [mAU*s]	Height [mAU]	Area %
1	11.918	MM	0.3863	1142.54785	49.29086	1.5201
2	26.100	MM	1.0459	7.40184e4	1179.53149	98.4799

Totals : 7.51609e4 1228.82236

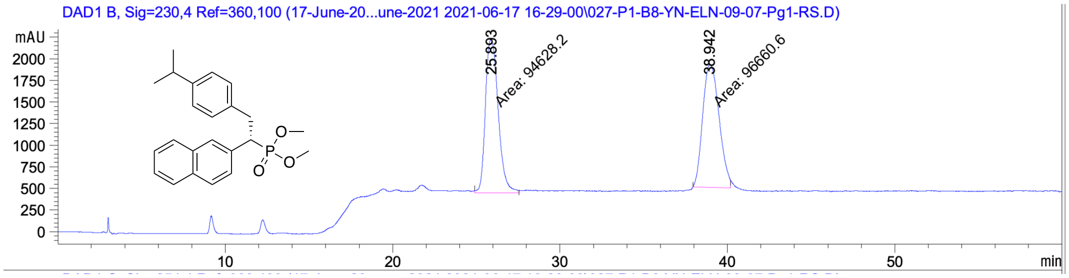

Peak #	RetTime [min]	Type	Width [min]	Area [mAU*s]	Height [mAU]	Area %
1	25.893	MM	0.8840	9.46282e4	1784.02478	49.4687
2	38.942	MM	1.1325	9.66606e4	1422.53210	50.5313

Totals : 1.91289e5 3206.55688

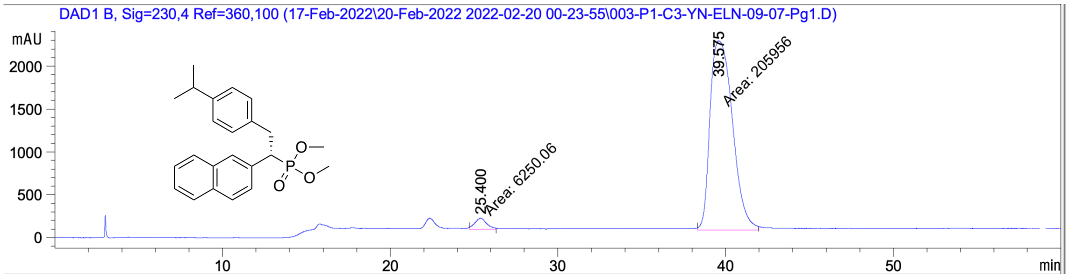

Peak #	RetTime [min]	Type	Width [min]	Area [mAU*s]	Height [mAU]	Area %
1	25.400	MM	0.8248	6250.06104	126.29051	2.9453
2	39.575	MM	1.5548	2.05956e5	2207.67676	97.0547

Totals : 2.12206e5 2333.96727

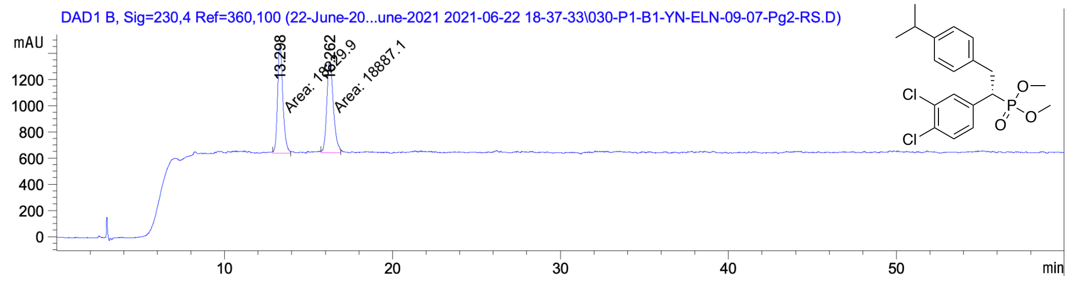

Peak #	RetTime [min]	Type	Width [min]	Area [mAU*s]	Height [mAU]	Area %
1	13.298	MM	0.3642	1.82299e4	834.27338	49.1147
2	16.262	MM	0.4532	1.88871e4	694.51489	50.8853

Totals : 3.71171e4 1528.78827

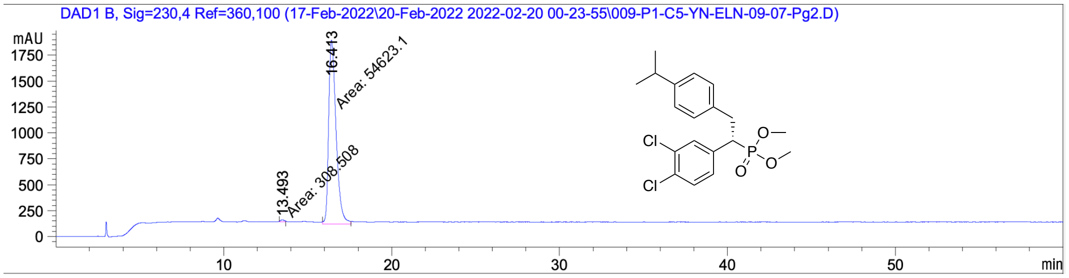

Peak #	RetTime [min]	Type	Width [min]	Area [mAU*s]	Height [mAU]	Area %
1	13.493	MM	0.2854	308.50833	18.01846	0.5616
2	16.413	MM	0.5135	5.46231e4	1772.96472	99.4384

Totals : 5.49316e4 1790.98318

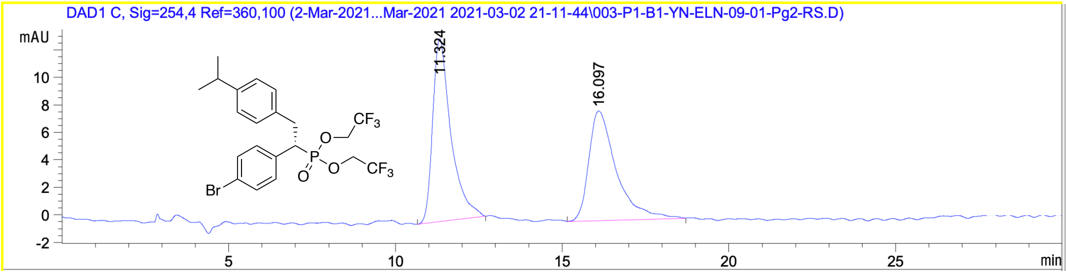

Peak #	RetTime [min]	Type	Width [min]	Area [mAU*s]	Height [mAU]	Area %
1	11.324	BB	0.5649	506.80612	13.28964	52.6348
2	16.097	BB	0.8439	456.06689	7.96670	47.3652

Totals : 962.87302 21.25634

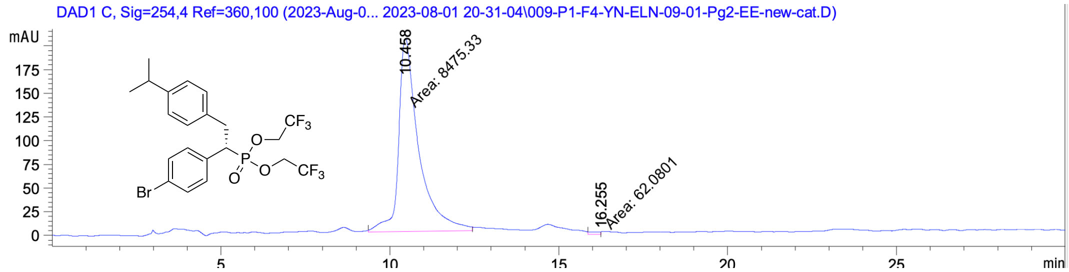

Peak #	RetTime [min]	Type	Width [min]	Area [mAU*s]	Height [mAU]	Area %
1	10.458	MM	0.6945	8475.32617	203.40036	99.2728
2	16.255	MM	0.3564	62.08010	2.90310	0.7272

Totals : 8537.40628 206.30346

10. X-Ray Crystallographic Data for Compound *11f, 11p and 15.*

Crystal Data and Experimental for Compound 8f (CCDC 219499).

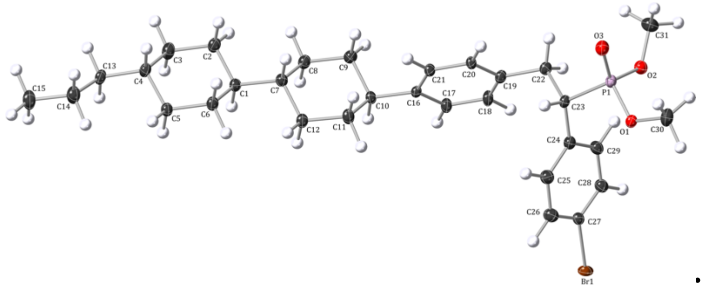

Experimental. Single colorless needle-shaped crystals of compound **11f** were recrystallised from a mixture of DCM and pentane by slow evaporation. A suitable crystal with dimensions 0.23 × 0.03 × 0.02 mm³ was selected and mounted on a loop with paratone on a XtaLAB Synergy, Dualflex, HyPix diffractometer. The crystal was kept at a steady T = 101(1) K during data collection. The structure was solved with the **ShelXT** 2018/2 (Sheldrick, 2018) solution and by using **Olex2** 1.5-alpha (Dolomanov et al., 2009) as the graphical interface. The model was refined with **olex2.refine** 1.5-alpha (Bourhis et al., 2015) using full matrix least squares minimisation on F^2.

Crystal Data. $C_{31}H_{44}BrO_3P$, M_r = 575.570, triclinic, P1 (No. 1), a = 5.48884(19) Å, b = 11.0126(4) Å, c = 24.3359(7) Å, α = 83.978(3)°, β = 85.694(3)°, γ = 83.124(3)°, V = 1449.46(8) Å³, T = 101(1) K, Z = 2, Z' = 2, μ(Cu K$_\alpha$) = 2.678, 15748 reflections measured, 7568 unique (R_{int} = 0.0873) which were used in all calculations. The final wR_2 was 0.1454 (all data) and R_1 was 0.0612 (I≥2 σ(I)).

Table S3. Crystal data and structure refinement for compound 11f

**Compound**	**11f**
Formula	$C_{31}H_{44}BrO_3P$
$D_{calc.}$/ g cm^{-3}	1.319
μ/mm^{-1}	2.678
Formula Weight	575.570
Colour	colourless
Shape	needle-shaped
Size/mm³	0.23×0.03×0.02
T/K	101(1)
Crystal System	triclinic
Flack Parameter	-0.05(3)
Hooft Parameter	-0.05(3)
Space Group	P1
a/Å	5.48884(19)
b/Å	11.0126(4)
c/Å	24.3359(7)
α/°	83.978(3)
β/°	85.694(3)
γ/°	83.124(3)
V/Å³	1449.46(8)
Z	2
Z'	2
Wavelength/Å	1.54184
Radiation type	Cu K$_\alpha$
Θ_{min}/°	3.66
Θ_{max}/°	73.45
Measured Refl's.	15748
Indep't Refl's	7568
Refl's I≥2 σ(I)	6344
R_{int}	0.0873
Parameters	751
Restraints	851
Largest Peak	0.5986
Deepest Hole	-0.3505
GooF	1.0375
wR_2 (all data)	0.1454
wR_2	0.1367
R_1 (all data)	0.0748
R_1	0.0612

Structure Quality Indicators

**Reflections:**	d min (Cu\a) 2Θ=146.9° 0.80	I/σ(I) 9.0	Rint 8.73%	Full 135.4° 94% to 146.9° 99.1
**Refinement:**	Shift -0.007	Max Peak 0.6	Min Peak -0.4	GooF 1.038	Hooft -.05(3)

A colorless needle-shaped-shaped crystal with dimensions 0.23 × 0.03 × 0.02 mm^3 was mounted on a loop with paratone. Data were collected using a XtaLAB Synergy, Dualflex, HyPix diffractometer equipped with an Oxford Cryosystems low-temperature device operating at T = 101(1) K.

Data were measured using ω scans with Cu K_α radiation. The diffraction pattern was indexed and the total number of runs and images was based on the strategy calculation from the program CrysAlisPro 1.171.41.116a (Rigaku OD, 2021). The maximum resolution that was achieved was Θ = 73.45° (0.80 Å).

The unit cell was refined using CrysAlisPro 1.171.41.116a (Rigaku OD, 2021) on 6826 reflections, 43% of the observed reflections.

Data reduction, scaling and absorption corrections were performed using CrysAlisPro 1.171.41.116a (Rigaku OD, 2021). The final completeness is 99.09 % out to 73.45° in Θ. An analytical numeric absorption correction using a multifaceted crystal model based on expressions derived by R.C. Clark & J.S. Reid. (Clark, R. C. & Reid, J. S. (1995). Acta Cryst. A51, 887-897) was performed using CrysAlisPro 1.171.41.108a (Rigaku Oxford Diffraction, 2021). An empirical absorption correction using spherical harmonics, implemented in SCALE3 ABSPACK scaling algorithm was also applied. The absorption coefficient μ of this material is 2.678 mm^{-1} at this wavelength (λ = 1.54184Å) and the minimum and maximum transmissions are 0.654 and 0.947.

The structure was solved and the space group P1 (# 1 determined by the ShelXT 2018/2 (Sheldrick, 2018) structure solution program and refined by full matrix least squares minimisation on $\boldsymbol{F}^2$ using version of **olex2.refine** 1.5-alpha (Bourhis et al., 2015). All non-hydrogen atoms were refined anisotropically. Hydrogen atom positions were calculated geometrically and refined using the riding model. Hydrogen atom positions were calculated geometrically and refined refined using the Hirshfeld model.

Refinement was by using NoSpherA2, an implementation of non-spherical atom-form-factors (F. Kleemiss, H. Puschmann, O. Dolomanov, S.Grabowsky - https://doi.org/10.1039/D0SC05526C – 2020). NoSpherA2 implementation of HAR makes use of tailor-made aspherical atomic form factors calculated from a Hirshfeld-partitioned electron density (ED) not from spherical-atom form factors. The ED was calculated from a Gaussian basis set single determinant SCF wavefunction from DFT using selected functionals for a fragment of this crystal. The following options were used: SOFTWARE: ORCA PARTITIONING: NoSpherA2 INT ACCURACY: Normal METHOD: PBE BASIS SET: def2-SVP CHARGE: 0 MULTIPLICITY: 1 DATE: 2021-10-08_16-37-20

The value of Z' is 2. This means that there are two independent molecules in the asymmetric unit.

The Flack parameter was refined to -0.05(3). Determination of absolute structure using Bayesian statistics

on Bijvoet differences using the Olex2 results in -0.05(3). Note: The Flack parameter is used to determine chirality of the crystal studied, the value should be near 0, a value of 1 means that the stereochemistry is wrong, and the model should be inverted. A value of 0.5 means that the crystal consists of a racemic mixture of the two enantiomers.

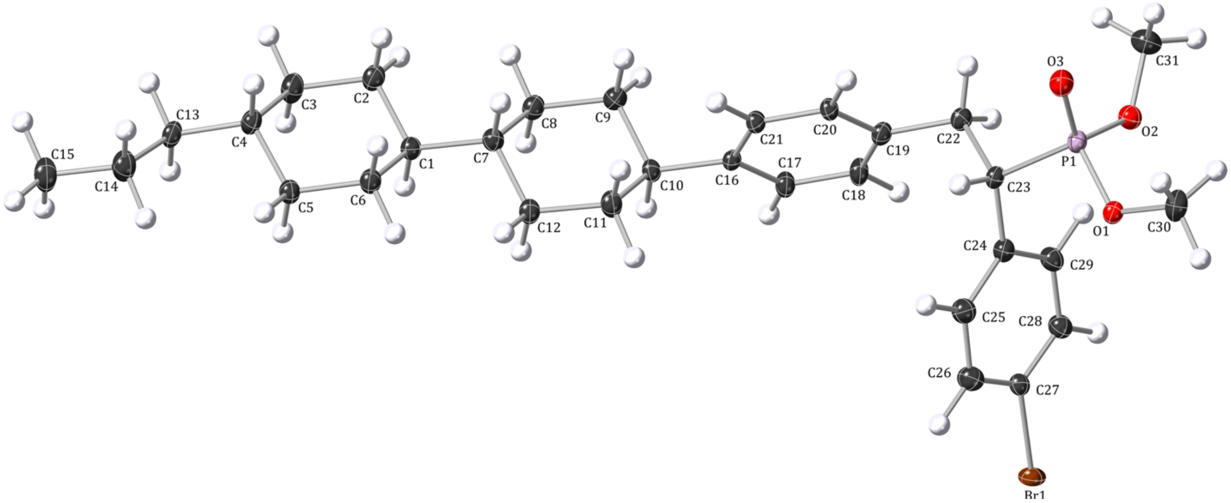

Figure S1: The molecular structure.

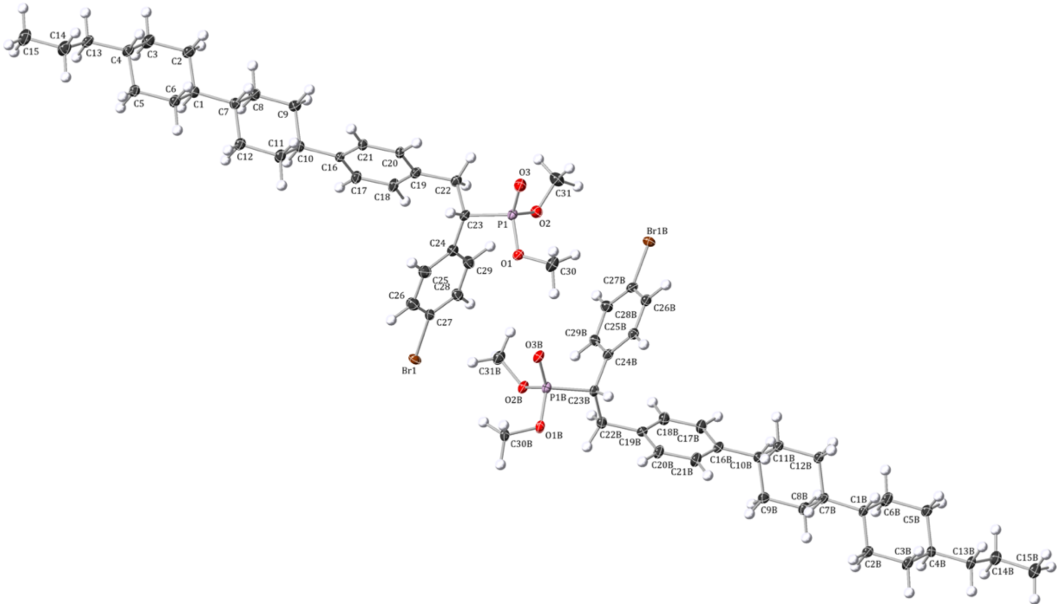

Figure S2: The asymmetric unit consists of two independent molecules.

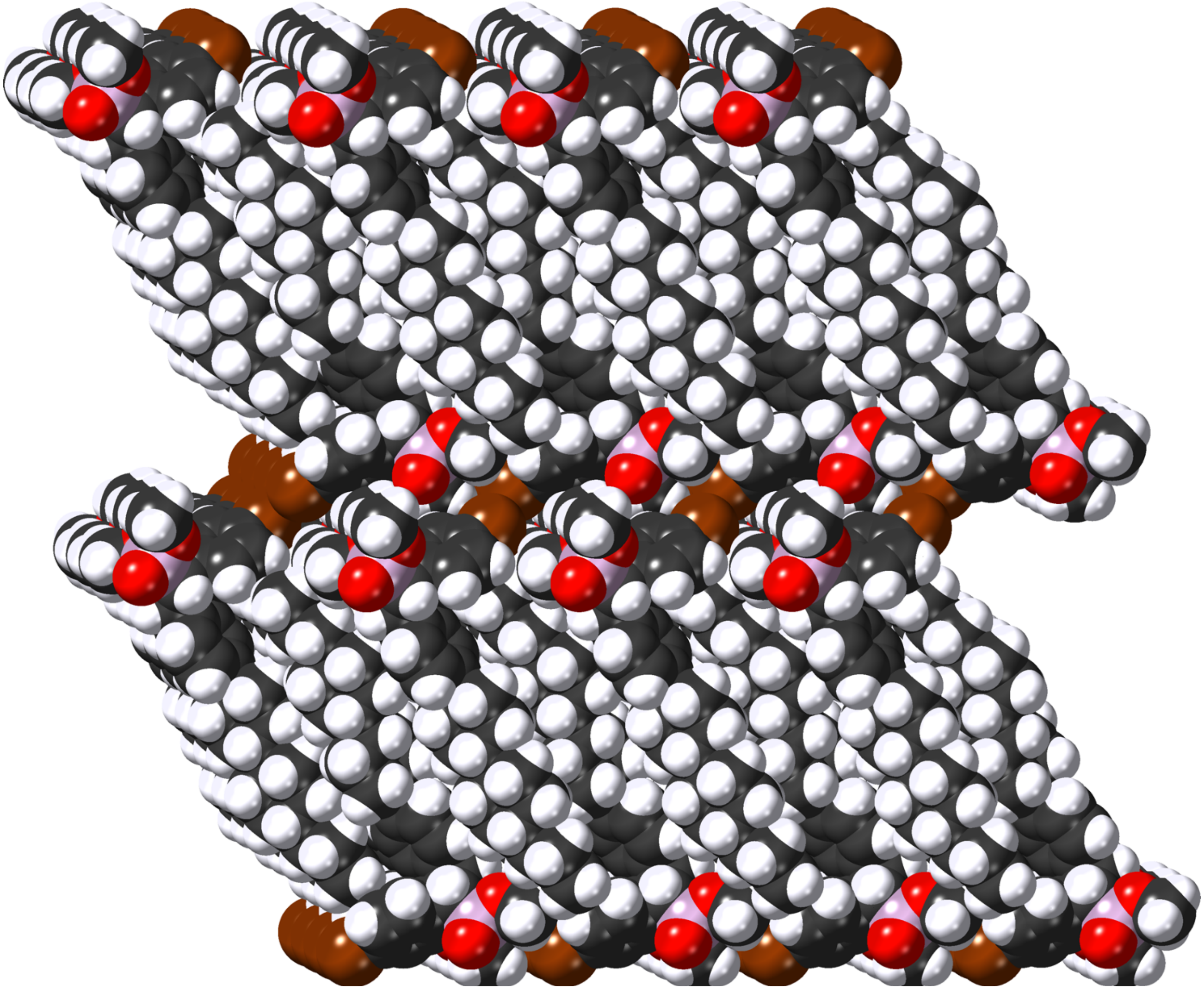

Figure S3: Molecular packing viewed along the a-crystallographic axis. The atoms are shown as spheres with van der Waals radii.

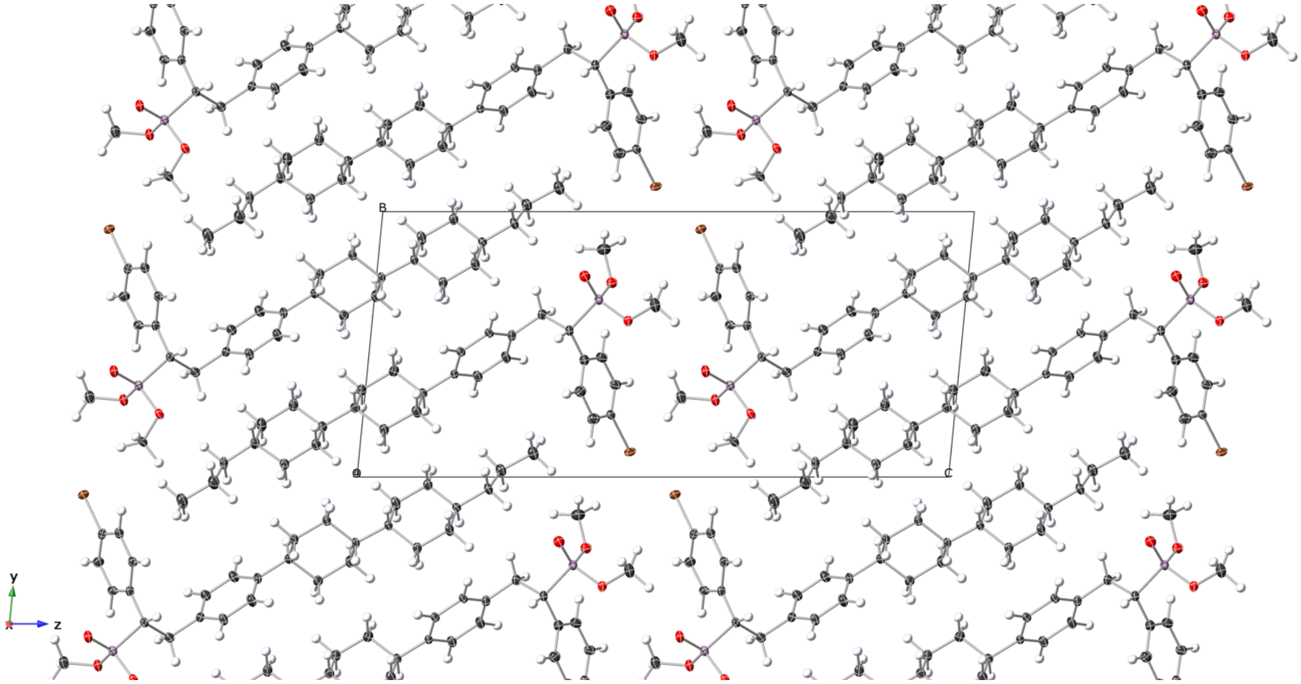

Figure S4: Molecular packing viewed along the a-crystallographic axis.

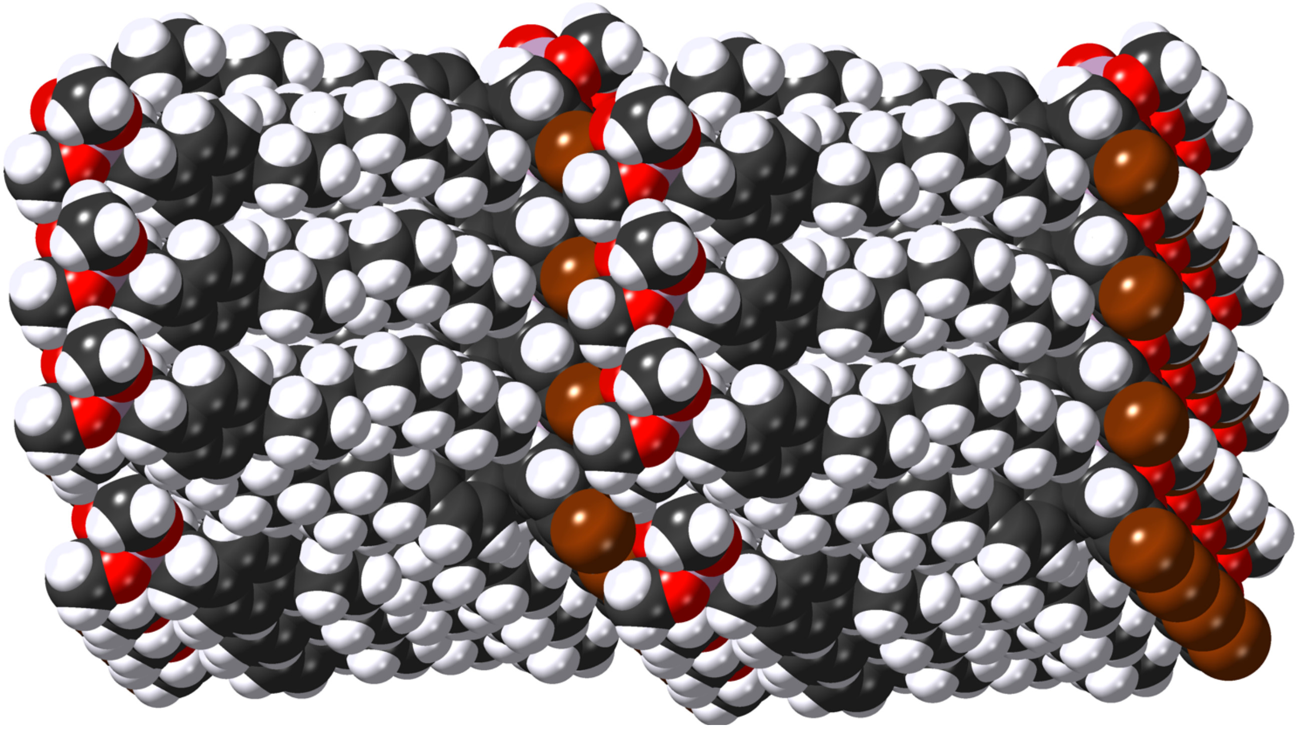

Figure S5: Molecular packing viewed along the b-crystallographic axis. The atoms are shown as spheres with van der Waals radii.

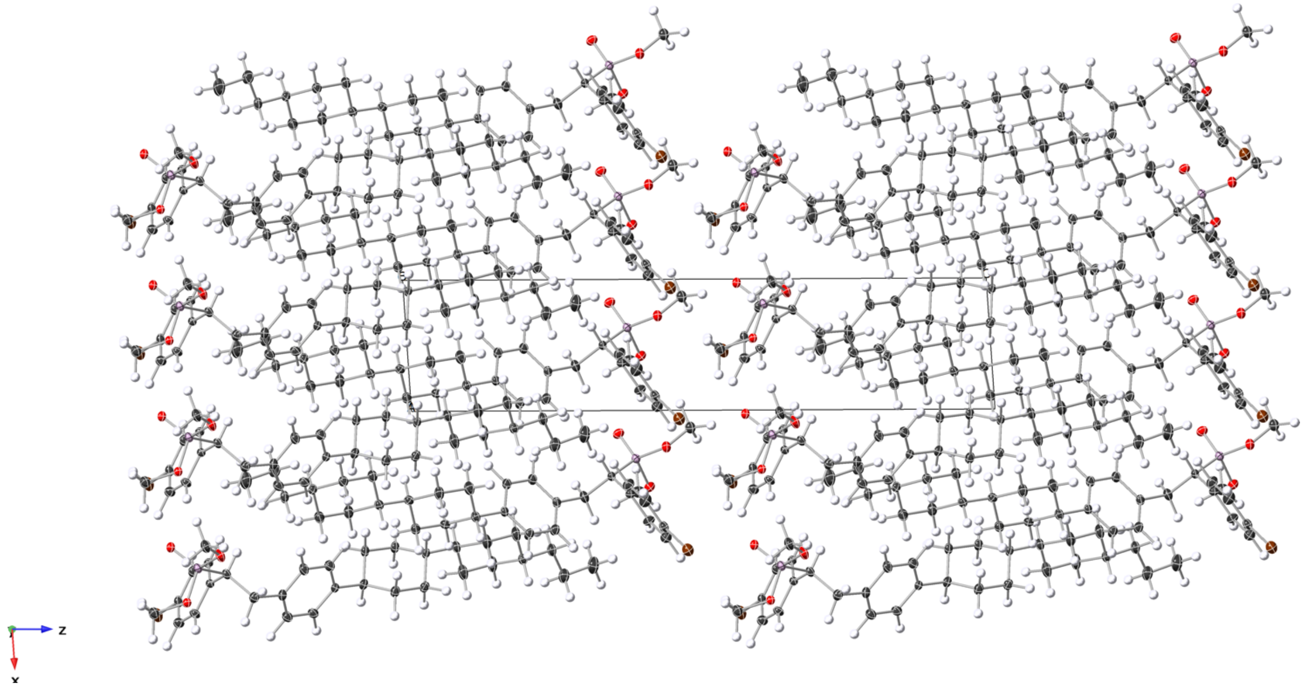

Figure S6: Molecular packing viewed along the b-crystallographic axis.

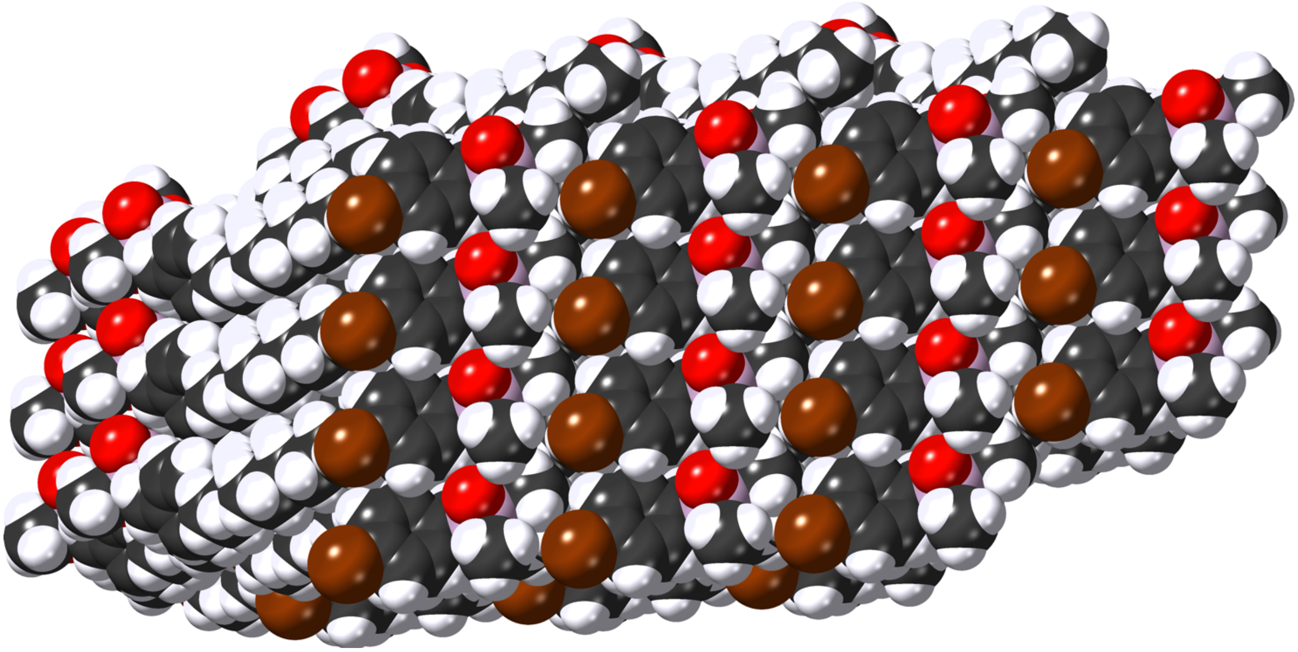

Figure S7: Molecular packing viewed along the c-crystallographic axis. The atoms are shown as spheres with van der Waals radii.

Data Plots: Diffraction Data

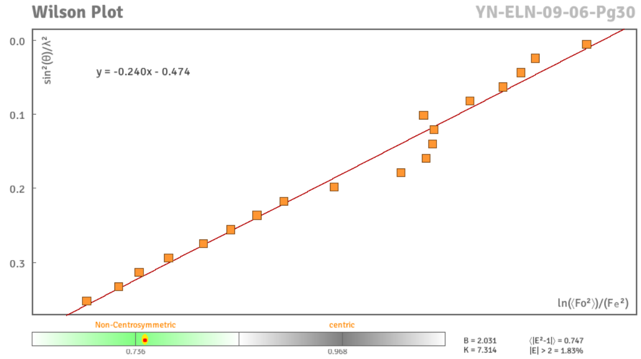

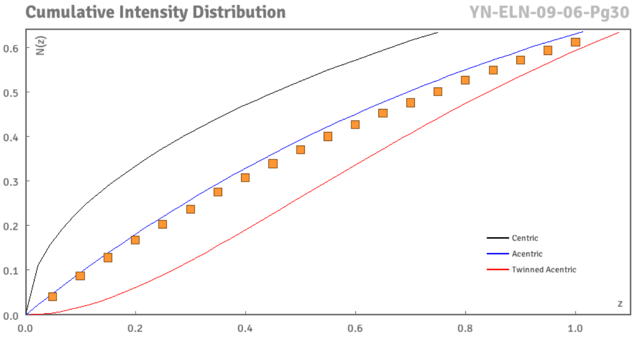

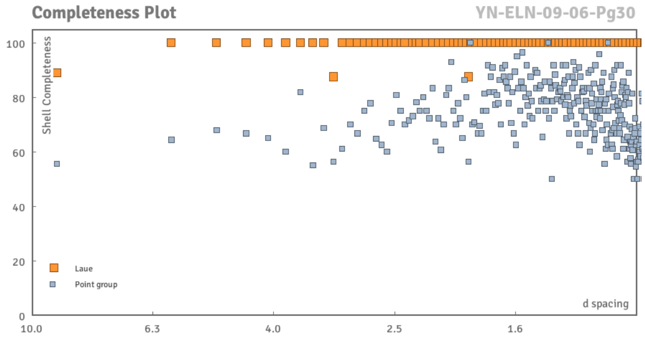

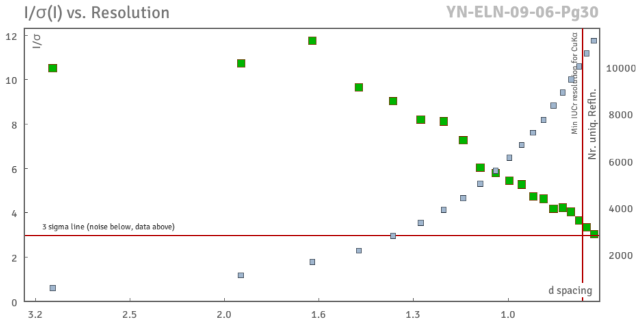

Data Plots: Refinement and Data

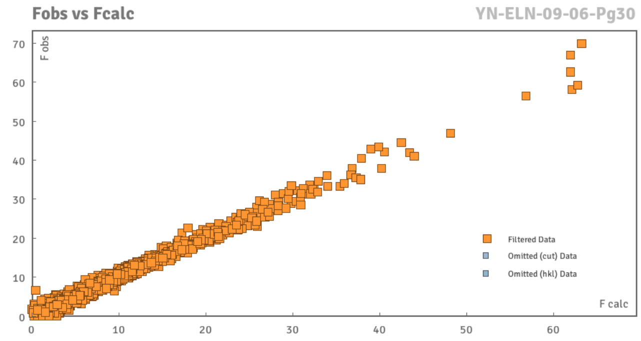

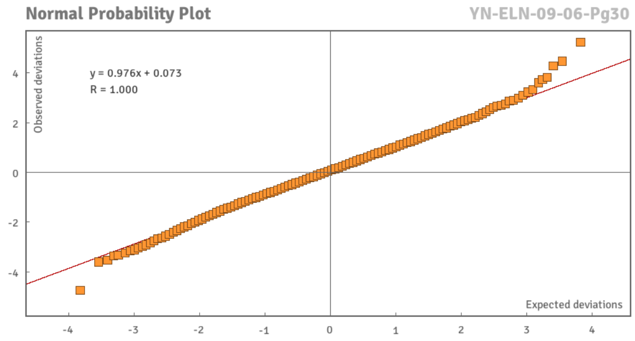

Reflection Statistics

Total reflections (after filtering)	15748	Unique reflections	7568
Completeness	0.648	Mean I/σ	6.98
hkl_{max} collected	(6, 13, 29)	hkl_{min} collected	(-6, -13, -25)
hkl_{max} used	(6, 13, 29)	hkl_{min} used	(-6, -13, -25)
Lim d_{max} collected	100.0	Lim d_{min} collected	0.77
d_{max} used	12.08	d_{min} used	0.8
Friedel pairs	2064	Friedel pairs merged	0
Inconsistent equivalents	2	R_{int}	0.0873
R_{sigma}	0.1106	Intensity transformed	0

Omitted reflections	0	Omitted by user (OMIT hkl)	1
Multiplicity	(3399, 2086, 1050, 526, 276, 128, 70, 24, 8, 1, 1)	Maximum multiplicity	11
Removed systematic absences	0	Filtered off (Shel/OMIT)	0

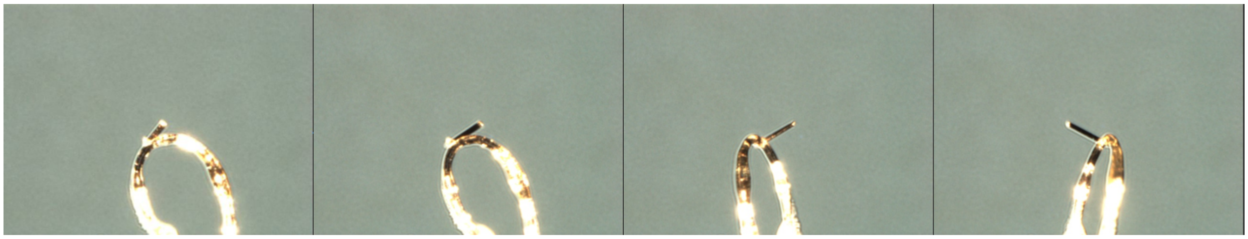

Table S4: Fractional Atomic Coordinates (×10⁴) and Equivalent Isotropic Displacement Parameters (Å²×10³) for compound **11f**. U_{eq} is defined as 1/3 of the trace of the orthogonalised U_{ij}.

Atom	x	y	z	U_{eq}
Br1	10640.1(5)	949.0(5)	4573.9(4)	23.6(6)
C1	7114(8)	1910(4)	-664.2(14)	17.9(12)
O1	2802(6)	5881(3)	4324.2(12)	19.6(9)
P1	3669.8(19)	6685.0(13)	3793.2(7)	16.1(4)
C2	7679(10)	2716(4)	-1193.2(16)	25.4(14)
O2	5835(5)	7327(3)	3988.2(16)	20.7(9)
C3	8046(8)	1987(5)	-1699.8(18)	25.6(15)
O3	1763(5)	7557(3)	3521.7(16)	22.7(10)
C4	5916(8)	1282(4)	-1781.8(16)	19.5(13)
C5	5312(9)	495(4)	-1247.9(16)	19.4(13)
C6	4929(8)	1224(5)	-744.0(18)	21.8(14)
C7	6776(8)	2613(4)	-145.3(14)	17.7(12)
C8	8970(8)	3290(5)	-64.8(17)	20.8(13)
C9	8615(8)	4002(4)	448.2(15)	19.0(12)
C10	8065(7)	3164(4)	977.5(14)	18.1(12)
C11	5885(8)	2472(5)	901.8(18)	25.5(15)
C12	6247(10)	1785(4)	380.5(16)	24.0(14)
C13	6487(8)	527(5)	-2273.6(19)	23.0(15)
C14	4470(9)	-196(6)	-2413(3)	30.3(17)
C15	5209(11)	-898(7)	-2917(3)	40(2)
C16	7669(7)	3888(4)	1478.2(16)	15.5(12)
C17	9401(8)	3803(5)	1873.6(19)	21.5(14)
C18	9084(8)	4512(5)	2320(2)	21.6(13)
C19	6992(7)	5340(4)	2383.3(16)	18.9(12)
C20	5222(7)	5432(5)	1995.0(18)	18.1(13)
C21	5558(7)	4723(5)	1550.3(19)	18.3(13)
C22	6586(8)	6122(4)	2863.9(15)	20.7(13)
C23	5006(7)	5525(3)	3351.0(13)	16.1(11)
C24	6355(7)	4411(3)	3649(2)	17.0(12)
C25	5646(8)	3236(3)	3635(2)	23.3(14)
C26	6879(8)	2195(4)	3903(2)	27.2(15)
C27	8879(7)	2350(3)	4191(2)	18.4(12)
C28	9683(8)	3484(3)	4216(2)	21.8(14)
C29	8396(8)	4513(4)	3941(2)	20.7(13)

Atom	x	y	z	U_{eq}
C30	1318(9)	6480(6)	4757(2)	27.2(15)
C31	6221(10)	8576(4)	3798(3)	27.2(16)
Br1B	5463.8(5)	9336.0(5)	5407.4(4)	24.1(6)
C1B	355(8)	8179(4)	10612.5(14)	18.9(12)
O1B	932(6)	2376(3)	6557.1(14)	22.5(10)
P1B	1900(2)	3448.6(13)	6158.1(6)	16.4(4)
C2B	-26(10)	7323(4)	11135.3(15)	25.7(14)
O2B	4527(5)	2897(3)	5930.8(13)	19.0(9)
C3B	-606(9)	8024(5)	11648.6(18)	26.8(15)
O3B	291(5)	3985(3)	5715.1(13)	19.9(9)
C4B	1306(8)	8858(4)	11735.8(16)	17.7(12)
C5B	1687(10)	9710(4)	11209.8(17)	28.3(16)
C6B	2288(9)	9016(5)	10697.7(19)	30.2(16)
C7B	834(7)	7518(4)	10085.3(13)	16.8(12)
C8B	3392(7)	6817(5)	10022.5(17)	21.9(14)
C9B	3727(9)	6093(4)	9512.2(15)	20.4(13)
C10B	3165(7)	6925(4)	8979.5(14)	18.2(12)
C11B	632(7)	7655(5)	9042.8(18)	19.5(14)
C12B	349(9)	8362(4)	9559.2(15)	20.1(13)
C13B	612(8)	9553(5)	12242.8(19)	22.9(14)
C14B	2594(9)	10252(6)	12421(2)	28.7(16)
C15B	1785(11)	10901(7)	12937(3)	37(2)
C16B	3429(7)	6186(4)	8484.5(16)	16.9(12)
C17B	5357(8)	6261(5)	8082.0(18)	20.5(13)
C18B	5617(8)	5553(4)	7635(2)	20.9(13)
C19B	3966(7)	4721(4)	7579.0(16)	17.5(12)
C20B	2004(8)	4643(5)	7975.1(19)	22.1(14)
C21B	1748(8)	5357(5)	8418(2)	22.4(14)
C22B	4137(8)	3958(4)	7091.0(15)	20.1(13)
C23B	2354(7)	4524(3)	6644.6(13)	17.1(12)
C24B	3081(6)	5709(3)	6349(2)	16.7(12)
C25B	1504(7)	6803(3)	6347(2)	17.5(12)
C26B	2144(7)	7892(4)	6070(2)	19.6(12)
C27B	4427(7)	7857(3)	5786(2)	19.8(12)
C28B	6060(7)	6801(3)	5769(2)	20.7(14)
C29B	5363(7)	5719(4)	6056(2)	18.3(12)
C30B	222(10)	1329(4)	6321(3)	22.5(15)
C31B	5313(9)	3010(6)	5351.8(17)	25.2(14)

Table S5: Anisotropic Displacement Parameters (×10[4]) for compound **11f**. The anisotropic displacement factor exponent takes the form: $-2\pi^2[h^2a^{*2} \times U_{11} + ... + 2hka^* \times b^* \times U_{12}]$

Atom	U_{11}	U_{22}	U_{33}	U_{23}	U_{13}	U_{12}
Br1	29.9(11)	13.7(10)	24.3(12)	3.4(7)	1.6(8)	2.3(8)
C1	19(3)	19(3)	16(2)	-1.4(16)	0.6(14)	-3.5(14)
O1	24(2)	20(2)	15.1(18)	-5.4(14)	-1.1(12)	-1.9(12)
P1	15.4(7)	18.2(8)	14.4(8)	-0.2(6)	-0.2(6)	-3.3(6)
C2	34(3)	28(3)	17(2)	-13(2)	-0.9(16)	-1.7(15)
O2	20(2)	21(2)	22(2)	-3.3(12)	-5.0(14)	-2.7(15)
C3	26(3)	36(3)	19(3)	-17(2)	0.8(18)	-5.2(19)

Atom	U_{11}	U_{22}	U_{33}	U_{23}	U_{13}	U_{12}
O3	19(2)	25(2)	23(2)	5.2(13)	-5.4(14)	-4.6(15)
C4	19(3)	25(3)	15(2)	-5.9(17)	-2.8(15)	-2.3(15)
C5	21(3)	21(3)	17(2)	-5.7(18)	-0.7(16)	-3.5(15)
C6	26(3)	23(3)	17(3)	-7.4(18)	0.5(17)	-3.7(18)
C7	16(2)	18(3)	18(2)	0.5(15)	0.0(14)	-3.7(14)
C8	22(3)	24(3)	17(3)	-5.0(17)	-0.5(16)	-2.3(17)
C9	16(3)	22(3)	19(2)	-4.6(18)	-0.5(15)	-0.8(15)
C10	23(3)	17(3)	15(2)	-2.9(16)	1.5(14)	-5.0(13)
C11	34(3)	26(3)	19(3)	-12.3(19)	1.3(17)	-6.0(19)
C12	33(3)	23(3)	17(2)	-9(2)	-0.8(16)	-3.9(15)
C13	29(3)	28(3)	13(3)	-8(2)	-0.7(18)	-3.2(18)
C14	35(3)	36(4)	24(3)	-15(2)	-4(2)	-7(2)
C15	58(5)	41(5)	25(4)	-16(3)	-3(3)	-12(3)
C16	19(2)	16(2)	12(2)	0.2(15)	-0.3(13)	-2.2(14)
C17	24(3)	22(3)	18(3)	3.5(18)	-6.2(15)	-6.5(17)
C18	23(3)	26(3)	17(3)	-0.4(17)	-4.3(16)	-8.0(17)
C19	21(2)	21(3)	14(2)	-1.7(16)	-0.1(14)	-2.3(15)
C20	22(3)	16(3)	15(2)	1.9(18)	-1.0(15)	-5.2(16)
C21	22(3)	17(3)	15(3)	2.4(16)	-1.4(15)	-4.1(16)
C22	26(3)	21(3)	15(2)	-5.1(17)	3.7(15)	-2.4(14)
C23	17(2)	20(2)	12(2)	-2.9(13)	0.1(13)	-2.4(12)
C24	14(2)	21(2)	17(3)	-3.1(12)	-0.6(15)	-2.7(14)
C25	25(3)	23(2)	24(3)	-6.9(14)	-6(2)	-2.3(15)
C26	32(3)	22(3)	28(3)	-4.5(15)	-6.3(18)	-0.9(17)
C27	22(2)	16(2)	16(3)	-0.2(13)	3.1(16)	-3.9(14)
C28	23(3)	19(2)	23(4)	-0.1(14)	-2.6(19)	-3.2(15)
C29	19(2)	19(3)	25(3)	-1.8(14)	-6.4(17)	-4.2(17)
C30	26(3)	37(4)	20(3)	-4(2)	-0.1(19)	-8(2)
C31	24(3)	22(3)	35(4)	-2.8(17)	-1(3)	-0.7(19)
Br1B	34.9(12)	15.0(10)	21.7(12)	-4.9(8)	1.4(8)	2.2(8)
C1B	26(3)	19(3)	13(2)	-6.0(17)	1.4(14)	-4.1(14)
O1B	32(2)	20.7(19)	16(2)	-5.2(13)	5.3(14)	-7.2(12)
P1B	17.2(7)	18.0(8)	14.2(9)	-0.9(6)	1.0(6)	-4.5(6)
C2B	42(4)	23(3)	15(2)	-14(2)	0.6(17)	-2.7(15)
O2B	16.6(18)	26(2)	14.1(19)	1.5(12)	-1.8(11)	-6.0(15)
C3B	38(3)	30(3)	15(3)	-18(2)	2.5(18)	-6.0(18)
O3B	19.1(19)	27(2)	14(2)	-1.9(15)	-1.9(12)	-5.0(14)
C4B	23(3)	15(3)	15(2)	-2.9(17)	0.3(15)	-4.0(15)
C5B	49(4)	22(3)	16(3)	-14(2)	-1.5(17)	-2.6(15)
C6B	45(3)	33(3)	17(3)	-22(2)	3.6(19)	-7.5(19)
C7B	19(3)	20(3)	12(2)	-0.5(16)	-3.8(13)	-3.4(13)
C8B	22(3)	26(3)	17(3)	4.5(18)	-5.1(16)	-4.0(18)
C9B	24(3)	20(3)	17(2)	2.3(18)	-4.3(15)	-1.6(15)
C10B	22(2)	18(3)	15(2)	-1.9(15)	-4.1(13)	-2.8(13)
C11B	24(3)	21(3)	13(3)	0.8(17)	-2.7(15)	-3.0(17)
C12B	25(3)	20(3)	14(2)	4.4(18)	-3.2(15)	-3.7(15)
C13B	30(3)	25(3)	15(3)	-3(2)	0.1(18)	-8.2(18)
C14B	39(3)	29(4)	21(3)	-8(2)	-5(2)	-7(2)
C15B	56(5)	34(4)	23(3)	-2(3)	-7(3)	-9(2)
C16B	16(2)	22(3)	13(2)	-2.4(15)	-2.6(14)	-3.7(14)
C17B	17(2)	25(3)	21(3)	-5.7(17)	1.2(15)	-8.0(16)
C18B	21(3)	22(3)	20(3)	-6.0(17)	2.2(17)	-5.7(17)

Atom	U_{11}	U_{22}	U_{33}	U_{23}	U_{13}	U_{12}
C19B	23(2)	17(3)	13(2)	-2.3(15)	-2.2(14)	-0.2(15)
C20B	27(3)	26(3)	16(3)	-11.0(18)	1.2(16)	-7.3(17)
C21B	22(3)	30(3)	19(3)	-8.8(18)	0.8(17)	-9.5(18)
C22B	23(3)	19(3)	18(2)	0.3(17)	-3.0(15)	-4.1(14)
C23B	19(3)	18(2)	14(2)	2.1(13)	2.6(13)	-4.7(12)
C24B	15(2)	21(2)	13(3)	1.3(12)	-0.2(15)	-2.4(13)
C25B	17(2)	19(2)	16(3)	1.7(13)	-1.9(17)	-3.4(15)
C26B	22(2)	21(3)	16(3)	-1.4(14)	-1.7(16)	-3.6(16)
C27B	25(2)	21(2)	13(3)	0.5(12)	-0.1(16)	0.2(14)
C28B	19(3)	20(2)	22(3)	-1.2(14)	-0.6(18)	-1.2(16)
C29B	16(2)	16(3)	20(3)	3.3(14)	4.2(16)	-2.3(17)
C30B	31(4)	18(3)	19(4)	-2.6(19)	3(3)	-7.6(19)
C31B	26(3)	31(4)	19(2)	1(3)	0.5(15)	-6.3(18)

Table S6: Bond Lengths in Å for compound **11f**.

Atom	Atom	Length/Å
Br1	C27	1.913(3)
C1	C2	1.519(3)
C1	C6	1.526(3)
C1	C7	1.538(3)
O1	P1	1.565(3)
O1	C30	1.448(3)
P1	O2	1.579(2)
P1	O3	1.478(2)
P1	C23	1.810(3)
C2	C3	1.529(3)
O2	C31	1.440(3)
C3	C4	1.515(3)
C4	C5	1.522(3)
C4	C13	1.520(3)
C5	C6	1.523(3)
C7	C8	1.525(3)
C7	C12	1.522(3)
C8	C9	1.531(3)
C9	C10	1.537(3)
C10	C11	1.526(3)
C10	C16	1.513(3)
C11	C12	1.533(3)
C13	C14	1.516(3)
C14	C15	1.521(3)
C16	C17	1.391(3)
C16	C21	1.403(3)
C17	C18	1.392(3)
C18	C19	1.388(3)
C19	C20	1.393(3)
C19	C22	1.512(3)
C20	C21	1.389(3)
C22	C23	1.552(3)
C23	C24	1.502(3)

Atom	Atom	Length/Å
C24	C25	1.400(3)
C24	C29	1.392(3)
C25	C26	1.385(3)
C26	C27	1.383(3)
C27	C28	1.382(3)
C28	C29	1.397(3)
Br1B	C27B	1.911(3)
C1B	C2B	1.519(3)
C1B	C6B	1.525(3)
C1B	C7B	1.532(3)
O1B	P1B	1.566(3)
O1B	C30B	1.446(3)
P1B	O2B	1.580(2)
P1B	O3B	1.475(3)
P1B	C23B	1.810(3)
C2B	C3B	1.530(3)
O2B	C31B	1.439(3)
C3B	C4B	1.516(3)
C4B	C5B	1.522(3)
C4B	C13B	1.520(3)
C5B	C6B	1.524(3)
C7B	C8B	1.524(3)
C7B	C12B	1.522(3)
C8B	C9B	1.533(3)
C9B	C10B	1.536(3)
C10B	C11B	1.526(3)
C10B	C16B	1.512(3)
C11B	C12B	1.535(3)
C13B	C14B	1.519(3)
C14B	C15B	1.520(3)
C16B	C17B	1.391(3)
C16B	C21B	1.402(3)
C17B	C18B	1.392(3)
C18B	C19B	1.387(3)
C19B	C20B	1.395(3)
C19B	C22B	1.516(3)
C20B	C21B	1.389(3)
C22B	C23B	1.549(3)
C23B	C24B	1.505(3)
C24B	C25B	1.396(3)
C24B	C29B	1.394(3)
C25B	C26B	1.383(3)
C26B	C27B	1.383(3)
C27B	C28B	1.383(3)
C28B	C29B	1.396(3)

Table S7: Bond Angles in ° for compound **11f**.

Atom	Atom	Atom	Angle/°
C6	C1	C2	109.0(3)

Atom	Atom	Atom	Angle/°
C7	C1	C2	113.3(3)
C7	C1	C6	113.3(3)
C30	O1	P1	118.7(3)
O2	P1	O1	103.9(2)
O3	P1	O1	116.60(19)
O3	P1	O2	113.4(2)
C23	P1	O1	101.75(17)
C23	P1	O2	107.84(18)
C23	P1	O3	112.2(2)
C3	C2	C1	112.1(3)
C31	O2	P1	122.4(3)
C4	C3	C2	114.2(3)
C5	C4	C3	109.2(3)
C13	C4	C3	110.9(3)
C13	C4	C5	112.4(3)
C6	C5	C4	113.0(3)
C5	C6	C1	113.1(3)
C8	C7	C1	113.2(3)
C12	C7	C1	112.5(3)
C12	C7	C8	108.7(3)
C9	C8	C7	113.0(3)
C10	C9	C8	111.8(3)
C11	C10	C9	109.7(3)
C16	C10	C9	111.1(3)
C16	C10	C11	112.5(3)
C12	C11	C10	112.4(3)
C11	C12	C7	113.2(3)
C14	C13	C4	116.3(3)
C15	C14	C13	112.1(4)
C17	C16	C10	122.3(3)
C21	C16	C10	120.8(3)
C21	C16	C17	116.8(3)
C18	C17	C16	122.2(3)
C19	C18	C17	120.2(3)
C20	C19	C18	118.6(3)
C22	C19	C18	121.6(3)
C22	C19	C20	119.8(3)
C21	C20	C19	120.7(3)
C20	C21	C16	121.4(3)
C23	C22	C19	111.8(3)
C22	C23	P1	109.7(2)
C24	C23	P1	114.6(3)
C24	C23	C22	113.0(3)
C25	C24	C23	121.6(3)
C29	C24	C23	120.7(3)
C29	C24	C25	117.7(3)
C26	C25	C24	122.4(3)
C27	C26	C25	117.5(3)
C26	C27	Br1	119.4(3)
C28	C27	Br1	117.8(2)
C28	C27	C26	122.7(3)
C29	C28	C27	118.2(3)

Atom	Atom	Atom	Angle/°
C28	C29	C24	121.4(3)
C6B	C1B	C2B	109.4(3)
C7B	C1B	C2B	113.7(3)
C7B	C1B	C6B	114.3(3)
C30B	O1B	P1B	118.7(3)
O2B	P1B	O1B	104.44(19)
O3B	P1B	O1B	116.10(19)
O3B	P1B	O2B	113.1(2)
C23B	P1B	O1B	101.14(17)
C23B	P1B	O2B	107.39(18)
C23B	P1B	O3B	113.49(19)
C3B	C2B	C1B	112.1(3)
C31B	O2B	P1B	122.7(3)
C4B	C3B	C2B	113.7(3)
C5B	C4B	C3B	109.0(3)
C13B	C4B	C3B	111.5(3)
C13B	C4B	C5B	112.6(3)
C6B	C5B	C4B	112.7(3)
C5B	C6B	C1B	112.9(3)
C8B	C7B	C1B	114.2(3)
C12B	C7B	C1B	113.3(3)
C12B	C7B	C8B	109.1(3)
C9B	C8B	C7B	112.5(3)
C10B	C9B	C8B	111.7(3)
C11B	C10B	C9B	110.5(3)
C16B	C10B	C9B	110.8(3)
C16B	C10B	C11B	112.4(3)
C12B	C11B	C10B	112.0(3)
C11B	C12B	C7B	112.1(3)
C14B	C13B	C4B	115.6(3)
C15B	C14B	C13B	112.6(4)
C17B	C16B	C10B	122.5(3)
C21B	C16B	C10B	120.9(3)
C21B	C16B	C17B	116.5(3)
C18B	C17B	C16B	122.1(3)
C19B	C18B	C17B	120.9(3)
C20B	C19B	C18B	117.9(3)
C22B	C19B	C18B	122.6(3)
C22B	C19B	C20B	119.4(3)
C21B	C20B	C19B	120.9(3)
C20B	C21B	C16B	121.7(3)
C23B	C22B	C19B	112.0(3)
C22B	C23B	P1B	112.6(2)
C24B	C23B	P1B	111.2(3)
C24B	C23B	C22B	112.9(3)
C25B	C24B	C23B	121.8(3)
C29B	C24B	C23B	119.6(3)
C29B	C24B	C25B	118.6(3)
C26B	C25B	C24B	122.2(3)
C27B	C26B	C25B	117.1(3)
C26B	C27B	Br1B	119.4(2)
C28B	C27B	Br1B	117.2(2)

Atom	Atom	Atom	Angle/˚
C28B	C27B	C26B	123.4(3)
C29B	C28B	C27B	118.1(3)
C28B	C29B	C24B	120.7(3)

Table S8: Torsion Angles in ˚ for compound **11f**.

Atom	Atom	Atom	Atom	Angle/˚
Br1	C27	C26	C25	179.5(5)
Br1	C27	C28	C29	-179.6(4)
C1	C2	C3	C4	55.2(5)
C1	C6	C5	C4	-55.6(5)
C1	C7	C8	C9	179.3(4)
C1	C7	C12	C11	-179.5(4)
O1	P1	O2	C31	-142.3(4)
P1	C23	C22	C19	-159.6(3)
P1	C23	C24	C25	120.0(4)
P1	C23	C24	C29	-61.4(4)
C2	C3	C4	C5	-52.4(5)
C2	C3	C4	C13	-176.8(4)
C3	C4	C5	C6	52.1(4)
C3	C4	C13	C14	-178.2(5)
C4	C13	C14	C15	178.9(6)
C7	C8	C9	C10	56.4(4)
C7	C12	C11	C10	-55.5(4)
C8	C9	C10	C11	-53.8(4)
C8	C9	C10	C16	-178.9(3)
C9	C10	C11	C12	53.4(4)
C9	C10	C16	C17	-106.9(5)
C9	C10	C16	C21	70.2(4)
C10	C16	C17	C18	177.0(5)
C10	C16	C21	C20	-177.1(5)
C16	C17	C18	C19	-0.1(7)
C16	C21	C20	C19	0.4(7)
C17	C18	C19	C20	0.7(7)
C17	C18	C19	C22	179.8(5)
C18	C19	C20	C21	-0.8(7)
C18	C19	C22	C23	-94.9(5)
C19	C22	C23	C24	71.2(4)
C22	C23	C24	C25	-113.4(4)
C22	C23	C24	C29	65.2(4)
C23	C24	C25	C26	179.4(5)
C23	C24	C29	C28	-179.4(5)
C24	C25	C26	C27	-0.1(7)
C24	C29	C28	C27	0.2(7)
C25	C26	C27	C28	-0.5(8)
C26	C27	C28	C29	0.5(7)
Br1B	C27B	C26B	C25B	-178.6(5)
Br1B	C27B	C28B	C29B	178.3(5)
C1B	C2B	C3B	C4B	-55.5(5)

Atom	Atom	Atom	Atom	Angle/˚
C1B	C6B	C5B	C4B	55.8(5)
C1B	C7B	C8B	C9B	175.8(4)
C1B	C7B	C12B	C11B	-175.1(4)
O1B	P1B	O2B	C31B	132.2(4)
P1B	C23B	C22B	C19B	-164.2(3)
P1B	C23B	C24B	C25B	110.7(4)
P1B	C23B	C24B	C29B	-67.6(4)
C2B	C3B	C4B	C5B	53.7(5)
C2B	C3B	C4B	C13B	178.7(4)
C3B	C4B	C5B	C6B	-53.5(4)
C3B	C4B	C13B	C14B	170.4(5)
C4B	C13B	C14B	C15B	-178.8(5)
C7B	C8B	C9B	C10B	55.6(5)
C7B	C12B	C11B	C10B	-56.4(4)
C8B	C9B	C10B	C11B	-52.9(4)
C8B	C9B	C10B	C16B	-178.2(3)
C9B	C10B	C11B	C12B	53.4(4)
C9B	C10B	C16B	C17B	-108.6(4)
C9B	C10B	C16B	C21B	69.6(5)
C10B	C16B	C17B	C18B	178.3(5)
C10B	C16B	C21B	C20B	-178.0(5)
C16B	C17B	C18B	C19B	-1.1(7)
C16B	C21B	C20B	C19B	0.3(7)
C17B	C18B	C19B	C20B	1.8(7)
C17B	C18B	C19B	C22B	178.2(5)
C18B	C19B	C20B	C21B	-1.4(6)
C18B	C19B	C22B	C23B	-98.6(5)
C19B	C22B	C23B	C24B	68.9(4)
C22B	C23B	C24B	C25B	-121.6(4)
C22B	C23B	C24B	C29B	60.0(4)
C23B	C24B	C25B	C26B	-179.1(5)
C23B	C24B	C29B	C28B	178.7(5)
C24B	C25B	C26B	C27B	0.6(7)
C24B	C29B	C28B	C27B	0.1(7)
C25B	C26B	C27B	C28B	-0.1(7)
C26B	C27B	C28B	C29B	-0.3(7)

Table S9: Hydrogen Fractional Atomic Coordinates (×10^4) and Equivalent Isotropic Displacement Parameters (Å^2×10^3) for **compound 11f.** U_{eq} is defined as 1/3 of the trace of the orthogonalised U_{ij}.

Atom	x	y	z	U_{eq}
H1	8686(9)	1225(4)	-608.4(14)	21.5(14)
H2a	6181(10)	3442(4)	-1253.7(16)	30.5(17)
H2b	9328(10)	3141(4)	-1151.7(16)	30.5(17)
H3a	8337(8)	2617(5)	-2065(2)	30.8(18)
H3b	9693(9)	1345(5)	-1660.9(18)	30.8(18)
H4	4329(9)	1940(4)	-1867.7(16)	23.4(15)
H5a	3659(9)	75(4)	-1291.6(16)	23.2(16)
H5b	6795(9)	-236(4)	-1181.3(16)	23.2(16)

Atom	x	y	z	U_{eq}
H6a	4607(8)	601(5)	-377.8(19)	26.1(17)
H6b	3305(8)	1880(5)	-787.8(18)	26.1(17)
H7	5197(9)	3296(4)	-196.4(14)	21.2(14)
H8a	9279(8)	3925(5)	-427.4(19)	24.9(16)
H8b	10594(8)	2631(5)	-28.9(18)	24.9(16)
H9a	10263(9)	4426(4)	491.0(15)	22.9(15)
H9b	7109(9)	4727(4)	394.8(15)	22.9(15)
H10	9655(8)	2492(4)	1038.6(14)	21.7(14)
H11a	5624(8)	1820(5)	1261(2)	30.6(19)
H11b	4238(9)	3118(5)	875.9(18)	30.6(19)
H12a	7760(10)	1062(4)	430.1(16)	28.8(17)
H12b	4606(10)	1355(4)	337.1(16)	28.8(17)
H13a	8111(9)	-112(5)	-2194.5(19)	27.6(17)
H13b	6939(8)	1142(5)	-2634(2)	27.6(17)
H14a	4039(9)	-838(6)	-2061(3)	36(2)
H14b	2829(9)	430(6)	-2492(3)	36(2)
H15a	5479(11)	-254(7)	-3278(3)	60(3)
H15b	6903(11)	-1490(7)	-2848(3)	60(3)
H15c	3770(11)	-1450(7)	-2983(3)	60(3)
H17	11041(8)	3168(5)	1832.9(19)	25.8(16)
H18	10469(8)	4418(5)	2618(2)	25.9(16)
H20	3574(8)	6061(5)	2040.2(18)	21.8(15)
H21	4167(8)	4815(5)	1254(2)	21.9(15)
H22a	8349(9)	6253(4)	3007.1(15)	24.9(16)
H22b	5674(8)	7017(4)	2725.3(15)	24.9(16)
H23	3603(7)	5231(3)	3183.0(13)	19.3(14)
H25	4085(9)	3136(3)	3407(2)	28.0(17)
H26	6301(8)	1300(4)	3887(2)	32.6(18)
H28	11257(9)	3572(3)	4441(2)	26.1(17)
H29	8997(8)	5405(4)	3954(2)	24.8(16)
H30a	-488(10)	6805(6)	4614(2)	41(2)
H30b	1141(9)	5832(6)	5120(2)	41(2)
H30c	2190(9)	7250(6)	4860(2)	41(2)
H31a	7133(10)	8608(4)	3387(3)	41(2)
H31b	4466(10)	9140(4)	3786(3)	41(2)
H31c	7357(10)	8920(4)	4079(3)	41(2)
H1B	-1357(9)	8770(4)	10566.1(14)	22.7(15)
H2Ba	-1525(10)	6793(4)	11088.4(15)	30.8(17)
H2Bb	1619(10)	6686(4)	11192.2(15)	30.8(17)
H3Ba	-760(9)	7367(5)	12009.9(19)	32.1(18)
H3Bb	-2372(9)	8572(5)	11612.1(18)	32.1(18)
H4B	3025(9)	8286(4)	11804.9(16)	21.2(15)
H5Ba	36(11)	10340(4)	11151.0(17)	33.9(19)
H5Bb	3174(11)	10247(4)	11257.6(17)	33.9(19)
H6Ba	2443(9)	9673(5)	10337(2)	36(2)
H6Bb	4052(10)	8467(5)	10735.4(19)	36(2)
H7B	-461(8)	6842(4)	10112.7(13)	20.1(14)
H8Ba	4752(8)	7461(5)	9990.4(17)	26.3(16)
H8Bb	3707(7)	6186(5)	10389.4(19)	26.3(16)
H9Ba	5602(9)	5658(4)	9474.7(15)	24.4(15)
H9Bb	2517(9)	5376(4)	9565.5(15)	24.4(15)
H10B	4514(8)	7577(4)	8915.8(14)	21.8(14)

Atom	x	y	z	U_{eq}
H11c	349(7)	8299(5)	8679.1(19)	23.4(17)
H11d	-765(8)	7032(5)	9070.5(18)	23.4(17)
H12c	-1498(10)	8831(4)	9595.8(15)	24.1(16)
H12d	1621(9)	9051(4)	9512.0(15)	24.1(16)
H13c	-998(9)	10199(5)	12160.0(19)	27.5(17)
H13d	102(8)	8903(5)	12585(2)	27.5(17)
H14c	4221(9)	9617(6)	12501(2)	34.4(19)
H14d	3078(9)	10926(6)	12086(3)	34.4(19)
H15d	191(11)	11558(7)	12855(3)	56(3)
H15e	1320(11)	10231(7)	13275(3)	56(3)
H15f	3268(11)	11376(7)	13047(3)	56(3)
H17B	6691(8)	6886(5)	8117.2(18)	24.6(16)
H18B	7121(8)	5653(4)	7329(2)	25.1(16)
H20B	669(8)	4020(5)	7937.1(19)	26.5(17)
H21B	218(8)	5272(5)	8718(2)	26.9(17)
H22c	6003(9)	3888(4)	6908.1(16)	24.1(15)
H22d	3712(8)	3039(4)	7235.8(16)	24.1(15)
H23B	720(7)	4729(3)	6844.0(13)	20.5(14)
H25B	-269(8)	6797(3)	6568(2)	21.0(15)
H26B	916(7)	8731(4)	6074(2)	23.6(15)
H28B	7819(8)	6813(3)	5542(2)	24.9(16)
H29B	6599(7)	4883(4)	6051(2)	22.0(15)
H30d	1452(10)	1128(4)	5964(3)	34(2)
H30e	325(10)	539(4)	6627(3)	34(2)
H30f	-1644(10)	1536(4)	6194(3)	34(2)
H31d	4734(9)	2263(6)	5155.7(17)	38(2)
H31e	4499(9)	3878(6)	5157.4(17)	38(2)
H31f	7298(10)	2973(6)	5306.6(17)	38(2)

Crystal Data and Experimental for Compound 11p (CCDC 2194598).

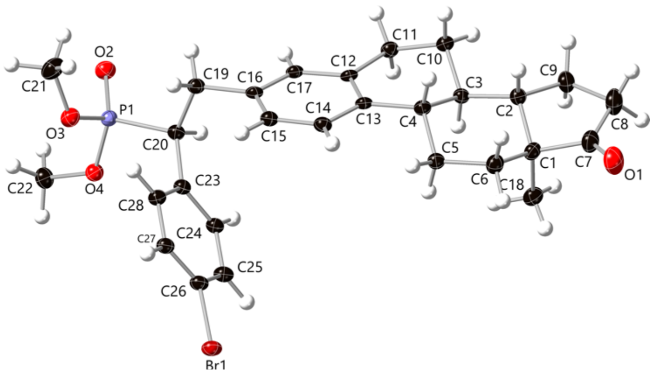

Experimental. Single colorless needle-shaped crystals of compound **11p** recrystallized from THF by slow evaporation. A suitable crystal with dimensions $0.28 \times 0.04 \times 0.02$ mm^3 was selected and mounted on a loop with paratone on a XtaLAB Synergy-S diffractometer. The crystal was kept at a steady T = 100.00(10) K during data collection. The structure was solved with the **ShelXT** (Sheldrick, 2015) solution program using iterative methods and by using **Olex2** 1.3-alpha (Dolomanov et al., 2009) as the graphical interface. The model was refined with **ShelXL** 2018/3 (Sheldrick, 2015) using full matrix least squares minimisation on $\boldsymbol{F^2}$.

Crystal Data. $C_{28}H_{34}BrO_4P$, M_r = 545.43, monoclinic, $P2_1$ (No. 4), a = 16.8546(7) Å, b = 6.1111(2) Å, c = 26.2684(10) Å, β = 105.468(4)°, α = γ = 90°, V = 2607.66(18) Å^3, T = 100.01(10) K, Z = 4, Z' = 2, μ(Cu K$_\alpha$) = 2.981, 29380 reflections measured, 9667 unique (R_{int} = 0.0715) which were used in all calculations. The final wR_2 was 0.1083 (all data) and R_1 was 0.0446 (I≥2 σ(I)).

Table S10. Crystal data and structure refinement for compound **11p**.

**Compound**	**11p**
Formula	$C_{28}H_{34}BrO_4P$
$D_{calc.}$/ g cm^{-3}	1.389
μ/mm^{-1}	2.981
Formula Weight	545.43
Color	colorless
Shape	needle-shaped
Size/mm^3	0.27×0.05×0.03
T/K	100.01(10)
Crystal System	monoclinic
Flack Parameter	-0.021(16)
Hooft Parameter	-0.000(12)
Space Group	$P2_1$
a/Å	16.8546(7)
b/Å	6.1111(2)
c/Å	26.2684(10)
α/°	90
β/°	105.468(4)
γ/°	90
V/Å^3	2607.66(18)
Z	4
Z'	2
Wavelength/Å	1.54184
Radiation type	Cu K$_\alpha$
Θ_{min}/°	2.720
Θ_{max}/°	72.957
Measured Refl's.	29380
Indep't Refl's	9667
Refl's I≥2 σ(I)	8623
R_{int}	0.0715
Parameters	692
Restraints	660
Largest Peak	0.448
Deepest Hole	-0.637
GooF	1.096
wR_2 (all data)	0.1083
wR_2	0.1044
R_1 (all data)	0.0524
R_1	0.0446

Structure Quality Indicators

**Reflections:**	d min (Cu\a) 2θ=145.9° 0.81	I/σ(I) 12.9	Rint 7.15%	CAP 130.0° 95% to 145.9° 98.8	
**Refinement:**	Shift -0.001	Max Peak 0.5	Min Peak -0.6	GooF 1.096	Flack -.021(16)

A colourless needle-shaped-shaped crystal with dimensions 0.27 × 0.05 × 0.03 mm^3 was mounted on a loop with paratone. Data were collected using a XtaLAB Synergy, Dualflex, HyPix diffractometer equipped with an Oxford Cryosystems low-temperature device operating at T = 100.01(10) K.

Data were measured using ω scans using Cu K_α radiation. The diffraction pattern was indexed and the total number of runs and images was based on the strategy calculation from the program CrysAlisPro 1.171.41.108a (Rigaku OD, 2021). The maximum resolution that was achieved was Θ = 72.957° (0.81 Å).

The unit cell was refined using CrysAlisPro 1.171.41.108a (Rigaku OD, 2021) on 5002 reflections, 17% of the observed reflections.

Data reduction, scaling and absorption corrections were performed using CrysAlisPro 1.171.41.108a (Rigaku OD, 2021). The final completeness is 98.30 % out to 72.957° in Θ. A Gaussian absorption correction using a multifaceted crystal model based on expressions derived by R.C. Clark & J.S. Reid. (Clark, R. C. & Reid, J. S. (1995) was performed using CrysAlisPro 1.171.41.98a (Rigaku Oxford Diffraction, 2021). An empirical absorption correction using spherical harmonics, implemented in SCALE3 ABSPACK scaling algorithm was also performed. The absorption coefficient μ of this material is 2.981 mm^{-1} at this wavelength (λ = 1.54184Å) and the minimum and maximum transmissions are 0.700 and 0.961.

The structure was solved and the space group $P2_1$ (# 4) determined with the **ShelXT** (Sheldrick, 2015) solution program using iterative methods and refined by full matrix least squares minimisation on $\boldsymbol{F^2}$ using version 2018/3 of **ShelXL** 2018/3 (Sheldrick, 2015). All non-hydrogen atoms were refined anisotropically. Most hydrogen atom positions were calculated geometrically and refined using the riding model, but some hydrogen atoms were refined freely.

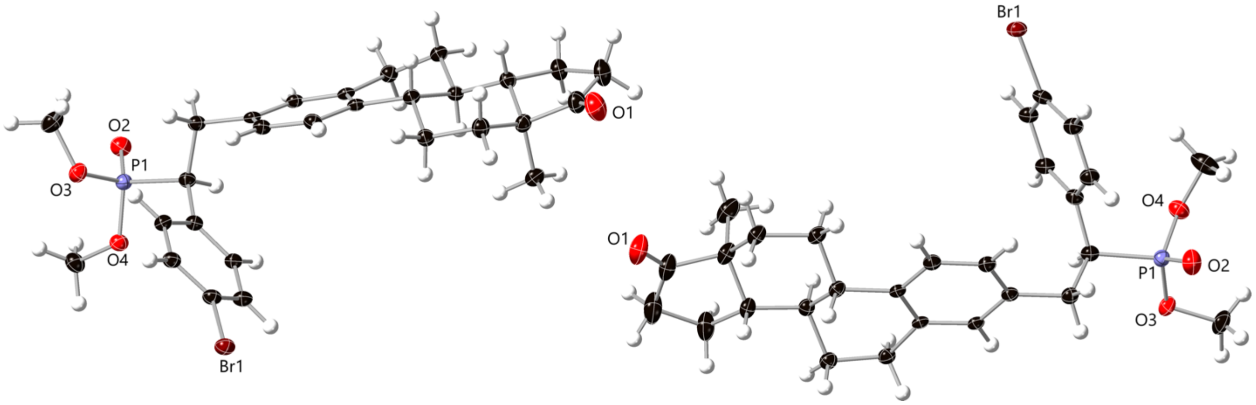

Figure S8: There are two molecules in the asymmetric unit that are rotamers of the $PO(OCH_3)_2$ group.

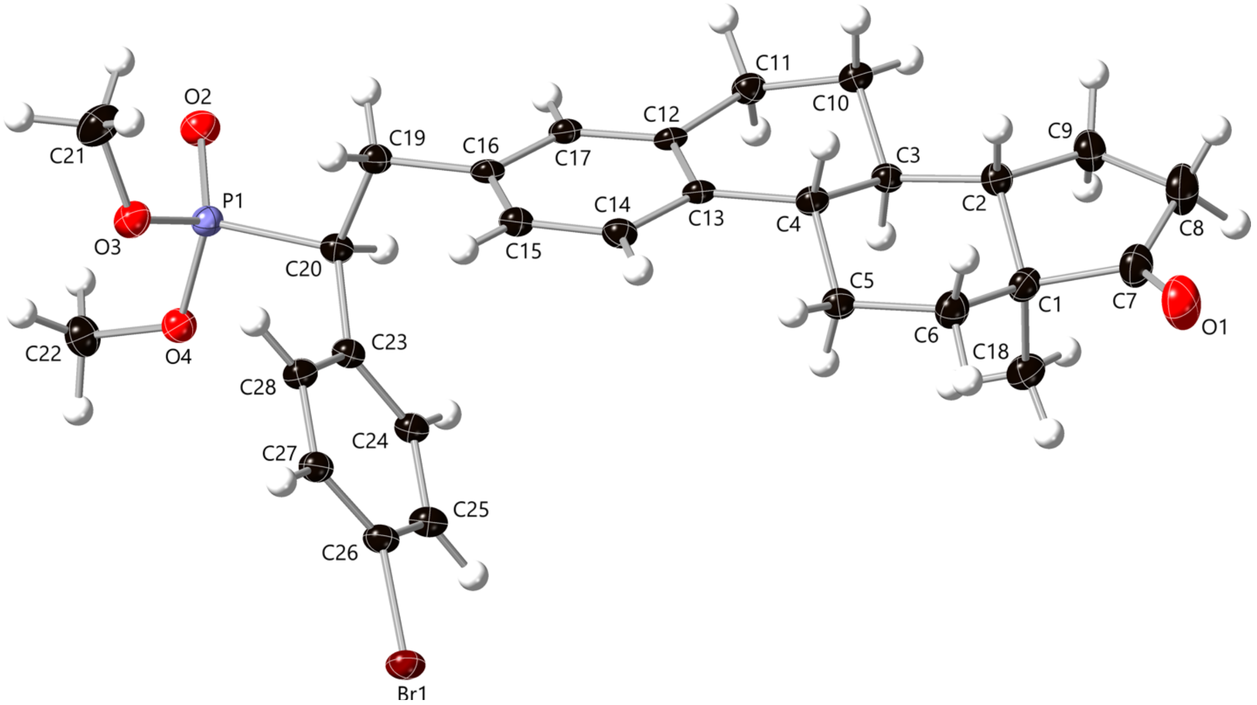

Figure S9: The molecular structure.

Data Plots: Diffraction Data

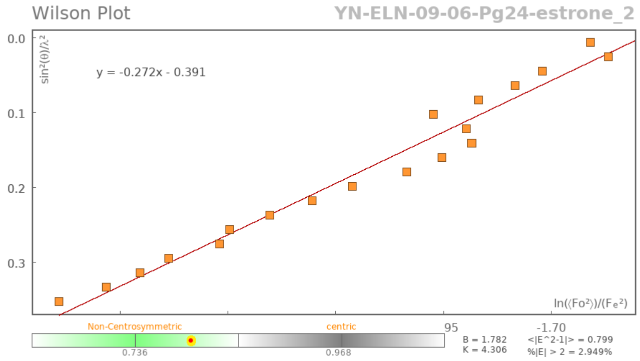

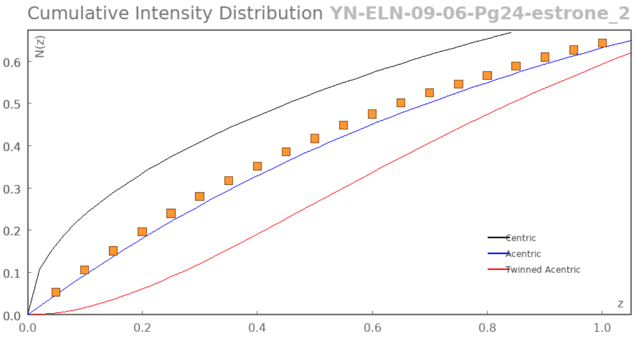

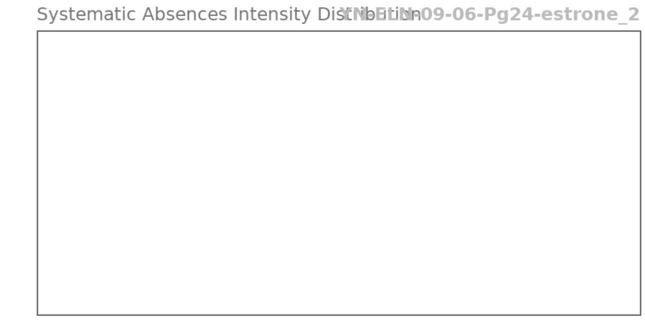

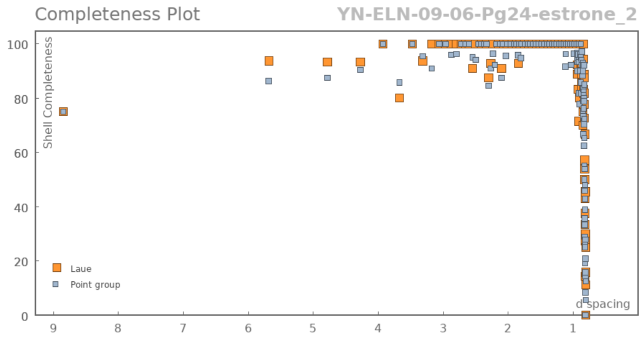

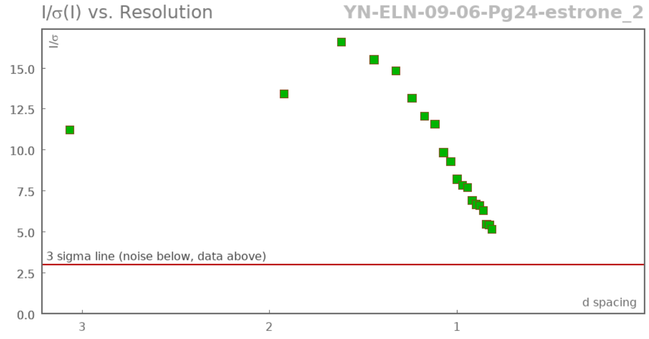

Data Plots: Refinement and Data

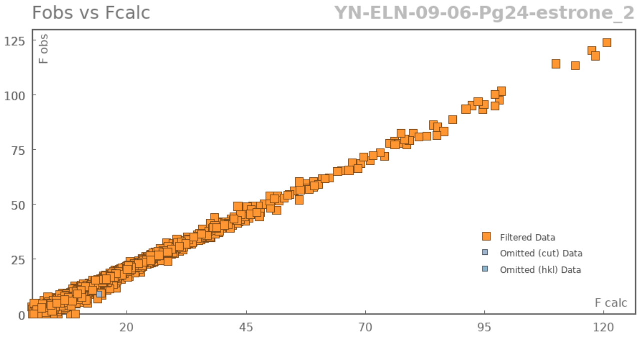

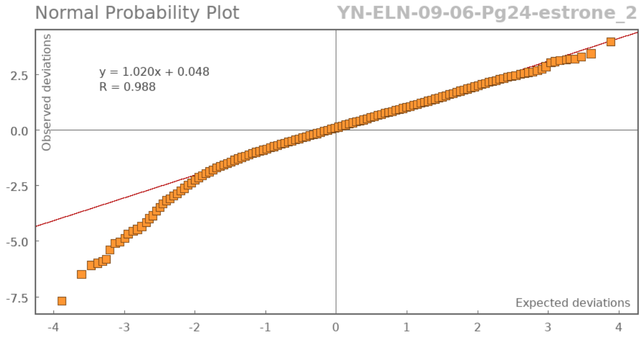

Table S11: Fractional Atomic Coordinates (×10⁴) and Equivalent Isotropic Displacement Parameters (Å²×10³) for compound **11p**. U_{eq} is defined as 1/3 of the trace of the orthogonalised U_{ij}.

Atom	x	y	z	U_{eq}
Br1_1	9031.7(4)	3329.8(10)	8011.5(2)	23.54(18)
C1_1	3383(4)	3882(11)	6729(2)	26.7(10)
C2_1	3197(4)	5194(10)	6209(2)	24.8(11)
C3_1	3960(3)	6121(9)	6092(2)	18.6(10)
C4_1	4537(3)	4193(9)	6062(2)	18.4(9)

Atom	x	y	z	U_{eq}
C5_1	4736(3)	2821(11)	6570(2)	23.0(11)
C6_1	3960(4)	1984(10)	6705(2)	25.5(12)
C7_1	2509(4)	3279(12)	6737(3)	33.2(12)
C8_1	1952(5)	5156(14)	6492(4)	47.9(18)
C9_1	2474(4)	6676(12)	6244(3)	38.7(15)
C10_1	3789(3)	7434(10)	5580(2)	20.6(11)
C11_1	4577(3)	8449(10)	5510(2)	19.6(10)
C12_1	5314(3)	6960(9)	5648.5(19)	15.6(9)
C13_1	5306(3)	4940(9)	5905(2)	16.4(9)
C14_1	6010(3)	3625(10)	6003(2)	19.6(10)
C15_1	6708(3)	4277(10)	5856(2)	19.8(10)
C16_1	6730(3)	6315(9)	5615(2)	17.1(9)
C17_1	6028(3)	7612(10)	5511.5(18)	16.5(9)
C18_1	3737(5)	5298(13)	7226(3)	37.3(16)
C19_1	7493(3)	7044(10)	5462(2)	19.6(10)
C20_1	8081(3)	8430(10)	5899.7(19)	18.6(10)
C21_1	9178(4)	12409(13)	5062(3)	34.7(15)
C22_1	10308(5)	9377(15)	6480(3)	48(2)
C23_1	8360(3)	7244(9)	6426(2)	18.9(10)
C24_1	8211(3)	8126(10)	6875(2)	20.7(10)
C25_1	8428(3)	7025(10)	7358(2)	21.8(11)
C26_1	8789(3)	4977(10)	7369(2)	21.1(11)
C27_1	8969(3)	4053(10)	6932(2)	22.4(11)
C28_1	8750(4)	5195(10)	6455(2)	21.8(11)
O1_1	2298(3)	1574(9)	6897(2)	41.9(12)
O2_1	9356(2)	7546(8)	5451.7(17)	29.8(9)
O3_1	8617(3)	11297(7)	5319.1(15)	26.3(9)
O4_1	9574(2)	10441(7)	6176.1(16)	26.8(9)
P1_1	8967.6(9)	9295(3)	5685.4(5)	19.2(3)
Br1_2	-3864.4(4)	-2139.6(10)	6977.8(2)	23.27(17)
C1_2	1848(4)	-348(10)	8367(2)	21.6(10)
C2_2	1979(3)	794(10)	8904(2)	19.8(10)
C3_2	1172(3)	1638(9)	8984(2)	16.6(9)
C4_2	596(3)	-344(9)	8965(2)	17.4(9)
C5_2	468(3)	-1627(11)	8446(2)	19.9(10)
C6_2	1282(3)	-2303(11)	8336(2)	23.7(11)
C7_2	2730(4)	-870(12)	8381(3)	29.0(12)
C8_2	3282(4)	915(12)	8699(3)	35.2(14)
C9_2	2714(3)	2319(11)	8944(2)	26.2(12)
C10_2	1259(3)	2910(10)	9497(2)	19.1(10)
C11_2	427(3)	3855(10)	9509(2)	19.8(10)
C12_2	-276(3)	2263(9)	9348.4(19)	15.8(9)
C13_2	-208(3)	285(9)	9091(2)	15.9(9)
C14_2	-889(3)	-1139(9)	8972(2)	18.9(10)
C15_2	-1609(3)	-610(10)	9105(2)	19.0(10)
C16_2	-1688(3)	1364(9)	9349(2)	16.5(9)
C17_2	-1020(3)	2774(10)	9462.4(18)	17.3(9)
C18_2	1534(4)	1216(12)	7897(2)	28.7(13)
C19_2	-2470(3)	1939(10)	9493(2)	19.2(10)
C20_2	-3075(3)	3254(10)	9046.3(19)	17.5(10)
C21_2	-4342(4)	1223(12)	9901(2)	31.6(14)
C22_2	-5270(4)	6254(11)	8755(3)	28.8(14)

Atom	x	y	z	U_{eq}
C23_2	-3317(3)	2003(9)	8529(2)	17.8(10)
C24_2	-3126(3)	2815(11)	8084.3(19)	19.3(10)
C25_2	-3297(4)	1621(10)	7616(2)	22.0(11)
C26_2	-3654(3)	-414(10)	7605(2)	20.1(10)
C27_2	-3865(3)	-1276(10)	8038(2)	20.6(10)
C28_2	-3691(3)	-65(9)	8501(2)	19.1(10)
O1_2	2965(3)	-2446(9)	8186.5(19)	41.6(12)
O2_2	-3711(2)	5707(7)	9722.1(15)	25.3(9)
O3_2	-4434(3)	2130(7)	9380.0(15)	23.7(9)
O4_2	-4513(2)	5145(7)	8751.0(15)	23.5(9)
P1_2	-3940.2(9)	4198(2)	9274.1(5)	17.6(3)

Table S12: Anisotropic Displacement Parameters (×10^4) for compound **11p.** The anisotropic displacement factor exponent takes the form: $-2\pi^2[h^2a^{*2} \times U_{11} + ... +2hka^* \times b^* \times U_{12}]$

Atom	U_{11}	U_{22}	U_{33}	U_{23}	U_{13}	U_{12}
Br1_1	25.6(3)	23.6(4)	18.9(3)	4.8(2)	1.5(2)	0.2(2)
C1_1	34(2)	25(2)	27(2)	-0.4(16)	17.0(16)	-4.1(16)
C2_1	25(2)	27(3)	26(2)	-0.2(19)	13.6(18)	-2.6(18)
C3_1	20.5(19)	19(2)	18(2)	-2.9(16)	6.8(16)	-1.7(15)
C4_1	22.6(19)	17(2)	16(2)	-1.6(16)	7.3(16)	-1.6(15)
C5_1	28(2)	22(3)	20(2)	3.3(19)	8.5(17)	-2(2)
C6_1	32(2)	25(2)	24(3)	1(2)	13(2)	-4.1(18)
C7_1	37(2)	32(2)	39(3)	-7(2)	25(2)	-6.7(18)
C8_1	40(3)	44(3)	70(5)	10(3)	34(3)	0(2)
C9_1	32(3)	34(3)	58(4)	4(3)	27(3)	3(2)
C10_1	22.9(19)	21(3)	18(2)	-1.8(18)	6.6(17)	-1.0(18)
C11_1	21.7(17)	19(2)	18(2)	2.6(19)	5.4(16)	1.7(16)
C12_1	20.1(17)	17.0(19)	9(2)	-0.7(15)	2.8(15)	0.0(14)
C13_1	19.8(17)	18.3(19)	11(2)	0.9(15)	2.9(16)	0.0(14)
C14_1	21.7(17)	18(2)	18(2)	3.1(19)	4.3(15)	1.4(15)
C15_1	21.4(19)	20(2)	17(2)	0.3(17)	3.7(18)	-0.4(16)
C16_1	19.7(17)	18.1(19)	12(2)	-2.7(15)	2.5(16)	-1.3(14)
C17_1	20.3(16)	18(2)	10(2)	-0.9(18)	3.2(14)	-0.9(14)
C18_1	53(4)	35(3)	29(3)	-6(2)	21(3)	-11(3)
C19_1	21.1(18)	21(3)	17(2)	-2.4(18)	5.7(16)	-0.6(17)
C20_1	21(2)	17(2)	17.5(16)	-0.7(15)	3.8(15)	1.2(18)
C21_1	38(3)	37(4)	35(3)	8(3)	20(3)	-9(3)
C22_1	30(3)	55(5)	48(4)	1(3)	-10(3)	4(3)
C23_1	19(2)	18.3(19)	17.8(16)	-0.5(14)	2.1(15)	-0.6(17)
C24_1	24(2)	19(2)	18.4(16)	-0.3(15)	3.2(15)	2(2)
C25_1	23(3)	22(2)	18.8(19)	1.4(16)	2.6(17)	2.4(18)
C26_1	19(2)	22(2)	19(2)	2.0(16)	-1.6(19)	2.0(18)
C27_1	25(3)	19(2)	21.4(18)	1.2(15)	2.8(16)	-2.1(19)
C28_1	25(3)	20(2)	20(2)	0.5(16)	4.8(18)	2.1(18)
O1_1	45(3)	36(2)	54(3)	-1(2)	31(2)	-8.8(19)
O2_1	30(2)	22(2)	41(2)	-8(2)	16.4(18)	-2.2(19)
O3_1	27(2)	29(2)	26(2)	9.1(17)	11.7(17)	-2.7(17)

Atom	U_{11}	U_{22}	U_{33}	U_{23}	U_{13}	U_{12}
O4_1	23.0(19)	29(2)	26(2)	-3.8(17)	0.6(16)	-4.1(17)
P1_1	19.7(7)	19.1(7)	19.4(7)	0.1(6)	6.2(6)	-1.7(6)
Br1_2	26.4(3)	25.2(4)	16.6(3)	-5.4(2)	2.9(2)	-0.5(3)
C1_2	22.4(19)	24(2)	20.2(19)	-0.7(15)	9.5(15)	1.1(15)
C2_2	17.9(18)	22(2)	20(2)	0.1(17)	5.2(15)	1.8(16)
C3_2	17.3(18)	16(2)	15.6(19)	0.6(16)	3.2(15)	0.9(15)
C4_2	20.0(18)	16(2)	15.8(19)	-0.8(16)	4.5(15)	-0.2(15)
C5_2	20(2)	22(3)	18.4(19)	-4.8(19)	4.9(15)	-1.4(19)
C6_2	23.6(19)	24(2)	26(3)	-3(2)	10.0(18)	0.9(17)
C7_2	24(2)	33(2)	33(3)	0(2)	12.7(18)	3.6(16)
C8_2	25(2)	37(3)	48(3)	-6(2)	18(2)	-1(2)
C9_2	20(2)	29(3)	32(3)	-1(2)	9(2)	-1.5(18)
C10_2	22.1(19)	19(3)	16.1(19)	-0.5(18)	5.4(16)	-0.5(19)
C11_2	22.2(17)	19(2)	18(2)	-3.6(19)	5.8(16)	-1.0(15)
C12_2	20.7(16)	16.8(18)	9(2)	-0.3(15)	2.9(15)	1.0(14)
C13_2	19.7(17)	16.8(18)	10(2)	-0.7(15)	2.0(15)	0.9(14)
C14_2	20.5(16)	18(2)	17(2)	-3.5(19)	2.7(15)	-0.2(14)
C15_2	20(2)	20(2)	16(2)	-0.6(17)	2.5(18)	0.5(16)
C16_2	19.3(17)	18.7(19)	10(2)	2.6(15)	1.1(15)	2.0(13)
C17_2	20.2(15)	20(2)	11(2)	-0.2(18)	2.8(14)	1.3(14)
C18_2	29(3)	36(3)	22(3)	5(2)	9(2)	2(2)
C19_2	20.1(19)	21(3)	16(2)	0.3(18)	3.3(15)	1.4(17)
C20_2	19(2)	17(2)	16.8(16)	-0.2(15)	3.4(14)	-0.5(18)
C21_2	42(4)	29(3)	28(2)	6(2)	16(2)	4(3)
C22_2	21(2)	34(3)	31(3)	2(3)	6(2)	9(2)
C23_2	18(2)	17.3(18)	16.6(16)	-0.3(14)	2.2(15)	0.8(16)
C24_2	20(2)	19(2)	16.9(16)	0.7(14)	2.5(15)	0(2)
C25_2	24(3)	23(2)	17.2(19)	-0.6(16)	1.9(18)	-0.2(18)
C26_2	21(2)	22(2)	16(2)	-2.9(16)	1.5(18)	0.9(18)
C27_2	20(2)	23(2)	17.6(17)	-1.9(14)	2.9(15)	2(2)
C28_2	22(3)	18.0(19)	16.7(19)	0.2(15)	5.0(18)	-0.7(17)
O1_2	29(2)	43(2)	56(3)	-13(2)	16(2)	6(2)
O2_2	28(2)	24(2)	24(2)	-7.9(17)	6.6(17)	2.3(18)
O3_2	27(2)	21(2)	23.8(17)	0.4(15)	9.4(15)	-5.1(17)
O4_2	21.3(18)	27(2)	21(2)	2.4(16)	5.3(15)	7.2(16)
P1_2	18.8(7)	17.1(7)	16.9(7)	-0.7(5)	5.1(5)	1.8(5)

Table S13: Bond Lengths in Å for compound **11p**.

Atom	Atom	Length/Å
Br1_1	C26_1	1.913(6)
C1_1	C2_1	1.542(9)
C1_1	C6_1	1.526(9)
C1_1	C7_1	1.524(8)
C1_1	C18_1	1.546(9)
C2_1	C3_1	1.510(8)

Atom	Atom	Length/Å
C2_1	C9_1	1.540(9)
C3_1	C4_1	1.543(8)
C3_1	C10_1	1.526(8)
C4_1	C5_1	1.537(8)
C4_1	C13_1	1.529(8)
C5_1	C6_1	1.531(8)
C7_1	C8_1	1.513(11)
C7_1	O1_1	1.212(9)
C8_1	C9_1	1.538(10)
C10_1	C11_1	1.521(8)
C11_1	C12_1	1.505(7)
C12_1	C13_1	1.409(8)
C12_1	C17_1	1.403(7)
C13_1	C14_1	1.400(8)
C14_1	C15_1	1.391(8)
C15_1	C16_1	1.402(8)
C16_1	C17_1	1.388(8)
C16_1	C19_1	1.514(8)
C19_1	C20_1	1.554(8)
C20_1	C23_1	1.519(7)
C20_1	P1_1	1.810(5)
C21_1	O3_1	1.467(7)
C22_1	O4_1	1.438(8)
C23_1	C24_1	1.382(8)
C23_1	C28_1	1.406(8)
C24_1	C25_1	1.394(8)
C25_1	C26_1	1.389(8)
C26_1	C27_1	1.384(8)
C27_1	C28_1	1.395(8)
O2_1	P1_1	1.470(4)
O3_1	P1_1	1.571(4)
O4_1	P1_1	1.579(4)
Br1_2	C26_2	1.909(5)
C1_2	C2_2	1.536(8)
C1_2	C6_2	1.517(8)
C1_2	C7_2	1.512(8)
C1_2	C18_2	1.540(9)
C2_2	C3_2	1.520(8)
C2_2	C9_2	1.532(8)
C3_2	C4_2	1.545(8)
C3_2	C10_2	1.529(7)
C4_2	C5_2	1.537(7)
C4_2	C13_2	1.527(8)
C5_2	C6_2	1.532(7)
C7_2	C8_2	1.530(10)
C7_2	O1_2	1.205(9)
C8_2	C9_2	1.547(9)
C10_2	C11_2	1.525(7)
C11_2	C12_2	1.505(8)
C12_2	C13_2	1.405(7)
C12_2	C17_2	1.398(7)
C13_2	C14_2	1.407(8)

Atom	Atom	Length/Å
C14_2	C15_2	1.388(8)
C15_2	C16_2	1.389(8)
C16_2	C17_2	1.386(8)
C16_2	C19_2	1.507(8)
C19_2	C20_2	1.558(7)
C20_2	C23_2	1.517(7)
C20_2	P1_2	1.812(5)
C21_2	O3_2	1.447(7)
C22_2	O4_2	1.447(7)
C23_2	C24_2	1.384(8)
C23_2	C28_2	1.406(8)
C24_2	C25_2	1.393(8)
C25_2	C26_2	1.379(8)
C26_2	C27_2	1.383(8)
C27_2	C28_2	1.386(8)
O2_2	P1_2	1.464(4)
O3_2	P1_2	1.579(4)
O4_2	P1_2	1.565(4)

Table S1: Bond Angles in ° for compound **11p**.

Atom	Atom	Atom	Angle/°
C2_1	C1_1	C18_1	113.4(6)
C6_1	C1_1	C2_1	109.8(5)
C6_1	C1_1	C18_1	110.7(6)
C7_1	C1_1	C2_1	99.7(5)
C7_1	C1_1	C6_1	116.5(5)
C7_1	C1_1	C18_1	106.3(5)
C3_1	C2_1	C1_1	113.1(5)
C3_1	C2_1	C9_1	121.5(5)
C9_1	C2_1	C1_1	103.3(5)
C2_1	C3_1	C4_1	107.9(5)
C2_1	C3_1	C10_1	113.9(5)
C10_1	C3_1	C4_1	109.2(4)
C5_1	C4_1	C3_1	111.4(4)
C13_1	C4_1	C3_1	112.2(5)
C13_1	C4_1	C5_1	113.1(4)
C6_1	C5_1	C4_1	112.4(5)
C1_1	C6_1	C5_1	110.4(5)
C8_1	C7_1	C1_1	107.7(6)
O1_1	C7_1	C1_1	125.9(6)
O1_1	C7_1	C8_1	126.4(6)
C7_1	C8_1	C9_1	106.1(6)
C8_1	C9_1	C2_1	102.8(6)
C11_1	C10_1	C3_1	110.8(4)
C12_1	C11_1	C10_1	114.6(5)
C13_1	C12_1	C11_1	122.2(5)
C17_1	C12_1	C11_1	118.5(5)
C17_1	C12_1	C13_1	119.4(5)
C12_1	C13_1	C4_1	120.5(5)
C14_1	C13_1	C4_1	121.2(5)

Atom	Atom	Atom	Angle/°
C14_1	C13_1	C12_1	118.3(5)
C15_1	C14_1	C13_1	121.6(5)
C14_1	C15_1	C16_1	120.4(5)
C15_1	C16_1	C19_1	120.5(5)
C17_1	C16_1	C15_1	118.2(5)
C17_1	C16_1	C19_1	121.3(5)
C16_1	C17_1	C12_1	122.1(5)
C16_1	C19_1	C20_1	112.6(4)
C19_1	C20_1	P1_1	110.2(4)
C23_1	C20_1	C19_1	113.2(5)
C23_1	C20_1	P1_1	109.9(4)
C24_1	C23_1	C20_1	120.6(5)
C24_1	C23_1	C28_1	119.3(5)
C28_1	C23_1	C20_1	120.2(5)
C23_1	C24_1	C25_1	121.7(6)
C26_1	C25_1	C24_1	117.5(5)
C25_1	C26_1	Br1_1	118.9(4)
C27_1	C26_1	Br1_1	118.3(4)
C27_1	C26_1	C25_1	122.8(5)
C26_1	C27_1	C28_1	118.5(6)
C27_1	C28_1	C23_1	120.2(5)
C21_1	O3_1	P1_1	117.3(4)
C22_1	O4_1	P1_1	121.8(5)
O2_1	P1_1	C20_1	114.4(3)
O2_1	P1_1	O3_1	116.5(3)
O2_1	P1_1	O4_1	113.6(2)
O3_1	P1_1	C20_1	102.2(3)
O3_1	P1_1	O4_1	102.1(2)
O4_1	P1_1	C20_1	106.5(2)
C2_2	C1_2	C18_2	112.9(5)
C6_2	C1_2	C2_2	110.0(5)
C6_2	C1_2	C18_2	111.7(5)
C7_2	C1_2	C2_2	100.1(5)
C7_2	C1_2	C6_2	115.8(5)
C7_2	C1_2	C18_2	105.9(5)
C3_2	C2_2	C1_2	111.4(4)
C3_2	C2_2	C9_2	121.5(5)
C9_2	C2_2	C1_2	104.7(5)
C2_2	C3_2	C4_2	107.9(4)
C2_2	C3_2	C10_2	114.5(4)
C10_2	C3_2	C4_2	109.8(4)
C5_2	C4_2	C3_2	111.4(4)
C13_2	C4_2	C3_2	112.5(4)
C13_2	C4_2	C5_2	113.4(4)
C6_2	C5_2	C4_2	112.6(4)
C1_2	C6_2	C5_2	111.0(5)
C1_2	C7_2	C8_2	108.3(5)
O1_2	C7_2	C1_2	126.2(6)
O1_2	C7_2	C8_2	125.5(6)
C7_2	C8_2	C9_2	105.6(5)
C2_2	C9_2	C8_2	102.3(5)
C11_2	C10_2	C3_2	109.7(4)

Atom	Atom	Atom	Angle/°
C12_2	C11_2	C10_2	114.2(5)
C13_2	C12_2	C11_2	122.1(5)
C17_2	C12_2	C11_2	118.7(5)
C17_2	C12_2	C13_2	119.2(5)
C12_2	C13_2	C4_2	120.8(5)
C12_2	C13_2	C14_2	118.2(5)
C14_2	C13_2	C4_2	121.0(5)
C15_2	C14_2	C13_2	121.1(5)
C14_2	C15_2	C16_2	121.1(5)
C15_2	C16_2	C19_2	121.1(5)
C17_2	C16_2	C15_2	117.8(5)
C17_2	C16_2	C19_2	121.1(5)
C16_2	C17_2	C12_2	122.6(5)
C16_2	C19_2	C20_2	111.8(4)
C19_2	C20_2	P1_2	109.8(3)
C23_2	C20_2	C19_2	112.4(5)
C23_2	C20_2	P1_2	114.1(3)
C24_2	C23_2	C20_2	120.6(5)
C24_2	C23_2	C28_2	118.6(5)
C28_2	C23_2	C20_2	120.7(5)
C23_2	C24_2	C25_2	121.2(6)
C26_2	C25_2	C24_2	118.5(5)
C25_2	C26_2	Br1_2	119.7(4)
C25_2	C26_2	C27_2	122.2(5)
C27_2	C26_2	Br1_2	118.1(4)
C26_2	C27_2	C28_2	118.5(6)
C27_2	C28_2	C23_2	120.9(5)
C21_2	O3_2	P1_2	123.0(4)
C22_2	O4_2	P1_2	120.4(4)
O2_2	P1_2	C20_2	113.7(3)
O2_2	P1_2	O3_2	113.7(2)
O2_2	P1_2	O4_2	116.2(2)
O3_2	P1_2	C20_2	108.2(3)
O4_2	P1_2	C20_2	101.0(2)
O4_2	P1_2	O3_2	102.7(2)

Table 215: Torsion Angles in ° for compound **11p**.

Atom	Atom	Atom	Atom	Angle/°
Br1_1	C26_1	C27_1	C28_1	176.3(4)
C1_1	C2_1	C3_1	C4_1	58.8(6)
C1_1	C2_1	C3_1	C10_1	-179.8(5)
C1_1	C2_1	C9_1	C8_1	-39.2(7)
C1_1	C7_1	C8_1	C9_1	10.5(8)
C2_1	C1_1	C6_1	C5_1	54.5(6)
C2_1	C1_1	C7_1	C8_1	-34.0(7)
C2_1	C1_1	C7_1	O1_1	144.1(7)
C2_1	C3_1	C4_1	C5_1	-56.1(6)
C2_1	C3_1	C4_1	C13_1	176.0(4)
C2_1	C3_1	C10_1	C11_1	175.9(5)
C3_1	C2_1	C9_1	C8_1	-167.3(6)

Atom	Atom	Atom	Atom	Angle/˚
C3_1	C4_1	C5_1	C6_1	55.7(6)
C3_1	C4_1	C13_1	C12_1	-20.4(7)
C3_1	C4_1	C13_1	C14_1	161.0(5)
C3_1	C10_1	C11_1	C12_1	42.4(6)
C4_1	C3_1	C10_1	C11_1	-63.5(6)
C4_1	C5_1	C6_1	C1_1	-54.4(6)
C4_1	C13_1	C14_1	C15_1	179.1(5)
C5_1	C4_1	C13_1	C12_1	-147.4(5)
C5_1	C4_1	C13_1	C14_1	34.0(7)
C6_1	C1_1	C2_1	C3_1	-59.2(7)
C6_1	C1_1	C2_1	C9_1	167.7(5)
C6_1	C1_1	C7_1	C8_1	-152.0(6)
C6_1	C1_1	C7_1	O1_1	26.0(9)
C7_1	C1_1	C2_1	C3_1	178.0(5)
C7_1	C1_1	C2_1	C9_1	44.8(6)
C7_1	C1_1	C6_1	C5_1	166.8(5)
C7_1	C8_1	C9_1	C2_1	17.7(8)
C9_1	C2_1	C3_1	C4_1	-177.5(5)
C9_1	C2_1	C3_1	C10_1	-56.1(7)
C10_1	C3_1	C4_1	C5_1	179.6(5)
C10_1	C3_1	C4_1	C13_1	51.7(6)
C10_1	C11_1	C12_1	C13_1	-10.3(7)
C10_1	C11_1	C12_1	C17_1	169.2(4)
C11_1	C12_1	C13_1	C4_1	-0.8(7)
C11_1	C12_1	C13_1	C14_1	177.8(5)
C11_1	C12_1	C17_1	C16_1	-178.7(5)
C12_1	C13_1	C14_1	C15_1	0.5(8)
C13_1	C4_1	C5_1	C6_1	-176.9(5)
C13_1	C12_1	C17_1	C16_1	0.8(7)
C13_1	C14_1	C15_1	C16_1	1.5(8)
C14_1	C15_1	C16_1	C17_1	-2.3(8)
C14_1	C15_1	C16_1	C19_1	179.1(5)
C15_1	C16_1	C17_1	C12_1	1.2(8)
C15_1	C16_1	C19_1	C20_1	-93.2(6)
C16_1	C19_1	C20_1	C23_1	57.7(6)
C16_1	C19_1	C20_1	P1_1	-178.7(4)
C17_1	C12_1	C13_1	C4_1	179.7(5)
C17_1	C12_1	C13_1	C14_1	-1.6(7)
C17_1	C16_1	C19_1	C20_1	88.3(6)
C18_1	C1_1	C2_1	C3_1	65.3(7)
C18_1	C1_1	C2_1	C9_1	-67.8(7)
C18_1	C1_1	C6_1	C5_1	-71.5(6)
C18_1	C1_1	C7_1	C8_1	84.1(7)
C18_1	C1_1	C7_1	O1_1	-97.9(8)
C19_1	C16_1	C17_1	C12_1	179.8(5)
C19_1	C20_1	C23_1	C24_1	-122.0(6)
C19_1	C20_1	C23_1	C28_1	55.9(7)
C19_1	C20_1	P1_1	O2_1	-47.1(5)
C19_1	C20_1	P1_1	O3_1	79.8(4)
C19_1	C20_1	P1_1	O4_1	-173.5(4)
C20_1	C23_1	C24_1	C25_1	177.1(5)
C20_1	C23_1	C28_1	C27_1	-176.8(5)

Atom	Atom	Atom	Atom	Angle/°
C21_1	O3_1	P1_1	C20_1	-179.0(4)
C21_1	O3_1	P1_1	O2_1	-53.4(5)
C21_1	O3_1	P1_1	O4_1	71.0(5)
C22_1	O4_1	P1_1	C20_1	104.6(6)
C22_1	O4_1	P1_1	O2_1	-22.3(6)
C22_1	O4_1	P1_1	O3_1	-148.6(6)
C23_1	C20_1	P1_1	O2_1	78.3(4)
C23_1	C20_1	P1_1	O3_1	-154.8(4)
C23_1	C20_1	P1_1	O4_1	-48.0(5)
C23_1	C24_1	C25_1	C26_1	-1.1(8)
C24_1	C23_1	C28_1	C27_1	1.1(9)
C24_1	C25_1	C26_1	Br1_1	-176.0(4)
C24_1	C25_1	C26_1	C27_1	2.8(9)
C25_1	C26_1	C27_1	C28_1	-2.4(9)
C26_1	C27_1	C28_1	C23_1	0.5(9)
C28_1	C23_1	C24_1	C25_1	-0.8(8)
O1_1	C7_1	C8_1	C9_1	-167.5(7)
P1_1	C20_1	C23_1	C24_1	114.3(5)
P1_1	C20_1	C23_1	C28_1	-67.9(6)
Br1_2	C26_2	C27_2	C28_2	178.0(4)
C1_2	C2_2	C3_2	C4_2	60.8(6)
C1_2	C2_2	C3_2	C10_2	-176.7(5)
C1_2	C2_2	C9_2	C8_2	-39.0(6)
C1_2	C7_2	C8_2	C9_2	8.2(7)
C2_2	C1_2	C6_2	C5_2	55.1(6)
C2_2	C1_2	C7_2	C8_2	-31.4(6)
C2_2	C1_2	C7_2	O1_2	146.9(7)
C2_2	C3_2	C4_2	C5_2	-56.5(5)
C2_2	C3_2	C4_2	C13_2	174.8(4)
C2_2	C3_2	C10_2	C11_2	174.7(5)
C3_2	C2_2	C9_2	C8_2	-166.1(5)
C3_2	C4_2	C5_2	C6_2	53.6(7)
C3_2	C4_2	C13_2	C12_2	-18.2(7)
C3_2	C4_2	C13_2	C14_2	164.5(5)
C3_2	C10_2	C11_2	C12_2	45.9(6)
C4_2	C3_2	C10_2	C11_2	-63.8(6)
C4_2	C5_2	C6_2	C1_2	-52.5(6)
C4_2	C13_2	C14_2	C15_2	177.3(5)
C5_2	C4_2	C13_2	C12_2	-145.8(5)
C5_2	C4_2	C13_2	C14_2	36.9(7)
C6_2	C1_2	C2_2	C3_2	-61.1(6)
C6_2	C1_2	C2_2	C9_2	165.8(5)
C6_2	C1_2	C7_2	C8_2	-149.5(5)
C6_2	C1_2	C7_2	O1_2	28.8(9)
C7_2	C1_2	C2_2	C3_2	176.6(5)
C7_2	C1_2	C2_2	C9_2	43.5(6)
C7_2	C1_2	C6_2	C5_2	167.6(5)
C7_2	C8_2	C9_2	C2_2	18.6(7)
C9_2	C2_2	C3_2	C4_2	-175.1(5)
C9_2	C2_2	C3_2	C10_2	-52.6(7)
C10_2	C3_2	C4_2	C5_2	178.1(4)
C10_2	C3_2	C4_2	C13_2	49.4(6)

Atom	Atom	Atom	Atom	Angle/˚
C10_2	C11_2	C12_2	C13_2	-14.6(7)
C10_2	C11_2	C12_2	C17_2	165.1(4)
C11_2	C12_2	C13_2	C4_2	0.5(7)
C11_2	C12_2	C13_2	C14_2	177.8(5)
C11_2	C12_2	C17_2	C16_2	-177.2(5)
C12_2	C13_2	C14_2	C15_2	0.0(8)
C13_2	C4_2	C5_2	C6_2	-178.2(5)
C13_2	C12_2	C17_2	C16_2	2.5(7)
C13_2	C14_2	C15_2	C16_2	1.5(8)
C14_2	C15_2	C16_2	C17_2	-0.9(8)
C14_2	C15_2	C16_2	C19_2	179.9(5)
C15_2	C16_2	C17_2	C12_2	-1.1(7)
C15_2	C16_2	C19_2	C20_2	-92.3(6)
C16_2	C19_2	C20_2	C23_2	59.1(6)
C16_2	C19_2	C20_2	P1_2	-172.7(4)
C17_2	C12_2	C13_2	C4_2	-179.3(5)
C17_2	C12_2	C13_2	C14_2	-1.9(7)
C17_2	C16_2	C19_2	C20_2	88.6(6)
C18_2	C1_2	C2_2	C3_2	64.4(6)
C18_2	C1_2	C2_2	C9_2	-68.7(6)
C18_2	C1_2	C6_2	C5_2	-71.1(6)
C18_2	C1_2	C7_2	C8_2	86.2(6)
C18_2	C1_2	C7_2	O1_2	-95.6(8)
C19_2	C16_2	C17_2	C12_2	178.1(5)
C19_2	C20_2	C23_2	C24_2	-118.2(6)
C19_2	C20_2	C23_2	C28_2	57.8(6)
C19_2	C20_2	P1_2	O2_2	62.6(5)
C19_2	C20_2	P1_2	O3_2	-64.8(4)
C19_2	C20_2	P1_2	O4_2	-172.2(4)
C20_2	C23_2	C24_2	C25_2	175.9(5)
C20_2	C23_2	C28_2	C27_2	-175.9(5)
C21_2	O3_2	P1_2	C20_2	99.2(5)
C21_2	O3_2	P1_2	O2_2	-28.1(5)
C21_2	O3_2	P1_2	O4_2	-154.5(5)
C22_2	O4_2	P1_2	C20_2	-175.2(5)
C22_2	O4_2	P1_2	O2_2	-51.7(5)
C22_2	O4_2	P1_2	O3_2	73.1(5)
C23_2	C20_2	P1_2	O2_2	-170.3(4)
C23_2	C20_2	P1_2	O3_2	62.4(4)
C23_2	C20_2	P1_2	O4_2	-45.0(5)
C23_2	C24_2	C25_2	C26_2	-0.8(8)
C24_2	C23_2	C28_2	C27_2	0.2(8)
C24_2	C25_2	C26_2	Br1_2	-177.9(4)
C24_2	C25_2	C26_2	C27_2	1.7(9)
C25_2	C26_2	C27_2	C28_2	-1.7(8)
C26_2	C27_2	C28_2	C23_2	0.7(8)
C28_2	C23_2	C24_2	C25_2	-0.2(8)
O1_2	C7_2	C8_2	C9_2	-170.0(7)
P1_2	C20_2	C23_2	C24_2	116.0(5)
P1_2	C20_2	C23_2	C28_2	-68.0(6)

Table S3: Hydrogen Fractional Atomic Coordinates (×10^4) and Equivalent Isotropic Displacement Parameters (Å^2×10^3) for compound **11p**. U_{eq} is defined as 1/3 of the trace of the orthogonalised U_{ij}.

Atom	x	y	z	U_{eq}
H2_1	2964(4)	4115(16)	5920(4)	30
H3_1	4246(5)	7099(14)	6393(4)	22
H4_1	4223(5)	3212(14)	5770(4)	22
H5A_1	5086(5)	1539(17)	6528(2)	28
H5B_1	5063(5)	3736(15)	6871(4)	28
H6A_1	3669(5)	907(16)	6429(4)	31
H6B_1	4118(4)	1211(13)	7055(4)	31
H8A_1	1753(5)	5956(16)	6768(5)	57
H8B_1	1461(7)	4601(15)	6214(5)	57
H9A_1	2673(5)	7983(19)	6475(4)	46
H9B_1	2154(5)	7189(14)	5885(5)	46
H10A_1	3543(4)	6446(14)	5273(4)	25
H10B_1	3379(6)	8621(16)	5587(2)	25
H11A_1	4479(3)	8922(11)	5132(4)	24
H11B_1	4705(3)	9795(17)	5737(3)	24
H14_1	6012(3)	2230(30)	6176(5)	24
H15_1	7181(12)	3320(20)	5920(3)	24
H17_1	6032(3)	9010(30)	5340(4)	20
H18A_1	4260(50)	5980(70)	7202(4)	56
H18B_1	3870(50)	4390(160)	7530(40)	56
H18C_1	3300(60)	6500(160)	7200(30)	56
H19A_1	7326(8)	7920(40)	5132(15)	24
H19B_1	7793(14)	5730(60)	5388(4)	24
H20_1	7820(20)	9580(100)	5948(4)	22
H21A_1	9326(5)	11360(20)	4790(6)	52
H21B_1	9690(50)	12880(150)	5300(30)	52
H21C_1	8890(50)	13940(160)	4830(30)	52
H22A_1	10480(20)	8300(120)	6260(20)	72
H22B_1	10190(60)	8680(190)	6820(40)	72
H22C_1	10660(40)	10790(80)	6570(40)	72
H24_1	7943(6)	9580(30)	6855(2)	25
H25_1	8328(4)	7679(16)	7679(7)	26
H27_1	9247(7)	2620(30)	6958(2)	27
H28_1	8869(4)	4561(16)	6137(7)	26
H2_2	2175(4)	-371(16)	9179(4)	24
H3_2	912(4)	2631(14)	8680(4)	20
H4_2	891(5)	-1360(14)	9255(4)	21
H5A_2	135(5)	-2974(18)	8463(2)	24
H5B_2	147(5)	-698(14)	8146(4)	24
H6A_2	1562(4)	-3433(16)	8602(4)	28
H6B_2	1168(3)	-2976(13)	7975(4)	28
H8A_2	3530(5)	1831(16)	8463(4)	42
H8B_2	3739(6)	249(14)	8983(4)	42
H9A_2	2991(4)	2701(12)	9322(5)	31
H9B_2	2543(4)	3702(18)	8737(3)	31
H10A_2	1467(4)	1910(14)	9807(4)	23
H10B_2	1669(5)	4128(16)	9522(2)	23

Atom	x	y	z	U_{eq}
H11A_2	464(3)	4387(11)	9876(4)	24
H11B_2	305(4)	5155(17)	9266(3)	24
H14_2	-854(3)	-2510(30)	8795(5)	23
H15_2	-2063(11)	-1630(30)	9027(3)	23
H17_2	-1069(3)	4160(30)	9626(4)	21
H18A_2	940(50)	1720(40)	7882(3)	43
H18B_2	1510(50)	400(140)	7580(30)	43
H18C_2	1860(30)	2680(60)	7940(30)	43
H19A_2	-2331(4)	2833(14)	9826(4)	23
H19B_2	-2746(4)	559(17)	9564(2)	23
H20_2	-2771(4)	4596(17)	8982(2)	21
H21A_2	-4079(7)	-320(30)	9921(3)	47
H21B_2	-4890(20)	1160(150)	10010(20)	47
H21C_2	-4000(40)	2280(90)	10190(20)	47
H22A_2	-5563(7)	6786(16)	8375(8)	43
H22B_2	-5130(40)	7570(70)	9010(20)	43
H22C_2	-5610(30)	5250(100)	8940(20)	43
H24_2	-2864(6)	4270(30)	8099(2)	23
H25_2	-3166(4)	2220(15)	7298(7)	26
H27_2	-4135(6)	-2720(30)	8018(2)	25
H28_2	-3832(4)	-664(15)	8815(7)	23

X-Ray Crystallographic Data for Compound 15 (CCDC 2254293).

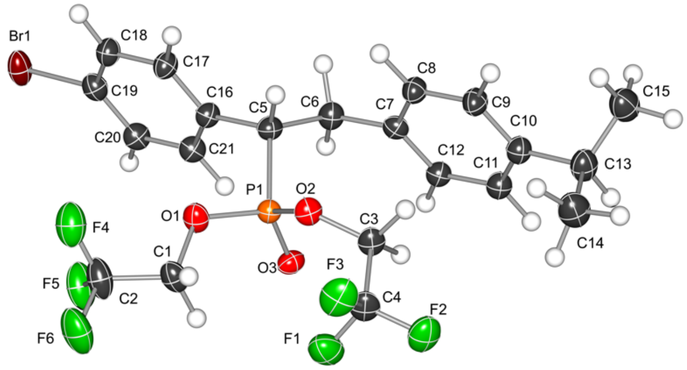

Experimental. Single colourless needle-shaped crystals of compound **15.** The crystal was chosen from the sample as supplied. A suitable crystal with dimensions 0.49 × 0.09 × 0.06 mm^3 was selected and mounted on a loop with paratone on a XtaLAB Synergy, Dualflex, HyPix diffractometer. The crystal was kept at a steady T = 99.96(12) K during data collection. The structure was solved with the ShelXT 2018/2 (Sheldrick, 2018) solution program using dual methods and by using Olex2 1.5-alpha (Dolomanov et al., 2009) as the graphical interface. The model was refined with olex2.refine 1.5-alpha (Bourhis et al., 2015) using full matrix least squares minimisation on $\boldsymbol{F}^2$.

Crystal Data. $C_{21}H_{22}BrF_6O_3P$, M_r = 547.275, monoclinic, $P2_1$ (No. 4), a = 14.0207(11) Å, b = 5.7946(7) Å, c = 14.8367(11) Å, β = 103.512(8)°, α = γ = 90°, V = 1172.03(19) Å^3, T = 99.96(12) K, Z = 2, Z' = 1, μ(Cu K$_\alpha$) = 3.666, 10893 reflections measured, 4188 unique (R_{int} = 0.0736) which were used in all calculations. The final wR_2 was 0.1735 (all data) and R_1 was 0.0625 (I≥2 σ(I)).

Table S17. Crystal data and structure refinement for compound **15**.

**Compound**	**15**
Formula	$C_{21}H_{22}BrF_6O_3P$
$D_{calc.}$/ g cm^{-3}	1.551
μ/mm^{-1}	3.666
Formula Weight	547.275
Colour	colourless
Shape	needle-shaped
Size/mm^3	0.49×0.09×0.06
T/K	99.96(12)
Crystal System	monoclinic
Flack Parameter	0.020(19)
Hooft Parameter	0.020(19)
Space Group	$P2_1$
a/Å	14.0207(11)
b/Å	5.7946(7)
c/Å	14.8367(11)
α/°	90
β/°	103.512(8)
γ/°	90
V/Å^3	1172.03(19)
Z	2
Z'	1
Wavelength/Å	1.54184
Radiation type	Cu K$_\alpha$
Θ_{min}/°	3.24
Θ_{max}/°	73.30
Measured Refl's.	10893
Indep't Refl's	4188
Refl's I≥2 σ(I)	3472
R_{int}	0.0736
Parameters	418
Restraints	399
Largest Peak	0.8749
Deepest Hole	-0.8954
GooF	1.0331
wR_2 (all data)	0.1735
wR_2	0.1582
R_1 (all data)	0.0747
R_1	0.0625

Structure Quality Indicators

**Reflections:**	d min (Cu\a) 2Θ=146.6° 0.80	I/σ(I) 14.2	Rint m=2.58 7.36%	Full 135.4° 92% to 146.6° 97.4
**Refinement:**	Shift -0.005	Max Peak 0.9	Min Peak -0.9	GooF 1.033	Hooft .020(19)

A colourless needle-shaped-shaped crystal with dimensions 0.49 × 0.09 × 0.06 mm^3 was mounted on a loop with paratone. Data were collected using a XtaLAB Synergy, Dualflex, HyPix diffractometer operating at T = 99.96(12) K.

Data were measured using ω scans with Cu K_α radiation. The diffraction pattern was indexed and the total number of runs and images was based on the strategy calculation from the program CrysAlisPro 1.171.42.74a (Rigaku OD, 2022). The maximum resolution that was achieved was Θ = 73.30° (0.80 Å).

The unit cell was refined using CrysAlisPro 1.171.42.74a (Rigaku OD, 2022) on 2526 reflections, 23% of the observed reflections.

Data reduction, scaling and absorption corrections were performed using CrysAlisPro 1.171.42.74a (Rigaku OD, 2022). The final completeness is 98.89 % out to 73.30° in Θ. A gaussian absorption correction was performed using CrysAlisPro 1.171.42.74a (Rigaku Oxford Diffraction, 2022) Numerical absorption correction based on gaussian integration over a multifaceted crystal model Empirical absorption correction using spherical harmonics, implemented in SCALE3 ABSPACK scaling algorithm.. The absorption coefficient μ of this material is 3.666 mm^{-1} at this wavelength (λ = 1.54184Å) and the minimum and maximum transmissions are 0.429 and 0.942.

The structure was solved and the space group $P2_1$ (# 4) determined by the ShelXT 2018/2 (Sheldrick, 2018) structure solution program using using dual methods and refined by full matrix least squares minimisation on $\boldsymbol{F}^2$ using version of olex2.refine 1.5-alpha (Bourhis et al., 2015). All non-hydrogen atoms were refined anisotropically. Hydrogen atom positions were calculated geometrically and refined using the riding model. Most hydrogen atom positions were calculated geometrically and refined using the riding model, but some hydrogen atoms were refined freely.

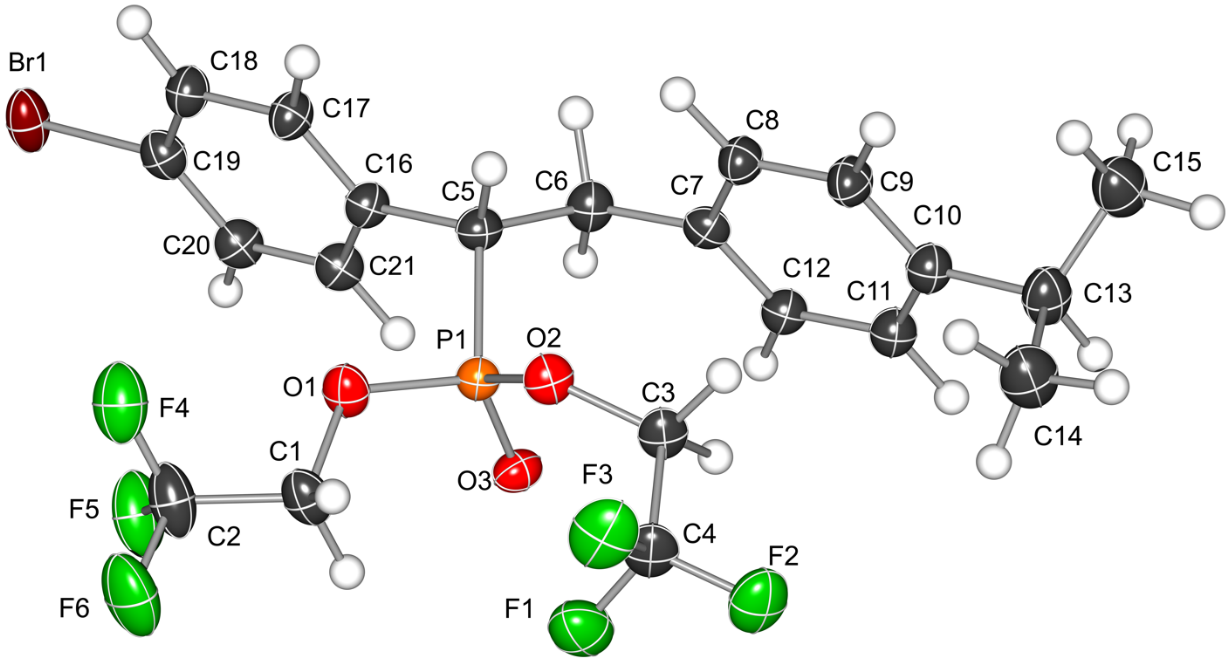

Figure S10:

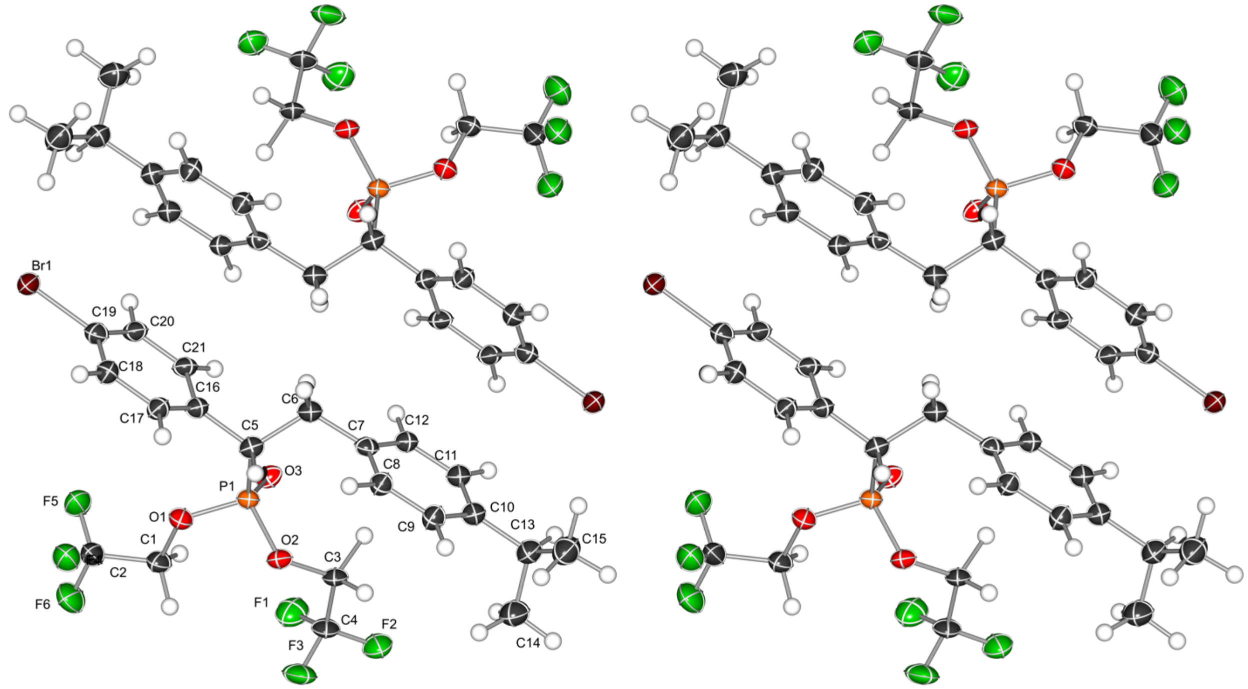

Figure S11:

_olex2_refine_details: Refinement using NoSpherA2, an implementation of NON-SPHERical Atom-form-factors in Olex2.Please cite: F. Kleemiss et al. Chem. Sci. DOI 10.1039/D0SC05526C - 2021NoSpherA2 implementation of HAR makes use of tailor-made aspherical atomic form factors calculated on-the-fly from a

Hirshfeld-partitioned electron density (ED) - not from spherical-atom form factors. The ED is calculated from a gaussian basis set single determinant SCF wave function - either Hartree-Fock or DFT using selected functionals - for a fragment of the crystal. This fragment can be embedded in an electrostatic crystal field by employing cluster charges or modelled using implicit solvation models, depending on the software used. The following options were used: SOFTWARE: ORCA PARTITIONING: NoSpherA2 INT ACCURACY: Normal METHOD: PBE BASIS SET: def2-SVP CHARGE: 0 MULTIPLICITY: 1 SOLVATION: Ethanol DATE: 2023-03-03_17-39-23

_*exptl_absorpt_process_details*: CrysAlisPro 1.171.42.74a (Rigaku Oxford Diffraction, 2022) Numerical absorption correction based on gaussian integration over a multifaceted crystal model Empirical absorption correction using spherical harmonics, implemented in SCALE3 ABSPACK scaling algorithm.

Data Plots: Diffraction Data

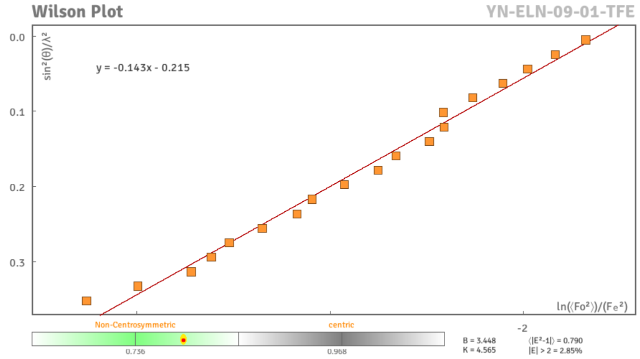

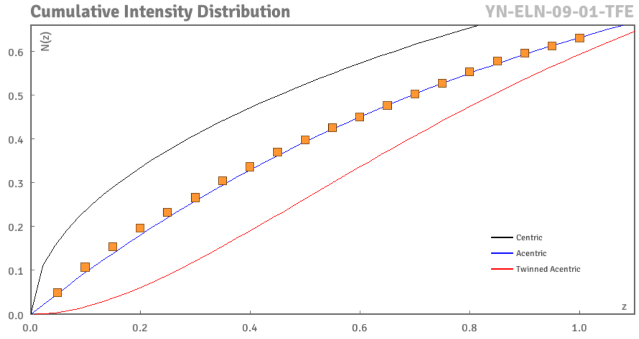

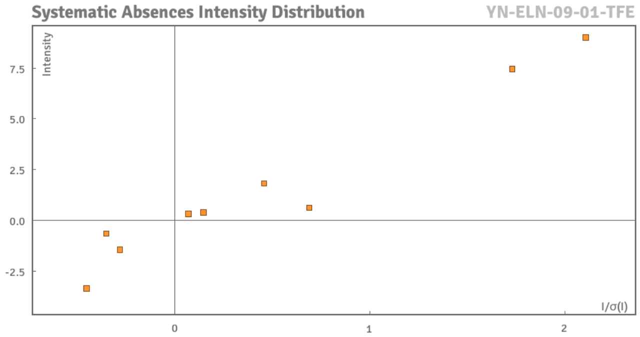

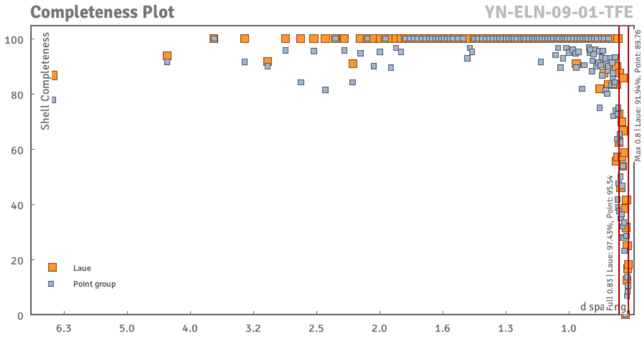

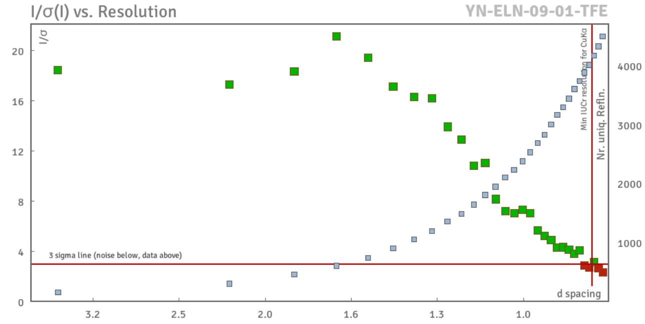

Data Plots: Refinement and Data

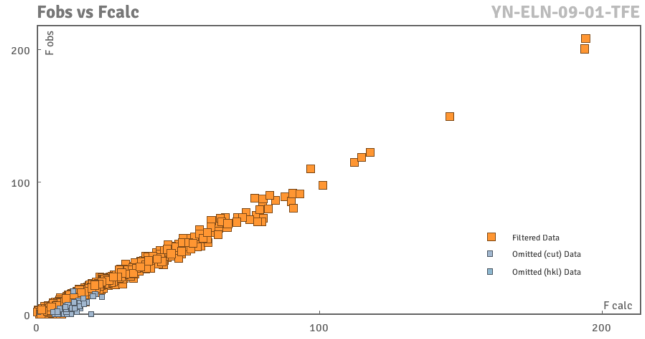

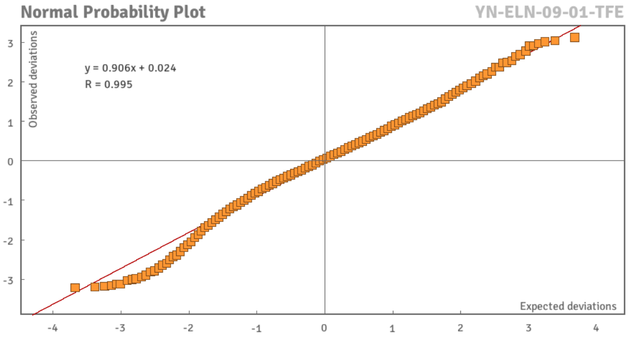

Reflection Statistics

Total reflections (after filtering)	10902	Unique reflections	4233
Completeness	0.898	Mean I/σ	9.9
hkl_{max} collected	(14, 7, 18)	hkl_{min} collected	(-17, -6, -16)
hkl_{max} used	(16, 7, 18)	hkl_{min} used	(-17, -6, 0)
Lim d_{max} collected	100.0	Lim d_{min} collected	0.77
d_{max} used	13.63	d_{min} used	0.8
Friedel pairs	1313	Friedel pairs merged	0
Inconsistent equivalents	27	R_{int}	0.0735
R_{sigma}	0.0707	Intensity transformed	0
Omitted reflections	0	Omitted by user (OMIT hkl)	40
Multiplicity	(2426, 1637, 777, 407, 161, 52, 18)	Maximum multiplicity	8
Removed systematic absences	9	Filtered off (Shel/OMIT)	0

Table S18: Fractional Atomic Coordinates (×10[4]) and Equivalent Isotropic Displacement Parameters (Å^2×10^3) for compound **15.** U_{eq} is defined as 1/3 of the trace of the orthogonalised U_{ij}.

Atom	x	y	z	U_{eq}
Br1	262.5(4)	7420.8(7)	4077.6(4)	49.1(2)
P1	4567.0(10)	4563(2)	7432.2(9)	29.9(3)
F2	7196(3)	1869(7)	9747(3)	62.1(12)
O3	4799(3)	2312(7)	7090(3)	37.0(8)
O1	3567(3)	4696(7)	7736(3)	41.2(10)
O2	5316(3)	5329(7)	8366(3)	37.3(9)
F1	5690(4)	956(7)	9222(3)	65.3(12)
F3	6065(4)	3760(7)	10174(3)	67.0(13)
F5	1823(3)	2140(8)	7508(3)	71.7(13)
F4	1890(3)	5184(8)	8345(3)	69.4(12)
F6	2069(4)	1830(8)	8992(3)	81.9(16)
C7	6285(4)	6759(9)	6608(4)	32.5(11)
C11	7859(4)	4834(11)	7069(4)	37.3(12)
C20	2118(5)	5288(11)	4814(4)	38.3(12)
C5	4449(4)	6940(9)	6621(4)	32.5(12)
C9	7639(5)	8541(11)	7706(5)	40.0(13)
C19	1534(4)	7228(12)	4848(4)	38.3(11)
C8	6671(4)	8609(11)	7185(4)	36.9(12)

Atom	x	y	z	U_{eq}
C12	6887(4)	4897(10)	6537(4)	34.0(12)
C21	3056(4)	5192(10)	5378(4)	35.9(12)
C16	3418(4)	6997(10)	5997(4)	33.3(11)
C17	2826(4)	8912(10)	6011(4)	36.1(12)
C6	5236(4)	6828(11)	6055(4)	38.7(14)
C4	6300(5)	2717(13)	9461(4)	45.9(12)
C10	8253(5)	6639(10)	7653(4)	37.8(12)
C18	1879(4)	9035(11)	5451(4)	38.7(13)
C1	3368(5)	3162(11)	8422(4)	43.1(14)
C3	6271(4)	4368(11)	8657(4)	37.6(12)
C13	9287(5)	6495(13)	8245(5)	49.0(14)
C2	2270(5)	3104(14)	8305(5)	56.9(14)
C14	9286(6)	5722(14)	9208(5)	60.5(19)
C15	9877(6)	8639(13)	8217(6)	60.8(19)

Table S19: Anisotropic Displacement Parameters (×10^4) for compound **15.** The anisotropic displacement factor exponent takes the form: $-2\pi^2[h^2a^{*2} \times U_{11}+ ... +2hka^* \times b^* \times U_{12}]$

Atom	U_{11}	U_{22}	U_{33}	U_{23}	U_{13}	U_{12}
Br1	32.0(3)	81.3(5)	32.0(3)	-5.9(3)	3.3(2)	6.5(4)
P1	31.7(7)	34.5(7)	23.3(6)	0.1(6)	6.1(5)	0.1(6)
F2	56.2(19)	86(3)	41(2)	14.5(16)	5.3(13)	17.7(19)
O3	40.2(19)	32(2)	35.1(19)	4(2)	1.9(15)	1(2)
O1	38(2)	56(3)	30.3(19)	-0.7(17)	9.6(15)	5.8(17)
O2	40.2(19)	47(2)	22.4(18)	-1.5(15)	2.3(13)	-1.9(16)
F1	66(3)	69(3)	57(3)	-5.7(16)	7.0(18)	19.2(17)
F3	78(3)	98(3)	26.5(18)	14(2)	14.4(15)	7.7(16)
F5	46(2)	118(4)	49.5(19)	-17(2)	7.4(14)	-23.2(19)
F4	52(2)	107(3)	53(2)	-1.3(17)	17.2(18)	-13.3(18)
F6	62(3)	129(5)	61(2)	-22(2)	28.0(17)	6(2)
C7	38(2)	36(2)	25(2)	-1.0(12)	8.1(13)	4.9(14)
C11	37(2)	44(3)	30(2)	1.6(14)	6.3(15)	0.8(16)
H11	46(9)	54(8)	50(20)	11(4)	-2(6)	-11(6)
C20	41(2)	47(3)	25(2)	-3.4(14)	4.1(15)	-1.1(17)
H20	49(11)	54(12)	60(30)	10(5)	-10(8)	-22(9)
C5	36(2)	33(3)	28(2)	-0.2(14)	4.4(13)	0.7(16)
H5	42(11)	33(7)	27(13)	-3(3)	8(4)	1(5)
C9	35(2)	42(3)	41(3)	-0.9(14)	4.0(16)	-2.7(18)
H9	46(9)	54(8)	50(20)	11(4)	-2(6)	-11(6)
C19	39(2)	48(3)	29(2)	-5.0(13)	9.2(13)	1.8(16)
C8	33(2)	38(3)	38(3)	0.5(14)	5.0(16)	-0.7(16)
H8	46(9)	54(8)	50(20)	11(4)	-2(6)	-11(6)
C12	35(2)	38(3)	28(2)	0.4(13)	5.4(15)	0.3(17)
H12	46(9)	54(8)	50(20)	11(4)	-2(6)	-11(6)
C21	38(2)	40(3)	28(2)	-2.0(14)	3.8(15)	-3.2(16)
H21	49(11)	54(12)	60(30)	10(5)	-10(8)	-22(9)
C16	34(2)	37(3)	27(2)	0.4(13)	4.0(13)	-0.0(14)
C17	33(2)	39(3)	33(3)	1.6(14)	3.2(16)	-2.5(16)
H17	37(12)	41(11)	40(30)	4(5)	-1(8)	-6(8)
C6	38(2)	51(4)	26(2)	1.4(14)	6.8(13)	1.5(17)

Atom	U_{11}	U_{22}	U_{33}	U_{23}	U_{13}	U_{12}
H6a	43(11)	54(6)	33(8)	1(3)	7(4)	7(3)
H6b	37(10)	51(6)	27(8)	2(3)	6(4)	1(3)
C4	49(2)	63(3)	26(2)	3.3(13)	8.6(12)	16.0(14)
C10	36(2)	45(3)	31(2)	1.3(13)	6.3(15)	1.4(15)
C18	33(2)	47(3)	35(3)	2.0(14)	6.4(15)	-1.0(17)
H18	42(12)	62(16)	60(40)	14(7)	-13(10)	-23(12)
C1	41(2)	61(4)	28(2)	-8.0(16)	8.8(13)	4.3(18)
H1a	44(11)	61(5)	31(11)	-8(3)	14(4)	6(3)
H1b	42(10)	65(11)	29(4)	-9(4)	9(2)	3(3)
C3	38(2)	53(3)	20(2)	0.8(16)	3.8(14)	6.9(16)
H3a	43(11)	53(9)	22(6)	0(4)	7(3)	6(3)
H3b	41(7)	54(7)	16(11)	-1(3)	3(3)	8(3)
C13	40(3)	64(3)	39(3)	1.3(15)	1.8(16)	5.8(19)
H13	40(9)	65(6)	42(10)	2(3)	3(4)	7(3)
C2	40(2)	95(3)	38(2)	-13.2(15)	13.6(12)	-11.7(16)
C14	58(4)	76(5)	43(3)	-4(3)	2.7(19)	9(2)
H14a	60(9)	80(10)	44(11)	-2(4)	4(4)	9(4)
H14b	60(12)	77(6)	48(12)	-5(4)	3(5)	10(3)
H14c	58(5)	77(13)	47(9)	-4(4)	1(3)	9(4)
C15	47(4)	71(4)	58(4)	-5(2)	-1(3)	12(2)
H15a	47(12)	77(12)	58(5)	-5(5)	0(3)	13(3)
H15b	45(11)	72(7)	57(11)	-5(4)	-3(5)	11(4)
H15c	47(4)	77(13)	62(10)	-4(3)	-2(3)	12(5)

Table 4: Bond Lengths in Å for compound **15**.

Atom	Atom	Length/Å
Br1	C19	1.883(6)
P1	O3	1.464(4)
P1	O1	1.572(4)
P1	O2	1.594(4)
P1	C5	1.811(5)
F2	C4	1.324(8)
O1	C1	1.427(7)
O2	C3	1.421(7)
F1	C4	1.325(8)
F3	C4	1.324(8)
F5	C2	1.325(8)
F4	C2	1.324(9)
F6	C2	1.341(9)
C7	C8	1.400(8)
C7	C12	1.389(8)
C7	C6	1.508(8)
C11	H11	1.084(3)
C11	C12	1.406(8)
C11	C10	1.388(9)
C20	H20	1.040(3)
C20	C19	1.398(9)
C20	C21	1.385(9)
C5	H5	1.0831(16)

Atom	Atom	Length/Å
C5	C16	1.524(8)
C5	C6	1.537(8)
C9	H9	1.084(3)
C9	C8	1.396(9)
C9	C10	1.411(9)
C19	C18	1.388(9)
C8	H8	1.084(3)
C12	H12	1.084(3)
C21	H21	1.040(3)
C21	C16	1.407(8)
C16	C17	1.389(8)
C17	H17	1.040(3)
C17	C18	1.395(9)
C6	H6a	1.0830(11)
C6	H6b	1.0830(11)
C4	C3	1.523(8)
C10	C13	1.512(9)
C18	H18	1.040(3)
C1	H1a	1.0830(13)
C1	H1b	1.0830(13)
C1	C2	1.509(9)
C3	H3a	1.0831(19)
C3	H3b	1.0831(19)
C13	H13	1.0831(19)
C13	C14	1.498(10)
C13	C15	1.498(10)
C14	H14a	1.0830(19)
C14	H14b	1.0830(19)
C14	H14c	1.0830(19)
C15	H15a	1.0831(19)
C15	H15b	1.0831(19)
C15	H15c	1.0831(19)

Table S21: Bond Angles in ° for compound **15.**

Atom	Atom	Atom	Angle/°
O1	P1	O3	115.4(2)
O2	P1	O3	113.3(2)
O2	P1	O1	100.8(2)
C5	P1	O3	116.1(2)
C5	P1	O1	101.8(2)
C5	P1	O2	107.8(2)
C1	O1	P1	120.7(4)
C3	O2	P1	122.5(4)
C12	C7	C8	119.1(5)
C6	C7	C8	120.1(5)
C6	C7	C12	120.8(5)
C12	C11	H11	119.3(3)
C10	C11	H11	119.3(4)
C10	C11	C12	121.5(6)

Atom	Atom	Atom	Angle/°
C19	C20	H20	120.3(3)
C21	C20	H20	120.3(4)
C21	C20	C19	119.4(6)
H5	C5	P1	107(3)
C16	C5	P1	110.3(4)
C16	C5	H5	111(3)
C6	C5	P1	111.4(4)
C6	C5	H5	105(4)
C6	C5	C16	111.6(4)
C8	C9	H9	119.4(4)
C10	C9	H9	119.4(4)
C10	C9	C8	121.2(6)
C20	C19	Br1	120.3(5)
C18	C19	Br1	118.9(5)
C18	C19	C20	120.8(5)
C9	C8	C7	120.3(6)
H8	C8	C7	119.9(3)
H8	C8	C9	119.9(4)
C11	C12	C7	120.3(5)
H12	C12	C7	119(4)
H12	C12	C11	120(4)
H21	C21	C20	119.6(4)
C16	C21	C20	120.8(6)
C16	C21	H21	119.6(3)
C21	C16	C5	121.9(5)
C17	C16	C5	119.6(5)
C17	C16	C21	118.5(5)
H17	C17	C16	119.3(3)
C18	C17	C16	121.4(5)
C18	C17	H17	119.3(3)
C5	C6	C7	115.9(5)
H6a	C6	C7	108.3(3)
H6a	C6	C5	108.3(3)
H6b	C6	C7	108.3(3)
H6b	C6	C5	108.3(3)
H6b	C6	H6a	107.4
F1	C4	F2	107.8(6)
F3	C4	F2	107.9(5)
F3	C4	F1	107.5(5)
C3	C4	F2	109.5(5)
C3	C4	F1	112.1(5)
C3	C4	F3	111.9(6)
C9	C10	C11	117.7(6)
C13	C10	C11	120.6(6)
C13	C10	C9	121.7(6)
C17	C18	C19	119.0(6)
H18	C18	C19	120.5(4)
H18	C18	C17	120.5(3)
H1a	C1	O1	110.3(3)
H1b	C1	O1	110.3(3)
H1b	C1	H1a	108.5
C2	C1	O1	107.3(5)

Atom	Atom	Atom	Angle/˚
C2	C1	H1a	110.3(4)
C2	C1	H1b	110.3(3)
C4	C3	O2	109.5(5)
H3a	C3	O2	109.8(3)
H3a	C3	C4	109.8(4)
H3b	C3	O2	109.8(3)
H3b	C3	C4	109.8(3)
H3b	C3	H3a	108.2
H13	C13	C10	105(3)
C14	C13	C10	110.8(6)
C14	C13	H13	101(4)
C15	C13	C10	113.5(6)
C15	C13	H13	112(3)
C15	C13	C14	113.5(7)
F4	C2	F5	108.2(6)
F6	C2	F5	107.9(6)
F6	C2	F4	108.0(5)
C1	C2	F5	111.6(5)
C1	C2	F4	112.6(6)
C1	C2	F6	108.3(6)
H14a	C14	C13	109.5
H14b	C14	C13	109.5
H14b	C14	H14a	109.5
H14c	C14	C13	109.5
H14c	C14	H14a	109.5
H14c	C14	H14b	109.5
H15a	C15	C13	109.5
H15b	C15	C13	109.5
H15b	C15	H15a	109.5
H15c	C15	C13	109.5
H15c	C15	H15a	109.5
H15c	C15	H15b	109.5

Table *S*22: Torsion Angles in ˚ for compound **15.**

Atom	Atom	Atom	Atom	Angle/˚
Br1	C19	C20	C21	179.2(4)
Br1	C19	C18	C17	-179.0(4)
P1	O1	C1	C2	-159.5(5)
P1	O2	C3	C4	-105.9(5)
P1	C5	C16	C21	-63.8(5)
P1	C5	C16	C17	118.4(4)
P1	C5	C6	C7	-56.1(4)
F2	C4	C3	O2	-178.3(5)
O1	C1	C2	F5	66.6(6)
O1	C1	C2	F4	-55.4(6)
O1	C1	C2	F6	-174.7(5)
O2	C3	C4	F1	62.1(5)
O2	C3	C4	F3	-58.8(5)
C7	C8	C9	C10	1.0(7)

Atom	Atom	Atom	Atom	Angle/˚
C7	C12	C11	C10	-2.0(6)
C7	C6	C5	C16	-179.9(5)
C11	C10	C9	C8	-0.3(7)
C11	C10	C13	C14	-97.2(7)
C11	C10	C13	C15	133.7(7)
C20	C19	C18	C17	1.3(6)
C20	C21	C16	C5	-179.7(5)
C20	C21	C16	C17	-1.9(7)
C5	C16	C17	C18	180.0(5)
C9	C10	C13	C14	79.7(7)
C9	C10	C13	C15	-49.4(7)
C19	C18	C17	C16	-1.8(7)
C21	C16	C17	C18	2.1(6)

Table S23: Hydrogen Fractional Atomic Coordinates (×10[4]) and Equivalent Isotropic Displacement Parameters (Å[2]×10[3]) for compound **15.** U_{eq} is defined as 1/3 of the trace of the orthogonalised U_{ij}.

Atom	x	y	z	U_{eq}
H11	8311(5)	3342(11)	7021(4)	53(10)
H20	1849(5)	3928(11)	4370(4)	58(16)
H5	4610(50)	8500(50)	7030(30)	34(7)
H9	7925(5)	9974(11)	8158(5)	53(10)
H8	6218(4)	10100(11)	7229(4)	53(10)
H12	6590(50)	3460(80)	6090(40)	53(10)
H21	3497(5)	3768(11)	5342(4)	58(16)
H17	3095(4)	10284(11)	6450(4)	41(16)
H6a	5148(4)	8316(11)	5601(4)	44(5)
H6b	5102(4)	5306(11)	5620(4)	39(5)
H18	1437(5)	10460(11)	5484(4)	60(20)
H1a	3641(5)	1451(11)	8330(4)	44(6)
H1b	3722(5)	3768(11)	9109(4)	45(6)
H3a	6458(4)	3451(11)	8085(4)	40(6)
H3b	6803(4)	5733(11)	8871(4)	38(5)
H13	9610(40)	5000(70)	7990(40)	50(6)
H14a	8808(6)	6832(14)	9492(5)	63(6)
H14b	9023(6)	3960(14)	9189(5)	63(6)
H14c	10024(6)	5813(14)	9638(6)	62(6)
H15a	9864(6)	9067(13)	7503(6)	63(6)
H15b	9567(6)	10050(14)	8533(6)	60(6)
H15c	10628(6)	8350(13)	8593(6)	64(6)

Bibliography

[1] CrysAlisPro (Rigaku, V1.171.42.74a, 2022)

[2] L.J. Bourhis and O.V. Dolomanov and R.J. Gildea and J.A.K. Howard and H. Puschmann, The Anatomy of a Comprehensive Constrained, Restrained, Refinement Program for the Modern Computing Environment - Olex2 Disected, *Acta Cryst. A*, (2015), **A71**, 59-71.

[3] O.V. Dolomanov and L.J. Bourhis and R.J. Gildea and J.A.K. Howard and H. Puschmann, Olex2: A complete structure solution, refinement and analysis program, *J. Appl. Cryst.*, (2009), **42**, 339-341.

[4] Sheldrick, G.M., ShelXT-Integrated space-group and crystal-structure determination, *Acta Cryst.*, (2015), **A71**, 3-8.

[5] CrysAlisPro (Rigaku, V1.171.41.108a, 2021)

[6] O.V. Dolomanov and L.J. Bourhis and R.J. Gildea and J.A.K. Howard and H. Puschmann, Olex2: A complete structure solution, refinement and analysis program, *J. Appl. Cryst.*, (2009), **42**, 339-341.

[7] Sheldrick, G.M., Crystal structure refinement with ShelXL, *Acta Cryst.*, (2015), **C71**, 3-8.

[8] CrysAlisPro (Rigaku, V1.171.41.116a, 2021)

[9] CrysAlisPro (ROD), Rigaku Oxford Diffraction, Poland (?).
